# Research Communications of the 32^nd^ ECVIM‐CA Online Congress

**DOI:** 10.1111/jvim.16559

**Published:** 2022-11-03


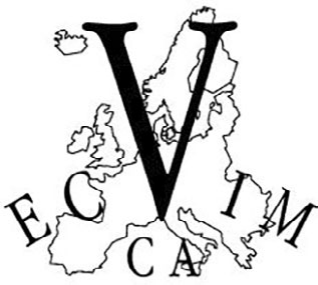
 **RESEARCH COMMUNICATIONS OF THE 32** ^**nd**^ **ECVIM‐CA CONGRESS**

**1‐3 September 2022**

**Swedish Exhibition and Congress Centre**

**Göteborg, Sweden**

*The European College of Veterinary Internal Medicine – Companion Animals (ECVIM‐CA) Congress and the Journal of Veterinary Internal Medicine (JVIM) are not responsible for the content or dosage recommendations in the abstracts. The abstracts are not peer reviewed before publication. The opinions expressed in the abstracts are those of the author(s) and may not represent the views or position of the ECVIM‐CA. The authors are solely responsible for the content of the abstracts*.

## 1 ESVC‐O‐1

### 1.1 ESVC ‐ European Society of Veterinary Cardiology

#### 1.1.1 Long‐term results of mitral valve repair with artificial chordae and annuloplasty in small‐breed dogs

##### 1.1.1.1 K. Kurogochi, M. Uechi

###### 1.1.1.1.1 JASMINE veterinary cardiovascular medical center Yokohama‐city Japan

Myxomatous mitral valve degeneration (MMVD) is the most common heart disease in small‐breed dogs. Dogs with severe MMVD often develop congestive heart failure (CHF), resulting in a median survival time of nine to twelve months after diagnosis with medical management. Mitral valve repair (MVR) is an alternative treatment option for advanced MMVD, but the long‐term outcome in dogs is poorly reported. Therefore, this study investigates how prolonged survival can be achieved with MVR.

This was a retrospective study of dogs with MMVD that underwent MVR from February 2017 to February 2019. Four hundred and forty‐four dogs were classified into three groups: the non‐CHF group that met the criteria of the EPIC‐study without heart failure (equivalent to ACVIM stage B2); the chronic‐CHF group that had a history of heart failure (equivalent to ACVIM stage C); and the severe‐CHF group that was defined using our proposed scoring system, which is based on the refractory condition to standard treatments and poor prognostic findings among chronic‐CHF (equivalent to ACVIM stage D). Values are expressed as median [interquartile range].

The number of cases of non‐CHF, chronic‐CHF, and severe‐CHF was 132, 264, and 48, respectively. The heart size was significantly lowered after MVR in all groups: the vertebral heart size was 11.6 [1.4] to 10.6 [1.3] (*p* < 0.01), the left atrium‐to‐aortic ratio was 2.09 [0.63] to 1.51 [0.35] (*p* < 0.01), and the normalized left ventricular internal dimension in diastole was 2.13 [0.38] to 1.63 [0.33] (*p* < 0.01). The three‐year survival rates for all‐cause mortality derived by Kaplan‐Meier curves were 80.8% for the non‐CHF, 73.2% for the chronic‐CHF, and 56.3% for the severe‐CHF groups (*p* = 0.001: Log‐rank test).

In conclusion, MVR appears to be an effective treatment for MMVD and can be applied to all advanced clinical stages. Furthermore, surgical intervention at an earlier stage is preferable for long‐term prognosis.

**Disclosures**

No disclosures to report

## 2 ESVC‐O‐2

### 2.1 ESVC ‐ European Society of Veterinary Cardiology

#### 2.1.1 Differences in mitral valve morphology between Cavalier King Charles Spaniels with mild mitral regurgitation and without mitral regurgitation.

##### 2.1.1.1 G. Menciotti, M. Borgarelli, A. Franchini, S.M. Lahmers, H.W. Jeong

###### 2.1.1.1.1 VA‐MD College of Veterinary Medicine Blacksburg United States

Mitral valve (MV) morphology is an important determinant of leaflet stress which in turn has been implicated in the pathogenesis of the most common canine cardiac disease, myxomatous valvular degeneration (MMVD). Particularly, MMVD is extremely prevalent in Cavalier King Charles Spaniels (CKCS) at a young age. In this study, we used transthoracic three‐dimensional echocardiography (3DTTE) to evaluate the MV morphology of CKCS with low‐grade murmurs (<3/6) and no more than mild mitral regurgitation (MR). We hypothesized that CKCS with mild MR (MR+), would have a MV of different morphology compared to CKCS without MR (MR‐). MR was quantified subjectively by color Doppler evaluation of the MV in all standard echocardiographic planes. Full‐volume 4‐beat 3DTTE datasets of the MV were acquired during the same examination and analyzed using dedicated software. Eighty dogs were prospectively enrolled; 41 had no murmur, 13 had a grade 1/6 murmur, 26 had a grade 2/6 murmur. The MV of MR+ was significantly different from MR‐. Particularly, MR+ had bigger annular diameters, circumference, and area (p<0.0001). Both leaflets had larger areas (p<0.01) and length in MR+ (p<0.05). The angle between both anterior and posterior leaflets and the annulus was reduced in MR+ (p<0.05). These findings indicate that CKCS with only mild MR have MV morphological differences compared to CKCS without MR. This further supports previous research suggesting a role of MV morphology in the pathophysiology of MMVD, and further investigation of a causative link is warranted.

**Disclosures**

Disclosures to report, please report below

A. Franchini's assistantship is partly funded by Ceva Sante Animale M. Borgarelli receives research support by Ceva Sante Animale M. Borgarelli act as consultant for Ceva Sante Animale

## 3 ESVC‐O‐3

### 3.1 ESVC ‐ European Society of Veterinary Cardiology

#### 3.1.1 Analysis of pimobendan prescriptions in small breed dogs with myxomatous mitral valve disease using Electronic Health Records in Primary Care Veterinary Practices in the United Kingdom

##### 3.1.1.1 M. Silva, E. Bode, D. Singleton, M. Hezzell , H. Hodgkiss‐Geere

###### 3.1.1.1.1 Leamington spa United Kingdom

Myxomatous mitral valve disease (MMVD) is the most common cardiovascular disease reported in dogs and is often managed in primary‐care veterinary practices. The use of pimobendan in MMVD has been reported to delay the onset of congestive heart failure by 15 months, as described in the EPIC study. This study aimed to determine whether pimobendan prescription increased following market authorization in 2017, describe the population characteristics and use of diagnostic tests reported in electronic health records (EHRs) from UK primary veterinary practices compared to those used in the EPIC study.

EHRs describing 2,945,591 consultations (861,814 unique dogs) were gathered (between 22nd November 2013 and 31st December 2018) by the Small Animal Veterinary Surveillance Network (SAVSNET) from 224 veterinary practices. Free‐text clinical narratives were manually read to identify first pimobendan prescriptions. Dogs were excluded if in the 6 months prior to the prescription of pimobendan they were: prescribed any medication other than pimobendan, diagnosed with a systemic disease, congenital or acquired cardiac disease, or had a pregnancy diagnosis. Diagnostic tests were categorised as “recommended” if these were advised before pimobendan prescription, and “suggested” if these were not deemed necessary for pimobendan prescription. Annual variation in pimobendan prescriptions were analysed using mixed effects binomial regression models.

Pimobendan prescriptions increased in 2018 vs. 2017 (p<0.001). 110 dogs met the inclusion criteria. 59 female (53.6%), 51 male (46.4%), 50 Cavalier King Charles spaniels (45.5%), 12 crossbreed (10.9%) and 11 Jack Russell terriers (10%). Recorded murmur grades were as follows: 1 (n=7, 6.4%), 2 (n=21, 19.1%), 3 (n=38, 34.5%), 4 (n=25, 22.7%), 5 (n=16, 14.5%), 6 (n=3, 2.7%). Recorded presenting complaints included cough (n=41, 37.3%), exercise intolerance (n=16, 14.5%), syncope (n=9, 8.2%). Performed and recorded diagnostic tests prior to pimobendan prescription were as follows: echocardiography (n=36, 32.7%), thoracic radiography (n=5, 4.5%), NT‐proBNP measurement (n=4, 3.6%), while 6 were referred for further evaluation (5.4%). Recorded recommendations included echocardiography (n=17, 15.4%), thoracic radiography (n=6, 5.4%) and referral (n=5, 4.5%). 7 had echocardiography suggested (6.3%). In 34 dogs, no diagnostic tests were recorded as being offered (30.9%).

Considering that the conclusions of the EPIC study are only relevant for dogs with significant cardiac remodelling, most dogs still had pimobendan prescribed without recorded evidence of assessment of cardiomegaly, either by echocardiography or thoracic radiography. Treatment with pimobendan is expensive and prescribing it to patients who might not benefit from it could impose an unnecessary economic burden on the client.

**Disclosures**

Disclosures to report, please report below

Hannah Hodgkiss‐Geere: no active disclosures but previously been awarded grants from BSAVA Petsavers and also Petplan Melanie Hezzell: previous or current research funding from PetSavers, the Langford Trust, Bristol Veterinary School, the American Kennel Club Canine Health Foundation, ACVIM, Cavalier Matters, Ceva Animal Health, Boehringer Ingelheim and IDEXX Laboratories

## 4 ESVC‐O‐4

### 4.1 ESVC ‐ European Society of Veterinary Cardiology

#### 4.1.1 Routine echocardiography in healthy senior cats. How reliable are left ventricular wall measurements?

##### 4.1.1.1 A. Pires, S. Raheb, W. Sears, A. Chong, S. Fonfara

###### 4.1.1.1.1 University of Guelph Guelph Canada

Echocardiographic reference intervals for cats have commonly been obtained from young individuals. Whether these apply to older cats is unknown. Furthermore, little agreement exists with respect to how left ventricular (LV) wall measurements should be obtained. Nonetheless, low inter‐ and intraobserver variabilities have been reported. Our hypothesis was that published echocardiographic normal values are of limited use in older cats. We further hypothesized that the comparability of diastolic LV wall measurements taken by different observers is poor. The objective of our study was to obtain echocardiographic normal values for healthy cats > 9 years of age, and to determine the interobserver reliability between veterinary cardiologists from different institutions measuring the interventricular septum (IVS).

78 healthy cats with an age range of 9.5‐18 years were prospectively recruited for echocardiography. 3 cats (3.9%) with diastolic LV wall measurements between 6.5‐7.4 mm were diagnosed with hypertrophic cardiomyopathy (HCM) and excluded from the study. For 75 cats, diastolic LV free wall and IVS measured between 2.0‐6.3 mm. 27 cats (36%) measured between 5.5‐6.3 mm. For these cats, echocardiography is repeated yearly. An IVS bulge, false tendons, and diastolic impairment were common findings.

From this population of cats, one echocardiographic still picture of the IVS from four individuals were selected. 30 observers from six countries performed five IVS measurements for each picture. The influence of picture, region of the IVS, and observer was investigated applying intraclass correlation coefficient (ICC). An ICC of 0.45 (95% confidence interval 0.38‐0.56) was observed for the base of the IVS and of 0.55 (95% confidence interval 0.50‐0.63) for the remainder of the IVS.

The results of the present study identified a high variability in LV geometry and diastolic LV wall thickness between cats > 9 years old. 36% of this cat population had diastolic wall measurements that are considered diagnostic for HCM applying published reference values. This suggests that specific reference values for older cats might be required. Furthermore, the low interobserver reliability questions the use of echocardiographic LV wall measurements to diagnose HCM.

**Disclosures**

No disclosures to report

## 5 ESVC‐O‐5

### 5.1 ESVC ‐ European Society of Veterinary Cardiology

#### 5.1.1 Genes of inflammation and coagulation are activated in hearts from cats with hypertrophic cardiomyopathy

##### 5.1.1.1 E. Colpitts, J. Caswell , L. O'Sullivan, S. Raheb, S. Fonfara

###### 5.1.1.1.1 Ontario Veterinary College Guelph Canada

Left atrial (LA) thrombus formation is a complication of hypertrophic cardiomyopathy (HCM) in cats. Little is known as to why LA thrombi develop in some cats with HCM.

We hypothesized that selected factors for coagulation, endothelial activation, inflammation, and remodelling are activated in the hearts of cats with HCM.

Left atrial and ventricular (LV) samples from 12 cats with HCM (five of which had LA thrombi), and six young (YC) and six adult (AC) control cats without cardiac disease were investigated for relative expression of the following genes using quantitative polymerase chain reaction: von Willebrand factor (vWF), a disintegrin and metalloproteinase with a thrombospondin type 1 motif member 13, platelet activating factor (PAF), E‐ and P‐selectin, intercellular and vascular adhesion molecules‐1, ß2‐integrin, vascular endothelial growth factor (VEGF), interleukin‐6 (IL‐6), monocyte chemoattractant protein‐1 (MCP‐1), heat shock protein‐70, and myocyte‐specific enhancer factor 2C.

Cats with HCM showed higher LA IL‐6 (p=0.041), MCP‐1 (p=0.005), and vWF (p=0.032) and lower VEGF (p=0.001) expression than AC cats. For LV samples, cats with HCM exhibited higher MCP‐1 (p=0.005) and PAF (p=0.039) expression in comparison to AC cats. The presence of an LA thrombus in cats with HCM was associated with higher LA IL‐6 (p=0.018) expression. Young control cats showed significantly lower gene expression than AC and cats with HCM.

This study further supports the involvement of inflammation and coagulation in HCM and the influence of age on myocardial gene expression. While IL‐6 appears to be associated with LA thrombi in feline HCM, markers of coagulation and endothelial activation might not play a role.

**Disclosures**

No disclosures to report

## 6 ESVC‐O‐6

### 6.1 ESVC ‐ European Society of Veterinary Cardiology

#### 6.1.1 Genome wide association study of congenital pulmonic stenosis in French Bulldogs

##### 6.1.1.1 M.L. Toom ^1^, V. Szatmári ^1^, K. Borgeat ^2^, N. Beijerink ^3^, F. Steenbeek ^1^

###### 6.1.1.1.1 ^1^ Faculty of Veterinary Medicine, University of Utrecht Utrecht Netherlands, ^2^ Small Animal Hospital, Langford Vets, University of Bristol Bristol United Kingdom; ^3^ Veterinary Specialists Vught Netherlands

Pulmonic stenosis (PS) is the most commonly diagnosed canine congenital heart disease in Europe, with a strong breed predisposition in the French bulldog. Pedigree analysis suggests an autosomal recessive mode of inheritance. The aim of this study was to identify genes associated with PS in French bulldogs. French bulldogs diagnosed with PS and a minimum Doppler‐derived pressure gradient of 50 mmHg (non‐sedated) were eligible for inclusion. Diagnosis of concurrent heart disease (except patent *foramen ovale*) was an exclusion criterion. A control group of French bulldogs with a normal echocardiogram were also recruited. Whole blood was used to isolate DNA in 47 affected dogs and 49 controls, and genotyping was performed using the Illumina 170K SNP‐chip. A genome‐wide association study (GWAS) was performed using PLINK. After quality control, 45 cases and 40 controls were included in GWAS, in which 156,582 SNPs passed the thresholds for missingness and minor allele frequency. Mapping pointed towards the possible involvement of a region on canine chromosome 34, with the most associated SNPs (p=1.1*10^‐6^) being located between 10.0 and 10.5 Mbp. This chromosome region contains two members of the Iroquois homeobox gene family, namely *IRX2* and *IRX4*. The *IRX2* gene is known to be associated with neuron differentiation and metanephros development. The *IRX4* gene is involved in cardiac development, and thought to play a role in the development of tetralogy of Fallot in humans. Since PS is a component of tetralogy, these findings in French bulldogs show promise for further study in both dogs and humans.

**Disclosures**

No disclosures to report

This study was funded by the ESVC clinical study fund.

## 7 ESVC‐O‐7

### 7.1 ESVC ‐ European Society of Veterinary Cardiology

#### 7.1.1 Agreement between different 2‐D transthoracic views and 3‐D transthoracic echocardiography in identifying mitral valve prolapse in dogs

##### 7.1.1.1 A. Franchini, M. Borgarelli, G. Menciotti, H. Jeong, S. Lahmers

###### 7.1.1.1.1 Virginia‐Maryland College of Veterinary Medicine Blacksburg United States

This study aimed to evaluate the agreement between different 2‐dimensional transthoracic echocardiography (2D‐TTE) views and 3‐dimensional TTE (3D‐TTE) in identifying anterior (ALP) and posterior mitral valve leaflet prolapse (PLP) in dogs.

A convenient sample of dogs with echocardiographic evidence of myxomatous mitral valve disease (MMVD) was retrospectively selected. To be included, a right parasternal four‐chamber view (RP4C), a right parasternal five‐chamber view (RP5C), a left apical four‐chamber (LA4C), and a full‐volume 3‐D dataset must have been available for review. A control group of healthy dogs was also selected. On 2D‐TTE, prolapse was defined as a portion of the mitral valve (MV) bulging on the atrial side of a line traced at the MV annulus level. On 3D‐TTE, prolapse was automatically identified by dedicated MV analysis software as a portion of MV protruding above MV annulus.

Cohen's kappa coefficient (k) was used to evaluate agreement in presence/absence of ALP and PLP between different 2D‐TTE views and 3D‐TTE.

Twenty dogs in different MMVD stages and ten healthy dogs were selected.

Agreement between RP4C and 3D‐TTE for ALP was almost perfect (k=0.92), while it was strong for PLP (k=0.89). Agreement between RP5C and 3D‐TTE was almost perfect for ALP (k=0.93) and PLP (k=1). Lastly, agreement between LA4C and 3D‐TTE for ALP was almost perfect (k=0.92), while it was moderate for the PLP (k=0.71).

The RP5C was the only view that showed almost perfect agreement for both ALP and PLP. The RP5C might be superior to other views for assessing MV leaflet prolapse.

**Disclosures**

Disclosures to report, please report below

M. Borgarelli receives research and travel support by Ceva Sante Animale and American Kennel Club. He is also a consultant for Ceva Sante Animale A. Franchini receives salary support from Ceva Sante Animale and American Kennel Club

## 8 ESVC‐O‐8

### 8.1 ESVC ‐ European Society of Veterinary Cardiology

#### 8.1.1 Aortic annular plane systolic excursion in cats with hypertrophic cardiomyopathy: 151 cases

##### 8.1.1.1 L.C. Dutton ^1^, I. Spalla ^2^, J. Seo ^3^, J. Silva ^4^, J. Novo Matos ^5^

###### 8.1.1.1.1 ^1^ The Royal Veterinary College (RVC) Potters Bar United Kingdom; ^2^ Ospedale Veterinario San Francesco Milan Italy; ^3^ Animal Referral Centre Auckland New Zealand; ^4^ North Downs Specialist Referrals Bletchingley United Kingdom; ^5^ University of Cambridge Cambridge United Kingdom

Impairment of left ventricular (LV) longitudinal function is an early marker of systolic dysfunction in people with hypertrophic cardiomyopathy (HCM), and correlates with HCM severity and outcome. Longitudinal function can be assessed using mitral annular plane systolic excursion (MAPSE) and strain; however, these require left apical views that can be challenging to acquire in cats. In humans, another measure of LV longitudinal function is aortic annular plane systolic excursion (AAPSE), obtained from a right parasternal short‐axis view. In humans, AAPSE correlates with longitudinal strain and MAPSE, providing prognostic information.

We aimed to assess AAPSE in cats with HCM (stage B and C) and compare it with normal control cats. We hypothesised that AAPSE would be reduced in cats with HCM, and that cats in stage C would have a lower AAPSE than stage B cats.

Patient records were retrospectively reviewed. HCM was defined as LV wall thickness ≥6mm. M‐mode bisecting the aorta in a right parasternal short‐axis view was used to measure AAPSE. Data are presented as mean (95% confidence interval).

One‐hundred and fifty‐one cats were enrolled, 57 normal, 57 HCM stage B and 37 HCM stage C. AAPSE was lower in HCM cats compared to normal cats (3.3 (3.1 – 3.5) mm versus 4.6 (4.4 – 4.8) mm, *P*<0.001). Furthermore, AAPSE in stage C cats was lower than in stage B (2.4 (2.19 – 2.6) mm versus 3.9 (3.7 – 4.1) mm, *P*< 0.001). ROC analysis of AAPSE to differentiate stage C from stage B cats gave an AUC of 0.92 (0.87 – 0.98), with an optimal cut‐off value of <2.85 mm (sensitivity 78% and specificity 93%). AAPSE positively correlated with measures of LV longitudinal function (MAPSE (r= 0.6 (0.4 – 0.7), *P*< 0.001) and tissue doppler S’ (r=0.5 (0.2 – 0.7), *P*=0.002)) but showed no correlation with LV fractional shortening (r=0.1 (‐0.06 – 0.25), *P*=0.23). There was also no correlation between AAPSE and indices of LV forward flow (aortic VTI (r=‐0.06 (‐0.28 – 0.16), *P*=0.60) and aortic Vmax (r=‐0.10 (‐0.26 – 0.06), *P*=0.23)). AAPSE showed excellent intra‐operator repeatability (intraclass correlation coefficient = 0.98, coefficient of variation = 4.4%).

This study suggests that AAPSE might be a useful and easily acquired echocardiographic variable of LV longitudinal function in cats. AAPSE was reduced in HCM, and decreased with disease progression. Therefore, AAPSE might provide prognostic information, and provides further evidence that longitudinal systolic function is decreased in cats with HCM.

**Disclosures**

No disclosures to report

## 9 ESVC‐O‐9

### 9.1 ESVC ‐ European Society of Veterinary Cardiology

#### 9.1.1 An overview on the inflammatory and systemic fibrinolysis status in cats with heart disease stratified by the presence and type of congestive heart failure

##### 9.1.1.1 A. Zoia ^1^, M. Drigo ^2^, F. Busato ^1^

###### 9.1.1.1.1 ^1^ Clinica Veterinaria Privata San Marco Veggiano Italy; ^2^ Padua University Legnaro Italy

We recently demonstrated that the risk of aortic thromboembolism (ATE) in cats with cardiogenic pleural effusion is significantly lower than that of cats with cardiogenic pulmonary oedema and of cats with cardiac disease but no congestive heart failure (CHF). This reduced risk is not offset from the increased left atrium (LA). This study aimed to assess if cats with cardiogenic pleural effusion have less systemic inflammation or have an enhanced systemic fibrinolysis preventing ATE.

Cross‐sectional study retrospectively evaluating client‐owned cats with heart disease presented between 2004 and 2018. All cats included have undergone a full echocardiography and determination of serum amyloid A (SAA) and plasma fibrinogen at the time of first presentation. Cats were divided into 3 groups: without CHF (group 1), with cardiogenic pulmonary edema (group 2) and with cardiogenic pleural effusion (group 3). Frequency of ATE among groups was compared by chi‐square test. The serum SAA (marker of inflammation), plasma fibrinogen, fibrinogen:SAA (marker of systemic fibrinolysis), and LA/aorta ratio (LA:Ao) were compared among groups by Kruskal‐Wallis test with Bonferroni post‐hoc analysis.

In the study were included 389 cats (group 1 = 246, group 2 = 49, and group 3 = 94). Forty‐five cats had ATE and its frequency was statistically (P<0.001) different among the 3 groups (group 1, 23/246 [9.3%]; group 2, 17/49 [34.7%]; group 3, 5/94 [5.3%]). LA:Ao was significantly (P<0.001) increased in cats of group 2 (median=2.10, IQR=1.83‐2.50) and group 3 (median=2.0, IQR=1.69‐2.37) compared with group 1 (median=1.40, IQR=1.20‐1.63), while there was no significant difference between groups 2 and 3. SAA was significantly (P=0.027) increased in cats of group 3 (median=3.35, IQR=0.45‐59.90) compared with group 1 (median=0.65, IQR=0.10‐18.45) while there were no differences between group 1 and group 2 (median=1.40, IQR=0.10‐12.60) and group 2 and 3. Finally, fibrinogen:SAA was significantly (P=0.005) decreased in cats of group 3 (median=56, IQR=6‐407.75) compared with group 1 (median=316, IQR=13‐1447.5) while there were no significantly differences between group 1 and group 2 (median=143, IQR=18‐1450) and group 2 and 3.

As previously demonstrated, cats with CHF had a bigger LA compared with cats without CHF and frequency of ATE was lower in cats with cardiogenic pleural effusion. Inflammation unlikely play a role in the genesis of ATE being SAA the highest in group 3, while systemic fibrinolysis may play a role in the low ATE occurrence in cats of group 3 being fibrinogen:SAA the lowest in this group.

**Disclosures**

No disclosures to report

## 10 ESVC‐O‐10

### 10.1 ESVC ‐ European Society of Veterinary Cardiology

#### 10.1.1 The impact of chronic right ventricular pressure overload on right and left heart morphology and function

##### 10.1.1.1 E Van Renterghem, M Lekane, K Gommeren, AC Merveille

###### 10.1.1.1.1 University of Liège Liege Belgium

Right ventricular (RV) pressure overload (PO) is a major cause of heart failure in dogs, with pulmonic stenosis (PS) and pulmonary hypertension (PHT) as the main differentials. In humans PS and PHT display a different pattern of cardiac adaptations, with more severe dilation and worse function observed in PHT. Reports on direct comparison of cardiac adaptations in dogs with PS and PHT is lacking.

We hypothesize that with significant RVPO, dogs with PS display less dilated right heart chambers and better function than dogs with chronic pre‐capillary PHT.

Dogs with PS, PHT and control dogs, presented at a university teaching hospital, were retrospectively examined. Patients with PS or PHT, without signs of congestive heart failure were included if a right systolic pressure gradient (RSPG) of >55 mmHg was calculated based on maximal pulmonic velocity or tricuspid regurgitation, in the absence of other severe cardiac disease. Acute causes of PHT (thromboembolism) were excluded.

Quantitative data regarding right and left heart morphology and function were documented: RV size (RVIDd/Ao; nRVAd), wall thickness (RVFWd/LVFWd), systolic function (nRVAs; RVFAC; nTAPSE), right atrial (RA) size (RAD/LAD; RAA/BSA), left ventricular (LV) size (EDVI; ESVI) and shape (LV eccentricity index (EIs, EId)); left atrial (LA) size (LA/Ao). Comparison between groups was performed using either Kruskal‐Wallis or Mann‐Whitney. The effect of RPSG and group (PS and PHT) on parameters was assessed using ANCOVA. Data are expressed as median and range.

Sixty‐four dogs, PS (n=31), PHT (n=18), and control (n=15), were included. Besides TAPSE, LVEF and LA/Ao, each parameter was significantly different between the control and PS, PHT or both (p<0,03). RSPG was significantly higher in PS (108 mmHg [56‐219]) than PHT (91 mmHg [55‐158]). RV was significantly more dilated (RVID/Ao: 0.13 [0.09‐0.25] vs 0.10 [0.07‐0.19]; nRVAd: 1.17 [0.65‐1.66] vs 0.83 [0.45‐1.45]), less hypertrophied (RVFWd/LVFWd: 0.9 [0.44‐1.4] vs 1,0 [0.75‐2.0]; RVFWd/RVIDd: 0.30 [0.19‐0.55] vs 0.44 [0.24‐0.67]), displayed poorer systolic function (nRVAs: 0.69 [0.27‐1.23] vs 0.35 [0.16‐0.92], RVFAC: 0.37 [0.14‐0.66] vs 0.57 [0.29‐0.71]) (p<0.02), and interventricular septal flattening was worse (EIs: 1.5 [1.09‐5.71] vs 1.22 [0.88‐2.1]) in PHT (p<0.02). For these parameters ANCOVA showed that RVID/Ao, nRVAd, nRVAs and RVFAC were significantly different between groups and not influenced by RSVP while RVFWd/RVIDd and EIs were both influenced by RSPG and the group.

This study suggests that RV adaptation in chronic RVPO is different between dogs with moderate to severe PS and PHT.

**Disclosures**

No disclosures to report

## 11 ESVC‐O‐11

### 11.1 ESVC ‐ European Society of Veterinary Cardiology

#### 11.1.1 Effects of angiographic contrast media on invasive pressure measurement in dogs with pulmonic stenosis undergoing balloon valvuloplasty.

##### 11.1.1.1 J. Escalda ^1^, J Novo‐Matos ^2^, S Regada ^2^, B Pedro ^3^, C Partington ^2^, J Loureiro ^4^, D Casamian‐Sorrosal ^5^, C Linney ^3^, J Neves ^3^, J Silva ^2^

###### 11.1.1.1.1 ^1^ University of cambridge Cambridge United Kingdom; ^2^ University of Cambridge Cambridge United Kingdom; ^3^ Willows Veterinary Centre & Referral service Solihull United Kingdom; ^4^ North Downs Specialist Referrals Bletchingley United Kingdom; ^5^ Universidad Católica de Valencia Valencia United Kingdom

Angiographic studies and invasive pressure measurements (IPM) are routinely performed during pulmonic balloon valvuloplasty (BPV), the treatment of choice for severe pulmonic stenosis (PS). IPM and calculation of pulmonary valve gradients (PG) are important factors in the decision‐making process during BVP. IPM is commonly performed prior to angiography to avoid potential interference of angiographic contrast media administration on IPM. However, the impact of contrast agents on IPM during BVP is unclear.

A multicentre, prospective, observational study was performed to assess IPM changes after angiography with iohexol in dogs undergoing BPV. IPM were obtained in 3 locations (pre‐ and post‐contrast injection): pulmonary artery (PA), right ventricular outflow tract (RVOT) and right ventricle (RV). Changes in the IPM pressures (DIPM= IPM post‐contrast ‐ IPM pre‐contrast) and PG (peak RV IMP ‐ peak PA IPM) were calculated. Variables that could have an impact on IPM were recorded, namely heart rate (HR), systemic blood pressure (SBP) and administration of intravenous fluids or vasoactive drugs. Data are presented as median [range] and mean (± standard deviation), as appropriate.

Thirty‐five dogs were enrolled. French Bulldogs were overrepresented (8/35 dogs). PS was classified as type A in 24/35 dogs, type B in 3/35 dogs and mixed type in 8/35 dogs. Pre‐BVP echocardiography derived PG was 115.1 (± 36.1) mmHg. Median iohexol dose was 1.0 [0.5‐1.6] ml/kg per contrast study. Comparison of pre‐ and post‐ contrast IPM showed an increase in systolic RV pressure (80.3 (± 23.9) mmHg versus 87.1 (± 25.6) mmHg, *P*= 0.02), systolic RVOT pressure (70 [40‐136] mmHg versus 73 [33‐156] mmHg, *P*= 0.03), and mean RVOT pressure (20.5 [12‐44] versus 25 [13‐58] mmHg, P<0.01). The average DIPM were as follows: DIPM^SystolicRV^ 6.7 mmHg, DIPM ^SystolicRVOT^ 4.9 mmHg and DIPM ^MeanRVOT^ 5.1 mmHg. There were no changes in PA pressures, HR or SBP pre‐ versus post‐ contrast. Likewise, PG did not change (pre‐contrast 62.3 (± 25.1) mmHg versus post‐contrast 68.3 (± 26.1) mmHg, *P*= 0.052). Univariable linear regression analysis showed no association between DIPM^SystolicRV^, DIPM ^SystolicRVOT^ and DIPM ^MeanRVOT^ with contrast volume, DHR, DSBP and time between IPM and contrast administration.

This study showed a statistically significant increase in RV IPM and RVOT IPM post‐contrast administration. However, this was a small increase and unlikely to have a clinical impact in the assessment of PS severity and the decision‐making process during BVP.

**Disclosures**

No disclosures to report

## 12 ESVC‐O‐12

### 12.1 ESVC ‐ European Society of Veterinary Cardiology

#### 12.1.1 Breakthrough: A First‐In‐Class Virtual Simulator for Dose Optimization Of Angiotensin Converting Enzyme Inhibitors in Cardiology

##### 12.1.1.1 J.P. Mochel, B. Schneider, S. Sotillo, A. Bourgois‐Mochel, A Mosichuk, J Smith, C Iennarella‐Servantez, C Johnson, C Garelli‐Paar, E Guillot, M Prikazsky, J Ward

###### 12.1.1.1.1 Iowa State University College of Veterinary Medicine Ames United States

Little is known about the optimal dosing of the angiotensin converting enzyme inhibitor (ACEi) benazepril in dogs with congestive heart failure (CHF). Seminal studies had initially suggested that the pharmacological action of benazepril was relatively independent of doses > 0.2 mg/kg, thereby providing a rationale for the current European labeled dose of 0.25 mg/kg P.O q24h in dogs with CHF. However, most of these earlier studies rely on measures of ACE activity, which is a suboptimal endpoint to characterize the effect of ACEi on the renin‐angiotensin aldosterone system (RAAS). In practice, the ACVIM recommends that most canine patients with CHF receive a daily dose of 1.0 mg/kg P.O of benazepril.

The objectives of this study were (1) to characterize the dose‐exposure‐response relationship of benazepril(at) on biomarkers of the RAAS which are relevant to CHF pathophysiology and associated with morbidity/mortality {aldosterone and angiotensins (I, II, III, IV, Ang1‐7, Ang1‐5)} using a quantitative systems pharmacology (QSP) modeling approach; and (2) to develop a software implementation of the benazeprilat‐RAAS QSP model capable of rapidly simulating pharmacodynamic response for optimal dosing of benazepril at the bedside.

Using a low‐sodium diet model of RAAS activation, nine healthy Beagle dogs were divided into three dosing groups: 0.125 mg/kg q12h, 0.25 mg/kg q12h, and 0.5 mg/kg q24h benazepril PO. Blood samples were collected throughout 24 hours for benazeprilat, angiotensins, and aldosterone determination. Experimental data were imported into the Monolix Suite 2020R1 for data exploration, mathematical model development and evaluation. Quality of fit was evaluated using standard goodness‐of‐fit diagnostics, while the user interface for the simulation engine was developed from the Shiny application.

The effect of ACE inhibition on the RAAS was modeled using a series of direct response models with feedback mechanisms to account for the complex biological interactions within the RAAS. Using this simulation engine, we could predict and quantify the effect of varying dosing schedules of benazepril on the classical and alternative arm of the RAAS on top of diurnal variations in untreated animals. Specifically, the simulator estimated a difference of only 10‐15% in AngII and Ang1‐7 vs. placebo between a 0.25 mg/kg q12h and 0.5 mg/kg q12h oral dose of benazepril. By developing an easy‐to‐use simulation interface for our model, we are now able to make a first prediction of the optimal dose/time of benazepril administration in dogs in support of future investigations in patients with CHF.

**Disclosures**

Disclosures to report, please report below

Jonathan P. Mochel is a consultant for Ceva Sante Animale. He is also a co‐founder of 3D Health Solutions, a startup company developing novel in vitro systems for drug discovery. Charles Johnson, Catherine GARELLI‐PAAR, Emilie Guillot and Marc Prikazsky are employees of Ceva Santa Animale.

## 13 ESVC‐O‐13

### 13.1 ESVC ‐ European Society of Veterinary Cardiology

#### 13.1.1 Systemic hypocoagulability in cats with arterial thromboembolism detected by global hemostatic tests

##### 13.1.1.1 R. Langhorn, M. Bach, A.S. Gravgaard, N. Graversen, C. Olsen, K.H. Monrad, J.L. Willesen, A.T. Kristensen, J. Koch, L.N. Nielsen

###### 13.1.1.1.1 University of Copenhagen Frederiksberg C Denmark

Arterial thromboembolism (ATE) is a known complication of feline cardiomyopathies (CM). Hypercoagulability is believed to be one predisposing factor. This prospective case‐control study aimed to assess global hemostatic tests for detection of hypercoagulability in cats with ATE.

Cats with CM with and without ATE were included along with healthy cats. For each cat, echocardiography, whole blood thromboelastography (TEG) and thrombin generation testing (TGT) were performed. Hypercoagulability was defined for TEG as shortened reaction time (R), shortened kinetics (K), increased angle, or increased maximal amplitude and for TGT as decreased lag‐time, decreased time‐to‐peak, increased peak, or increased endogenous thrombin potential (ETP). If ≥1 parameter showed significant (P<0.05) hypercoagulability compared to the control group, and the remaining parameters either tended in the same direction or were not significantly different from the control group in the opposite direction, the patient was considered hypercoagulable. Hypocoagulability was defined by the opposite criteria. Comparison between groups was performed with unpaired analysis of variance (for parametric data) or Kruskal‐Wallis test (for non‐parametric data) followed by individual post‐hoc analyses.

Six cats with ATE, 11 with CM only, and 13 healthy cats were included. Significant hypocoagulability was detected for cats with ATE compared to both cats with CM and healthy cats, characterized by prolonged (median (range)) R (ATE: 12.0 (6.6‐16.3) min, CM: 4.8 (3.5‐10.8) min, healthy: 4.0 (2.8‐7.5) min) and K (ATE: 5.9 (2.1‐9.9) min, CM: 2.6 (1.2‐6.4) min, healthy: 1.7 (1.1‐3.8) min) on TEG and increased time‐to‐peak (ATE: 6.0 (3.9‐6.5) min, CM: 3.8 (3.0‐5.5) min, healthy: 3.3 (2.7‐5.3) min) and decreased peak (ATE: 61 (26‐198) nm, CM: 179 (52‐286) nm, healthy: 238 (149‐293) nm) and ETP (ATE: 251 (115‐610) nm/min, CM: 488 (224‐704) nm/min, healthy: 569 (446‐735) nm/min) on TGT. Additionally, cats with ATE also had significantly decreased angle on TEG compared to healthy cats, but not to cats with CM (ATE: 31.2 (4.3‐63.8) degrees, CM: 56.2 (30.9‐71.6) degrees, healthy: 67.4 (45.7‐73.1) degrees).

A hypocoagulable state was detected in cats with ATE as opposed to the expected hypercoagulability. While the cause is unclear, platelet exhaustion and local hypercoagulability in the endocardium with subsequent systemic exhaustion of coagulation factors are among speculated causes.

**Disclosures**

Disclosures to report, please report below

This study was funded by Agria and SKK's Research Fund and by the Fund of Scientific Studies in Companion Animals, University of Copenhagen. Individual disclosures for authors: Langhorn: Other research funded by Agria and SKK's Research Fund and by the Fund of Scientific Studies in Companion Animals. Prior Speaker engagement with various larger clinical practices in Denmark. Bach: Other research funded by Agria and SKK's Research Fund and by the Fund of Scientific Studies in Companion Animals. Willesen: Other research funded by a university research grant (University of Copenhagen), by the Danish Kennel Club, by the Danish Bull Terrier Club, and by Elanco Animal Health. Research collaboration with Zoetis Animal Health. Prior speaker engagement with Bayer Animal Health, Ceva Animal Health, Zoetis Animal Health, Elanco Animal Health, and Boehringer Ingelheim. Kristensen: Other research funded by the Independent Research Fund Denmark. Koch: Other research funded by a university research grant (University of Copenhagen), by Agria and SKK's Research Fund, by the Fund of Scientific Studies in Companion Animals, by the Danish Kennel Club, and by the Danish Bull Terrier Club. Nielsen: Academic laboratory manager of The Veterinary Diagnostic Laboratory, University of Copenhagen. Other research (PhD and Post Doc projects) funded by the Independent Research Fund Denmark. Research collaboration with Eiken Chemicals Ltd and Zoetis. Prior Speaker engagement with both IDEXX International and various larger clinical practices in Denmark.

## 14 ESVC‐O‐14

### 14.1 ESVC ‐ European Society of Veterinary Cardiology

#### 14.1.1 Comparison of the diagnostic value of a small, single channel, electrocardiographic monitoring patch with a standard 3‐lead Holter system over 24 hours in dogs

##### 14.1.1.1 N. Schreiber ^1^, R. Willis ^2^, T.M. Glaus ^1^, M. Baron Toaldo ^1^

###### 14.1.1.1.1 ^1^ Vetsuisse Faculty, University of Zurich Zurich Switzerland; ^2^ Sarah Smith Cardiology Derby United Kingdom

Ambulatory ECG recordings are pivotal to diagnose and manage cardiac arrhythmias. Some Holter equipment might be too large and uncomfortable to wear for dogs. Moreover, the need of applying several electrodes can have an impact on the quality and therefore editing time of the recording. Some easy to apply small patch event recorders are available on the market for people, and a few have been tested in animals. The carnation ambulatory monitor (CAM) from BardyDX, is a novel, small, light, and easy to wear patch monitor/event recorder that can function as a traditional Holter ECG. The aim of this study was to compare the CAM device with a traditional Holter system in a population of dogs with underlying rhythm disturbance.

Nineteen adult dogs with different types of arrhythmias and different underlying cardiac and extracardiac diseases were prospectively included in this comparative explorative study. The 2‐channel CAM and a standard 3‐channel Holter system (Spacelabs Healthcare) were simultaneously applied to each dog for 24 consecutive hours. One single operator blinded to the dogs’ identity read and edited all the traces at the end of the study period. Selective ECG variables were acquired for each system and compared.

Recording and trace analysis were feasible for all dogs. Operator's reading time (P=0.013) and percentage of artifacts (P<0.001) were greater for CAM (121 minutes [40‐264]; and 11% [0‐34], respectively) compared to standard Holter (64 minutes [18‐270]; and 1% [0‐9], respectively). While total number of beats (P=0.017), and maximum (P=0.02) and mean (P=0.037) heart rates were lower for CAM (113,806 beats [61,449‐143,981]; 227 bpm [166‐300]; and 88 bpm [42‐140], respectively) compared to standard Holter (131,640 beats [67,563‐243,081]; 260 bpm [173‐430]; and 92 bpm [42‐164], respectively). Minimal heart rate (P=0.725), number of pauses (P= 0.078), duration of the longest pause (P=0.087), number of ventricular ectopic beats (P=0.55), couplets (P=0.186), ventricular tachycardia (P=0.05), ventricular arrhythmia grade (Lown grade)(P=0.203), and presence or absence of ventricular bigeminy, trigeminy, supraventricular tachycardia and atrial fibrillation (P=0.98) did not differ between the two recorders.

To conclude, the CAM is feasible in dogs to analyze the underlying rhythm over 24 hours. Its dimension and comfort to wear are appealing aspects, and minimal differences were found in the analyzed ECG parameters when compared to a traditional Holter system. On the other side, the time spent in editing and reading the traces and the higher percentage of artifacts, might represent limiting factors in the clinical setting.

**Disclosures**

Disclosures to report, please report below

Grants/research: This work was supported by the ESVC clinical study fund. The authors have no financial interest in the Bardy company and have not received any payment. Tony Glaus received speaker fees from Boehringer‐Ingelheim.

## 15 ESVC‐O‐15

### 15.1 ESVC ‐ European Society of Veterinary Cardiology

#### 15.1.1 Genetic susceptibility of canine myxomatous mitral valve disease

##### 15.1.1.1 M. Hezzell ^1^, M. Wallace ^2^, J. Wilshaw ^3^, A. Boswood ^3^, L. Davison ^3^

###### 15.1.1.1.1 ^1^ University of Bristol Bristol United Kingdom; ^2^ University of Oxford Oxford United Kingdom; ^3^ Royal Veterinary College London United Kingdom

Myxomatous mitral valve disease (MMVD) is the most common cardiovascular disease affecting adult dogs, often leading to congestive heart failure (CHF). A genetic component is suggested by strong breed predispositions e.g. Cavalier King Charles Spaniel (CKCS). Novel therapies directed at underlying patho‐physiological mechanisms are urgently needed. We hypothesize that a combination of genetic variants contribute to risk of MMVD and CHF in CKCS, and may offer new treatment targets. Some variants may be at high frequency in the breed, and others present at higher frequency in CKCS with earliest‐onset CHF compared to CKCS with late onset CHF. The aims of this study were to 1) identify genetic variants associated with early onset of CHF in CKCS with MMVD and 2) to explore these variants further in a validation cohort.

Archived surplus blood or buffy coat samples from a total of 142 client‐owned dogs with MMVD were used for this case‐control genotyping study including 121 CKCS.

Whole‐genome sequencing (WGS) of a discovery cohort of 12 CKCS with MMVD was undertaken at 30X coverage on the Illumina platform. Additional unpublished WGS data was available from 48 dogs across 7 breeds for comparison, as well as data in public databases (e.g. Dog Biomedical Variant Database Consortium). A bespoke bioinformatics workflow based on Genome Analysis Tool Kit (GATK) best practices identified c.2000 variants of potential cardiovascular relevance. These were either high frequency within CKCS compared to other breeds, or at highest frequency in CKCS with earliest‐onset CHF. Variants were prioritized further and validated by two rounds of targeted high throughput sequencing based genotyping of additional cohorts. In addition to CKCS, additional dogs with MMVD from 7 further breeds were included in follow up genotyping (1‐4 dogs per breed). Additional targeted sequencing genetic data were available from a total of 165 dogs without MMVD across 9 breeds.

We identified >100 variants of potential importance in CKCS MMVD and associated CHF. Notably, many significant variants were located in genes and biological pathways with potential for therapeutic intervention, including transcriptional regulation, calcium signaling, cell adhesion, inflammation, and wound healing. Evaluation of the impact on gene and cell function of the genetic variants associated with MMVD and CHF in MMVD is now required.

These findings offer novel pathway targets for pharmacological intervention and future precision treatment of MMVD and CHF.

**Disclosures**

Disclosures to report, please report below

The authors have received research funding from Boehringer Ingelheim, Ceva Animal Health. Dechra Veterinar Products, the Evetts‐Luff Feline Endowment, the American Kennel Club Canine Health Foundation, the Hong Kong Jockey Club, the Academy of Medical Sciences, the Winm Feline Foundation, UKRI, the Petplan Charitable Trust, BSAVA PetSavers, ECVIM Clinical Studies Fund, the Medical Research Council, the Juvenial Diabetes Research Foundation, the University of Dublin, Zoetis, the Wellcome Trust, the Langford Trust and Royal Canin. Melanie Hezzell has received speaker honoraria and travel expenses from the Veterinary Cardiovascular Society, the American College of Veterinary Internal Medicine, Boehringer Ingelheim, Ceva Animal Health and IDEXX Laboratories. Jenny Wilshaw has a consultancy agreement with Boehringer Ingelheim for research and speaking. Adrian Boswood holds consultancy agreements with Boehringer Ingelheim Ltd and CEVA Animal Health. Lucy Davison has received honoraria and travel expenses from the ACVIM, the European Society of Veterinary Endocrinology and Improve International.

## 16 ESVC‐O‐16

### 16.1 ESVC ‐ European Society of Veterinary Cardiology

#### 16.1.1 Left atrial fractional shortening in cats: a comparison between two echocardiographic views.

##### 16.1.1.1 A. Victorino Machado, L. Gardner, C. Partington, J. . Silva, J. Novo Matos

###### 16.1.1.1.1 University of Cambridge Cambridge United Kingdom

Left atrial fractional shortening (LA‐FS%) is a widely used index of left atrial (LA) systolic function and is part of the standard‐of‐care echocardiographic protocol for feline cardiomyopathies. In cats with hypertrophic cardiomyopathy (HCM), decreased LA‐FS% is associated with increased risk of cardiac death, congestive heart failure and aortic thromboembolism. LA‐FS% can be determined from a M‐mode right parasternal short‐axis view (LA‐FS%^RPSA‐MM^) or two‐dimensional right parasternal long‐axis 4‐chamber view (LA‐FS%^RPLA‐2D^). However, these methods may not be interchangeable and LA‐FS% determined by LA‐FS%^RPSA‐MM^ can potentially be affected by left ventricular (LV) systolic function and aortic root motion. We aimed to assess the agreement between LA‐FS%^RPSA‐MM^ and LA‐FS%^RPLA‐2D^ in normal and HCM stage B cats; and to assess the association between LA‐FS%^RPSA‐MM^ and LA‐FS%^RPLA‐2D^ with LV function and aortic annular plane systolic excursion (AAPSE), a measurement of aortic root motion. Clinical and echocardiographic databases were retrospectively reviewed. HCM was defined as LV wall thickness ≥5.5 mm. M‐mode bisecting the aorta and LA body in a short‐axis view was used to measure LA‐FS%^RPSA‐MM^ and AAPSE. LA‐FS%^RPLA‐2D^ was determined in a long‐axis 4‐chamber view. All echocardiograms were reviewed and measured by one operator. Data are presented as mean (95% confidence interval). Eighty‐eight cats were enrolled, 43 normal, and 45 with HCM. LA‐FS% was lower in HCM cats compared to normal cats using both echocardiographic methods (LA‐FS%^RPSA‐MM^ *P*< 0.0001; LA‐FS%^RPLA‐2D^ *P*= 0.008). There was no difference between LA‐FS%^RPSA‐MM^ and LA‐FS%^RPLA‐2D^ in the whole population (27.2% (26.1‐28.4) versus 27.7% (26.3‐29.0), *P*= 0.48), normal or HCM cats. On Bland‐Altman analysis there was an insignificant bias between LA‐FS%^RPSA‐MM^ and LA‐FS%^RPLA‐2D^, but the limits of agreement were wide (bias ‐0.44, 95% limits of agreement ‐ 11.8‐11.0). There was a weak correlation between LA‐FS%^RPSA‐MM^ and LA‐FS%^RPLA‐2D^ with AAPSE (r= 0.5 (0.3‐0.6); r= 0.36 (0.16‐0.53), respectively), aortic Vmax (r= ‐0.4 (‐0.56‐ ‐0.24); r= ‐0.25(‐0.4‐ ‐0.04)) and aortic VTI (r= ‐0.36 (‐0.53‐ ‐0.16); r= ‐0.22 (‐0.41‐ ‐0.007)). There was no correlation between LA‐FS%^RPSA‐MM^ or LA‐FS%^RPLA‐2D^ and LV fractional shortening. Intra‐operator measurement variability was excellent for LA‐FS%^RPSA‐MM^ and AAPSE (intraclass correlation coefficient (ICC) 0.90 and 0.97; coefficient of variation (%CV) 5.3% and 2.9%, respectively); and moderate‐good for LA‐FS%^RPLA‐2D^ (ICC 0.59; %CV 10.6%). Our study suggests that LA‐FS%^RPSA‐MM^ and LA‐FS%^RPLA‐2D^ cannot be used interchangeably when assessing LA‐FS%, as the limits of agreement between these 2 methods were unacceptably large. In our population, neither LV systolic performance nor AAPSE seem to markedly affect LA‐FS% measurements.

**Disclosures**

No disclosures to report

## 17 ESVC‐O‐17

### 17.1 ESVC ‐ European Society of Veterinary Cardiology

#### 17.1.1 Prevalence and clinical significance of heart murmurs in 809 cats

##### 17.1.1.1 L. Ferasin, H. Ferasin, A. Cala, N. Creelman

###### 17.1.1.1.1 Specialist Veterinary Cardiology Consultancy Alton United Kingdom

Heart murmurs are commonly detected in cats and echocardiographic evaluation is ultimately required to identify the cause of blood flow turbulence responsible for this clinical finding. Some studies have previously reported the prevalence of heart murmurs in this species; however, a methodical evaluation of the precise origin of murmurs in cats has not been reported, especially after the recent description of inducible heart murmurs in cats. Therefore, the aim of this study was to systematically evaluate the precise origin and clinical significance of murmurs in cats referred for echocardiographic evaluation.

Clinical records of 1461 cats that underwent echocardiographic examination for evaluation of a heart murmur between June 2009 and February 2022 were retrospectively reviewed. Only cases with complete clinical information were included in this study (n=809). Sedation before examination was deemed necessary in 8.8% of cats. Their median age was 5.7 (CI 5.3‐6.2) years and median body weight was 4.38 (CI 4.3‐4.52) Kg. Sixty three percent of cats were male, while 37% were female.

The timing of heart murmurs was systolic in 801 cats (99.0%), continuous in 4 cats (0.5%), diastolic in 3 cats (0.4%) and to‐and‐fro in 1 cat (0.1%).

A dynamic heart murmur was detected in 27.1% of cats, while 72.9% had a murmur that did not change in intensity during auscultation.

The origin of the murmur was identified in 92% of cats suggesting that, in the remaining 8%, the murmur was not associated with any functional abnormality (flow murmurs). Two different turbulent flows were associated with an audible murmur in 25.9% of cats, while 1.2% of cats had three different causes of murmur.

The majority of cats did not have any structural cardiac lesion (55.9%), whereas the remaining cats had acquired (30.8%) or congenital (13.3%) cardiac abnormalities.

The most common source of heart murmur was dynamic left ventricular outflow obstruction (LVOTO) secondary to systolic anterior motion of the mitral valve (SAM, 406 cases), followed by dynamic right ventricular outflow obstruction (DRVOTO, 326 cases), tricuspid regurgitation (n=68), mitral regurgitation (n=67), mid‐LVOTO (n=19), and various flow abnormalities associated with congenital defects in the remaining cases. DRVOTO was iatrogenic in 16% of cases and 4.4% of cases with SAM were only detected after provocative testing.

To the best of our knowledge, this is the largest study on prevalence and clinical significance of heart murmurs in cats and provides novel information about the potential causes of heart murmurs in this species.

**Disclosures**

Disclosures to report, please report below

All authors receive a salary from their employer

## 18 ESVC‐O‐18

### 18.1 ESVC ‐ European Society of Veterinary Cardiology

#### 18.1.1 The Vertebral Right Heart index: a new radiographic method to assess right heart enlargement in dogs

##### 18.1.1.1 C. Puccinelli ^1^, T. Vezzosi ^1^, G. Grosso ^1^, R. Tognetti ^1^, E. Auriemma ^2^, O. Domenech ^2^, S. Citi ^1^

###### 18.1.1.1.1 ^1^ Department of Veterinary Sciences, University of Pisa San Piero a Grado Italy; ^2^ Anicura Istituto Veterinario Novara Novara Italy

In veterinary medicine the radiographic assessment of right heart enlargement (RHE) is essentially subjective, and it concerns predominantly the evaluation of margins and shape of the cardiac silhouette. The aim of this study was to evaluate the vertebral right heart index (VRHi), a new quantitative radiographic method to detect RHE in dogs.

This was a multicenter, retrospective, observational study, including dogs with RHE and healthy dogs. All dogs had to have been performed a thoracic radiographic study and complete echocardiography on the same day. Right cardiac enlargement was defined as the presence of right atrial enlargement, right ventricular enlargement and/or hypertrophy based on echocardiography. For the radiographic study, right lateral (RL), left lateral (LL), ventro‐dorsal (VD) and dorso‐ventral (DV) views were considered. In lateral views, the long axis and the maximal short axis of the heart were identified as previously described for the vertebral heart scale. On the short axis, the distance from the cranial margin of the cardiac silhouette to the long axis line was measured and expressed in thoracic vertebral body units. This measurement was named lateral VRHi. In sagittal views, the thoracic longitudinal axis was drawn as a line superimposed on the vertebral column. The maximal transversal axis of the heart was drawn perpendicular to the thoracic longitudinal axis. On the transversal axis, the distance from the right margin of the cardiac silhouette to the thoracic longitudinal axis was calculated and measured against thoracic vertebrae in lateral views, as described above. This measurement was named sagittal VRHi.

A total of 204 dogs were included: 113 healthy dogs and 91 dogs with RHE (pre‐capillary pulmonary hypertension, n=52; pulmonary stenosis, n=33; tricuspid dysplasia, n=6). The VRHi (RL), the VRHi (LL) and the VRHi (VD) were significantly higher in dogs with RHE compared to controls (p<0.0001). The best diagnostic accuracy was observed for VRHi (LL) [area under the curve (AUC) 0.86, p<0.0001; cut‐off ≥3.5, sensibility (Se) 71%, specificity (Sp) 89%], followed by VRHi (RL) (AUC 0.85, p<0.0001; cut‐off ≥3.3, Se 83%, Sp 72%) and VRHi (VD) (AUC 0.80, p=0.0004; cut‐off ≥3, Se 57%, Sp 95%). Maximum Sp (100%) was obtained with the following cut‐offs: VRHi (LL) ≥4.2, VRHi (RL) ≥4 and VRHi (VD) ≥3.2.

In conclusion, the lateral VRHi in LL and RL, and the sagittal VRHi in VD, could represent useful radiological tools for the detection of RHE in dogs.

**Disclosures**

No disclosures to report

## 19 ESVC‐O‐19

### 19.1 ESVC ‐ European Society of Veterinary Cardiology

#### 19.1.1 Echocardiographic reference intervals for right pulmonary artery dimension and distensibility index: a prospective study in 269 healthy dogs

##### 19.1.1.1 G. Grosso ^1^, R. Tognetti ^1^, O. Domenech ^2^, F. Marchesotti ^2^, V. Patata ^2^, T. Vezzosi ^1^

###### 19.1.1.1.1 ^1^ University of Pisa San Piero a Grado, Pisa Italy; ^2^ Anicura Istituto Veterinario Novara Granozzo con Monticello, Novara Italy

Pulmonary artery (PA) is one of the main anatomic sites evaluated for the echocardiographic assessment of pulmonary hypertension (PH) in dogs according to the American College of Veterinary Internal Medicine guidelines. The dimension of the main PA and the right pulmonary artery distensibility index (RPADi) are two of the echocardiographic parameters used to assess the probability of PH in dogs. However, no data are available on the echocardiographic reference intervals (RIs) for the dimension of the PA branches and the RPADi in a large sample of healthy dogs. There is evidence that PA branches dilatate earlier that main PA in case of PH in humans. Therefore, the aim of the study was to describe the echocardiographic RIs of right pulmonary artery (RPA) dimensions and RPADi in dogs.

This multicenter observational study prospectively included 269 healthy dogs. The RPA maximum systolic diameter (RPA_max) and the RPA minimum diastolic diameter (RPA_min) were measured using M‐mode echocardiography from the right parasternal short axis view of the heart base. The M‐mode cursor was directed perpendicular to the longitudinal axis of the RPA, tangent to the aortic wall of the non‐coronary cusp. Prediction intervals for RPA_max and RPA_min were generated using allometric scales. In addition, the RIs of RPA_max and RPA_min indexed to the aortic annulus (RPA_max/Ao and RPA_min/Ao) and RPADi were defined. Finally, to verify the suitability of the RPA dimensions in a clinical setting, intra and interobserver measurement variability were determined using the coefficient of variation (CV).

Strong positive linear relationship between RPA_max, RPA_min and body weight (BW) was evident after logarithmic transformation (R^2^=0.787 and R^2^=0.725, respectively; P<0.0001). According to allometric scales, the 95% prediction interval of the RPA_max normalized to BW [RPA_max_N=RPA_max(mm)/BW(kg)^0.335^] was between 3.23 and 5.62, whilst the 95% prediction interval of the RPA_min normalized to BW [RPA_min_N=RPA_min(mm)/BW(kg)^0.364^] was between 1.62 and 3.30. The median RPA_max/Ao was 0.70 (RI, 0.53‐0.98), the median RPA_min/Ao was 0.40 (RI, 0.29‐0.61) and the median RPADi was 40.4% (RI, 31.2‐54.2%). Intraobserver and interobserver measurement variability for RPA_max yielded average CVs of 1.5% and 5.3%, and for RPA_min yielded average CVs of 1.6% and 6.4%.

We described the RIs of RPA dimensions and RPADi in a large sample of healthy dogs. The objective quantification of the RPA size could help in the identification of PA branches enlargement in dogs, especially useful in the echocardiographic screening for PH, and possibly in its severity assessment.

**Disclosures**

No disclosures to report

## 20 ESVC‐O‐20

### 20.1 ESVC ‐ European Society of Veterinary Cardiology

#### 20.1.1 Feasibility of isomeric myography to measure vascular function in pet dogs

##### 20.1.1.1 M. Mazzarella ^1^, N.K. Jones ^2^, G. Culshaw ^1^

###### 20.1.1.1.1 ^1^ The University of Edinburgh Roslin United Kingdom; ^2^ The University of Edinburgh Edinburgh United Kingdom

Isomeric myography is the gold standard *ex vivo* technique to study vascular function. Vascular dysfunction contributes to human cardiovascular disease, but no studies have been conducted in pet dogs using isomeric myography. This study aimed to assess the feasibility of isomeric myography in pet dogs, and to determine its use in quantifying vascular function in dogs with cardiac disease.

Femoral, renal, and mesenteric arteries were collected from three dogs euthanised for welfare reasons and confirmed as arteries by histology. The mitral valve was examined and graded 1‐4 (Whitney classification) for myxomatous mitral valve disease (MMVD). Arteries were stored in physiological saline solution (PSS) at 4°C. Ring sections of 2mm length were obtained under stereo microscope and then they were mounted in wells with PSS at 37°C bubbled with gas mixture of 95% oxygen and 5% carbon dioxide. The arteries were stretched to an internal circumference (IC) that generated the maximal difference between active and passive wall tension (IC_1_). The ratio between the IC_1_ and IC at which the passive wall tension achieved the target pressure of 100mmHg (IC_100_) determined the normalisation factor. Vasoconstriction to phenylephrine, and vasodilation to acetylcholine (endothelial‐dependent) and sodium nitroprusside (endothelial‐independent) were assessed by cumulative response curves at normalised IC. Concentrations of agents that gave 50% of the maximal contraction (EC_50_) and inhibition (IC_50_) were calculated.

Mean values of normalisation factors were 0.94±0.09 (renal), 1.06±0.1 (femoral) and 1.1±0.12 (mesenteric). Responses to phenylephrine (EC_50_=5.05±0.49 ‐log(M)) were similar between dogs (p=0.14). Reduced responses to acetylcholine (IC_50_=7.58±0.69 ‐log(M)) compared with sodium nitroprusside (IC_50_=7.95±2.99 ‐log(M)) were present in one dog with MMVD grade 3 (p=0.03) but not in two dogs with MMVD grades 1‐2.

Isomeric myography of arteries from pet dogs is feasible. It identifies loss of endothelial‐dependent relaxation and may be used to quantify vascular dysfunction in dogs with MMVD.

**Disclosures**

Disclosures to report, please report below

M.O. Mazzarella: studentship for masters by research funded by BSAVA‐PetSavers and The Kennel Club. The study presented here is part of the MScR N.K. Jones: funding kidney research UK(project not involved in the study presented here) G.J. Culshaw: funding from Kidney research UK, BSAVA‐PetSavers, The Kennel Club

## 21 ESVC‐O‐21

### 21.1 ESVC ‐ European Society of Veterinary Cardiology

#### 21.1.1 Atrial depolarization waves localization and characterization on surface electrocardiogram in dogs with fast supraventricular tachycardias

##### 21.1.1.1 S. Battaia ^1^, M. Perego ^1^, D. Cavallini ^2^, R. Santilli ^1^

###### 21.1.1.1.1 ^1^ Clinica Veterinaria Malpensa ‐ Anicura Samarate (Varese) Italy; ^2^ University of Bologna Ozzano dell'Emilia (Bologna) Italy

Narrow complex tachycardias are a spectrum of tachycardias, typically of supraventricular origin that, despite different pathophysiological mechanism, are often characterized by similar electrocardiographic presentation. The aims of the present study were to localize and characterize atrial deflections (AD) on 12‐lead electrocardiogram in a population of dogs affected by sustained fast supraventricular tachycardia (SVT), and to assess their utility in differentiating the tachycardia's type.

Data of dogs affected by sustained SVT with a ventricular rate >180 bpm, in which the electrocardiographic diagnosis was confirmed by EP study, were retrospectively reviewed and grouped according to tachycardia's type: orthodromic atrioventricular reciprocating tachycardia (OAVRT), focal atrial tachycardia (FAT) and atrial flutter (AFL). AD position and characteristics on 12‐lead surface electrocardiogram were assessed according to the sequence of intracardiac activation, and the relationship between AD and QRS complex interval (AD‐R) and QRS complex and AD interval (R‐AD) were evaluated. A control group of dogs with sinus tachycardia (ST) was also included. For numerical variables, mixed‐model was used; groups were included as fixed‐effect, and dogs as random‐effect. Non‐normal parameters were Box‐Cox transformed. Categorical variables were analysed using a nominal‐logistic‐model with groups as effects.

92 dogs with OAVRT, 33 with FAT, 17 with AFL and 40 with ST were analysed. Mean AD‐AD interval was 213 ms ± 30 in OAVRT (regular in 96% and alternant in 4% of cases); 270 ms ± 38 in FAT (regular in 55%, cycle length irregularity in 45%); 199 ms ± 57 in AFL (regular in 100%), 292 ms ± 31 in ST (regular in 100%). Considering AD position, OAVRT were characterized by: short R‐AD interval (100%); FAT: long R‐AD (52%), AD on T wave (33%), short R‐AD (12%), variable position of AD (3%); AFL: variable position of AD (59%), long R‐AD (18%), short R‐AD (18%), AD on T wave (6%); ST: long R‐AD (100%). The R‐AD/AD‐R ratio was different between the four groups (median in OAVRT: 0.54, IQR 0.45‐0.64; FAT: 1.45, 0.92‐1.67; AFL: 0.81, 0.63‐1.13; ST: 2.68, 2.25‐3.05) (P<0.05). Differences between groups were found for AD axis on frontal plane, AD duration, atrial rate and ventricular rate (P<0.05). The atrioventricular conduction ratio was 1:1 in 100% of OAVRT and ST; in FAT: 97% 1:1 and 3% 2:1; in AFL: 41% 1:1, 41% 2:1 and 18% variable.

These results suggest that analysis of AD position and appearance on 12‐lead ECG could be helpful in the differentiation of most common SVT of dogs.

**Disclosures**

No disclosures to report

## 22 ESVC‐O‐22

### 22.1 ESVC ‐ European Society of Veterinary Cardiology

#### 22.1.1 Association between echocardiographic indexes and urinary Neutrophil Gelatinase‐Associated Lipocalin (uNGAL) in dogs with myxomatous mitral valve disease

##### 22.1.1.1 S Oricco ^1^, F Fidanzio ^1^, S Crosara ^1^, F Dondi ^2^, C Mazzoldi ^2^, G Romito ^2^, MC Sabetti ^1^, R Troia ^2^, C Quintavalla ^1^

###### 22.1.1.1.1 ^1^ University of Parma Parma Italy; ^2^ University of Bologna Ozzano dell'Emilia Italy

Type 2 chronic cardiorenal syndrome has been described in dogs with myxomatous mitral valve disease (MMVD). Urine neutrophil gelatinase‐associated lipocalin (uNGAL) and uNGAL normalized with urinary creatinine (uNGALC) increase after renal tubular injury and are biomarkers of renal damage both in humans and dogs.

The aim of this study was to evaluate the association between echocardiographic variables, and uNGAL or uNGALC, in patients with MMVD.

Seventy‐seven dogs with MMVD, at different ACVIM stage, were included in the multicentric prospective observational case‐control study. All dogs underwent echocardiographic exam, serum chemistry and urinalysis.

Echocardiographic data analyzed were shortening fraction (SF), normalized left ventricular diastolic (LVIDDn) and systolic (LVIDSn) diameters, left atrium to aortic root ratio (LA/Ao), maximal (LAV_max_) and minimal (LAVmin) left atrial volumes, LA total emptying volume (LA_TEV_), expansion index, emptying index, mitral valve E wave velocity (E_Vmax_), E/E', aortic (VTI_Ao_) and mitralic (VTI_mit_ ) velocity time indexes and their ratio (VTI_mit_/VTI_Ao_) and tricuspid regurgitation velocity (TR_Vmax_).

The associations of echocardiographic index with uNGAL and uNGALC were investigated using univariate and multivariable logistic regression analyses. According with normal uNGAL and uNGALC values, previously reported, echocardiographic data were explored as continuous variables and uNGAL and uNGALC were expressed dichotomously.

In the univariate analysis, LA_TEV_, TR_Vmax_, LAV_max_, LVIDDn and VTI_mit_/VTI_Ao_ were independent predictor of increased uNGAL and uNGALC, however only LA_TEV_ [(OR: 3.26, 95% CI: 1.13–9.38), P = 0.03 for NGAL and (OR: 4.62, 95% CI: 1.44–14.88), P = 0.01 for NGALC] and TR_Vmax_ [(OR: 1.77, 95% CI: 1.16–2.73), P < 0.01 for NGAL and (OR: 1.52, 95% CI: 1.01–2.30), P = 0.05 for NGALC] remained statistically significant in the multivariable analysis. ROC curve analysis showed the best cutoff value of LA_TEV_ > 0.76 ml/kg (AUROC: 0.68, 95% CI: 0.56–0.80; Se: 90.6%; Sp: 38.6%) and TR_Vmax_ m/s > 2.31 m/s (AUROC: 0.71, 95% CI: 0.59–0.83; Se: 72.7%; Sp: 68.2%) for identifying increased uNGAL. The best cutoff value of LA_TEV_ > 1.11 ml/kg (AUROC: 0.75, 95% CI: 0.64–0.86; Se: 78.9%; Sp: 60.5%) and TR_Vmax_ > 2.27 m/s (AUROC: 0.66, 95% CI: 0.53–0.78; Se: 71.8%; Sp: 63.2%) for identifying increased uNGALC.

Based on our results LA_TEV_ and TR_Vmax_ are associated with increased uNGAL and uNGALC. These parameters might detect patients at higher risk to develop kidney damage in course of MMVD.

**Disclosures**

No disclosures to report

## 23 ESVC‐O‐23

### 23.1 ESVC ‐ European Society of Veterinary Cardiology

#### 23.1.1 Effect of the sodium‐glucose cotransporter‐2 inhibitor dapagliflozin on platelet aggregation response in Cavalier King Charles Spaniels with and without cardiac disease

##### 23.1.1.1 A Speitzer ^1^, E Øgelund ^1^, MJ Reimann ^1^, JE Møller ^2^, GF Skovsted ^1^, MK Lyhne ^1^, S Verma ^3^, I Ljungvall ^4^, J Häggström ^4^, L. Olsen ^1^

###### 23.1.1.1.1 ^1^ University of Copenhagen Frederiksberg C Denmark; ^2^ Copenhagen University Hospital Rigshospitalet Copenhagen Denmark; ^3^ St. Michael's Hospital and University of Toronto Toronto Canada; ^4^ Swedish University of Agricultural Sciences Uppsala Sweden

Sodium‐glucose cotransporter‐2 (SGLT2) inhibitors have shown beneficial cardiovascular effects in human patients both with and without diabetes mellitus. The exact mechanisms behind the cardiovascular protection is still unknown, but platelet inhibition has been suggested to play a role. The aim of the present study was to examine if the SGLT2 inhibitor dapagliflozin (DAPA) inhibited *in vitro* platelet aggregation response in Cavalier King Charles Spaniels (CKCS). Secondary aims were to evaluate if the degree of inhibition was associated with DAPA concentration, age, sex, platelet count and preclinical myxomatous mitral valve disease (MMVD), and if nitric oxide (NO) combined with prostacyclin enhanced the inhibitory effect of DAPA.

Inhibitory effects of DAPA at concentrations clinically relevant for human patients (4 μM) and higher (30 μM) were evaluated in 26 CKCS with and without preclinical MMVD using light transmission aggregometry. Aggregation response variables were maximal aggregation response, aggregation velocity and area under the aggregation curve. Inhibitory effects of 30 μM DAPA were found on all platelet aggregation response variables (*P*<0.01), whereas 4 μM slightly increased platelet aggregation response (*P*<0.05). The inhibitory effect of DAPA decreased with advancing age, higher platelet counts and preclinical MMVD, however the findings were not consistent for all platelet aggregation response variables. NO (100 nM sodium nitroprusside) and prostacyclin (1 nM iloprost) did not enhance the inhibitory effect of DAPA. In conclusion, DAPA significantly inhibited platelet aggregation in CKCS at a high concentration, but not at a lower human clinical relevant concentration. Further studies are warranted to elucidate effects of SGLT2 inhibitors in dogs with cardiovascular disease.

**Disclosures**

Disclosures to report, please report below

Dr. Reimann is currently employed at Boehringer Ingelheim. Dr. Møller has a research grant from Abiomed outside submitted work. Dr. Verma holds a Tier 1 Canada Research Chair in Cardiovascular Surgery; and reports receiving research grants and/or speaking honoraria from Amarin, Amgen, AstraZeneca, Bayer, Boehringer Ingelheim, Eli Lilly, EOCI Pharmacomm Ltd, HLS Therapeutics, Janssen, Novartis, Novo Nordisk, Pfizer, PhaseBio, Sanofi, Sun Pharmaceuticals, and the Toronto Knowledge Translation Working Group. He is the President of the Canadian Medical and Surgical Knowledge Translation Research Group, a federally incorporated not‐for‐profit physician organization. Dr. Häggström and Dr. Ljungvall have received funding from Boehringer Ingelheim Animal Health GmbH within the last 5 years for research (outside submitted work), travel, speaking fees, consultancy fees, and/or preparation of educational materials. Dr. Lyhne, Dr. Skovsted and Dr. Olsen are affiliated with a research center at University of Copenhagen supported by Novo Nordisk A/S.

## 24 ESVC‐O‐24

### 24.1 ESVC ‐ European Society of Veterinary Cardiology

#### 24.1.1 Inhibitory effect of the active pimobendan metabolite UDCG 212 on platelet aggregation in Cavalier King Charles Spaniels

##### 24.1.1.1 A Petersen ^1^, M Wermer Steen ^1^, JE Møller ^2^, J Häggström ^3^, I Ljungvall ^3^, S Cremer ^1^, L. Olsen ^1^, MJ Reimann ^1^

###### 24.1.1.1.1 ^1^ University of Copenhagen Frederiksberg C Denmark; ^2^ Copenhagen University Hospital Rigshospitalet Copenhagen Denmark; ^3^ Swedish University of Agricultural Sciences Uppsala Sweden

Pimobendan is used to treat dogs with cardiac volume overload due to myxomatous mitral valve disease (MMVD) in dogs and is metabolized to a potent active metabolite; UDCG 212. Studies indicate an *in‐vitro* inhibitory effect of pimobendan on platelet aggregation response at high concentrations.

The study aimed to investigate the *in‐vitro* effect of UDCG 212 on platelet aggregation response at therapeutic (0.09 μM) and high (10 μM) concentrations in privately owned Cavalier King Charles Spaniels (CKCS) with no or preclinical MMVD. Secondary aims were to investigate if the effect of UDCG 212 was influenced by selected clinical variables (sex, age, platelet count in platelet rich plasma (PRP), or preclinical MMVD).

This prospective study included venous blood samples from 26 CKCS with no or preclinical MMVD not receiving medical treatment. Light transmission aggregometry was used to assess adenosine diphosphate‐induced platelet aggregation response (area under the curve (AUC), maximal aggregation (MaxA), and velocity (Vel)).

UDCG 212 inhibited platelet aggregation response (AUC, MaxA and Vel) at both therapeutic and high concentrations (*P*<0.0001). Multivariable regression analysis indicated that the inhibitory effect of UDCG 212 (MaxA) was less pronounced in CKCS in with preclinical MMVD (*P*=0.013). Furthermore, increasing platelet count in PRP was associated with a more rapid inhibitory effect (Vel) (*P*=0.019).

In conclusion, UDCG 212 had a significant inhibitory effect on *in‐vitro* platelet aggregation at both therapeutic and high concentrations. Further studies are warranted to determine possible clinical implications with regard to beneficial antiplatelet effects and risk of bleeding.

**Disclosures**

Disclosures to report, please report below

Dr. Reimann is currently employed at Boehringer Ingelheim. Dr. Møller has a research grant from Abiomed outside submitted work. Dr. Häggström and Dr. Ljungvall have received funding from Boehringer Ingelheim Animal Health GmbH within the last 5 years for research (outside submitted work), travel, speaking fees, consultancy fees, and/or preparation of educational materials. Dr. Cremer is currently employed at Coloplast A/S. Dr. Olsen is affiliated with a research center at University of Copenhagen supported by Novo Nordisk A/S.

## 25 SCH‐O‐1

### 25.1 SCH ‐ Society of Comparative Hepatology

#### 25.1.1 Copper‐associated chronic hepatitis in Cavalier King Charles Spaniels

##### 25.1.1.1 R. Nivy, S. Kuzi, I. Gajanayake, A. Berkowitz, P.J. Watson

###### 25.1.1.1.1 Koret School of Veterinary Medicine, Hebrew University of Jerusalem, Israel Tel‐Aviv Israel

Abnormal hepatic copper accumulation engenders oxidative stress and might culminate in the development of copper‐associated chronic‐hepatitis (CuCH).

Twelve Cavalier King Charles Spaniels (CKCS) with CuCH were recruited. Clinical presentation highly varied and included decreased appetite (n=7) and diarrhea/vomiting (n=7). Portal hypertension and ascites were documented in 5 (41%) dogs. Increased transaminase activities (11 dogs, 92%) and elevated biliary enzyme concentrations (8 dogs, 70%) were common. Six dogs (50%) had ≥3 abnormalities in measures of liver function, including hypocholesterolemia, hypoalbuminemia, hyperbilirubinemia, decreased urea concentration and hyperammonemia.

All tested dogs were homozygous negative for the COMMD1 deletion mutation reported in Bedlington terriers, but homozygous positive (n=5) or heterozygous (n=4) for the ATP7B mutation reported in Labrador retrievers.

The median copper score was 3/5. Salient histological findings included marked architectural distortion by regenerative nodules, severe, often bridging, fibrosis, moderate inflammatory infiltrate, biliary ductular reaction and rare necrosis. Centrilobular copper accumulation characterized early cases with minimal fibrosis, but in most cases, where fibrosis was significant, copper accumulation was most conspicuous within regenerative nodules.

Copper chelation, in addition to anti‐inflammatory and antioxidant therapy, resulted in normalization of alanine transaminase activity in 5/6 dogs, including resolution of ascites and liver function abnormalities in one dog. The remaining dogs died despite (n=1) or without (n=6) chelation therapy.

Considering the findings herein, CuCH should be considered in CKCS with suspected liver disease. Long‐term prognosis, albeit based on a small number of dogs, seems favorable in dogs receiving chelation therapy, notwithstanding the presence of previously reported negative prognostic markers.

**Disclosures**

No disclosures to report

## 26 SCH‐O‐2

### 26.1 SCH ‐ Society of Comparative Hepatology

#### 26.1.1 Clinical findings, bacteria implicated and antimicrobial resistance in 28 dogs with bacterial cholangitis in Spain: a multicentric retrospective study.

##### 26.1.1.1 A. Rodriguez‐Cobos ^1^, L. Feo Bernabe ^2^, C. Martinez ^3^, S. Atencia ^1^

###### 26.1.1.1.1 ^1^ VETSIA Veterinary Hospital Leganés Spain; ^2^ Anicura Ars Veterinaria Barcelona Spain; ^3^ AUNA Especialidades Veterinarias ‐ IVC Evidensia Valencia Spain

Bacterial cholangitis (BC) is an increasingly reported disease. *Escherichia Coli* and *Enterococcus spp*. have been described as the most common agents involved in the BC in previous studies, with a high percentage of antibiotic resistance.

The aim of this retrospective study was to describe the most common clinicopathologic abnormalities, main bacteria involved, antimicrobial susceptibilities and survival on a population of dogs diagnosed with BC from two referral hospitals in Spain.

A total of 28 client owned dogs with histopathological‐confirmed diagnosis of BC were included in this study. All patients had a positive bile and/or liver culture. Fourteen patients presented with acute clinical signs (< 1 week) and 14 with chronic disease (> 1 week). Most frequent clinical signs at presentation were vomiting (23/28, 82%) and hyporexia/anorexia (20/28, 71%). Increased liver enzyme activities were the most common clinical pathology finding (27/28), followed by hyperbilirubinaemia (16/26), leukocytosis (13/28), and hypercholesterolaemia (9/26).

Bacterial growth was identified in 20/24 (83.3%) and 17/21 (81%) of the liver and bile cultures respectively. The most frequent bacteria isolated were *E. Coli* (10/41, 24%), *Klebsiella pneumoniae* (4/41, 10%), *Pseudomonas aeruginosa* (4/41, 10%) and *Enterococcus faecium* (3/41, 7.32%). Multiple antibiotic resistant bacteria (MRB) were detected, with 56% of them showing resistance to more than five antibiotics. Eighty percent of *E. Coli* and 100% of *E. faecium* isolates were susceptible to amoxicillin‐clavulanate.

A Kaplan‐Meier curve was performed to determine the median survival time (MST) and to evaluate the relationship between MST, chronicity of the disease, and infection by MRB.

MST of this population of dogs was 298 days (96% CI: 0‐621). An inverse relationship between MST and chronicity of the disease was found (p<0.05). Cases being infected by MARB did not have consequences on MST (p=0.596).

*E. Coli* was isolated in only one of the nine patients who had received antibiotics prior to referral, and it showed resistance to amoxicillin clavulanate. This result justifies performing liver or bile cultures in patients who do not respond to antibiotic treatment to evaluate if a dose adjustment or a change of antibiotic therapy is needed.

In conclusion, *E. Coli* was the most common bacteria involved in the BC in this population of dogs, and it presented higher susceptibilities to amoxicillin clavulanate than previously reported. This indicates that amoxicillin clavulanate could be used as first line antibiotic therapy while waiting for final culture results.

**Disclosures**

Disclosures to report, please report below

Alfredo Rodríguez‐Cobos's ECVIM‐CA Residency programme is partially funded by Idexx Laboratories

## 27 SCH‐O‐3

### 27.1 SCH ‐ Society of Comparative Hepatology

#### 27.1.1 Prevalence and clinical relevance of cholelithiasis in cats: a multicentric retrospective study

##### 27.1.1.1 C. Duperrier‐Simond ^1^, A. Brunet ^1^, S. Amoyal ^2^, G. Bencheckroun ^3^, J. Hernandez ^2^, L. Lecot ^3^, J.L. Cadoré ^1^, E. Krafft ^1^

###### 27.1.1.1.1 ^1^ Université de Lyon, VetAgro Sup, Campus vétérinaire de Lyon Marcy‐l'Étoile France; ^2^ ONIRIS Nantes France; ^3^ Ecole Nationale Vétérinaire d'Alfort Maisons Alfort France

While recent data suggest that cholelithiasis are an uncommon and mainly incidental finding in dogs, current literature on this topic is scarce in cats.

This multicentric retrospective study aimed to report prevalence, clinical presentation and management of cholelithiasis in cats.

Electronic database from 3 veterinary hospitals was searched for cats diagnosed with cholelithiasis by ultrasonography between January 2010 and December 2021. Cholelithiasis was classified as incidental or symptomatic depending on clinico‐pathological signs, biliary tract ultrasonographic appearance and presence of another disease explaining the clinical presentation. Statistical analysis was carried out using Pearson's Chi‐squared and Mann‐Whitney U tests (significance set at p<0.05). The prevalence was calculated by dividing the number of cases by the number of cats that underwent abdominal ultrasonography during the same time period.

Ninety‐eight cats (median age 12 years, IQR:9‐14) were included (prevalence 0.8%; CI:0.64‐0.96). Cholelithiasis was considered incidental in 40.8% and symptomatic in 59.1% of cases. Age, sex and weight were not statistically different between groups. Symptomatic cases showed more often multiple choleliths in different locations. Among symptomatic cats (n=58), cholangitis and/or extrahepatic bile duct obstruction (EHBDO) were diagnosed in 41.3% and 43% respectively.

Overall, 43% of cats were treated with antibiotics, 22% with ursodesoxycholic acid; 40% of obstructive cases were treated surgically. Persistent/relapsing clinical signs were observed in 18.2% of cats with follow‐up.

This study is the first one to describe a large population of cats with choleliths and suggests that cholelithiasis may be clinically relevant in cats. Concurrent diagnosis of cholangitis or EHBDO was common.

**Disclosures**

No disclosures to report

## 28 SCH‐O‐4

### 28.1 SCH ‐ Society of Comparative Hepatology

#### 28.1.1 Does type and amount of food influence postprandial serum bile acid concentrations in healthy dogs?

##### 28.1.1.1 N. Devriendt ^1^, M. Toom ^1^, S. Minnoye ^1^, A. Hellemans ^1^, E. Stock ^1^, G. Junius ^2^, H. De Rooster ^1^

###### 28.1.1.1.1 ^1^ Ghent University Merelbeke Belgium; ^2^ Medvet Antwerp Belgium

In dogs with liver disorders, serum bile acid concentrations (sBA) are often determined, with postprandial sBA being more sensitive but less specific for the diagnosis of liver disease. The aim of the current study was to identify whether the type and amount of food influences the concentrations of postprandial sBA in healthy dogs.

Forty healthy client‐owned dogs, not receiving any medication, were prospectively enrolled. Inclusion criteria were the absence of any abnormalities on haematology, serum biochemistry and abdominal ultrasound, and the presence of normal preprandial sBA. Different feeding regimes were randomly assigned: two teaspoons of a recovery diet (RD), two teaspoons of a liver‐supportive diet (LD), 10% resting energy requirements (RER) RD, 10% RER LD, or 50% RER LD.

Median preprandial sBA was 2 μmol/L (0‐14 μmol/L) and did not differ between dogs receiving different feeding regimes (p=0.502). Wilcoxon matched‐pair signed‐rank tests revealed that postprandial sBA were only significantly higher than preprandial sBA in dogs receiving 10% RER RD and 50% RER LD (p=0.039 and p<0.001, respectively).

In order to have a consistent gallbladder contraction and, consequently, a significant increase in postprandial sBA in healthy dogs, either 10% RER RD or 50% RER LD should be administered. Although a LD would be preferable in dogs with liver disease, sick dogs might refuse to eat as much as 50% RER and such amount of food precludes sedation or anaesthesia for further diagnostic work‐up.

**Disclosures**

No disclosures to report

## 29 SCH‐O‐5

### 29.1 SCH ‐ Society of Comparative Hepatology

#### 29.1.1 Serum liposoluble vitamins in canine cholestatic liver disease

##### 29.1.1.1 V. Habermaass, F. Bartoli, E. Gori, A. Pierini, P.A. Erba, C. Mariti, S. Citi, V. Marchetti

###### 29.1.1.1.1 University of Pisa Pisa Italy

Both human and canine chronic cholestatic diseases can progressively lead to liver injury and failure subsequent from the biliary tract impairment. Compared to healthy people, chronic cholestatic patients have lower serum liposoluble vitamins (A, D, E). Few studies have been performed in veterinary medicine to evaluate whether vitamins may vary in canine cholestatic hepatopathic patients. Dogs with gallbladder mucocele, chronic liver disease and congenital extrahepatic portosystemic shunts showed low serum vitamin D. Also, vitamin A was found to be low in 16 dogs with chronic liver disease and congenital extrahepatic portosystemic shunts.

This study aimed to compare liposoluble vitamin levels between chronic cholestatic disorder and healthy dogs.

In this prospective case‐control study, 102 client‐owned dogs with cholestatic chronic hepatopathy (group A) and 24 healthy blood‐donor dogs (group B) were included. Cholestatic chronic hepatopathy diagnosis was based on both blood biochemistry profile (2 or more between alkaline phosphatase >250 U/L, gamma‐glutamyl transferase > 11 U/L, total bilirubin > 0.3 mg/dL, cholesterol >280 mg/dL) and signs of biliary tract disease at abdominal ultrasound (i.e., biliary sludge or immobile echogenic bile accumulation, abnormalities of the gallbladder wall, enlarged and/or tortuous extrahepatic bile tract). Dogs with recent liposoluble vitamin supplementation were excluded. To measure vitamin concentrations, ‐80°C stored leftover serum samples of both groups were used. 25‐hydroxycholecalciferol, α‐tocopherol and retinol, respectively vitamin D, E and A metabolites, were measured with High Performance Liquid Chromatography (HPLC) technique. Data were expressed as median and range and Mann‐Whitney U‐test were used to compare vitamins between groups.

25‐hydroxyvitamin D and tocopherol were significatively lower in group A than group B (median 5.28 nmol/mL (0.76‐33.12) vs 11.6 nmol/mL (3.7‐37.9), p<0.001; median 13.7 nmol/mL (0.3‐269.1) vs 27.1 nmol/mL (8.8‐86.7), p=0.02). Retinol was significantly higher in group A than group B (median 4.11 nmol/mL (0.02‐21.48) vs 2.29 nmol/mL (1.07‐11.97), p=0.037.

Vitamins D and E were lower if cholestatic disease was present. In agreement with what is reported for humans, cholestasis and biliary alteration may affect lipids and liposoluble compounds absorption, resulting in lower blood liposoluble vitamins concentration.

Contrarily, vitamin A was higher if cholestatic chronic hepatopathy was present. This result is in line with some recent human studies, where retinol demonstrate to increase as an expression of dysregulated vitamin A and related metabolites homeostasis in the Ito cells and hepatocytes.

**Disclosures**

No disclosures to report

## 30 ESVNU‐O‐1

### 30.1 ESVNU ‐ European Society of Veterinary Nephrology and Urology

#### 30.1.1 Long‐term outcome of dogs recovering from acute kidney injury: 132 cases (2015‐2021)

##### 30.1.1.1 M. Bar Nathan, H. Chen, D. Rimer, G. Segev

###### 30.1.1.1.1 Koret School of Veterinary Medicine, The Hebrew University of Jerusalem Rehovot Israel

The short‐term outcome of dogs with acute kidney injury (AKI) is described, however information regarding the long‐term prognosis of dogs surviving AKI is very limited. The aim of this study is to determine the proportion of dogs recovering from AKI that have normalization of serum creatinine (sCr) at discharge and during the follow up period, to describe the long‐term outcome, and to identify predictors for creatinine normalization and long‐term outcome.

This retrospective study included 132 dogs recovering with AKI and surviving ≥30 days post‐discharge. Estimated median survival time (MST) was 1322 days (95% CI, 1147‐1626). Normalization of sCr was documented in 55% of the dogs at discharge and in additional 20% during the follow‐up, resulting in overall sCr normalization in 75% of the dogs. The proportion of dogs with sCr normalization decreased with the increase in AKI Grade (*P* = 0.019). All dogs that remained azotemic were classified as IRIS CKD Stage 2. Long‐term survival was not associated with creatinine normalization (*P* = 0.633) but was associated with the etiology (*P* = 0.004).

This study demonstrates that the long‐term survival of dogs with AKI is longer than previously described. Estimated MST of dogs with creatinine normalization was not different compared with dogs that developed azotemic CKD, presumably, due to a slow progression rate. The etiology is an important factor determining creatinine normalization and the long‐term survival, demonstrating the importance of reversibility of renal injury rather than its severity.

**Disclosures**

No disclosures to report

## 31 ESVNU‐O‐2

### 31.1 ESVNU ‐ European Society of Veterinary Nephrology and Urology

#### 31.1.1 Calcium‐phosphate metabolism in normocalcaemic non‐pedigree cats with upper urinary tract stones

##### 31.1.1.1 R. Geddes ^1^, P. Tang ^2^, L. Davison ^1^, J. Elliott ^2^, H. Syme ^1^

###### 31.1.1.1.1 ^1^ Royal Veterinary College North Mymms United Kingdom; ^2^ Royal Veterinary College London United Kingdom

Feline upper urinary tract stones (UUTS) are typically calcium oxalate, but the majority of UUTS‐forming cats have total and ionised normocalcaemia. We hypothesized that UUTS‐forming cats have disrupted calcium‐phosphate metabolism despite normocalcaemia.

Non‐pedigree, neutered cats with ionised calcium concentrations [iCa] ≤1.4mmol/l, with or without current or historic UUTS on ultrasound, were prospectively recruited if they had had stable serum creatinine concentrations <250μmol/l for at least one month. Control cats had to be ≥9 years of age. Exclusion criteria included medications or illnesses known to alter calcium‐phosphate homeostasis, other than CKD. Residual samples were used to measure variables involved in calcium‐phosphate homeostasis. Data (mean±SD or median (IQR)) were assessed for normality and groups compared using t‐tests or Mann‐Whitney U tests.

Nineteen control cats (5 female) were included with the following diagnoses: chronic enteropathy (n=6), healthy cat (n=4), CKD (n=7), struvite cystolithiasis (n=1), historic acromegaly (n=1). The 19 stone‐formers (10 female) had significantly higher [iCa] (1.36±0.03 vs 1.32±0.05 mmol/l; P=0.003) and fractional excretion of calcium (0.73 (0.27‐1.33) vs 0.19 (0.15‐0.89) %; P=0.045), and were younger (9.3±3.9 vs 13.8±3.1 years; P<0.001), but demonstrated no difference in serum creatinine, phosphate or total calcium, plasma FGF23 or PTH, urine specific gravity, urinary calcium:creatinine, phosphate:creatinine or fractional excretion of phosphate compared to controls. Consumption of ≥50% clinical renal diet was not different between groups (7/19 stone‐formers versus 10/19 controls; P=0.51).

Increased renal calcium excretion via an increased filtered calcium load and reduced tubular calcium reabsorption may contribute to calculi formation in normocalcaemic UUTS‐forming cats.

**Disclosures**

Disclosures to report, please report below

This study was funded by a PetPlan Charitable Trust Pump Priming grant. R. Geddes has received speaker honoraria, consulting fees or grant funding from an RVC Internal Grant, The Academy of Medical Sciences, Everycat Foundation, Boehringer Ingelheim and Royal Canin SAS. P.K. Tang is undertaking a PhD studentship funded by Royal Canin SAS. L.J.Davison has received speaker honoraria, consulting fees or grant funding from Nuffield Department of Medicine, PetPlan Charitable Trust, BSAVA Petsavers, Evetts‐Luff Feline Endowment Fund, American Kennel Club Canine Health Foundation, Morris Animal Foundation, Hong Kong Jockey Club, Academy of Medical Sciences, Everycat Foundation, UKRI, ECVIM Clinical Studies Fund, MRC Clinician Scientist Fellowship, London Inter‐Disciplinary Doctoral Training Partnership, Dechra, Royal Canin SAS, Juvenile Diabetes Research Foundation, University of Dublin Seedcorn grant, Wellcome Trust, Improve International. J. Elliott has received speaker honoraria, consulting fees or grant funding from Elanco Ltd, CEVA Animal Health Ltd, Boehringer Ingelheim Ltd, MSD Animal Health Ltd., Orion Incorp, Idexx Ltd, Nextvet Ltd, Waltham Centre for Pet Nutrition, Kindred Biosciences Inc, Invetx Inc, Royal Canin SAS, Idexx Ltd., Zoetis Ltd. Is a member of the International Renal Interest Society. H.M.Syme has received speaker honoraria, consulting fees and grant funding from Royal Canin SAS, Hill's, MSD, Boehringer Ingelheim, Dechra, Vetoquinol, Pfizer and PetPlan Charitable Trust. Is a member of the International Renal Interest Society.

## 32 ESVNU‐O‐3

### 32.1 ESVNU ‐ European Society of Veterinary Nephrology and Urology

#### 32.1.1 Cats with amyloid‐A amyloidosis and urinalysis: proteinuria, electrophoresis and biomarker discovery

##### 32.1.1.1 C. Palizzotto ^1^, S Ferro ^2^, M Weiss ^3^, L Cavicchioli ^2^, C Callegari ^4^, M Manfredi ^5^, L Carcangiu ^5^, F Porporato ^4^, F Rossi ^4^, E Zini ^4^

###### 32.1.1.1.1 ^1^ Anicura Istituto Veterinario di Novara Granozzo con Monticello Italy; ^2^ Università degli studi di Padova Legnaro Italy; ^3^ LABOKLIN GmbH & Co. KG Bad Kissingen Germany; ^4^ Anicura Istituto veterinario di Novara Granozzo con Monticello Italy; ^5^ Università del Piemonte Orientale Novara Italy

Amyloid‐A amyloidosis (AAA) is a protein misfolding disease arising from serum amyloid‐A, an acute phase protein undergoing conformational changes and deposition in extracellular tissues. In cats, AAA is described both as a familial and acquired disease, and is associated with azotemia and proteinuria. Based on necropsy findings, acquired AAA occurs in 45‐70% of shelter cats. Identification of AAA requires tissue biopsies, making the diagnosis difficult in these cats. Measurement of serum amyloid‐A is not useful to diagnose AAA in cats. Hence, the aim of this study was to investigate urine protein profiles and biomarkers to diagnose AAA in cats housed in shelters.

Cats of three shelters were included if leftovers from urine samples collected within 30 days before death were available, and if kidney, liver and spleen were collected post‐mortem for histological examination. To confirm AAA, Congo red stain and immunohistochemistry were performed. Kidney damage was characterized using light microscopy and amyloid deposits were scored under polarized light. Urine protein‐to‐creatinine (UPC) ratio, urine amyloid A‐to‐creatinine ratio (UAAC), sodium dodecyl sulphate‐polyacrylamide gel electrophoresis (SDS‐PAGE) and liquid chromatography‐mass spectrometry (LC‐MS) were performed.

Twenty‐nine shelter cats were included; all were domestic shorthair, 15 were male and 14 female. Nineteen cats had AAA with renal deposition. Compared to cats without AAA, those with AAA had higher UPC ratio [median=3.9 (range=0.6‐12.7) vs. 1.5 (0.6‐3.1); p=0.026] and UAAC ratio [median=0.007 (range=0.000‐0.021) vs. 0.001 (0.000‐0.006); p=0.039]. SDS‐PAGE identified mixed‐type proteinuria in 84% of cats with AAA and in 66% without AAA (p=0.190). Among cats with AAA, those with amyloid localized in the glomerulus were more likely to have marked glomerular‐type proteinuria compared to those with deposition in other parts of the kidney (p=0.038). LC‐MS identified 63 potential biomarkers associated with AAA (p<0.05); among them, urine apolipoprotein E was higher in cats with AAA [median=91x10^6^(range=15.8x10^6^‐713.5x10^6^) vs. 7x10^6^ (0.5x10^6^‐22.7x10^6^); p=0.019].

In conclusion, AAA with renal deposition leads to proteinuria in shelter cats. Urine apolipoprotein E may represent a biomarker to identify affected cats, while urine amyloid A, due to the large overlap between affected and unaffected cases, is of limited use. Urine electrophoresis may predict the glomerular localization of amyloid in cats with AAA and renal involvement.

**Disclosures**

No disclosures to report

## 33 ESVNU‐O‐4

### 33.1 ESVNU ‐ European Society of Veterinary Nephrology and Urology

#### 33.1.1 Tissue S100/calgranulin expression and blood neutrophil‐to‐lymphocyte ratios in urothelial carcinoma in dogs

##### 33.1.1.1 J. Weinekötter ^1^, C. Gurtner ^2^, M. Protschka ^1^, W. Bomhard ^3^, D. Böttcher ^1^, A. Schlinke ^1^, G. Alber ^1^, S. Rösch ^4^, J.M. Steiner ^5^, J. Seeger ^1^, G. Oechtering ^1^, R. Heilmann ^1^

###### 33.1.1.1.1 ^1^ College of Veterinary Medicine, University of Leipzig Leipzig Germany; ^2^ Vetsuisse Faculty, University of Bern, Switzerland Bern Switzerland; ^3^ Specialty Center for Veterinary Pathology Munich Germany; ^4^University of Veterinary Medicine Hannover Hannover Germany; ^5^ Texas A&M University, Gastrointestinal Laboratory College Station United States

Urothelial carcinoma (UC) is the most common neoplasm of the canine lower urinary tract, and clinical signs of UC can easily be confused with those of urinary tract infection or urolithiasis. Diagnosis and treatment are challenging due to a lack of disease‐specific markers and treatments. The S100A8/A9 complex and S100A12 protein are Ca^2+^‐binding alarmins that have shown promise as urinary screening markers (individual protein concentrations and their ratios) for UC in dogs. The neutrophil‐to‐lymphocyte ratio (NLR) can also help to distinguish certain neoplastic from inflammatory conditions. This study aimed to evaluate the tissue expression of the S100/calgranulins and the blood NLR in dogs with UC.

Urinary bladder and/or urethral tissue samples (except for one UC case, all either surplus material or obtained post‐mortem) from dogs with UC (n=10), non‐neoplastic inflammatory lesions (NNUTD; n=6), or no histologic lesions (n=11) were used for S100A8/A9‐ and S100A12‐immunohistochemistry and evaluation of positive staining cells among these groups of dogs (ethics approval #TVA23‐18). Blood NLRs (extracted from the medical records) were compared between dogs with UC (n=9) and NNUTD (n=7), and a receiver operating characteristic curve was used to determine the best cut‐off NLR to differentiate UC from NNUTD. Complete patient information was retrieved from medical records and follow‐up telephone consultations. Statistical significance was set at *P*<0.05.

Tissue S100A12‐positive (S100A12^+^) cell counts were significantly higher in dogs with lower urinary tract disease than control dogs (UC: *P*=0.0267; NNUTD: *P*=0.0049), with no significant difference between both disease groups. Tissue S100A8/A9‐positivity was higher with NNUTD than UC and healthy controls, but the differences were not statistically significant. The S100A8/A9^+^‐to‐S100A12^+^ ratio was significantly decreased in neoplastic and inflamed lower urinary tract tissues compared to histologically normal specimens (UC: *P*=0.0062; NNUTD: *P*=0.0030). NLRs were significantly higher in dogs with UC than in dogs with NNUTD, and a cut‐off NLR of ≤5.46 best distinguished UC from NNUTD. Higher NLRs were associated with poor overall survival times (*P*=0.0063).

These findings confirm a role of the S100/calgranulins in the immune response to both inflammatory and neoplastic lower urinary tract diseases in dogs. However, the tissue S100/calgranulin expression differs from previously reported urinary S100/calgranulin concentrations. Further investigation of the S100/calgranulin pathways in UC and their potential as diagnostic or prognostic tools and potential therapeutic targets is warranted. The NLR as a routinely available marker might be a useful surrogate to distinguish UC from inflammatory conditions.

**Disclosures**

No disclosures to report

N/A

## 34 ESVNU‐O‐5

### 34.1 ESVNU ‐ European Society of Veterinary Nephrology and Urology

#### 34.1.1 Follicular cystitis in dogs – Role of intramural E.coli bacteria

##### 34.1.1.1 S.J. Viitanen, N. Salonen, K. Kegler

###### 34.1.1.1.1 University of Helsinki University of Helsinki Finland

Follicular cystitis refers to a non‐specific benign inflammatory change in the urinary bladder wall characterized by follicles of lymphoid tissue in the lamina propria. Etiology of the lesions is not fully known, but in human medicine, it has been connected to chronic urinary tract infections (UTIs) and chemical irritants.

Eight dogs diagnosed with follicular cystitis were retrospectively identified from patient records. All dogs were female (4/8 intact, 4/8 spayed) and median age of the dogs was 2.7 years (IQR 0.6‐4.9 years). All dogs had a history of recurrent UTIs: Median duration of clinical signs at presentation was 7 months (IQR 3‐17 months) and all dogs had experienced several previous bacterial UTIs (range 2‐8). All dogs underwent full clinical evaluation including ultrasonography (8/8), cystoscopy (7/8) and histopathologic evaluation of bladder wall biopsies (8/8). Surplus paraffin embedded biopsies were retrieved from pathology archives and stained with an in situ hybridization (ISH) probe targeting E.coli RNA (RNAscope® technology, Probe B‐E.Coli‐16SrRNA cat# 433291, Advanced Cell Diagnostics, Newark, CA). Evaluation of ISH stained biopsies revealed intramural E.coli in all dogs (intraepithelial localization in 3/8, submucosal in 8/8 and intrafollicular in 6/8 dogs). Severity of intramural bacterial infiltration varied from mild (3/8) to moderate (4/8) and marked (1/8).

In our study, follicular cystitis was diagnosed in female dogs with recurrent UTIs. Intramural E.coli bacteria were demonstrated in bladder wall biopsies in all dogs and represents a likely etiology for the development of follicular lesions in these dogs.

**Disclosures**

No disclosures to report

Sanna Viitanen has received a research grant for this study from the Foundation of Finnish Veterinary Research.

## 35 ESVNU‐O‐6

### 35.1 ESVNU ‐ European Society of Veterinary Nephrology and Urology

#### 35.1.1 Owner's experiences of caring for a cat with chronic kidney disease (CKD) in the United Kingdom

##### 35.1.1.1 H. Reyes‐Hughes ^1^, J. Elliot ^2^, A. Hibbert ^3^, N. Finch ^3^

###### 35.1.1.1.1 ^1^ University of Bristol Bristol United Kingdom; ^2^ Highcroft Veterinary Group Bristol United Kingdom; ^3^ Langford Vets Bristol United Kingdom

The aim of this study was to describe owner reported management, experiences and feelings associated with caring for a cat with CKD. Data was collected and analysed from one‐hundred responses of UK owners of cats with CKD to an on‐line multiple choice and Likert scale questionnaire. Data was collected over three months. Question themes included medical management and owner feelings associated with caring for their cat. Responses were analysed using descriptive statistics.

The most frequent management reported included ensuring adequate hydration (91%; with 19% of owners administering subcutaneous fluid therapy) and feeding a renal diet (76%). Of the owners feeding a renal diet 71% stated their decision was based on a veterinary recommendation. Nearly one third of owners visited a veterinary practice with their cat every one to two months with only 3% visiting >12 months. 84% of owners sourced information related to their cat's CKD from their vet. 91% of owners experienced anxiety associated with caring for their cat with CKD, with 72% feeling extreme anxiety about prognosis and 63% about quality of life. 66% of owners reported a significant change to their daily routine since their cat's diagnosis and 37% felt that caring for their cat had negatively impacted them.

The findings suggest that owners of cats with CKD are often highly motivated, although the data likely reflects a biased population. Furthermore, that vets have a key influential role in the decision making of owners. Many owners expressed extreme feelings of anxiety and caregiver burden. There is a lack of awareness of caregiver burden which can lead to stress and symptoms of depression. Management of CKD often involves a high level of supportive care and compliance from the owner. To ensure optimal management, the veterinary profession needs to consider caregiver burden when recommending individual management plans to owners of cats with CKD.

**Disclosures**

No disclosures to report

## 36 ESVNU‐O‐7

### 36.1 ESVNU ‐ European Society of Veterinary Nephrology and Urology

#### 36.1.1 Essential and non‐essential serum amino acids in dogs at different stages of chronic kidney disease

##### 36.1.1.1 I Lippi ^1^, F Perondi ^1^, A Pierini ^1^, E Gori ^1^, F Bartoli ^2^, C Mariti ^1^, V Marchetti ^1^

###### 36.1.1.1.1 ^1^ University of Pisa San Piero a Grado‐Pisa Italy; ^2^ Department of Translational Research and New Technologies in Medicine and Surger Pisa Italy

Serum aminoacidic abnormalities were documented in human CKD, and characterized by reduction in essential amino acids (EAA), and increase in non‐essential amino acids (NEAA). These disorders were associated with CKD complications, such as metabolic acidosis, and malnutrition.

The aim of the present study was to evaluate serum EAA and NEAA in dogs at different stages of CKD, with special reference to CaxP abnormalities, metabolic acidosis and protein energy wasting syndrome (PEW). EAA (L‐Histidine (HIS), L‐Isoleucine (ILE), L‐Leucine (LEU), L‐Lysine (LYS), Methionine (MET), L‐Phenylalanine (PHE), L‐Threonine (THR), Tryptophan (TRP), L‐Valine (VAL)), and NEAA (L‐Alanine (ALA), L‐Arginine (ARG), L‐Aspartic acid (ASP), L‐Cysteine (CYS), L‐Glutamic acid (GLU), Glycine (GLY), Proline (PRO), L‐Serine (SER), L‐Tyrosine (TYR)) were analyzed with HPLC on left over serum samples of CKD (n=62), and clinically healthy dogs (n=25). CKD dogs were classified according to IRIS staging, and subclassified according to the presence of abnormal CaxP (CaxP>70 mg^2^/dL^2^), metabolic acidosis, or PEW. Data were compared by One‐way ANOVA or Kruskal‐Wallis, and t‐test or Mann‐Whitney (p<0.05).

CKD dogs were distributed in IRIS 1 (n=12), IRIS 2 (n=16), IRIS 3 (n=14), and IRIS 4 (n=20), and showed lower levels of HIS (p=0.000), ILE (p=0.000), TRP (p=0.000), ALA (p=0.013), CYS (p=0.000), and SER (p=0.002), and higher levels of PRO (p=0.000), LEU (p=0.001), LYS (p=0.000), VAL (p=0.000), ARG (p=0.002), GLU (p=0.002), and GLY (p=0.010). Dogs with abnormal CaxP showed significantly higher levels of CYS (p=0.003), and lower levels of TRP (p=0.025); dogs with metabolic acidosis showed significantly higher levels of CYS (p=0.003) and TRP (p=0.025); and dogs with PEW showed significantly lower levels of TRP (p=0.003) , VAL (p=0.003), ALA (p=0.001), ILE (p=0.001), LEU (p=0.001), LYS (p=0.014), GLY (p=0.045), TYR (p=0.016), HIS (p=0.006), PHE (p=0.049), THR (p=0.007), PRO (p=0.011), and SER (p=,0.031), and significantly higher levels of GLU (p=0.002) and CYS (p=0.010).

CKD dogs showed significant abnormalities in both serum EAA and NEAA levels. Abnormal aminoacidic pattern was evident since IRIS 1, but no significant association with the progression of CKD was noticed. Although serum HIS, ILE and TRP were consistently lower compared to healthy dogs, our patients did not show the typical reduction in EAA, usually seen in human CKD. Abnormal serum levels of some EAA and NEAA may be related to multiple causes, such as increased catabolism rate, worsening of glomerular and tubular functions, and malnutrition. Among the different CKD complications, PEW was associated with the highest number of amino acid abnormalities.

**Disclosures**

No disclosures to report

## 37 ESVNU‐O‐8

### 37.1 ESVNU ‐ European Society of Veterinary Nephrology and Urology

#### 37.1.1 Retrospective evaluation of 22 dogs with leptospirosis requiring intermittent hemodialysis (2018‐2021)

##### 37.1.1.1 A. Ioannou, E.M. Butty , C. Tai, M.A. Labato

###### 37.1.1.1.1 Cummings School of Veterinary Medicine at Tufts University North Grafton United States

Leptospirosis is a widespread zoonotic disease caused by spirochetes of the genus Leptospira. Outcomes of dogs with oliguric or anuric acute kidney injury (AKI) secondary to Leptospirosis and requiring renal replacement therapies are still poorly reported. A study published in 2000 found a survival rate of 86% in the 14 dogs managed with hemodialysis. The aim of this retrospective study was to describe the clinical profile, management, survival to discharge, and long‐term outcomes (> 6 months) of a population of dogs with AKI secondary to leptospirosis requiring intermittent hemodialysis (IHD).

Between January 2018 and December 2021, 22 dogs with leptospirosis required IHD as part of their disease management. The mean age on presentation was 5 (+4) years. The mean duration of illness prior to presentation to our facility was 4.0 (±2.2) days. Twenty‐one (95%) dogs were presented as a referral. On presentation, 16 dogs (72%) were either oliguric or anuric and 13 dogs (59%) were fluid overloaded. The mean blood urea nitrogen (BUN) and creatinine on presentation were 151.4 (±47.2) mg/dL and 9.6 (± 2.6) mg/dL, respectively. For each patient, the mean number of organ systems affected throughout their hospitalization was 3.0 (± 1.5). Renal, hepatic, and coagulation systems were the most commonly affected. The mean number of IHD sessions for each patient was 3.0 (±1.8). The mean hospitalization time was 11.6 (±6.3) days. Fifteen (68.2%) dogs survived to discharge. Of the 7 dogs that did not survive, 4 were euthanized due to lack of improvement or progressive disease, and 3 suffered cardiopulmonary arrest. BUN (p=0.7) and creatinine (p=0.8) concentrations on intake were not associated with survival to discharge. The number of body systems (p=0.01) affected was negatively associated with survival.

Thirteen dogs had long‐term follow‐up and 8 had bloodwork available for IRIS CKD staging based on their most recent recheck. Three (37.5%) dogs were classified as IRIS CKD Stage I at a 6‐month follow‐up. Four (50%) dogs were classified as IRIS CKD Stage II, two at 1‐year and two at 2‐year follow‐up. One dog was classified as IRIS Stage CKD IV at 6‐month follow‐up. The five dogs without bloodwork available were documented to have been euthanized or died within 3 years after discharge.

This study presents the short‐ and long‐term prognosis of a dog population with AKI secondary to leptospirosis requiring management with IHD.

**Disclosures**

Disclosures to report, please report below

MA. Labato serves as a consultant for Boehringer Ingelheim

## 38 ESVNU‐O‐9

### 38.1 ESVNU ‐ European Society of Veterinary Nephrology and Urology

#### 38.1.1 Neutrophil gelatinase‐associated lipocalin and symmetric dimethylarginine in dogs with carcinoma or sarcoma

##### 38.1.1.1 A. Rixon, E. Meyer, S. Daminet, A. Goddard, A. Celliers, T. Kongtasai, P. Pazzi

###### 38.1.1.1.1 Onderstepoort Pretoria South Africa

Elevations in neutrophil gelatinase‐associated lipocalin (NGAL) and symmetric dimethylarginine (SDMA) in humans and/or dogs with tumours, questions the specificity of these renal biomarkers. Studies evaluating concentrations in carcinoma, sarcoma and metastatic disease in light of renal histopathology are lacking.

This retrospective study aimed to evaluate the effect of neoplasia and metastatic disease on NGAL and SDMA in dogs with carcinoma or sarcoma.

Serum NGAL (sNGAL), urinary NGAL (uNGAL), uNGAL/creatinine (uNGAL/Cr), serum SDMA (sSDMA), serum creatinine concentrations and urine specific gravity were determined in stored samples from 48 dogs diagnosed with carcinoma (n=23) or sarcoma (n=25) and 20 healthy, age‐controlled dogs. Sample collection, and complete post‐mortems of tumour‐bearing dogs, were performed in a prior study. Histopathology reports were reviewed for evidence of renal or metastatic disease. Biomarker concentrations were compared between tumour‐bearing dogs without moderate‐severe histopathological renal disease or renal azotaemia (n=39), tumour‐bearing dogs with renal disease (n=9) and unaffected dogs. Additionally, all tumour‐bearing dogs without renal disease but with evidence of metastatic disease (n=21) were compared to dogs without metastatic disease (n=18).

Tumour‐bearing dogs without renal disease had significantly elevated uNGAL (*P*<.001) and uNGAL/Cr (*P*<.001) compared to unaffected dogs, while sNGAL and sSDMA was not significantly different between the two groups. Biomarker concentrations did not significantly differ between tumour‐bearing dogs with or without renal disease, or between dogs without renal disease with or without metastatic disease.

Carcinoma and sarcoma resulted in elevated uNGAL; while sNGAL and sSDMA was not influenced by tumour presence. Metastatic disease did not affect biomarker concentrations.

**Disclosures**

No disclosures to report

## 39 ESVNU‐O‐10

### 39.1 ESVNU ‐ European Society of Veterinary Nephrology and Urology

#### 39.1.1 A cross‐sectional study assessing macroscopic nephrocalcinosis using ultrasonography in cats with chronic kidney disease

##### 39.1.1.1 P.K. Tang ^1^, D.H.N. Van den Broek ^2^, R. Geddes ^1^, R.E. Jepson ^1^, Y.M. Chang ^1^, J. Elliott ^1^

###### 39.1.1.1.1 ^1^ Royal Veterinary College London United Kingdom; ^2^ Utrecht University Utrecht Netherlands

Microscopic nephrocalcinosis is of high prevalence at post‐mortem in feline chronic kidney disease (CKD) and is associated with plasma total calcium concentration. However, it remains to be determined at what point nephrocalcinosis develops and whether this can be detected by imaging and assessed over the cat's life‐time. Therefore, this study evaluated the presence of macroscopic nephrocalcinosis in feline CKD using ultrasonography.

Cats with CKD were prospectively recruited and categorised as hypercalcaemic or normocalcaemic based on their current and historic ionised calcium (iCa) status. Ultrasonography was performed to detect macroscopic nephrocalcinosis. Scans were evaluated by a trained diagnostic imager blinded to the cat's iCa status. Baseline variables at scanning between groups were compared using Mann‐Whitney *U* test, independent sample *t* test and Chi‐square test.

Twenty‐nine euthyroid cats with CKD (IRIS stages 1 [n=1], 2 [n=21], 3 [n=6], 4 [n=1]) were recruited; 15 were hypercalcaemic (baseline iCa≥1.4 mmol/L or baseline iCa>1.37 mmol/L with historic iCa≥1.4 mmol/L) and 14 cats were normocalcaemic (baseline and historic iCa≤1.37 mmol/L). A significantly larger proportion of hypercalcaemic cats had ultrasonographic evidence of nephrocalcinosis compared to normocalcaemic cats (73.3% vs. 35.7%; *P*=0.042). Hypercalcaemic cats had significantly higher plasma albumin concentration than normocalcaemic cats (31.7±1.5 g/L vs. 29.2±3.7 g/L; *P*=0.024). The presence of medullary rim sign on ultrasonography did not differ between groups (hypercalcaemia, 20% vs. normocalcaemia, 14.3%; *P*=1).

Ionised hypercalcaemia is associated with increased prevalence of suspected macroscopic nephrocalcinosis in feline CKD. The progression of nephrocalcinosis and its implications for CKD progression should be assessed longitudinally by ultrasonography.

**Disclosures**

Disclosures to report, please report below

P.K. Tang received PhD studentship funded by Royal Canin SAS. D.H.N. van den Broek received PhD studentship funded by Royal Canin SAS. R. Geddes received funding from Petplan, an RVC Internal Grant, The Academy of Medical Sciences and The Winn Feline Foundation; has a consultancy agreement with Boehringer Ingelheim; speaking honoraria from Boehringer Ingelheim. R. Jepson received funding from PetPlan, Feline Foundation for Renal Research, RVC Internal Grant, PetSavers, and consultancy agreements: Boehringer Ingelheim, Merial, CEVA. Speaking honoraria: Boehringer Ingelheim, Hills Pet Nutrition, CEVA. Y.M. Chang had no conflicts of interest to declare. J. Elliott received funding from Consultancies: Elanco Ltd, CEVA Animal Health Ltd, Boehringer Ingelheim Ltd, MSD Animal Health Ltd., Orion Incorp, Idexx Ltd, Nextvet Ltd, Waltham Centre for Pet Nutrition, Kindred Biosciences Inc, Invetx Inc; grant funding from Elanco Ltd, Waltham Centre for Pet Nutrition, Royal Canin SAS, Idexx Ltd., Zoetis Ltd, CEVA Animal Health, Member of the International Renal Interest Society.

## 40 ESVNU‐O‐11

### 40.1 ESVNU ‐ European Society of Veterinary Nephrology and Urology

#### 40.1.1 Does the iodinated contrast agent administered to dogs during computed tomography imaging reduce the sensitivity of detecting a urinary tract infection?

##### 40.1.1.1 I. Chan, V. Black, M. Costa, E. Barker

###### 40.1.1.1.1 University of Bristol Bristol United Kingdom

Iodine is bactericidal and some iodinated contrast agents have antibacterial properties. Intravenous iodinated contrast agents are used during computed tomography (CT)‐imaging and are rapidly cleared via the kidneys. This raised concern that their use may interfere with the sensitivity of bacterial culture from urine samples collected immediately following their administration. The effect of iopamidol, a non‐ionic iodinated contrast agent, at clinically relevant concentrations on bacterial culture and retrieval before and following refrigerated storage was investigated.

Free‐catch urine samples were collected from eleven dogs following intravenous administration of iopamidol for diagnostic imaging purposes. High‐performance capillary electrophoresis was used to measure iodine concentration in the urine. The effect of iopamidol on reference strains of two common bacterial species (*Escherichia coli* and *Staphylococcus epidermidis*) was then investigated. ‘Mock urine’ comprising 33% (Stuart's) urea broth spiked with iopamidol (to 0, 15, 30, 60, and 150 mg/mL) were inoculated with bacterial stock suspension to three different levels of colony forming units (CFU) [~2x10^2^ CFU/mL, ~2x10^3^ CFU/mL, and ~2x10^4^ CFU/mL]. Duplicate suspensions were stored refrigerated for 0, 24, and 48 hours prior to assessment of growth at 37 °C by serial measurement of optical density at 0, 24, and 48 hours or assessment of bacterial viability by plating 10μl onto blood agar and colony counting.

Urine iopamidol concentrations ranged between 6‐70 mg/mL; this was included in the range of concentrations tested in the subsequent in vitro testing. As iopamidol concentrations increased there was a trend towards increased bacterial viability for *E. coli* at ~2x10^2^ CFU/mL and ~2x10^3^ CFU/mL starting inoculum. Storage tended to have a detrimental effect on bacterial viability. In contrast, there was an inverse relationship between iopamidol concentration and bacterial growth in a urea broth‐based solution, that was most marked for *Staph. epidermidis* at both lower CFUs and increased storage duration prior to incubation at 37 °C. Storage duration had no effect on bacterial growth of *E. coli*.

The results of this study suggest that while iopamidol at clinically relevant concentrations in urine was likely to reduce the rate of growth of bacteria, it did not reduce the retrieval rate of bacteria.

There is no need to collect urine for culture prior to procedures that involve intravenous administration of iopamidol.

**Disclosures**

No disclosures to report

## 41 ESVNU‐O‐12

### 41.1 ESVNU ‐ European Society of Veterinary Nephrology and Urology

#### 41.1.1 Urine gamma‐glutamyl transferase to creatinine ratio in dogs undergoing contrast‐enhanced computed‐tomography examination – a pilot study

##### 41.1.1.1 B. Cuq ^1^, O. Waite ^2^, C. Lassaigne ^1^, R. Shiel ^1^, M. Augusto ^1^, C. Skelly ^1^, L. Mooney ^1^

###### 41.1.1.1.1 ^1^ Univeristy College Dublin School of Veterinary Medicine Dublin Ireland; ^2^ Royal Veterinary College London United Kingdom

Urinary biomarkers such as urinary GGT (uGGT) and urinary GGT:Creatinine ratio (uGGT:Creat), have been reported to be early markers of acute kidney injury (AKI) in veterinary medicine. Contrast‐associated AKI is well documented in people undergoing computed tomography (CT). However, there is no data available in dogs undergoing contrast‐enhanced CT (ce‐CT).

The objectives of the study were to describe uGGT:Creat changes following nonionic iodinated contrast media (ioversol) administration in canine patients undergoing ce‐CT. Free catch urine was collected pre and post CT in 87 dogs undergoing ce‐CT and 9 dogs undergoing non‐contrast enhanced CT (nc‐CT). uGGT:Creat were determined using the Attellica® (Siemens). Dogs with pre‐existing azotaemia were excluded from the study. uGGT:Creat was determined in 18 healthy dogs. Owner consent was obtained prior to enrolment.

uGGT:Creat significantly increased when comparing dogs pre‐ and post‐ce‐CT (median: 0.29 IQR [0.16‐0.48] and 0.45 [0.25‐0.69] respectively; p<0.01). uGGT:Creat was significantly higher in dogs undergoing ce‐CT pre‐ and post‐CT when compared to healthy dogs (0.11 [0.09‐0.15], p<0.01). There was no difference in uGGT:Creat pre‐ and post‐nc‐CT (0.49 [0.15‐0.93] and 0.61 [0.24‐0.90] respectively; p = 0.29). Similarly, uGGT:Creat was higher in dogs undergoing nc‐CT when compared to healthy controls (p=0.01 and p<0.01).

Changes in uGGT were detectable by the Attellica®. The increase in uGGT:Creat in the ce‐CT group and not in the nc‐CT group suggests tubular damage associated with contrast administration, and thus possible contrast‐associated AKI. Further investigations are needed to characterize its clinical relevance and to establish reference intervals on this analyser.

**Disclosures**

No disclosures to report

## 42 ESVNU‐O‐13

### 42.1 ESVNU ‐ European Society of Veterinary Nephrology and Urology

#### 42.1.1 A retrospective analysis of risk factors for thrombotic disease in dogs with protein losing nephropathy

##### 42.1.1.1 L. Fortuna ^1^, H. Syme ^2^

###### 42.1.1.1.1 ^1^ Highcroft Veterinary Referrals Bristol United Kingdom; ^2^ Royal Veterinary College London United Kingdom

Dogs with protein losing nephropathies (PLN) are at increased risk of thrombotic disease (TD). There is limited work exploring risk factors for TD within the subset of dogs with PLN, and these may differ for differing sites of TD. The objective of this study was to compare signalment and clinicopathological data between dogs with PLN with or without TD.

50 dogs with PLN and TD, and 100 controls with PLN but no TD, were identified through a retrospective database search between 2004 and 2021. Signalment, TD location, and clinicopathological data were obtained from the clinical records. Categorical variables were compared with the Fishers exact test, normal data with the independent T test, and non‐normal data with the Mann Whitney U tests. Variables with P<0.1 were included in multivariate analysis.

Breed distribution differed between controls and TD (P=0.024) and between TD locations (P=0.016); Cavalier King Charles Spaniels and Sighthounds were over‐represented for TD. The location of the thrombus was pulmonary in 11, systemic arterial in 24 and systemic venous in 10 dogs. In 5 dogs the location was mixed. Entire dogs were over‐represented for venous thrombosis (P=0.003).

Compared to controls, TD had higher monocytes (P=0.008) and neutrophils (p<0.001), and lower eosinophils (P<0.001), potassium (P=0.018), total calcium (P=0.003), and albumin (P=0.003). Eosinophil count and potassium concentration were independently associated with TD in the multivariate analysis.

This study identifies several parameters that may help identify dogs with PLN at increased risk of TD, and occurrence of TD subtypes.

**Disclosures**

Disclosures to report, please report below

The authors have not received funding that is directly related to the material presented in this abstract. However, general support for the feline research group that Harriet Syme is part of, and consultancies and speakers honoraria have been received by Harriet from Royal Canin, Mars Petcare, Hill's, Idexx, Boehringer Ingelheim, Vetoquinol, MSD, Pfizer, Elanco and PetPlan Charitable Trust.

## 43 ESVNU‐O‐14

### 43.1 ESVNU ‐ European Society of Veterinary Nephrology and Urology

#### 43.1.1 Comparison between the glomerular filtration markers symmetric dimethylarginine and creatinine in serum from 458 cats

##### 43.1.1.1 C Tegner ^1^, I Ljungvall ^2^, J Häggström ^2^, J Öberg ^3^, C Bergman , L Pelander ^2^

###### 43.1.1.1.1 ^1^ University Animal Hospital, Swedish University of Agricultural Sciences Uppsala Sweden; ^2^ Swedish University of Agricultural Sciences Uppsala Sweden; ^3^ AniCura Animal Hospital Bagarmossen Sweden

Symmetric dimethylarginine (SDMA) is proposedly a sensitive marker of impaired glomerular filtration rate. The objectives of this study were to investigate associations between serum creatinine and SDMA concentrations measured with Catalyst (C‐SDMA) and IDEXX SDMA (I‐SDMA) in cats with and without kidney disease, and to assess variability of the C‐SDMA assay.

458 client‐owned cats of varying ages and breeds were included. C‐SDMA and I‐SDMA were used to determine SDMA concentrations in surplus serum samples with known creatinine concentrations. The agreement between assays was assessed and coefficient of variability (CV) for C‐SDMA was calculated based on results of ten successive runs on fresh serum from azotemic (n=5) and non‐azotemic (n=4) cats.

Comparing creatinine to C‐SDMA (n=458), 64% of samples had a result within reference range for both markers, 13% increased in both, 16% increased creatinine only and 7% increased SDMA only. Comparing creatinine to I‐SDMA (n=374), 58% of samples had a result within reference range for both markers, 21% increased in both, 9% increased creatinine only and 12% increased SDMA only. Results of C‐ and I‐SDMA assays correlated, but the 95%‐limit of agreement was ‐6 – 11 μg/dL (SDMA reference interval 0 – 14 μg/dL) and mean bias was 2.6. Median CV for C‐SDMA was 11.9% (range 9.48% – 19.86%).

SDMA concentrations within reference range were found in a clinically relevant proportion of azotemic cats. Agreement between the two SDMA assays was poor and variability for C‐SDMA high. Caution should be taken when interpreting single SDMA results in individual cats.

**Disclosures**

No disclosures to report

## 44 ESVNU‐O‐15

### 44.1 ESVNU ‐ European Society of Veterinary Nephrology and Urology

#### 44.1.1 Urinary protein: creatinine ratio and urine specific gravity in cats: cystocentesis versus home sampling

##### 44.1.1.1 F. Mortier, S. Daminet, L. Duchateau, D. Paepe

###### 44.1.1.1.1 Ghent University Merelbeke Belgium

The urinary protein: creatinine ratio (UPC) and urine specific gravity (USG) are essential for the diagnosis and monitoring of feline chronic kidney disease. Urine collection by cystocentesis in the clinic is not always possible, but data comparing cystocentesis with home sampling are lacking in cats.

The aims of this prospective study were to compare UPC and USG between cystocentesis samples versus urine collected at home, and to detect clinically relevant changes in UPC (i.e. a different proteinuria substage according to the International Renal Interest Society) or USG.

Owners of 92 cats (43 healthy, 49 diseased) collected urine at home and within 1‐15 hours, cystocentesis was performed in the clinic.

Of 165 attempts to collect urine at home, 92 (56%) were successful. With cystocentesis, median (range) values for UPC and USG were 0.24 (0.06‐5.08) and 1.036 (1.008‐1.060) respectively. With home sampling, these values were 0.19 (0.00‐4.01) and 1.043 (1.007‐1.064). A substantial agreement existed between sampling techniques for UPC (weighted к 0.68) and USG (weighted к 0.64) categories. Proteinuria (UPC >0.4) was observed in 25% of cystocentesis and 14% of voided samples. Suboptimal urine concentration (USG <1.035) was present in 46% of cats based on cystocentesis samples, versus 32% of cats when examining voided urine.

When feasible, home sampling of feline urine is a valid alternative to cystocentesis for UPC and USG determination. Since clinically relevant differences in UPC and USG each are present in >10% of cats, the same collection method should be used to monitor the same cat.

**Disclosures**

Disclosures to report, please report below

This study is part of a PhD project that is financially supported by IDEXX Laboratories Inc.

## 45 ESCG‐O‐1

### 45.1 ESCG ‐ European Society of Comparative Gastroenterology

#### 45.1.1 Dogs with chronic enteropathy have increased intestinal permeability

##### 45.1.1.1 P. Ishii ^1^, R. Pilla ^1^, A. Pereira ^2^, F. Teixeira ^2^, J. Steiner ^1^, J. Lidbury ^1^, D. Ricardo ^3^, J.S. Suchodolski ^1^

###### 45.1.1.1.1 ^1^ Gastrointestinal Laboratory College Station United States; ^2^School of Veterinary Medicine and Animal Science of University of Sao Paulo Sao Paulo Brazil; ^3^ Faculdade Metropolitana Unidas Sao Paulo Brazil

Increased intestinal permeability (IP) has been hypothesized to play a role in the pathogenesis of chronic enteropathy (CE) in dogs. CE can be subdivided into four major subgroups based on treatment response: food‐responsive (FRE), immunosuppressive‐responsive (IRE), antibiotic‐responsive (ARE), and non‐responsive (NRE). Recently, iohexol has been described as an IP marker in healthy dogs. The purpose of this study was to assess IP in dogs with CE.

Forty‐six dogs were enrolled for this study: 25 healthy control dogs (HC) and 21 dogs with CE. Clinical activity scoring was obtained using the canine chronic enteropathy activity index (CCECAI). A decrease of 50% of the CCECAI index was considered as positive response to treatment. From the 21 dogs with CE, 12 dogs (57%) were classified FRE and 9 dogs (43%) as IRE. All dogs with CE were first prescribed a 14‐day diet trial with a commercially available hydrolyzed protein diet before further workup. Iohexol testing was performed on day 0 for all HC dogs and dogs with CE. In addition, iohexol follow‐up testing was done on day 14 for 17 of the 21 dogs with CE. Dogs were fasted for 12 hours before oral administration of 2.0 ml/kg iohexol (Omnipaque‐350, GE Healthcare). Blood samples were collected from the jugular vein 2 hours after iohexol administration, centrifuged, and frozen. Serum iohexol concentrations were measured by a commercially available enzyme‐linked immunoassay (ELISA).

Dogs with CE had significantly higher serum iohexol concentrations (median: 31.20 μg/mL; range: 18.9‐126.9) than HC dogs (median: 23.7 μg/mL; range: 13.5‐56.7; p = 0.0144). In the 17 dogs with CE that had a follow‐up iohexol permeability testing, there was a significant decrease in serum iohexol concentrations after the 14‐day diet trial (p = 0.0113). In dogs with FRE (n = 10) with a follow‐up iohexol permeability test, there was a significant decrease in serum iohexol concentrations after the diet trial (p = 0.019). In contrast, there was no significant difference in serum iohexol concentrations in dogs with IRE (n = 7) after the diet trial (p = 0.296). This study suggests that dogs with CE have increased IP when compared to HC dogs and that a hydrolyzed protein diet leads to a significant decrease of IP in dogs with FRE, but not IRE. This finding reinforces the need for further studies to clarify the role of increased IP in the pathogenesis of CE in dogs.

**Disclosures**

No disclosures to report

## 46 ESCG‐O‐2

### 46.1 ESCG ‐ European Society of Comparative Gastroenterology

#### 46.1.1 Evaluation of intestinal permeability, NetF toxin genes, sepsis and albumin in dogs with acute haemorrhagic diarrhoea syndrome

##### 46.1.1.1 A. Reisinger ^1^, H Stübing ^2^, J Suchodolski ^3^, P Ishii ^3^, S Unterer ^4^, J Steiner ^3^, J Lidbury ^3^, K Hartmann ^2^, K Busch‐Hahn ^2^

###### 46.1.1.1.1 ^1^ Ludwig Maximilians University of Munich Munich Germany; ^2^ Clinic of Small Animal Medicine, Ludwig Maximilians University Munich Germany; ^3^ Gastrointestinal Laboratory, Department of Small Animal Clinical Sciences College Station United States; ^4^ Clinic for Small Animal Internal Medicine Zurich Switzerland

Intestinal histopathology in dogs with acute haemorrhagic diarrhoea syndrome (AHDS) shows necrotizing enteritis. It has been proposed that cytotoxic NetF toxin of *Clostridium perfringens* is responsible for the intestinal lesions. The study aim was to evaluate the association between intestinal permeability as assessed by serum iohexol, the prevalence of *netF*‐genes, septic criteria and serum albumin concentration. Thirty client‐owned dogs with AHDS were included in this prospective study. At day of presentation, dogs were administered 2 ml/kg body weights of iohexol orally and serum was obtained two hours later to measure iohexol concentration (SIC) by enzyme‐linked immunosorbent assay. Faeces from twenty‐five dogs were collected daily during hospitalization and afterwards at day 7, 21, 42 for evaluation of *netF*‐gene carrying *C perfringens (netF)* via quantitative PCR. Criteria by Hauptman et al. (PMID: 9381665) were used to define septic patients. Mann‐Whitney‐tests were used to compare SIC between *netF* positive and negative dogs and between septic and non‐septic dogs. No significant (p=0.37) difference in SIC was found between dogs positive (n=14; median: 71 μg/ml; min‐max: 9 to 246) or negative for *netF* (n = 11; 44 μg/ml; 16 to 165). A total of 14 of 25 (56%) dogs with AHDS harboured *netF* at presentation, but only three of them remained positive on day 7. After day 21 all dogs were *netF* negative. There was no significant difference (p=0.056) between SIC of dogs with sepsis (median: 134 μg/ml; min‐max: 9 to 246) and dogs without (42 μg/ml; 21 to 116). But a ROC curve with 47% sensitivity (95% CI: 25% to 70%) and 100% specificity showed that a cut off value of over 136 μg/ml was associated with septic criteria. Moreover, there was a significant negative correlation between SIC and serum albumin concentrations (r_s_ = ‐ 0.7; p=0.0004). *NetF* is associated with AHDS but decrease rapidly. Based on SIC values dogs with AHDS have increased intestinal permeability, which correlates negatively with serum albumin concentrations. Furthermore, increased SIC values are associated with the development of sepsis.

**Disclosures**

No disclosures to report

## 47 ESCG‐O‐3

### 47.1 ESCG ‐ European Society of Comparative Gastroenterology

#### 47.1.1 Serum transcobalamin analysis in dogs – method validation and association with serum cobalamin status in dogs with chronic enteropathy

##### 47.1.1.1 S. Kather ^1^, F. Reich ^1^, E. Nexo ^2^, G. Köller ^1^, N. Grützner ^3^, H. Pfannkuche ^1^, F. Dengler ^4^, G. Gäbel ^1^, R.M. Heilmann

###### 47.1.1.1.1 ^1^ University of Leipzig College of Veterinary Medicine Leipzig Germany; ^2^ Aarhus University Hospital Aarhus Denmark; ^3^ VetaRegio Köthen Germany; ^4^ University of Veterinary Medicine Vienna Vienna Austria

Assessment of the cobalamin (vitamin B_12_) status in small animal medicine is performed if a chronic enteropathy (CE) is suspected and is based on measuring serum cobalamin concentration. However, this does not necessarily reflect the intracellular availability of cobalamin. In the blood, cobalamin is bound to transcobalamin (TC), a protein needed to transport cobalamin into the cells. TC also increases in inflammatory conditions in humans, particularly those involving macrophage activation. So far, it has not been possible to measure TC in canine samples. This study aimed to establish an in‐house enzyme‐linked immunosorbent assay (ELISA) to quantify total (unsatured + saturated) TC in canine serum and evaluate serum TC concentrations in dogs with CE in relation to the serum cobalamin status.

A sandwich‐ELISA was established using left‐over canine sera and the gammaglobulin fraction of a rabbit polyclonal anti‐recombinant human TC as both capturing (insolubilized on ELISA plates) and detecting (after biotinylation) reagent. Serially diluted human serum served as calibrator. The assay was analytically validated by determining the lower detection limit, dilutional linearity, spiking recovery, and intra‐ and inter‐assay variability. Serum total TC concentrations were measured in 53 dogs with CE and compared among the subgroups with different serum cobalamin levels.

The lower detection limit of the ELISA was 80 aU/L. Mean observed‐to‐expected ratios for serial dilutions ranged from 77.2−121.6% for 10 serum samples, and for spiking recovery ranged from 73.3−120.7% (mean±SD: 95.6±14.8%) for 10 different spiked serum samples. Intra‐ and inter‐assay coefficients of variation for 6 different serum samples were 1.9−16.2% and 4.4−19.8%, respectively. Serum TC concentrations were significantly higher in CE dogs with suboptimal serum cobalamin status (185–290 pmol/L, n=15; median TC: 421 aU/L) than in hypocobalaminemic (<180 pmol/L, n=18; median TC: 296 aU/L, *P*=0.0151) or normo‐/hypercobalaminemic dogs with CE (≥296 pmol/L, n=20; median TC: 97 aU/L, *P*=0.0039).

Our TC‐ELISA being linear, accurate, precise, reproducible, and sufficiently analytically sensitive, appears suitable for measuring TC in canine samples. Alterations of TC detected in dogs with CE according to serum cobalamin levels might reflect (i) efforts to compensate an increased demand for cobalamin, eventually leading to depletion, or (ii) autoantibodies against TC interfering with cobalamin and also ELISA‐antibody binding. Future studies are needed to optimize this assay, establish a reference interval, and explore its utility for diagnosing cobalamin deficiency. In humans, paired measurement of the cobalamin‐saturated TC portion (holoTC) and cobalamin is increasingly used to diagnose cobalamin deficiency.

**Disclosures**

No disclosures to report

N/A

## 48 ESCG‐O‐4

### 48.1 ESCG ‐ European Society of Comparative Gastroenterology

#### 48.1.1 Hypocobalaminemia in dogs with parvovirus enteritis

##### 48.1.1.1 B. Blatt, H. Plickert, I. Schwendenwein, A. Tichy, R. Marculescu, I. Burgener, N. Luckschander‐Zeller

###### 48.1.1.1.1 Vienna Austria

Enterocyte damage by intracellular parvovirus replication might impair cobalamin uptake causing hypocobalaminemia. It was hypothesized that dogs with parvovirus enteritis (PV) have lower serum cobalamin concentrations (CBL) compared to age‐matched healthy controls (HC) and that cobalamin status correlates with outcome and clinical pathology data.

Thirty client‐owned hospitalized dogs with naturally acquired PV and thirty HC were included in this prospective observational study. Complete blood count (CBC), blood chemistry and CBL were performed at presentation. For statistical analysis the graph pad prism statistic program was used. P <0.05 was considered as statistically significant.

Significant differences in CBL were found between HC (mean 824+/‐195pg/ml) and PV (mean 431+/‐271pg/ml; p<0.0001); survivors (S; N=19; mean 520+/‐290pg/ml) and non‐survivors (NS; N=11; mean 277+/‐143pg/ml; p=0.015). In the S group significant correlations were detected between CBL and white blood cell count (p<0.0001; r=0.66), neutrophils (p<0.0001; r=0.55), and platelets (p<0.0001; r=0.83). In the NS group significant correlations were detected between CBL and hematocrit (p=0.05; r=0.6).

The significant differences in CBL between HC and PV as well as S and NS suggest that young dogs with PV are prone to hypocobalaminemia. The correlations between CBL and CBC‐data might offer information regarding disease severity. Therefore, addressing hypocobalaminemia in canine PV may yield an additional therapeutic target.

**Disclosures**

Disclosures to report, please report below

Sponsored by Vet‐Concept GmbH & Co. KG

## 49 ESCG‐O‐5

### 49.1 ESCG ‐ European Society of Comparative Gastroenterology

#### 49.1.1 Investigation of the relationship of mucosa‐associated bacteria to clinical and histological findings in dogs with protein losing enteropathy (PLE)

##### 49.1.1.1 F. Lotti ^1^, S Priestnall ^2^, K Simpson ^3^, F. Procoli ^1^

###### 49.1.1.1.1 ^1^ Ospedale Veterinario "I Portoni Rossi" ‐ Anicura Zola Predosa (Bologna) Italy; ^2^ Royal Veterinary College Hatfield, Hertfordshire United Kingdom; ^3^ Cornell University Ithaca, NY United States

Canine idiopathic protein losing enteropathy (PLE) is a syndrome characterized by excessive loss of protein across the intestinal tract. Common intestinal histopathological findings in dogs with PLE include abnormal morphology of villi, lacteals and crypts and proprial neutrophilic inflammation. The latter has been attributed to transmucosal bacterial translocation.

The purpose of this study was to evaluate the relationship of mucosa‐associated bacteria to clinical and histological findings in dogs with PLE.

Dogs with PLE and small intestinal biopsies evaluated between 2018 and 2020 were included in the study. PLE was defined as presence of hypoalbuminemia (serum albumin <2.5 g/dl) after exclusion of non‐gastrointestinal causes. Histopathological review and scoring was performed by a board certified pathologist according to a simplified version of the WSAVA scoring system. The presence and spatial distribution of mucosal bacteria in small intestinal biopsies was determined by eubacterial Fluorescence in situ hybridization (FISH). Whether antimicrobials had been received prior to presentation was recorded but antimicrobial treatment was not considered an exclusion criteria.

Twenty dogs were eligible for the study. Median CCECAI and serum albumin at presentation were 7.5 (range 2‐12) and 1.55 (range: 0,9‐2,4 g/dl) respectively. Ten dogs had received antimicrobials recently prior to presentation. Duodenal biopsies were collected in 18/20 and ileal in 4/20 cases. Villous stunting, crypt dilation and lymphangiectasia with a WSAVA score ≥ 2 was present in 9/18, 8/18, and 10/18 duodenal samples and 1/4, 1/4 and 2/4 ileal samples respectively. Mild‐to‐moderate neutrophilic infiltration was present in 6/18 duodenal but 0/4 of the ileal biopsies. Mucosa‐associated bacteria were present in 3/18 duodenal (17%) and 0/4 ileal biopsies. In all 3 cases, bacteria were adherent to the epithelial mucus but were not visualized within the mucosa. No left shift was found on CBC in these 3 dogs. All FISH positive cases had significant villous stunting, crypt dilation, with mild neutrophilic inflammation in 2/3. Two out of three FISH positive cases had been receiving antibiotics at the time of intestinal biopsies.

In conclusion, we found that mucosa‐associated bacteria were infrequent in a cohort of dogs with PLE. We found no evidence of mucosally invasive bacteria. Two out of three FISH positive cases already received antimicrobials, possibly indicating antibiotic resistant strains. Further study in a larger cohort of dogs that is controlled for antimicrobial therapy is required to fully evaluate the role of mucosa‐associated bacteria in canine PLE.

**Disclosures**

Disclosures to report, please report below

This study was funded by Anicura Research Fund

## 50 ESCG‐O‐6

### 50.1 ESCG ‐ European Society of Comparative Gastroenterology

#### 50.1.1 A survey‐based observational study on diagnostic and therapeutic approach to canine idiopathic protein‐losing enteropathy: general practitioners’ versus gastroenterologists’ point of view

##### 50.1.1.1 F. Lotti, F. Procoli

###### 50.1.1.1.1 Ospedale Veterinario "I Portoni Rossi" ‐ Anicura Zola Predosa (Bologna) Italy

Canine idiopathic protein losing enteropathy (PLE) is a syndrome characterized by an excessive loss of protein across the intestinal tract due to different causes. Probably owing to the lack of prospective studies regarding treatment, and/or lack of agreement on ideal treatment strategies, the approach to dogs with PLE varies across small animal practitioners and gastroenterologists alike.

The aim of this observational study was to compare how general practitioners (GPs) and gastroenterologists (GEs) diagnostically and therapeutically approach idiopathic canine PLE.

An online questionnaire made of 32 questions was sent to 2 groups of small animal veterinarians: those working in clinics belonging to the same corporate represented across Northern and Western Europe (GPs), and members of two gastroenterology‐only interest societies (GEs). Descriptive analysis of results was used; chi square test was used to compare frequency of answers between groups.

A total of 118 questionnaires were returned: 77 from GPs and 41 from GEs overall from 20 different countries. When presented with a dog with PLE, the majority of respondents across groups (59/77, 77% GPs vs 34/41, 83 % GEs) proceeded with GI endoscopy before defining a treatment protocol. Prior to performing endoscopy, significantly more GEs requested an abdominal ultrasound (68/77 GPs vs 41/41 GEs; p=0,02). Significantly more GEs attempted to rule out atypical hypoadrenocorticism by assessing basal cortisol and/or performing ACTH stimulation test (34/41 vs 49/77; p=0.04). Regarding treatment, significantly more GEs preferred sequential approach starting with dietary trial first (77% vs 55%; p=0,04). Regarding diet choice, 52% GEs vs 15% GPs awaited the histological findings before selecting between a low‐fat or hydrolysed diet. Overall, 32% of GEs and 30% of GPs prescribed a low‐fat diet as first choice. Twenty‐three percent of GEs vs 45% of GPs opted to start treatment with a multimodal approach made of diet together with a combination of 1 or 2 immunosuppressants, and/or antibiotics. Overall, significantly more GPs prescribed antibiotics as part of the treatment of PLE (66% vs 37%; p=0,03). Lastly, significantly more GEs (78%) routinely prescribes anti‐platelets drugs vs 51% of GPs (p=0,008).

In conclusion, this study showed significant differences on the diagnostic and therapeutic approach to idiopathic canine PLE between GPs and GEs. Some of these differences may have an impact on clinical response and patient survival. This study further highlights the need of treatment‐based randomized controlled studies and the definition of clearer unanimous recommendations regarding the diagnostic approach to canine PLE.

**Disclosures**

No disclosures to report

## 51 ESCG‐O‐7

### 51.1 ESCG ‐ European Society of Comparative Gastroenterology

#### 51.1.1 The impact of diet reassessment in the medical management of Refractory Chronic Enteropathy in dogs‐ A retrospective multicentric study

##### 51.1.1.1 N. Santos ^1^, S. Duvergé ^2^, M.J. Dias ^3^, M. Hebert ^4^, E. Bettin ^5^, F. Signorelli ^5^, F. Procoli ^5^, J. Hernandez ^4^, R. Leal ^3^

###### 51.1.1.1.1 ^1^ Veterinary Teaching Hospital, Fac. Vet. Med., ULisboa Lisbon Portugal; ^2^ Veterinary Teaching Hospital ‐ Faculty of Veterinary Medicine ; ULisboa Lisbon Portugal; ^3^ Centre for Interdisciplinary Research in Animal Health, Fac.Vet.Med., ULisboa Lisbon Portugal; ^4^ Oniris, Ecole Nationale Vétérinaire Agroalimentaire et de l'Alimentation Nantes France; ^5^ Anicura Ospedale Veterinario Portoni Rossi, Bologna Italy

The role of further dietary trials in the therapeutic management of canine refractory chronic enteropathy (RCE) is called into question since often hydrolysed diets have already been tried without clinical improvement.

The aim of this study is to assess the impact of further dietary change in dogs with RCE that have already been fed a hydrolysed diet.

Medical records of dogs presented with chronic diarrhoea (> 3 weeks duration) as a referral or second opinion consultation between April 2018 and December 2021, in three referral centres, were reviewed. Cases with RCE (defined as persistent diarrhoea after exclusion of extra‐digestive and infectious causes and despite previous unsuccessful trials with a combination of hydrolysed diet, probiotics ± antibiotics, and immunesuppressants) were selected. Cases were further characterised based on dietary choice and clinical response over time.

In a total of 142 dogs with chronic diarrhoea, 26/142 (18.3%) were considered RCE. Of them, 15/26 (57.7%) had a histologic diagnosis of chronic lympho‐plasmacytic enteritis while in 11/26 (42.3%) histopathology was not available.

Among RCE dogs, 23/26 (88.5%) were transitioned onto another alternative diet, either a different commercial hydrolysed diet, commercial novel protein source diet, novel protein source home‐made diet, commercial prebiotic‐rich/hyperdigestible specific diet or commercial hyperdigestible non‐hydrolysed diet.

Dietary change was exclusively performed in 16/23 (69.4%): 11/16 (68.8%) switched for another hydrolysed diet, 2/16 (12.5%) for a home‐made diet, 1/16 (6.3%) for a prebiotic‐rich/hyperdigestible specific diet, 1/16 (6.3%) for a commercial novel protein source diet and 1/16 (6.3%) for a commercial hyperdigestible non‐hydrolysed diet. From these, 11/16 (68.8%) showed clinical improvement: eight with hydrolysed diet, one with home‐made diet, one with prebiotic‐rich/hyperdigestible specific diet and one with a commercial novel protein source diet. The remaining 5/16 (31.3%) did not improve with dietary change.

Association of medical treatment was made concurrently with dietary change in 7/23 (30.4%). Apart from medical adjustment, 5/7 (71.4%) transitioned onto another hydrolysed diet while 2/7 (28.6%) onto a home‐made diet. Clinical improvement was observed in 3/7 (42.9%), two fed a hydrolysed vs one a home‐made diet. The remaining 4 dogs continued refractory to treatment.

Dietary reassessment was frequent in dogs with RCE, accounting for clinical improvement not only in cases in which concurrent treatments were simultaneously adjusted but also in most of those to whom diet was the single therapeutic measure. This study highlights that a novel dietary trial should be considered before declaring a canine chronic enteropathy refractory to treatment.

**Disclosures**

Disclosures to report, please report below

No conflict of interest to declare. This work was supported by FCT – Fundação para a Ciência e Tecnologia IP, grant UIDB/00276/2020.

## 52 ESCG‐O‐8

### 52.1 ESCG ‐ European Society of Comparative Gastroenterology

#### 52.1.1 The effect of metronidazole versus a synbiotic on selected intestinal bacteria and clinical improvement in dogs with acute diarrhoea

##### 52.1.1.1 H. Stübing ^1^, A. Reisinger ^2^, J. Suchodolski ^3^, M. Werner ^4^, A. Ziese ^5^, S. Unterer ^4^, J. Steiner ^3^, J. Lidbury ^3^, K. Hartmann ^6^, K. Busch‐Hahn ^2^

###### 52.1.1.1.1 ^1^ Ludwig Maximilian University Munich Germany; ^2^ Clinic of Small Animal Internal Medicine, Ludwig Maximilian University Munich Germany; ^3^ Gastrointestinal Laboratory, Department of Small Animal Clinical Sciences, Texas College Station United States; ^4^ Clinic for Small Animal Internal Medicine, Vetsuisse Faculty Zurich Switzerland; ^5^ Clinic of Small Animal Medicine, Centre for Clinical Veterinary Medicine, LMU Munich Germany; ^6^ Clinic of Small Animal Medicine, Centre for Clinical Veterinary Medicine, LMU Munich Germany

*Escherichia coli* and *Clostridium perfringens* are often mentioned as potential enteropathogens, but their role in acute uncomplicated diarrhoea (AD) of dogs remains controversial. In contrast, *Clostridium hiranonis* plays a major role in maintaining a normal intestinal microbiota due to its bile acid converting function. Metronidazole or synbiotics are frequently used to treat AD in dogs. Thus, the aim of this study was to evaluate effects of these two therapies on clinical improvement and the bacteria mentioned above.

Seventeen dogs with AD of < 5 days duration were included in this prospective, randomized, double‐blinded clinical trial. Dogs were assigned to a metronidazole (METg; n=12; 10‐20 mg/kg PO q12h for 7 days) or synbiotic group (SYNg; n=7; *E. faecium* DSM 10663 NCIMB 10415/4b1707; 10^8^ CFU/kg PO q12h for 7 days). The bacteria *C hiranonis, E coli and C perfringens were analyzed in faeces using qPCR* on day 0, 6 and 30. In all dogs, faecal consistency was assessed using the Purina faecal scoring chart daily for eleven days. Bacteria were analyzed using mixed models with repeated measures. Clinical improvement defined as two consecutive fecal scores ≤ 4 was analyzed using a Welch's t‐test.

There was no significant difference in bacterial abundance between both groups at baseline. *E coli* showed a significant increase in the METg on day 6 (median: 7.5; min‐max: 5.8 to 8.2) vs. SYNg (median: 3.7; min‐max: 1.4 to 8.0; *p=0.003*) and day 30 (METg median: 6.8; min‐max: 2.2 to 8.3; SYNg median: 2.7; min‐max: 1.4 to 7.3; *p=0.003*). No significant difference in *C perfringens* was observed between both treatments during the study. There was a significant decrease of *C hiranonis* in the METg on day 6 (METg median: 0.6; min‐max: 0.1 to 6.4) vs. SYNg (SYNg median: 6.5; min‐max: 5.6 to 6.8; *p<0.0001*) and day 30 (METg median: 6.2; min‐max: 0.1 to 7.0; SYNg median 6.6; min‐max: 5.9 to 7.0, *p<0.0001*). No significant difference (*p=0.1)* was observed in days until resolution of diarrhoea between the METg (mean 2.0; SD 1.0) and SYNg (mean 3.3; SD 2.4).

In this study, metronidazole induced an increase in *E coli* and a decrease in the beneficial bacterium *C hiranonis*, but had no effect on *C perfringens*. Thus, metronidazole in AD has a negative influence on the intestinal microbiome but shows no superiority in shortening the time until resolution of diarrhoea.

**Disclosures**

Disclosures to report, please report below

The doctoral student was financially supported by the company NutraPro to perform this study. We are confident, that this financial support did not have any impact on the results of the study, since it was designed as a prospective blinded treatment trial.

## 53 ESCG‐O‐9

### 53.1 ESCG ‐ European Society of Comparative Gastroenterology

#### 53.1.1 Course of disease in dogs hospitalized for acute pancreatitis – correlation of lipase activity (Roche colorimetric lipase assay, LIPC), pancreatic lipase immunoreactivity (PLI, measured as Spec PL) and clinical variables over time

##### 53.1.1.1 C. Cueni ^1^, N. Hofer‐Inteeworn ^2^, C. Kümmerle‐Fraune ^2^, C. Müller ^2^, P. Kook ^2^

###### 53.1.1.1.1 ^1^ Clinic for Small Animal Internal Medicine Zurich Switzerland; ^2^ Clinic for Small Animal Internal Medicine, Vetsuisse faculty Zurich Switzerland

The course of serum lipase activity and lipase immunoreactivity (PLI) over time in dogs with acute pancreatitis (AP) and correlations of lipases with clinical signs are unknown.

Records were searched for eligible cases presented between 01/2019 and 08/2021. Inclusion criteria were hospitalization for AP, available consecutive measurements of lipase activity and surplus serum for corresponding PLI determinations. Diagnosis of AP was based on compatible clinical signs (lethargy, anorexia, vomiting, abdominal pain, diarrhea) of ≤ 7d duration, and lipase activity >350U/L (RI, 24‐108U/L) at admission (d0).

Like PLI (RI,0‐200μg/L) being reported up to 1500ug/L, lipase activities (Roche colorimetric lipase assay using DGGR as substrate) were truncated at 1500U/L for analyses. Spearman's correlation was used for assessing relationships among both lipases and c‐rP concentration on days 0,1,2, and between both lipases and presence or absence of vomiting, hematemesis, diarrhea, hematochezia, anorexia, and abdominal pain on days 0,1,2.

Thirty‐nine hospitalizations for AP were analyzed. Median (range) duration of clinical signs before admission was 2d (1‐7d). Median (range) lipase activity and PLI at d0 were 1070U/L (357‐1500) and 1111μg/L (292‐1500). Correlation of both lipase assays at d0 was very strong (r_s_ 0.955, p<.0001; n= 39), and remained very strong on d1 (r_s_ 0.96, p<.0001; n=37) and d2 (r_s_ 0.957, p<.0001; n=22). On d1, 13/39 (33%), and 18/39 (46%) cases had normalized (within RI) lipase activities and PLI. Lipase activities of the 5 dogs with PLI<200μg/L on d1 were minimally increased (median 124 U/L; range, 119‐131). On d2, 4 more dogs had completely normalized lipase results (lipase activity and PLI).

Lipases decreased at d1 but increased again in 2 dogs, increased at d1 and decreased again in 2 dogs, and remained markedly increased in 3 dogs. Nature of change was always the same for both lipase assays. None of the recorded clinical signs correlated significantly with lipases at any time. C‐rP correlated only on d1 significantly with lipase activity and PLI (r_s_=0.321, p=.048 and r_s_=.373, p=.027; n=35), but neither on d0 (n=36) nor on d2 (n=19).

Rapid normalization of markedly increased lipase results in dogs with signs compatible with AP is a new finding and can complicate a diagnosis of AP when lipase measurement is delayed. The consistently very high correlation between both lipase assays answers questions about the specificity of the LIPC lipase activity assay. Clinically irrelevant discrepancies are related to the reference intervals. No correlation of clinical signs with lipases could be identified.

**Disclosures**

No disclosures to report

## 54 ESCG‐O‐10

### 54.1 ESCG ‐ European Society of Comparative Gastroenterology

#### 54.1.1 The clinical course and the utility of a clinical severity scoring system and 1,2‐o‐dilauryl‐rac‐glycero glutaric acid‐(6’‐methylresorufin)‐ester‐lipase for monitoring of dogs with chronic pancreatitis

##### 54.1.1.1 S. Kuzi, D Adlersberg, I Aroch, G Segev

###### 54.1.1.1.1 Koret school of veterinary medicine, Hebrew University of Jerusalem Tel Aviv Israel

Chronic pancreatitis (CP) is common in dogs; however, the utility of 1,2‐o‐dilauryl‐rac‐glycero glutaric acid‐(6’‐methylresorufin)‐ester‐(DGGR)‐lipase activity in monitoring CP is unknown.

This study aimed to examine the association between DGGR‐lipase activity and the clinical control of CP, assessed by a CP clinical severity scoring system (CPCSS) during follow‐ups.

This Retrospective study included dogs diagnosed with CP based on clinical signs and DGGR‐lipase activity >250 U/L. Dogs were followed by CPCSS and DGGR‐lipase activity.

The study included 24 dogs (134 visits; median, 10 visits/dog; range, 2‐11). CPCSS (*P* <0.001) and DGGR‐lipase activity (*P* =0.003) were higher in unscheduled (i.e., emergency; n=44; 32.8%) compared with scheduled (i.e., elective; n=90; 67.2%) visits. Based on the CPCSS, mild‐moderate (score 0‐3) and severe (score ≥4) disease were noted in 94 (70%) and 40 (30%) visits, respectively. DGGR‐lipase activity was associated (*P* = 0.009) with CPCSS, with lower activity in mild‐moderate (CPSS 0‐3; median 391 U/L; range 49‐3747), compared to severe CP (CPCSS score ≥4; median, 558 U/L; range 63‐7133). DGGR‐lipase activity significantly and weekly correlated the CPSS (r = 0.233, P = 0.007). DGGR‐lipase activity inefficiently discriminated mild‐moderate vs severe disease (area under the receiver operator characteristics curve, 0.64; 95%CI, 0.53‐0.75; *P* =0.012). A DGGR‐lipase activity cutoff of 428 U/L corresponded to sensitivity of 65% and specificity of 63%.

Increased CPCSS is associated with emergency revisits, warranting increased vigilance. DGGR‐lipase activity is inaccurate in classifying the disease severity, nevertheless, there is an association between DGGR‐lipase activity and the CPCSS over follow‐ups.

**Disclosures**

No disclosures to report

## 55 ESCG‐O‐11

### 55.1 ESCG ‐ European Society of Comparative Gastroenterology

#### 55.1.1 Fecal acute phase proteins as biomarkers in cats with chronic enteropathies

##### 55.1.1.1 D. Karra ^1^, C. Chadwick ^2^, E. Stavroulaki ^1^, M. Pitropaki ^1^, K. Allenspach ^3^, J. Lidbury ^4^, J.M. Steiner ^4^, P. Xenoulis ^1^

###### 55.1.1.1.1 ^1^ University of Thessaly Karditsa Greece; ^2^ Life Diagnostics West Chester United States; ^3^ Iowa State University Ames United States; ^4^ Texas A&M University Austin United States

Chronic enteropathies (CE) are common in cats and their classification is currently based on response to treatment and/or histopathologic evaluation of gastrointestinal (GI) biopsies. Reliable biomarkers that can distinguish different causes and predict or monitor response to treatment in cats with CE are currently lacking. The aim of this study was to evaluate certain acute phase proteins (APPs) in feces that could potentially be used as biomarkers in cats with CE.

Feces from 29 cats with IBD (n=16; 3 were food‐responsive and 13 were steroid‐responsive) or small cell alimentary lymphoma (SCAL) (n=13), diagnosed by means of gastrointestinal endoscopy and histopathological examination of biopsies, were collected before treatment (T0), 30 (T1), and 90 days (T2) after initiation of treatment, and concentrations of haptoglobin, alpha‐1‐acid‐glycoprotein (AGP), pancreatitis associated protein (PAP‐1) and ceruloplasmin were determined using SPARCL™ immunoassays. Clinical activity was assessed using FCEAI (mild to severe decrease in activity and/or appetite and mild to severe diarrhoea, vomiting and/or weight loss). Cats were treated with diet and/or prednisolone (IBD cats), plus chlorambucil (SCAL cats). Thirty healthy cats without any GI signs for at least 6 months were used as control group.

Compared to controls, fecal AGP concentrations were significantly lower (24.7 μg/g vs 3.1 μg/g; p=0.0019) and fecal haptoglobin (0.17 μg/g vs 0.79 μg/g) and PAP‐1 (0.04 μg/g vs 0.42 μg/g) concentrations were significantly higher (p<0.0001) in cats with CE. Fecal AGP concentrations were significantly lower (p=0.0358) in cats with IBD (0.6 μg/g) compared to cats with SCAL (10.2 μg/g). No significant differences were found in fecal concentrations of haptoglobin, PAP‐1 and ceruloplasmin between cats with IBD and SCAL. A significant reduction from T0 to T2 was found only for fecal ceruloplasmin concentrations, (7.6 vs T2: 2.2 μg/g; p=0.0131). A significant correlation was found between fecal PAP‐1 concentration and clinical scoring at T2 (r=0.5438, p=0.0196). No significant correlations were found between the remaining APPs and clinical scoring.

In conclusion, fecal AGP, haptoglobin and PAP‐1 concentrations only show promise to differentiate between cats with CE and healthy cats, whilst fecal AGP concentration shows promise as a biomarker for the differentiation of cats with IBD and cats with SCAL. Finally, fecal ceruloplasmin concentration shows promise as a marker of treatment response in cats with CE. Further studies are needed to further investigate these findings.

**Disclosures**

No disclosures to report

## 56 ESCG‐O‐12

### 56.1 ESCG ‐ European Society of Comparative Gastroenterology

#### 56.1.1 Long‐term follow‐up in a cohort of 97 cats diagnosed with low‐grade intestinal T‐cell lymphoma or lymphoplasmacytic enteritis: clinical, biological and therapeutic features

##### 56.1.1.1 P Remmel ^1^, P Bernard ^2^, V Freiche ^2^

###### 56.1.1.1.1 ^1^ Ecole Nationale Vétérinaire d'Alfort, CHUVA Maisons‐Alfort France; ^2^ Ecole Nationale Vétérinaire d'Alfort Maisons‐Alfort France

Recent data reporting long‐term follow‐up and prognostic factors in feline lymphoplasmacytic enteritis (LPE) and low‐grade intestinal T‐cell lymphoma (LGITL) are lacking. Differentiation of these entities remains challenging in elderly cats.

The objectives were 1) to report follow‐up and identify prognostic indicators 2) to describe epidemiological and biological features in cats diagnosed with LPE or LGITL according to recently published histological and immunohistochemical criteria.

Ninety‐seven cats (55 LGITL and 42 LPE cases) were retrospectively reviewed (2015‐2022). Kaplan‐Meier analysis with log‐rank testing was used to estimate median survival times (MST) and the association between reduced survival time and selected variables.

Among LPE cases, 5 were lost to follow‐up, 16 died and 21 were alive. Among LGITL cases, 7 were lost to follow‐up, 29 died and 19 were alive. LPE cats had a longer MST than LGITL (1,818 and 599 days respectively, p=0.029). Among LGITL cases, mild hypoalbuminemia, previous administration of glucocorticoids and lack of improvement over three months of treatment were significantly associated with a decreased survival time (p=0.01, p=0.019 and p=0.049, respectively). Choice of chemotherapeutic protocol did not affect MST. Longer clinical signs duration and hypocobalaminemia were associated with LGITL (p<0.001 and 0.011, respectively). Oral and parenteral cobalamin supplementation were effective in most cases. Finally, 3 LGITL cats had histological follow‐up that showed no persistent neoplastic lymphocytes.

Clinical signs duration and hypocobalaminemia are important criteria to differentiate LGITL from LPE cases. Hypoalbuminemia, previous glucocorticoids administration and lack of improvement at initial follow‐up seem to represent negative prognostic factors in LGITL cats.

**Disclosures**

No disclosures to report

## 57 ESVIM‐O‐1

### 57.1 ESVIM ‐ European Society of Veterinary Internal Medicine

#### 57.1.1 Metabolomic approaches highlight biochemical pathways altered in hypertensive cats

##### 57.1.1.1 F. Scott‐Baumann ^1^, A. Watson ^2^, M. Beckmann ^1^, A.J. Mur ^1^, H. Syme ^2^

###### 57.1.1.1.1 ^1^ Aberystwyth University Aberystwyth United Kingdom; ^2^ Royal Veterinary College Hatfield United Kingdom

Early diagnosis and management of spontaneous hypertension remains a significant problem in cats, with multiple potential sequelae including blindness. With a lack of routine blood pressure screening in primary care practice and the potential for situational hypertension a confirmatory test for hypertension could prove useful. Here we use untargeted metabolomics to identify biochemical changes in cats with, or under treatment for, hypertension. Knowledge of these could help to further understanding of the pathogenesis of hypertension in cats.

Biobanked surplus urine samples were selected from cats (>9 years old) that were normotensive (n=19), or hypertensive (n=13). Samples were also obtained following amlodipine treatment of hypertensive cats (n=12). Samples were profiled using flow infusion electrospray ‐ high‐resolution mass spectrometry (FIE‐HRMS) and differences assessed using univariate (ANOVA, t‐test) and multivariate (Principal Component Analysis, Partial Least Squares Discriminant Analysis). Metabolites were tentatively identified using the Kyoto Encyclopaedia of Genes and Genomes (KEGG) database and linked to biological pathways using MetaboAnalyst.

Significant (*P* < 0.005) biochemical differences were observed between each of the sample groups. Most significantly altered metabolites belonged to the classes of peptides and glycosyl compounds. A key biochemical pathway change between treated and untreated hypertensives included taurine and hypotaurine metabolism. Between the hypertensives and normotensives both diacylglycerols, which can be involved in cell signalling, and some energy generating pathways were significantly altered.

Metabolic changes in feline hypertensives appear similar to those reported in other species. It may be possible to identify biomarkers to assist with hypertension diagnosis, monitoring and response to treatment.

**Disclosures**

Disclosures to report, please report below

Statement of disclosures: Scott‐Baumann: I have not received funding that is directly related to the material presented in this abstract. However my current PhD, into bovine tuberculosis in badgers, is funded by a Knowledge Economy Skills Scholarship (KESS 2), NFU Cymru and Protem services (industrial sponsor). Watson: I am receiving funding for my PhD from the BBSRC and Boehringer Ingelheim (my industrial sponsor). Mur: I have not received funding that is directly related to the material presented in this abstract. However, general support for the metabolomic research group that I am part of is funded from the BBSRC, NERC, EU, FAPESP (Brazil) and the KESS2 programme. Beckmann: I have not received funding that is directly related to the material presented in this abstract. However, general support for the metabolomic research group that I am part of is funded from the BBSRC, MRC, EUand the KESS2 programme. Syme: I have not received funding that is directly related to the material presented in this abstract. However, general support for the feline research group that I am part of, and consultancies and speakers honoraria have been received from Royal Canin, Mars Petcare, Hill's, Idexx, Boehringer Ingelheim, Vetoquinol, MSD, Pfizer, Elanco and PetPlan Charitable Trust.

## 58 ESVIM‐O‐2

### 58.1 ESVIM ‐ European Society of Veterinary Internal Medicine

#### 58.1.1 Trapped Neutrophil Syndrome in Border Collies: Clinical Presentation, Diagnostic Findings, Treatment and Outcome in 10 dogs within the UK and USA

##### 58.1.1.1 A Suciu ^1^, D Starybrat ^2^, E Llewellyn ^3^, C Gil‐Morales ^4^, H Matson ^5^, R Jepson ^6^, M Williams ^7^, M Lyraki ^4^, L McMahon ^8^

###### 58.1.1.1.1 ^1^ Midlothian United Kingdom; ^2^ Hospital for Small Animals, The Royal (Dick) School of Veterinary Studies, Easter Bush Campus, Midlothian United Kingdom; ^3^ Hospital for Small Animals, The Royal (Dick) School of Veterinary Studies Easter Bush Campus, Midlothian United Kingdom; ^4^ School of Veterinary Sciences, University of Bristol Bristol United Kingdom; ^5^ Department of Clinical Science and Services, Royal Veterinary College, Hertfordshire United Kingdom; ^6^ Department of Clinical Science and Services, Royal Veterinary College, Hertfordshire United Kingdom; ^7^ Department of Clinical Sciences, Colorado State University Fort Collins, Colorado United States; ^8^ Anderson Moores Veterinary Specialists, The Granary, Bunstead Barns, Poles Lane Hursley, Winchester, Hampshire United Kingdom

Trapped neutrophil syndrome (TNS) is an inherited autosomal recessive neutropaenia that affects Border Collie and Border Collie cross breed dogs. The condition is rarely reported in the literature. Currently the optimal management for these dogs has not been determined and little is known about their prognosis.

The aims of this study were to evaluate the clinical signs, diagnostic findings, treatment administered and short (survival to 28 days) and long term prognosis (survival > 6 months) for these dogs.

This was a retrospective, multicentre, descriptive study. Study inclusion criteria was TNS diagnosis following documentation of a homozygous mutant phenotype with DNA testing. Ten dogs (8 Border Collies, 2 Border Collie cross breeds) were included in the study. The median age at diagnosis was 4.25 months (range 0.75 to 8 months). The most common clinical signs at time of diagnosis were pyrexia (100% dogs), lameness or abnormal gait (50%), gastro‐intestinal signs (50%), underdevelopment (40%) and lethargy (30%). One dog presented with an upper respiratory tract obstruction. The most common concurrent condition was metaphyseal osteopathy (70%). Five (50%) dogs had arthrocentesis performed and all were consistent with immune‐mediated polyarthritis. Median initial segmented neutrophil count was 2.27 x 10^9^/L (range 0.96 ‐ 22.41 x 10^9^/L). Five dogs had a segmented neutrophil count below, four dogs within and one dog above the analyser reference interval at presentation. All dogs received at least one antimicrobial agent within the first 28 days following diagnosis, with amoxicillin‐clavulanic acid being the most commonly administered (80%) and median course length being 9 days (range 4‐28 days). Eight (80%) dogs received immunosuppressant treatment with prednisone or prednisolone (median starting dose 1mg/kg/day; range 0.5‐2.5mg/kg/day). Glucocorticoids were started following antimicrobial treatment in 7/8 (88%) dogs. Follow‐up blood samples were available for 3/4 (75%) dogs who were alive at 6 months. Median segmented neutrophil counts for each of these dogs were 1.2 x 10^9^/L (range 0.47 – 3.43 x 10^9^/L), 4.272 x 10^9^/L (range 1.369 ‐ 22.41 x 10^9^/L), 2.7 x 10^9^/L (range 0.2 ‐ 4.6 x 10^9^/L). Seven (70%) dogs were alive at 28 days and four (40%) dogs were alive at both 6 and 12 months post diagnosis.

Our results suggest pyrexia and musculoskeletal abnormalities are the most common clinical examination findings and neutrophil counts may be low, normal or increased in dogs with TNS. Antibiotics and glucocorticoid medications were commonly used as treatment, with survival past 1 year possible.

**Disclosures**

No disclosures to report

## 59 ESVIM‐O‐3

### 59.1 ESVIM ‐ European Society of Veterinary Internal Medicine

#### 59.1.1 Outcome and predictive factors for response to treatment of idiopathic immune‐mediated polyarthritis in dogs: A retrospective study

##### 59.1.1.1 D. Pichard ^1^, T Robin ^2^, K Le Boedec ^2^, C Maurey ^1^, M Kurtz ^1^, F Da Riz ^1^, S Blot ^1^, M Canonne ^1^, M Cervone ^3^, E Krafft ^4^, JL Cadoré ^4^, L Desquilbet ^5^, G Benchekroun ^1^

###### 59.1.1.1.1 ^1^ Ecole Nationale Vétérinaire d'Alfort Maisons‐Alfort France; ^2^ Centre Hospitalier Vétérinaire Fregis Arcueil France; ^3^ Clinique vétérinaire Evolia L'Isle‐Adam France; ^4^ Université de Lyon, VetAgro Sup, campus vétérinaire Marcy‐l'Etoile France; ^5^ Ecole Nationale Vétérinaire d'Alfort, IMRB Maisons‐Alfort France

Immune‐mediated polyarthritis (IMPA) is one of the most common joint disease in dogs. While its prognosis is usually considered as good, relapses are common and predictive factor of remission and recurrence are currently lacking. The aims of our study were to assess the outcome after medical management of IMPA in dogs, and to identify predictive factors for remission and relapse.

Dogs diagnosed with IMPA between 2010 and 2022 among four veterinary referral centers were retrospectively included. Signalment, clinical presentation, imaging and clinicopathological findings, and treatments were recorded. Based on clinical findings and serum C‐reactive protein (CRP) concentration during follow‐up, remission was defined as partial (improvement but without resolution of clinical signs and/or persistently increased serum CRP concentration) or complete (complete resolution of clinical signs and normal serum CRP concentration). Relapse was defined as recurrence of clinical signs and/or increase in serum CRP concentration, after a remission. Continuous data were presented as median [interquartile range]. To identify exposures at diagnosis associated with remission and relapse, survival analyses were performed using Kaplan‐Meier curves (and log‐rank test) and univariate Cox models. Significance level was set at p=0.05.

Overall, 119 dogs of various breeds were included. The median age at diagnosis was 4.8 years [3‐7]. Non‐erosive IMPA was the most common diagnosis (93%). One hundred and fourteen dogs (96%) achieved partial (32/114, 28%) or complete (82/114, 72%) remission. At 2 months after diagnosis, remission rate was 85%. The median time from diagnosis to remission was 22 days [15‐54]. Among the 114 dogs achieving remission, 92 had available data for survival analysis. Forty‐two dogs (45%) had experienced at least one relapse 5 months after remission. Median time from remission to relapse was 7.9 months [3.7‐35.9].

Lethargy (RR = 1.6 [1.0‐2.5]_95%_, p=0.047), cervical pain (RR = 1.7 [1.0‐2.8]_95%_, p=0.038), inflammation detected in more than 75% of joints sampled (RR = 1.9 [1.0‐3.5]_95%_, p=0.038), neutrophilic leukocytosis (RR = 1.6 [1.1‐2.4]_95%_, p=0.027), and serum CRP concentration ⩾ 50 mg/L (RR = 1.6 [1.1‐2.5]_95%_, p=0.026) were significantly associated with remission occurrence. Dogs ⩾ 5 years old (RR= 2.4 [1.2‐4.8]_95%_, p=0.009), azotemia (RR=4.0 [1.2‐13.7]_95%_, p=0.026), thrombocytosis (RR= 3.2 [1.4‐7.3]_95%_, p=0.005) and a CRP/Alb ratio ⩾ 3 (RR= 2.6 [1.1‐6.2]_95%_, p=0.037) were significantly associated with relapse occurrence.

This study further reports a good clinical outcome for canine IMPA, with a high remission rate but a relatively high relapse rate. Our results identified potential factors associated with remission and relapse.

**Disclosures**

No disclosures to report

## 60 ESVIM‐O‐4

### 60.1 ESVIM ‐ European Society of Veterinary Internal Medicine

#### 60.1.1 Serum 25‐hydroxyvitamin D and 24,25‐dihydroxyvitamin D in dogs with sinonasal aspergillosis

##### 60.1.1.1 A. Snoeck ^1^, J. Jaffey ^2^, F. Billen ^1^, S. Peeters ^1^, C. Le Goff ^1^, D. Peeters ^1^, N. Fernandes Rodrigues ^1^, E. Cavalier ^1^, C. Clercx ^1^

###### 60.1.1.1.1 ^1^ University of Liège Liège Belgium; ^2^ Midwestern University Arizona United States

Sinonasal aspergillosis (SNA) is a common cause of chronic nasal disease with a still poorly understood pathophysiology and which remains a challenge to treat. There is increasing evidence that vitamin D plays a role in both innate and adaptative immunity. A preliminary retrospective study showed that serum 25‐hydroxyvitamin D concentration, measured by high performance liquid chromatography (HPLC), was significantly lower in dogs affected with SNA compared to healthy dogs. Objectives of this prospective study were 1) to compare serum 25(OH)D and 24,25(OH)_2_D concentrations in dogs with SNA to healthy control dogs and dogs with lymphoplasmacytic rhinitis (LPR) or nasal neoplasia; and 2) to determine if serum 25(OH)D and 24,25(OH)_2_D concentrations in dogs with SNA change from the time of diagnosis to when a cure is achieved. Twenty dogs with a novel diagnosis of SNA, 12 healthy control dogs, 9 dogs with LPR, 10 dogs with nasal neoplasia were included. Nine dogs with SNA were available for follow up until cure. Serum vitamin D concentrations were measured by liquid chromatography tandem mass spectrometry (LC‐MS/MS) and compared: 1) among the different groups using a One‐way ANOVA and 2) from diagnosis to cure with a paired t‐test (significant p‐value <0,05 for both tests). The vitamin D metabolite ratio (VMR) was calculated by dividing the 25(OH)D by the 24,25(OH)_2_D concentration. Serum 25(OH)D and 24,25(OH)_2_D were lower in dogs with SNA at the time of diagnosis (mean ± standard deviation = 23.5 ± 7,1 ng/ml – 10,5 ± 4,2 ng/ml, respectively) than in healthy dogs (34,1 ± 7,5 ng/ml; p=0,017 ‐ 18,2 ± 5.4 ng/ml; p = 0,005) while there was no difference between healthy and dogs with tumor (27,8 ± 10,9 ng/ml – 15,4 ± 6,5 ng/ml) or LPR (27,4 ± 13,7 ng/ml – 14,3 ± 8,7 ng/ml). There was no significant difference in serum 25(OH)D and 24,25(OH)_2_D between dogs with SNA at the time of diagnosis and dogs achieving cure. The VMR was higher in SNA dogs (2,4 ± 0,7) than in control dogs (1,9 ± 0,3; p=0,031 t‐test), indicating a decreased catabolic clearance of vitamin D in SNA dogs. These results further support the rationale that vitamin D could play a role in dogs with SNA as it does in human with aspergillosis. Whether hypovitaminosis D could contribute to the development of SNA or if oral supplementation could be a beneficial adjunctive therapy in affected dogs is unknown and warrants future investigations.

**Disclosures**

No disclosures to report

## 61 ESVIM‐O‐5

### 61.1 ESVIM ‐ European Society of Veterinary Internal Medicine

#### 61.1.1 Sinonasal Aspergillosis in dogs: a retrospective study of treatment and outcome in multiple UK referral centres (2011‐2021)

##### 61.1.1.1 C.D. Prior ^1^, A. Kent ^1^, D. Batchelor ^2^, M. Best ^3^, N. Bommer ^4^, S. Borgonovi ^5^, R. Burrow ^6^, S. Calleja ^7^, I. Calvo‐Saiz ^8^, L.E. Castro ^9^, K. Clarke ^10^, I. Elgueta ^11^, A. Farges ^9^, S. Fowlie ^8^, C. Gil‐Morales ^12^, B. Glanemann ^13^, M. Hernandez‐Perello ^14^, A. Hrovat ^15^, E. Izaguirre ^16^, P. Jamieson ^11^, M. Keane ^2^, D. Kelly ^17^, J. Kennils ^12^, A. Kortum ^18^, N. Lau ^10^, C. Lea ^17^, E. Lopez ^12^, K. Murphy ^19^, A. Paul ^20^, C. Piazza ^15^, N. Reed ^21^, A. Ridyard ^9^, E. Roberts ^22^, L. Rutherford ^13^, C. Shales ^1^, M. Sharman ^23^, E.J. Shelton ^13^, G. Specchia ^15^, S. Spence ^16^, H. Swales ^24^, A. Tamborini ^25^, S. Tappin ^26^, N. Steen ^27^, F. Allerton ^1^

###### 61.1.1.1.1 ^1^ Willows Veterinary Centre & Referral Service Birmingham United Kingdom; ^2^ Small Animal Teaching Hospital. University of Liverpool Liverpool United Kingdom; ^3^ Eastcott Veterinary Hospital Swindon United Kingdom; ^4^ Vet Specialists Scotland Livingston United Kingdom; ^5^ Paragon Referrals Wakefield United Kingdom; ^6^ Northwest Vet Specialists Runcorn United Kingdom; ^7^ Lumbry Park Veterinary Specialists Alton United Kingdom; ^8^ Southfields Veterinary Specialists Basildon United Kingdom; ^9^ University of Glasgow Small Animal Hospital, School of Veterinary Medicine Glasgow United Kingdom; ^10^ Davies Vets Hitchin United Kingdom; ^11^ Vets‐Now Hospital Glasgow United Kingdom; ^12^ Langford Small Animal Referral Hospital Bristol United Kingdom; ^13^ Queen Mother Hospital for Animals, Royal Veterinary College London United Kingdom; ^14^ Wear Referrals Stockton‐on‐Tees United Kingdom; ^15^ Pride Veterinary Centre Derby United Kingdom; ^16^ North Downs Specialist Referrals Bletchingley United Kingdom; ^17^ Southern Counties Veterinary Specialists Ringwood United Kingdom; ^18^ University of Cambridge, Department of Veterinary Medicine Cambridge United Kingdom; ^19^ Rowe Referrals Bristol United Kingdom; ^20^ Anderson Moores Veterinary Specialists Hampshire United Kingdom; ^21^ Veterinary Specialists Scotland Livingstone United Kingdom; ^22^ Highcroft Veterinary Referrals Bristol United Kingdom; ^23^ VetCT Cambridge United Kingdom; ^24^ Moorview Referrals Cramlington United Kingdom; ^25^ IDEXX Laboratories Italia Milano United Kingdom; ^26^ Dick White Referrals Cambridgeshire United Kingdom; ^27^ Cave Veterinary Specialists Wellington United Kingdom

A variety of treatment protocols have been described to manage canine sinonasal aspergillosis (SNA). An optimal approach has not been established.

This multi‐centre retrospective study compares outcomes for dogs managed by oral anti‐fungal therapy only (ORAL), topical anti‐fungal therapy without debridement (TOPIC), rhinoscopic debridement with or without topical antifungals (RHINO), trephination +/‐ administration of topical antifungals (TREPH), placement of temporary indwelling catheters (CATH) or sinusotomy/rhinotomy (OTOMY). Inclusion was based on compatible clinical signs and imaging findings, ± observation of fungal plaques. Clinical outcome was categorised as complete, partial, or no resolution. First treatment and overall, complete resolution remission rates were compared by treatment modality.

620 case records were recorded into a data capture platform (CastorEDC) from 23 referral practices. Forty‐nine records lacked outcome details and were excluded from this analysis. Six dogs were euthanized without treatment. Cases were excluded from comparative analysis if first treatment involved a combination of rhinoscopic (RHINO or TOPIC) and surgical (TREPH, CATH or OTOMY) treatments (n=102).

First treatment outcome, recorded a median of 23 days (IQR 14‐35) after treatment, was available for 463 dogs; 22 dogs managed without any antifungal medication were excluded. Complete resolution was achieved after first treatment in 0/17 (0%) ORAL; 15/63 (24%) TOPIC; 45/109 (41%) RHINO; 65/197 (33%) TREPH; 2/5 (40%) CATH and 23/50 (46%) of OTOMY dogs. Further (second or more) treatment was pursued in 361 dogs (including some with complete clinical remission); 80 dogs were lost to follow‐up at this stage.

A further 49 cases were excluded from final outcome comparison after deviating from primary treatment type. Final treatment outcome data was available for 312 dogs. Complete resolution was achieved in 3/7 (43%) ORAL; 32/45 (71%) TOPIC; 61/76 (80%) RHINO; 97/150 (65%) TREPH; 2/2 (100%) CATH and 23/32 (72%) of OTOMY dogs.

Overall rates of complete resolution were lower for each modality than previously reported. In this population of patients, clinicians favoured treatment with RHINO and TREPH. Given the retrospective nature of the study it is unknown if this decision was influenced by the extent of the disease or clinician familiarity. However, at least 78% of patients required additional repeat procedures highlighting the challenging nature of this condition.

**Disclosures**

No disclosures to report

Funding: The Small Animal Medicine Society (SAMSoc)

## 62 ESVIM‐O‐6

### 62.1 ESVIM ‐ European Society of Veterinary Internal Medicine

#### 62.1.1 Sinonasal Aspergillosis in dogs: a retrospective review of diagnostic findings in multiple UK referral centres (2011‐2021)

##### 62.1.1.1 C.D. Prior ^1^, A. Kent ^1^, D. Batchelor ^2^, M. Best ^3^, N. Bommer ^4^, S. Borgonovi ^5^, R. Burrow ^6^, S. Calleja ^7^, I. Calvo‐Saiz ^8^, L.E. Castro ^9^, K. Clarke ^10^, I. Elgueta ^11^, A. Farges ^9^, S. Fowlie ^8^, C. Gil‐Morales ^12^, B. Glanemann ^13^, M. Hernandez‐Perello ^14^, A. Hrovat ^15^, E. Izaguirre ^16^, P. Jamieson ^11^, M. Keane ^2^, D. Kelly ^17^, J. Kennils ^12^, A. Kortum ^18^, N. Lau ^10^, C. Lea ^17^, E. Lopez ^12^, K. Murphy ^19^, A. Paul ^20^, C. Piazza ^15^, N. Reed ^21^, A. Ridyard ^9^, E. Roberts ^22^, L. Rutherford ^13^, C. Shales ^1^, M. Sharman ^23^, E.J. Shelton ^13^, G. Specchia ^15^, S. Spence ^16^, H. Swales ^24^, A. Tamborini ^25^, S. Tappin ^26^, N. Steen ^27^, F. Allerton ^1^

###### 62.1.1.1.1 ^1^ Willows Veterinary Centre & Referral Service Birmingham United Kingdom; ^2^ Small Animal Teaching Hospital. University of Liverpool Liverpool United Kingdom; ^3^ Eastcott Veterinary Hospital Swindon United Kingdom; ^4^ Vet Specialists Scotland Livingston United Kingdom; ^5^ Paragon Referrals Wakefield United Kingdom; ^6^ Northwest Vet Specialists Runcorn United Kingdom; ^7^ Lumbry Park Veterinary Specialists Alton United Kingdom; ^8^ Southfields Veterinary Specialists Basildon United Kingdom; ^9^ University of Glasgow Small Animal Hospital, School of Veterinary Medicine Glasgow United Kingdom; ^10^ Davies Vets Hitchin United Kingdom; ^11^ Vets‐Now Hospital Glasgow United Kingdom; ^12^ Langford Small Animal Referral Hospital Bristol United Kingdom; ^13^ Queen Mother Hospital for Animals, Royal Veterinary College London United Kingdom; ^14^ Wear Referrals Stockton‐on‐Tees United Kingdom; ^15^ Pride Veterinary Centre Derby United Kingdom; ^16^ North Downs Specialist Referrals Bletchingley United Kingdom; ^17^ Southern Counties Veterinary Specialists Ringwood United Kingdom; ^18^ University of Cambridge, Department of Veterinary Medicine Cambridge United Kingdom; ^19^ Rowe Referrals Bristol United Kingdom; ^20^ Anderson Moores Veterinary Specialists Hampshire United Kingdom; ^21^ Veterinary Specialists Scotland Livingstone United Kingdom; ^22^ Highcroft Veterinary Referrals Bristol United Kingdom; ^23^ VetCT Cambridge United Kingdom; ^24^ Moorview Referrals Cramlington United Kingdom; ^25^ IDEXX Laboratories Italia Milano United Kingdom; ^26^ Dick White Referrals Cambridgeshire United Kingdom; ^27^ Cave Veterinary Specialists Wellington United Kingdom

Various ancillary diagnostic tests are available to confirm the diagnosis of sinonasal aspergillosis (SNA). This multi‐centre retrospective study describes the diagnostic tests used and their sensitivity in dogs diagnosed with SNA in the UK.

Dogs were included if SNA was diagnosed based on a combination of clinical signs and imaging findings +/‐ observation of fungal plaques. Case details were collected using a data capture platform (CastorEDC). Results of ancillary diagnostic tests were recorded and sensitivity calculated.

Six hundred twenty cases of SNA were identified from 23 centres. Computed tomography (CT) was performed in 548/620 (88%), magnetic resonance imaging (MRI) in 34/620 dogs (5%) and skull radiography in 26/620 (4%) of dogs. Rhinoscopy was performed in 530/620 dogs (85%); fungal plaques were visualized in 360/530 (68%), and 392/530 (74%) had evidence of turbinate destruction. CT or MRI demonstrated turbinate destruction in 566/582 (97%), with involvement of the frontal sinus in 341/582 (58%) and nasal cavity in 554/582 (95%). Thickened reactive bone 118/582 (20%) and cribriform plate lysis 56/582 (10%), were less common.

Histopathology was performed in 317 dogs (51%). Fungal hyphae were detected in 158 (50%) and absent in 159 (50%) samples (test sensitivity 50%). Fungal culture was performed in 290 dogs (47%) on 114 fungal plaques (39%), 131 mucosal biopsies (45%) and 23 nasal swabs (8%); 35 sample types were not recorded (12%). Culture was positive in 157 (54%) and negative in 129 (44%) of the samples (sensitivity 55%), 4 samples had indeterminate results. Aspergillus serology was performed in 102 (16%) dogs with 54 samples positive (sensitivity 53%). Cytology was performed in 79 (13%) dogs using direct smears from the nasal exudate in 21 (27%), squash preparations in 31 (39%), brushings of plaques with endoscopic guidance in 15 (19%) and blind endonasal swabs 2 (3%) with 10 samples from unrecorded sites. Cytology was positive in 48 (61%), negative in 18 (23%) and unknown in 13 samples (sensitivity 73%). Aspergillus PCR was performed in 9 (1%) dogs and was positive in 6. No ancillary diagnostic testing was performed in 168 dogs (27%).

Ancillary tests were pursued in a majority of dogs to confirm SNA. Of these tests, cytology was found to be most sensitive, (although some tests may be affected by low sample numbers risking a type I error). The sensitivities of histopathology and culture were lower than previously reported despite preferential use of appropriate sample type (fungal plaques).

**Disclosures**

No disclosures to report

Study funded: The Small Animal Medicine Society (SAMSoc)

## 63 ESVIM‐O‐7

### 63.1 ESVIM ‐ European Society of Veterinary Internal Medicine

#### 63.1.1 Nasopharyngeal stenosis in cats : a retrospective study of 21 cases comparing endoscopic and surgical treatment (2018‐2022)

##### 63.1.1.1 A. Champetier, J Lemetayer, O Dossin

###### 63.1.1.1.1 ADVETIA Veterinary Referral Hospital Vélizy‐Villacoublay France

Feline nasopharyngeal stenosis (NPS) is an uncommon disease. To the best of our knowledge comparison between endoscopic and surgical treatment has not been reported.

Medical record was searched for cases of NPS and cases were divided in two groups: endoscopic (balloon dilation) or surgical treatment. Data are presented as median and range. Comparison of success rates between balloon dilation and surgery was assessed with a Chi square test.

Twenty one cases were included: 10 males and 11 females with a median age of 6.7 years (0.5‐14.4). Clinicals signs included permanent stertor (19/21), increased respiratory efforts (9/21), nasal discharge (9/21) and cough/reverse sneezing (4/21) with a duration of 3 to 60 months. Diagnosis was based on endoscopy (21 cases) and CT (19 cases). Twelve cases were treated with endoscopic balloon dilation with 11 success (2 cats had 2 dilations) and 1 failure that was treated surgically after 1 unsuccessful balloon dilation. Eight cats were treated surgically including one case with silicone stenting that was considered as a failure. Two cats were not treated and are still alive more than 3 years after diagnosis. The success rate was not different between endoscopic (11/12) and surgically treated cases (7/8) (p=0.85). The long‐term outcome (6 to 48 months) was considered excellent with a success rate of 18/23 procedures and 17/19 treated cats had no clinical signs to minimal stertor.

This preliminary study suggests that minimally invasive endoscopic treatment of NPS has a high success rate and should be attempted in all cases.

**Disclosures**

No disclosures to report

## 64 ESVIM‐O‐8

### 64.1 ESVIM ‐ European Society of Veterinary Internal Medicine

#### 64.1.1 Evaluation of thermophysiological characteristics of brachycephalic obstructive airway syndrome utilizing the combination of a cold stimulus and infrared thermography.

##### 64.1.1.1 T. Lee‐Fowler, J Gallman , M Grobman , S Clark‐Price

###### 64.1.1.1.1 Auburn University Auburn United States

Brachycephalic obstructive airway syndrome (BOAS) is a prevalent breed related condition resulting in increased morbidity and mortality from airflow restriction and inflammation. Infrared thermography (IRT) detects heat associated with inflammation allowing investigation of physiology associated with pathologic heat production. The aim of this study was to investigate a novel technique combining a cold stimulus and IRT for evaluation of BOAS thermophysiology.

Dogs with BOAS (BOAS; n=10), brachycephalic dogs without BOAS (Brachy; n=7), and mesocephalic control dogs (Meso; n=14) were recruited. IRT temperatures (T_mean_) were acquired from the region of interest under the neck (pharyngeal area) at baseline and at time 0, 5, and 15 minutes after application of a cool pack. Statistical analysis was completed using t‐test, one‐way repeated measures ANOVA, rank sum test, and repeated measures ANOVA on ranks, respective to the distribution of the data (*p*<0.05).

No difference in age and weight was found between groups (*p*>0.05). BOAS had a higher BCS than Meso (*p*=0.048). T_mean_ was significantly lower at time 0 compared to baseline in all groups *(p*<0.001); however, T_mean_ returned to baseline by 5 minutes in Brachy and BOAS groups (p<0.001) and 15 minutes in the Meso group (*p*<0.001).

Brachycephalic dogs, with or without clinical signs of BOAS, demonstrate faster rewarming of the pharyngeal area after a cold stimulus when compared to Meso dogs. Faster rewarming has been demonstrated in humans with obstructive airway disease. IRT in combination with a cold stimulus can be successfully utilized to assess thermophysiology of BOAS.

**Disclosures**

Disclosures to report, please report below

Drs. Lee‐Fowler, Grobman and Clark‐Price are employed by Auburn University. Dr. Gallman is a SAIM resident at Auburn University and is also employed by the U.S. Army. Drs. Lee‐Fowler & Grobman disclose grant funding by the AVMF/VPRF research grant in the amount of $10,819. Dr. Clark‐Price discloses $300 for speaking & consultancies.

## 65 ESVIM‐O‐9

### 65.1 ESVIM ‐ European Society of Veterinary Internal Medicine

#### 65.1.1 Evaluation of Infrared Thermography and 6 Minute Walk Tests to Assess Thermoregulation and Exercise Intolerance in Dogs with Brachycephalic Obstructive Airway Syndrome

##### 65.1.1.1 J Gallman, M Grobman, T Lee‐Fowler, S Clark‐Price

###### 65.1.1.1.1 Auburn University AUBURN United States

Brachycephalic obstructive airway syndrome (BOAS) is associated with significant morbidity and mortality. Routine clinical evaluation is insufficient to detect physiologic consequences of BOAS including exercise intolerance, and impaired thermoregulation. Infrared thermography (IRT) has been used in dogs to assess thermoregulation. Combined use of a six‐minute walk test (6MWT) and IRT may aid clinical management by assessing the physiologic consequences of BOAS. Our objectives were to compare 6MWT and IRT parameters between healthy mesocephalic (Meso, n=16) and brachycephalic (Brachy; n=10) dogs, and dogs with clinical BOAS (BOAS; n=10). 6MWT parameters (distance walked (D), rectal temperature, pulse, respiratory rate, and SPO2) and IRT temperatures (T_mean_) at 3 regions of interest over the head/neck (rostral, dorsal, pharyngeal) were collected. Evaluation timepoints were pre‐6MWT, T_0_, T_5min_, and T_15min_. Comparisons were made by Mann‐Whitney Rank Sum Test, One‐way ANOVA on Ranks, and Spearman Rank Order correlation (*p*<0.05). No significant difference in D, pulse oximetry, or rectal temperatures were found between groups (*p*>0.05). BOAS dogs showed significantly increased dorsal and rostral IRT temperatures compared to Meso dogs at all timepoints (*p*<0.05). BOAS dogs had increased IRT temperatures compared to Brachy dogs at baseline and T_15_ and T_5_ and T_15_ for dorsal and rostral IRT temperatures respectively (*p*<0.001). IRT did not correlate with D (*p*>0.05). In conclusion, IRT may detect subclinical inflammation and impaired thermoregulation, characterized by increases in T_mean_ in dogs with clinical BOAS.

**Disclosures**

No disclosures to report

## 66 ESVIM‐O‐10

### 66.1 ESVIM ‐ European Society of Veterinary Internal Medicine

#### 66.1.1 Expression of Fibroblast Activation Protein in lungs of dogs with idiopathic pulmonary fibrosis and dogs with lung cancer

##### 66.1.1.1 E. Rizzoli ^1^, A. Fastrès ^1^, E. Roels ^1^, M.M. Garigliany ^1^, T. Marichal ^2^, E. Puré ^3^, C. Clercx ^1^

###### 66.1.1.1.1 ^1^ FARAH, Faculty of Veterinary Medicine, University of Liège Liège Belgium; ^2^ GIGA Institute and Faculty of Veterinary Medicine, University of Liège Liège Belgium; ^3^ University of Pennsylvania Philadelphia United States

Canine Idiopathic Pulmonary Fibrosis (CIPF) is a progressive fibrotic interstitial lung disease of unknown aetiology, affecting predominantly the West Highland White Terrier (WHWT) breed. Currently, there is no curative treatment option available. Fibroblast Activation Protein (FAP) is a cell surface protease usually absent from normal tissue but specifically expressed in areas of active tissue remodelling such as in fibroblast foci in human idiopathic pulmonary fibrosis. In humans, it is also upregulated in various types of cancers, either in cancer‐associated fibroblasts (CAFs), in cancer cells or in both, depending on the tumour type. The aim of this study was to assess the expression and localization of FAP in the lungs of WHWTs affected with CIPF, in comparison with WHWTs with healthy lungs and dogs with lung cancer.

Post‐mortem formalin‐fixed lung biopsies prepared from WHWTs with CIPF (n=17, age from 10 to 15y), control WHWTs exempt from lung disease (n=4, age from 11 to 15y) and dogs from various breeds with lung cancer (n=8, age from 8 to 14y) were retrospectively used. Included lung neoplasia were adenocarcinomas (n=6), histiocytic sarcoma (n=1) and metastasized mammary adenocarcinoma (n=1). Immunohistochemistry (IHC) was performed using a rabbit anti‐human FAP monoclonal antibody (#ab207178). An IHC staining index (absent, low, moderate or high) was attributed according to the percentage of positive cells combined with the staining intensity.

FAP was identified in the lungs of 16 out of 17 (94%) WHWTs with CIPF (IHC index high, moderate, or low in respectively 10, 4 and 2 dogs), 2 out of 4 (50%) WHWTs with healthy lungs (1 of each moderate and low), and 7 out of 8 (88%) dogs with lung cancer (high and moderate in respectively 6 and 1 dogs). FAP was expressed by fibroblasts in areas of active fibrosis in CIPF and by CAFs (all types of cancer) and cancer cells (adenocarcinomas only, n=5) in lung tumours.

Results of this study showed that FAP is moderately to markedly expressed by fibroblasts in most dogs affected with either CIPF or lung cancer. Accordingly, FAP should be considered as an interesting potential therapeutic target for both diseases and should encourage further studies in the future. The expression of FAP in healthy lungs of WHWTs should be further investigated, particularly in comparison with FAP expression in dogs from other breeds, as it might serve as an indicator of early fibrosis.

**Disclosures**

No disclosures to report

## 67 ESVIM‐O‐11

### 67.1 ESVIM ‐ European Society of Veterinary Internal Medicine

#### 67.1.1 Bronchiectasis, dynamic bronchomalacia and bronchiectasis/bronchomalacia syndrome in dogs: a retrospective study

##### 67.1.1.1 A. Lyssens, A. Fastrès, C. Clercx, F. Billen

###### 67.1.1.1.1 University of Liège Liège Belgium

The aim of this retrospective study was to describe clinicopathological findings in dogs with bronchiectasis, dynamic generalized bronchomalacia or both, based on endoscopic criteria.

Dogs in which bronchoscopy was performed (between November 2014 and November 2021) and either bronchiectasis and/or bronchomalacia was reported, were retrospectively selected. Videos were reviewed and blindly assessed by 2 independent observers. Dogs were classified into 3 different groups: multifocal and/or diffuse bronchiectasis (BE), bronchomalacia >25% collapse (BM) and bronchiectasis + bronchomalacia (BE/BM). BM was further subdivided into 3 grades according to the severity of the airway collapse: BM‐1 and BE/BM‐1 (25‐50%), BM‐2 and BE/BM‐2 (50‐75%) and BM‐3 and BE/BM‐3 (>75%). The presence of inflammation (mild/moderate or severe), final diagnosis and results of microbiological analysis were recorded.

Sixty‐five client‐owned dogs were retrospectively included, and comprised 16 dogs in the BE group, 31 in the BM group (10, 11 and 10 in BM‐1, BM‐2 and BM‐3, respectively), and 18 in the BE/BM group (5, 11 and 2, in BE/BM‐1, BE/BM‐2 and BE/BM‐3, respectively).

No significant difference was noted in the degree of inflammation (mild/moderate and severe) between the 3 groups (p=0.1025).

Chronic bronchitis was the most commonly diagnosed in BE (10/16 dogs), followed by eosinophilic bronchopneumopathy (5/16) and recurrent bacterial pneumonia with *Pseudomonas spp*. (1/16). In BM, 30/31 dogs were diagnosed with chronic bronchitis whilst 1 dog was diagnosed with idiopathic pulmonary fibrosis. Regarding BE/BM, chronic bronchitis was diagnosed in 14/18 dogs, eosinophilic bronchopneumopathy in 1/18 dog and idiopathic pulmonary fibrosis in 3/18 dogs.

BALF cytology, culture and qPCR for *Bordetella bronchiseptica* and *Mycoplasma cynos* were performed on all dogs. Concomitant bacterial infection was diagnosed in 10/65 dogs, based on clinical signs (all dogs), BALF cytology (all dogs), culture (8/10 dogs) and qPCR testing for *Bordetella bronchiseptica* and *Mycoplasma cynos* (2/10 dogs). No significant difference was noted in presence of concomitant bacterial infection between the 3 groups (p = 0,158). Three dogs received antimicrobials at presentation, none had bacterial infection.

Based on endoscopic findings, we identified a bronchomalacia/bronchiectasis syndrome not previously described in canine medicine. Further characterization of these conditions using dynamic imaging is warranted. All 3 conditions can be associated with mild to severe inflammation and bacterial infection. However, the role of bacteria in their pathogenesis should further be analyzed.

**Disclosures**

No disclosures to report

## 68 ESVIM‐O‐12

### 68.1 ESVIM ‐ European Society of Veterinary Internal Medicine

#### 68.1.1 Seropositivity for Aspergillus and House Dust Mites antigens in dogs with eosinophilic bronchopneumopathy

##### 68.1.1.1 A. Canonne ^1^, P. Loos ^2^, C. Maquet ^2^, F. Billen ^3^, D. Peeters ^3^, L. Gillet ^2^, C. Clercx ^3^

###### 68.1.1.1.1 ^1^ Ecole Nationale Veterinaire d'Alfort Maisons Alfort France; ^2^ Immunology‐Vaccinology, Department of Infectious and Parasitic Diseases Liege Belgium; ^3^ Department of Clinical Sciences, Faculty of Veterinary Medicine Liege Belgium

Canine eosinophilic bronchopneumopathy (EBP) is characterized by idiopathic eosinophilic infiltration of the lung and bronchial mucosa. Hypersensitivity reaction to aerosolized antigens is suspected, although previous investigations failed to identify inciting antigens. In humans*, Aspergillus* spp. and house‐dust mites (HDM) have been proposed as potential triggers for allergic bronchopulmonary disease. In dogs in EBP, *Aspergillus* has occasionally been detected by culture or quantitative PCR in bronchoalveolar lavage fluid (BALF) but *Aspergillus*‐ and HDM‐specific antibodies have not yet been measured neither in serum nor in BALF.

The aim of the study was to quantitatively assess the presence of *A. fumigatus‐*and HDM‐specific immunoglobulins E (IgE) and G (IgG) in serum and BALF from dogs with EBP in comparison with samples from dogs with chronic bronchitis (CB) and healthy dogs.

Sera and BALF samples collected from 17 dogs with EBP, 9 dogs with CB and 10 healthy dogs were retrospectively analysed using a homemade validated ELISA to detect *A. fumigatus‐* and HDM‐specific IgE and IgG at several dilutions. Comparison between EBP dogs and CB dogs and between EBP dogs and healthy dogs were performed with Mann Whitney test. Values of *P* ≤ 0.05 were considered significant.

*A.fumigatus*‐specific IgG were significantly higher in the serum of dogs with EBP, compared with dogs with CB or healthy dogs, at both 1/1000 (p<0.01) and 1/10 000 dilutions (p<0.05 and p<0.01); however the concentration of these specific IgG in BALF did not differ between groups. Dogs with EBP had also increased HDM‐specific IgG in both serum and BALF compared to dogs with CB (but not compared to healthy dogs), at both 1/1000 (p<0.001) and 1/10 000 dilutions (p<0.01).

Concentrations of *A.fumigatus*‐specific IgE in serum or BALF were very low in all dogs and there was no difference between groups.

In conclusion, dogs with EBP seem to have higher concentrations of serum *A. fumigatus*‐ specific IgG than dogs with CB and healthy dogs as well as higher concentration of serum HDM‐specific IgG than dogs with CB. In the future, deciphering the relationship between those IgG and lung hypersensitivity as well as investigating of reactions to additional environmental saprophytes or mites’ allergens will be needed to better understand the pathogenesis of canine EBP.

**Disclosures**

No disclosures to report

## 69 ESVIM‐O‐13

### 69.1 ESVIM ‐ European Society of Veterinary Internal Medicine

#### 69.1.1 Prospective evaluation of the efficacy of inhaled steroids administered via the AeroDawg spacing chamber in management of dogs with chronic cough

##### 69.1.1.1 J. Chan, L. Johnson

###### 69.1.1.1.1 Davis United States

Glucocorticoids are required for management of cough due to inflammatory airway disease (IAD) and airway collapse (AWC). However, oral steroids have a plethora of undesirable systemic side effects.

The aim of this study was to determine the efficacy of inhaled steroids in controlling cough in dogs with airway disease.

Client‐owned dogs with cough were recruited for this prospective, placebo‐controlled cross‐over study. IAD was diagnosed through bronchoalveolar lavage cytology. AWC was diagnosed through bronchoscopy, or if dogs were unsuitable anesthetic candidates, by crackles on auscultation, radiographic changes in airway diameter, and/or fluoroscopy. Dogs were randomly assigned to placebo or fluticasone propionate for the first two weeks of the trial then crossed over to fluticasone. A quality of life (QOL) survey (best score 0, worst score 85) was completed at 0 and 6 weeks. A visual‐analog cough survey was submitted at 0, 2, 4, and 6 weeks.

After one year of recruitment, interim analysis demonstrated a significant difference (P < 0.05) in QOL and cough scores in treated versus placebo groups. Therefore, only the treatment arm was continued. A total of 26 dogs completed the study. QOL score at study end (median 5, range 1‐36) was significantly decreased (P < 0.0001) compared to study entry (median 21, range 5‐62), with a median 74% improved QOL score. Cough frequency, duration, and severity were significantly (P < 0.0001) decreased at the 6‐week time point.

This study supports the use of inhaled fluticasone propionate for management of cough due to IAD or AWC.

**Disclosures**

Disclosures to report, please report below

Funding provided by the Center for Companion Animal Health at UC Davis and Trudell Medical International

## 70 ESVIM‐O‐14

### 70.1 ESVIM ‐ European Society of Veterinary Internal Medicine

#### 70.1.1 Allergy testing in cats with feline lower airway disease

##### 70.1.1.1 B. Hartung, R. Mueller, T. Boehm, T. Weitzer, J. Palic, B. Schulz

###### 70.1.1.1.1 München Germany

Feline asthma (FA) and chronic bronchitis (CB) are common feline lower airway diseases (FLAD) in cats. As in humans, allergens are discussed as potential triggers. The aim of this study was to investigate results of intradermal testing (IDT) and serum allergen‐specific immunoglobulin‐E‐ (IgE‐) testing (SAT) in affected cats.

Sixteen Cats with clinical, radiographic, and cytological signs consistent with a diagnosis of FA or CB were prospectively included. Standardized IDT was performed in 16 cats, and SAT in 11. The control group for SAT were 10 healthy cats.

In cats with FLAD, 15/16 showed reactions on IDT and 6/11 on SAT, while 9/10 control cats had positive reactions on SAT. When reactions of IDT and SAT of cats with FLAD were compared, significantly more positive reactions were detected on IDT (20,5%) than on SAT (9,1%) (p=0.019). The most common reactions on IDT in cats with FLAD were ambrosia (6/16), Bermuda grass (5/16) and timothy grass (4/16). On SAT, reactions against American house dust mite (4/11), timothy grass (3/11) and Kentucky bluegrass (3/11) were most prevalent. There was no significant difference in the number of positive reactions on SAT between cats with FLAD and healthy cats (p=0.253).

IDT revealed more positive reactions in cats with FLAD than SAT. However, healthy cats did not differ in the number of IgE‐responses compared to sick cats, therefore, allergy test results need to be interpreted in the context of clinical history and environment.

**Disclosures**

No disclosures to report

## 71 ESVIM‐O‐15

### 71.1 ESVIM ‐ European Society of Veterinary Internal Medicine

#### 71.1.1 Correlation of radiographic and clinical parameters in cats with feline lower airway disease

##### 71.1.1.1 H. Gareis ^1^, L Hörner ^2^, J Palić ^3^, S Hecht ^4^, B. Schulz ^2^

###### 71.1.1.1.1 ^1^ Ludwig Maximilian University of Munich Munich Germany; ^2^ Clinic of Small Animal Medicine Munich Germany; ^3^ Vet Med Labor GmbH, Division of IDEXX Laboratories Ludwigsburg Germany; ^4^ University of Tennessee, College of Veterinary Medicine Knoxville United States

Airway inflammation in cats with feline lower airway disease (FLAD) leads to typical clinical and radiological abnormalities. In cats with FLAD, therapeutic success is commonly evaluated by clinical examination only. Studies regarding improvement of radiographic findings with adequate therapy show controversial results.

The aim of the present study was to investigate, whether radiographic findings in cats with FLAD improve with therapy, and to evaluate the correlation with clinical signs.

Twenty‐four cats with typical clinical signs, diagnosed with feline asthma or sterile chronic bronchitis on the basis of cytological examination of bronchoalveolar lavage fluid, were prospectively included in the study.

At two examination time points (day 0 and day 60), a clinical evaluation of the patients according to a standardised examination protocol (clinical 12‐point‐score) and a radiographic examination of the thorax in two views (radiological 10‐point‐score) were performed. Therapy with corticosteroids +/‐ bronchodilators was given to all patients from day 0, based on disease severity and compliance of the cat.

Clinical and radiographic scores were statistically compared between both time points using Wilcoxon‐signed‐rank‐test, and then evaluated for correlation using Kendall‐Tau correlation‐coefficient. The significance level was p ≤ 0.05.

The clinical score improved significantly over the two time points (p<0.001). The radiological score also showed significant improvement during therapy (p=0.005). Clinical and radiographic scores correlated significantly on day 60 (p=0.015).

Both clinical and radiographic scores improved with therapy and correlated. In addition to clinical signs, radiographic findings can be used to monitor the success of therapy in cats with FLAD.

**Disclosures**

No disclosures to report

## 72 ESVIM‐O‐16

### 72.1 ESVIM ‐ European Society of Veterinary Internal Medicine

#### 72.1.1 Utility of ultrasound‐guided fine needle aspiration of non‐circumscribed pulmonary lesions in dogs and cats.

##### 72.1.1.1 A. Preibisz, C. Schwedes

###### 72.1.1.1.1 Anicura Augsburg Augsburg Germany

Ultrasound‐guided fine needle aspiration of the lungs usually is performed to diagnose solitary lesions shown by diagnostic imaging. The aim of this retrospective study is to evaluate the utility of ultrasound‐guided fine needle aspiration of pulmonary lesions usually seen as interstitial or alveolar lung patterns affecting one or more lung lobes which cannot be described as pulmonary masses. A total number of 110 animals (69 dogs, 41 cats) was included in the study.

Cases of dogs and cats with pulmonary lesions other than circumscribed pulmonary or mediastinal masses that underwent an ultrasound‐guided pulmonary fine needle aspiration between 2009 and 2021 were reviewed retrospectively. The cytological results were classified as normal lung pattern, inflammatory pattern, or neoplastic pattern. For some of the cases, bacterial culture was performed. Adverse effects after fine needle aspiration were noted.

The probability of obtaining a diagnostic sample amounted to 90,7%. Only 5 out of 110 patients showed clinical adverse effects that could possibly be linked to the procedure, among these were 2 patients that died within less than 48 hours after the procedure. Overall, in 88,2 % of the patients, the cytology was decisive for the further management of the patient. In 38% of the patients, a definitive diagnosis could be reached without further diagnostics.

We conclude that ultrasound‐guided fine needle aspiration of non‐circumscribed pulmonary lesions is a valuable alternative diagnostic tool for diagnosing diseases of the lower respiratory tract as opposed to bronchoscopy with bronchoalveolar or tracheobronchial lavage, especially in unstable or severely dyspnoeic patients. Lower cost and the dispensability of general anesthesia could encourage some patients’ owners to pursue further diagnostics. In many cases of a diffuse neoplastic pulmonary process, a correct diagnosis can be reached quickly and thus avoids unnecessary suffering of the animal.

Cytology enables a rapid diagnosis of bacterial pneumonia. Moreover, aspirates can be used for bacterial culture. Fine needle aspiration can be repeated in patients with bacterial pneumonia treated with antibiotics to confirm therapeutic efficacy.

**Disclosures**

No disclosures to report

## 73 ISCAID‐O‐1

### 73.1 ISCAID ‐ International Society for Companion Animal Infectious Diseases

#### 73.1.1 Fecal Feline Coronavirus Shedding and Spike Gene Mutations in Cats with Feline Infectious Peritonitis Treated with GS‐441524

##### 73.1.1.1 M. Meli ^1^, A. Spiri ^1^, K. Zwicklbauer ^2^, D. Krentz ^2^, S. Felten ^2^, M. Bergmann ^2^, R. Dorsch ^2^, K. Matiasek ^3^, M. Alberer ^4^, L. Kolberg ^4^, U. Von Both ^4^, K. Hartmann ^2^, R. Hofmann‐Lehmann ^1^

###### 73.1.1.1.1 ^1^ Clinical Laboratory and Center for Clinical Studies Zürich Switzerland; ^2^ Clinic of Small Animal Medicine, Centre for Clinical Veterinary Medicine, LMU Munich Germany; ^3^ Institute of Veterinary Pathology, Centre for Clinical Veterinary Medicine, LMU Munich Germany; ^4^ Dr. von Hauner Children's Hospital, University Hospital, LMU Munich Germany

Feline infectious peritonitis (FIP) caused by feline coronavirus (FCoV) is a fatal disease if untreated. A recent prospective controlled treatment trial in field cats with confirmed FIP demonstrated excellent efficacy of GS‐441524.

The aims of this study were to investigate the effect of GS‐441524 treatment on fecal FCoV RNA shedding and presence of FCoV spike (S‐) gene mutations in different body compartments in treated FIP cats as well as in 12 companion cats cohabitating with the FIP cats. Eighteen cats with confirmed FIP were treated with oral GS‐441524 for 84 days. Viral loads in feces, blood, and effusion were determined by RT‐qPCR. In the first three days of treatment, 11/18 treated FIP cats (61%) shed FCoV RNA in feces, but all of them tested negative by day six. In one of them, fecal shedding reoccurred on day 83. Two cats initially negative in feces were transiently positive 1–4 weeks into the treatment. FCoV RNA loads in feces decreased in all treated FIP cats with time, comparable with those in blood and effusion. S‐gene mutations linked to systemic FCoV spread were consistently found in blood and effusion from treated FIP cats, but not in feces from treated or companion cats. Phylogenetic analyses of the S‐gene revealed a clustering of fecal samples of the companion cats with the corresponding FIP cats.

Oral treatment with GS‐441524 effectively decreased viral RNA loads in feces, blood, and effusion in cats with FIP. Nonetheless, re‐shedding can occur, most likely if cats are re‐exposed to FCoV.

**Disclosures**

No disclosures to report

## 74 ISCAID‐O‐2

### 74.1 ISCAID ‐ International Society for Companion Animal Infectious Diseases

#### 74.1.1 A case series of 25 cats with effusive and non‐effusive feline infectious peritonitis treated with a combination of remdesivir and GS‐441524

##### 74.1.1.1 J. Green, H. Syme, S. Tayler

###### 74.1.1.1.1 The Royal Veterinary College Hatfield United Kingdom

Feline infectious peritonitis (FIP) has until recently carried a guarded prognosis due to a lack of efficacious treatments. Preliminary studies using remdesivir and GS‐441524 for treatment of FIP have shown promising results. Licensing of these drugs for use in humans with COVID‐19 has resulted in improved accessibility for veterinary use in the UK. This case series describes clinical outcomes of cats with FIP treated with injectable remdesivir and oral GS‐441524.

Twenty‐five cats were diagnosed with FIP at a referral hospital in the UK between August 2021 and March 2022. Diagnosis was based on a combination of clinical signs, laboratory, imaging and cytological findings, and immunocytochemistry. Twenty cats presented with effusive FIP and 5 cats with non‐effusive FIP. Five cats had neurological signs and 4 cats had significant ocular involvement.

At the time of writing, 10 cats had successfully completed an 84‐day course of remdesivir and GS‐441524 treatment with complete clinical and biochemical remission. Ten cats were in clinical remission and still receiving treatment and 5 cats were euthanised a median of 2 days (range 1‐13) after starting treatment.

All surviving cats received remdesivir intravenously for a median of 5 days (range 2‐9) at a median dose of 15 mg/kg (range 10‐20). Higher doses were used for cats with non‐effusive FIP, neurological or ocular signs, and for effusive cases presenting with severe clinical signs. All 20 cats demonstrated significant clinical response after a median of 2 days (range 2‐5). Two cats received subcutaneous remdesivir treatment only following initial intravenous therapy. Eight cats received subcutaneous remdesivir for a median of 14 days (range 4‐68) before transitioning onto an equivalent dose of oral GS‐441524. Ten cats received oral GS‐441524 treatment immediately following intravenous remdesivir therapy. Both drugs were well tolerated although local skin reactions and pain with subcutaneous injections of remdesivir was observed.

Of the five cats that were euthanised, three cats were euthanised within 3 days of starting treatment as a result of comorbidities or financial constraints. One ragdoll developed a T3‐L3 myelopathy 7 days into treatment with 15mg/kg remdesivir, with no clinical improvement following dose escalation. Post‐mortem confirmed FIP within the central nervous system. Another ragdoll also developed central nervous system signs 13 days into treatment with 20mg/kg remdesivir and was subsequently euthanised.

This case series demonstrated the successful use of injectable remdesivir and oral GS‐441524 for the treatment of FIP in 20 out of 25 (80%) cats.

**Disclosures**

Disclosures to report, please report below

UK Specials company BOVA contributed financially to remdesivir treatment in one cat. They gave several vials of remdesivir to this cat free of charge.

## 75 ISCAID‐O‐3

### 75.1 ISCAID ‐ International Society for Companion Animal Infectious Diseases

#### 75.1.1 Prevalence of different outcomes of feline leukemia virus infection in four European countries

##### 75.1.1.1 J. Giselbrecht ^1^, S. Jähne ^1^, M. Bergmann ^1^, S. Teichmann‐Knorrn ^2^, M.G. Pennisi ^3^, N. Layachi ^4^, R. Serra ^5^, S. Bo ^6^, M.L Meli ^7^, R. Hofmann‐Lehmann ^7^, K. Hartmann ^1^

###### 75.1.1.1.1 ^1^ Clinic of Small Animal Medicine, LMU Munich Munich Germany; ^2^ Veterinary Clinc Oberhaching Oberhaching Germany; ^3^ Department of Veterinary Sciences, University of Messina Messina Italy; ^4^ Layachi Veterinary Clinic Bordeaux France; ^5^ Investigacao Veterinaria Independente Lisbon Portugal; ^6^ Ambulatorio Veterinario Bo‐Ferro Turin Italy; ^7^ Department of Clinical Diagnostics and Services, Clinical Laboratory Zurich Switzerland

Prevalence of progressive feline leukemia virus (FeLV) infection is known to still be high in cats in Europe, especially in Southern Europe, but prevalence of other outcomes of FeLV infection has not been determined in most countries. The aim of this study was to investigate the prevalence of progressive, regressive, abortive, and focal infection in four European countries, two with a high (Portugal, Italy), and two with a low expected prevalence (Germany, France).

Blood samples of 934 cats (Italy: 269; Portugal: 240; France: 107; Germany: 318) were evaluated for p27 antigen as well as anti‐whole virus, anti‐SU, and anti‐p15E antibodies by enzyme‐linked immunosorbent assay in serum and for proviral DNA by quantitative polymerase chain reaction (qPCR) in whole blood. Positive p27 antigen ELISA results were confirmed by reverse transcriptase‐qPCR detecting viral RNA in saliva swabs and/or blood. Outcome of FeLV infection was categorized as presumptively progressive (antigen‐positive, viral RNA‐positive, provirus‐positive), regressive (antigen‐negative, provirus‐positive), abortive (antigen‐ and provirus‐negative, antibody‐positive) and focal (antigen‐positive, provirus‐negative) infection.

Overall FeLV prevalence was 20.8% in Italy, 20.5% in Portugal, 9.3% in France, and 9.1% in Germany. Prevalence of presumptively progressive, regressive, abortive, and focal infection in Italy was 7.8%, 4.5%, 5.9%, and 2.6%; in Portugal 3.8%, 8.3%, 6.7%, and 1.7%; in France 1.9%, 3.7%, 2.8%, and 0.9%; and in Germany 1.9%, 1.3%, 3.1%, and 2.8%, respectively.

Overall FeLV prevalence is still very high, especially in Southern European countries. Therefore, testing, separation of infected cats, and vaccination are still important measures to reduce risk of FeLV infection.

**Disclosures**

No disclosures to report

## 76 ISCAID‐O‐4

### 76.1 ISCAID ‐ International Society for Companion Animal Infectious Diseases

#### 76.1.1 Feline panleukopenia outbreaks and risk factors in cats in animal shelters

##### 76.1.1.1 T. Rehme ^1^, K. Hartmann ^1^, U. Truyen ^2^, Y. Zablotski ^1^, M. Bergmann ^1^

###### 76.1.1.1.1 ^1^ Clinic of Small Animal Medicine, LMU Munich Germany; ^2^ Institute of Animal Hygiene and Veterinary Public Health, University of Leipzig Leipzig Germany

Outbreaks of feline panleukopenia in shelters are common and often associated with high fatality rates. The aim of this study was to determine risk factors for such outbreaks.

Four shelters (A‐D) with 150 cats were included. Information on the cats, on husbandry, hygiene, and infection management were collected. Diagnosis of feline panleukopenia was based on presence of clinical signs (anorexia, diarrhea, vomiting, and/or fever) and direct fecal virus detection by point‐of‐care (POC) antigen test and/or parvovirus polymerase chain reaction (PCR). Fecal samples were evaluated by PCR, and PCR‐positive samples underwent cell culture and sequencing. Information on cats, husbandry, hygiene, and infection management were analyzed to determine risk factors for feline panleukopenia and parvovirus shedding by logistic regression. At the time of sampling, feline panleukopenia occurred in 28.0% (42/150) of cats (0 in shelter D). Shedding was found in 48.7% (73/150) (A: 21/73; B: 29/73; C: 7/73; D: 16/73). Of 73 PCR‐positive fecal samples, 65.8% (48/73) were culture‐positive; sequencing revealed feline panleukopenia virus (FPV) isolates in 34/48 samples and vaccine virus isolate in 14/48; canine parvovirus was not detected. Feline panleukopenia were significantly more likely in cats from shelter A (p<0.05), unvaccinated cats (p<0.001), and young cats (4 weeks‐2 years; p=0.008). Parvovirus shedding was significantly more common in young cats (p<0.001), cats with feline panleukopenia (p=0.033), and group‐housed cats (p=0.025). Vaccination management has a greater impact on the incidence of feline panleukopenia outbreaks than husbandry and hygiene management. Risk of parvovirus shedding is especially high in young, group‐housed cats.

**Disclosures**

No disclosures to report

## 77 ISCAID‐O‐5

### 77.1 ISCAID ‐ International Society for Companion Animal Infectious Diseases

#### 77.1.1 Presence of potential enteropathogens and their association with diarrhoea in multi‐cat households

##### 77.1.1.1 K. Bogedale ^1^, U Klein‐Richers ^1^, S Felten ^1^, M Bergmann ^1^, N Pantchev ^2^, Y Zablotski ^1^, J Suchodolski ^3^, K Busch ^1^, S Unterer ^4^, K Hartmann ^1^

###### 77.1.1.1.1 ^1^ Clinic of Small Animal Medicine, Centre for Clinical Veterinary Medicine Munich Germany; ^2^ IDEXX Laboratories Kornwestheim Germany; ^3^ Gastrointestinal Laboratory, College of Veterinary Medicine,Texas A&M University College Station, TX United States; ^4^ Clinic for Small Animal Internal Medicine, University of Zurich Zurich Switzerland

Diarrhoea in cats is common in multi‐cat households and “faecal panels” are commonly used to diagnose potential enteropathogens in these environments. This prospective study evaluated the presence of potential faecal enteropathogens and their association with diarrhoea in multi‐cat households.

Faeces of 234 cats from 41 multi‐cat (≥ 5 cats) catteries were evaluated for faecal consistency according to a visual scoring system, ranging from 1 to 7 (Purina Fecal Score). Real‐time polymerase chain reaction for *Clostridium perfringens* encoding for enterotoxin gene, *Campylobacter jejuni/coli*, *Salmonella enterica*, *Cryptosporidium* spp., *Tritrichomonas foetus, Giardia* spp., and *Toxoplasma gondii,* and faecal flotation for *Giardia* spp. cysts, coccidia oocysts, *Taenia*/*Echinococcus* spp. as well as *Ancylostoma tubaeforme* eggs were performed. Logistic regression models were applied associating the impact of potential pathogens with diarrhoea (faecal score ≥ 4).

Diarrhoea was present in 23/234 cats. None of the pathogens was associated with diarrhoea (*Clostridium perfringens*: percentage of diarrheic cats (D) 0%, percentage of non‐diarrheic cats (ND) 8.5%, p=0.296; *Campylobacter jejuni/coli*: D 8.7%, ND 4.3%, p=0.664; *Salmonella enterica*: D 4.3%, ND 0.5%, p=0.469;*Cryptosporidium* spp.: D 4.3%, ND 3.8%, p=1.000; *Tritrichomonas foetus*: D 4.3%, ND 6.2%, p=1.000; *Giardia* spp.: D 4.3%, ND 10.9%, p=0.534; *Toxoplasma gondii*: D 0%, ND 0.5%, p=1.000; coccidia: D 0%, ND 2.4%, p=1.000; *Taenia/Echinococcus* spp.: D 0%, ND 1.4%, p=1.000; *Ancylostoma tubaeforme*: D 4.3%, ND 0.5%, p=0.469).

Presence of enteropathogens was not associated with clinical signs of diarrhoea in multi‐cat households. Cats without diarrhoea often possess these organisms as enteric commensals.

**Disclosures**

Disclosures to report, please report below

RT‐qPCR and PCR panel results were kindly provided for free by IDEXX Laboratories. IDEXX played no role in the interpretation of data or in the decision to submit this abstract. Other than receiving the RT‐qPCR and PCR results for free, this research received no ex‐ternal funding.

## 78 ISCAID‐O‐6

### 78.1 ISCAID ‐ International Society for Companion Animal Infectious Diseases

#### 78.1.1 Case control study into acute diarrhoea in dogs potentially caused by Providensia alcalifaciens

##### 78.1.1.1 M. Domingues ^1^, G. Paterson ^2^, S. Salavati Schmitz ^2^

###### 78.1.1.1.1 ^1^ The Royal (Dick) School of Veterinary Studies and The Roslin Institute Edinburgh United Kingdom; ^2^ The Royal (Dick) School of Veterinary Studies and The Roslin Institute EDINBURGH United Kingdom

A recent outbreak of severe acute haemorrhagic diarrhoea in dogs in Norway, thought to be associated with *Providensia alcalifaciens* (PA), prompted the interest to further investigate this potential pathogen. Literature on PA as a cause of enteritis in dogs is scarce. The aim of this study was to describe a population of dogs presenting to a UK referral hospital in which PA was isolated on faecal culture (FC) compared to dogs with similar signs but free of PA. The hospital's database was retrospectively searched for FC of dogs in which PA was detected. Controls, defined as dogs with a FC without PA were selected from the same database and time frame (breed or aged matched wherever possible) at a 2:1 ratio. Inclusion criteria for both groups were the presence of acute diarrhoea (< 14 days duration), availability of clinical data, and routine blood work from the time of presentation. Data on signalment, presenting complaint(s), physical examination findings, CBC, biochemistry, other faecal analysis, diagnostic imaging findings, treatment, duration of hospitalisation and outcome were collected. Descriptive statistics were performed. Logistic regression models and Pearson Chi‐Square tests were used to compare categorical variables. For comparison of numerical variables, t‐tests were performed. Significance was set at p < 0.05. Of 17 cases with PA identified between the years 2017 to 2021, 6 were excluded due to incomplete data. The remaining 11 dogs consisted of 2 mixed breeds and other individual breeds. Median age was 72 months (range 2‐122). For the 22 control dogs, predominant breeds included Jack Russel Terrier and Labrador retriever (n= 3 each) and other individual breeds. Median age was 65 months (range 4‐163). Diagnoses for control dogs included enteritis (n= 16, 4 of those HGE), Giardia (n=3), pancreatitis (n=2) and intussusception (n=1). Dogs with PA were more likely to be dehydrated (p=0.021), require electrolyte supplementation (p=0.022) and transfusion of blood products (p=0.002); and stayed hospitalised for longer (mean 4.55 days, sd 2.38) than the control dogs (mean 3.86, sd 3.31) (p= 0.034). The remaining clinical, clinicopathological and imaging variables were not different between groups. In conclusion, this study suggests that there were no discernible clinical criteria between dogs with PA identified on FC in comparison to dogs with other causes of acute diarrhoea. However, PA may be associated with more severe clinical disease, resulting in more intensive treatment.

**Disclosures**

No disclosures to report

## 79 ISCAID‐O‐7

### 79.1 ISCAID ‐ International Society for Companion Animal Infectious Diseases

#### 79.1.1 A series of 46 cases of Corynebacterium ulcerans infections in pets in France

##### 79.1.1.1 K. Museux ^1^, G. Arcari ^2^, G. Rodrigo ^1^, M. Hennart ^2^, E. Badell‐Ocando ^2^, J. Toubiana ^2^, S. Brisse ^2^

###### 79.1.1.1.1 ^1^ Laboratoire Cerba Vet Massy France; ^2^ Institut Pasteur Paris France

*Toxigenic Corynebacterium (C.) ulcerans* can cause diphtheria in human and is considered a zoonotic disease, pets being the potential source of transmission. Dogs and cats can be asymptomatic carriers of *C. ulcerans* but can also present clinical symptoms, as non‐healing wounds and chronic rhinitis. The typical clinical expressions of diphtheria in human are: (i) pseudo‐membranous diphtheric angina that can provoke a deadly obstruction of upper airways; (ii) cutaneous manifestations with ulcers often covered by a greyish pseudo‐membrane and (iii) toxigenic complications (polyneuropathy, myocarditis or hemorragic syndrome e.g). In France, emergence of *C. ulcerans* in human is noticed since 2003. We aimed to describe infections by *C.ulcerans* in pets from France over a period of 2 years (August 2019 – August 2021).

17 286 sick dogs and cats with rhinitis, dermatitis, non‐healing wounds and otitis were sampled for diagnostic purpose in metropolitan France. *C. ulcerans* identified by MALDI TOF were sent to the National Reference Center for confirmation and detection of the tox gene by real time PCR. Furthermore these isolates were analyzed by whole genome sequencing.

*C.ulcerans* was identified in 46 cases (18 cats and 28 dogs), including 20 toxigenic cases (9 cats and 11 dogs). Rhinitis was the most frequent presentation (14 of 46). 10 cases of the 46 cases were monoinfections pointing towards a primary pathogenic potential. Large breed dogs, especially German Shepherds were overrepresented (9 of 28 dogs), but mostly for non‐toxigenic infections (7 German Shepherds of 14 non‐toxigenic infections). The carriage rate was 0,27% (46 *C. ulcerans*/17 286 samples). Follow‐up of some toxigenic *C. ulcerans* infections showed a lack of antibiotic susceptibility despite an antibiogram suggesting susceptibility. The wide geographic distribution suggests a general phenomenon not related to a particular local source and proven by sequencing of the present *C. ulcerans*.

These cases revealed a lack of recommendations for treatment, management, and the importance of vaccination of veterinary clinic staff and owners in order to be protected from a potential exposure. Despite the low actual carriage rate, this study emphasizes the importance to address this One Health issue, considering the emergence of human indigenous cases and to explore a potential reservoir. These data could be used to develop guidelines for the veterinarian concerning toxigenic *Corynebacterium* infections. Recommendations concerning sampling, treatment, follow‐ups are suggested.

**Disclosures**

Disclosures to report, please report below

The authors declare a conflict of interest as 2 authors are employed as salaries at Cerba Vet, which performs diagnostic testing. A funding was received by French Government's Investissement d'Avenir program Laboratoire d'Excellence “Integrative Biology of Emerging Infectious Diseases” (ANR‐10‐LABX‐62‐IBEID).

## 80 ISCAID‐O‐8

### 80.1 ISCAID ‐ International Society for Companion Animal Infectious Diseases

#### 80.1.1 Serological prevalence and criteria for selection of dogs for screening of vector borne diseases using a point‐of‐care test in a referral population in the United Kingdom – multicentre retrospective study (2015 – 2020)

##### 80.1.1.1 A. Bello ^1^, E. Fairbanks ^2^, B. Glanemann ^3^, L. Hjalmarsson ^3^, S. Tappin ^4^, T. Gamston ^4^, C. Lee ^5^, D. Walker ^5^, N. Bexfield ^6^, V. Neale ^6^, P. Defauw ^7^, E. Doyle ^7^, H. Reyes‐Hughes ^8^, J. Reeve ^8^, P. Silvestrini ^9^, D. Thompson ^10^, L. Knowlden ^10^, C. Scudder ^11^, I. Calvo ^11^, F. Allerton ^12^, L. Bree ^12^, J. Cruzado‐Perez ^13^, V. Lamb ^13^, D. Gardner ^14^, A. Hrovat ^1^

###### 80.1.1.1.1 ^1^ Pride Veterinary Centre Derby United Kingdom; ^2^ Swiss Tropical and Public Health Institute Basel Switzerland; ^3^ Queen Mother Hospital for Animals, Royal Veterinary College Hatfield United Kingdom; ^4^ Dick White Referrals Cambridgeshire United Kingdom; ^5^ Anderson Moores Veterinary Specialists Winchester United Kingdom; ^6^ The Queen's Veterinary School Hospital, University of Cambridge Cambridge United Kingdom; ^7^ Lumbry Park Veterinary Specialists Alton United Kingdom; ^8^ Langford Vets, Small Animal Referral Hospital Langford United Kingdom; ^9^ School of Veterinary Science, University of Liverpool Liverpool United Kingdom; ^10^ Davies Veterinary Specialists Higham Gobion United Kingdom; ^11^ Southfields Veterinary Specialists Laindon United Kingdom; ^12^ Willows Veterinary Centre & Referral Service Shirley United Kingdom; ^13^ Southern Counties Veterinary Specialists Hangersley United Kingdom; ^14^ School of Veterinary Medicine and Science, University of Nottingham Nottingham United Kingdom

Growing pet travel and climate change have increased the threat of vector borne diseases (VBDs) in the UK. However, the prevalence of dogs testing positive remains low and there is uncertainty around criteria used to select patients for screening.

The aims of this retrospective study were to assess the prevalence and clinical signs of positive dogs tested with a point‐of‐care test (IDEXX SNAP 4DX Plus Test) in a referral population of dogs in the UK and to examine the rationale for selection of dogs for screening. To be included in the study, dogs had to be tested for VBDs using IDEXX SNAP 4DX Plus Test. Details of the history, rationale for requesting the test and final diagnosis had to be available as well.

Inclusion criteria were met by 4022 dogs; 2518 had not and 743 dogs had travelled outside of the UK. Travel history was unknown in 761 dogs.

IDEXX SNAP 4DX Plus Test was positive in 86 and negative in 3936 dogs. Among dogs that tested positive, 71 (82.5%) had travelled, 12 (14%) had not and 3 (3.5%) had unknown travel history. Therefore, 9.5% and 0.5% of dogs with and without a travel history respectively tested positive.

Barnard's test revealed a significant association between travel history and positive result for *Ehrlichia spp*. (p=0.0002), *Anaplasma spp*. (p=0.0143) and *Borrelia burgdorferi* (p=0.0002). *Ehrlichia spp* was documented in 55 dogs, 1 (1.8%) without and 54 (98.1%) with a travel history, *Anaplasma spp* in 22 dogs, 7 (31.8%) without and 15 (68.1%) with a travel history, and *Borrelia burgdorferi* in 9 dogs, 6 (66.6%) without and 3 (33.3%) with a travel history. No dogs without a travel history tested positive for *Dirofilaria immitis*.

Multivariate binary logistic regression showed that dogs with a travel history with hyperglobulinaemia, pancytopenia or bicytopenia (anaemia and thrombocytopenia) were significantly more likely to test positive. Tick exposure, spinal pain or pancytopenia significantly increased the probability of a dog without a travel history testing positive.

Pyrexia (19.1%), anaemia (18.7%), arthropathy (14.2%) and thrombocytopenia (12.4%) were cited as a rationale for requesting the test in 3936 of dogs that tested negative. Hyperglobulinemia (0.5%), pancytopenia (1.3%) and bicytopenia (2.8%) were less common motivations in dogs that tested negative.

Results from this large referral population of dogs in the UK showed that VBDs are uncommon in dogs without a travel history, but also highlighted a suboptimal selection of dogs for screening for VBDs.

**Disclosures**

No disclosures to report

## 81 ISCAID‐O‐9

### 81.1 ISCAID ‐ International Society for Companion Animal Infectious Diseases

#### 81.1.1 Immunologic factors in dogs with pulmonary and disseminated coccidioidomycosis: a pilot study

##### 81.1.1.1 J. Jaffey, L. Shubitz, A. Da Cunha, A. Murthy, C. Bolch, R. Monasky, I. Carswell, J. Spiker

###### 81.1.1.1.1 Midwestern University College of Veterinary Medicine Glendale United States

The immunologic factors associated with coccidioidomycosis and progression from pulmonary to disseminated disease in dogs is unknown. In addition, there are no reliable biomarkers that can predict extent of infection (i.e., pulmonary versus disseminated). Therefore, our primary objective was to compare constitutive plasma cytokine concentrations, leukocyte expression of toll‐like receptor (TLR)2 and TLR4, and serum c‐reactive protein (CRP) concentrations between healthy controls and dogs with coccidioidomycosis as well as between those with pulmonary and disseminated *Coccidioides* spp. infections. A secondary objective was to determine whether serum CRP concentration could predict extent of *Coccidioides* spp. infection. Dogs with a novel diagnosis of coccidioidomycosis were included and subsequently categorized as pulmonary or disseminated. Healthy, non‐immune, controls were also included. Serum CRP was measured with a commercially available ELISA. Constitutive plasma cytokines were measured with a canine‐specific multiplex bead‐based assay. Leukocyte expression of TLR2 and TLR4 was evaluated with flow cytometry. A two‐sample t‐test was used to compare outcomes between coccidioidomycosis vs. control dogs as well as between pulmonary vs. disseminated dogs. A ROC curve was used to determine utility of serum CRP to predict extent of *Coccidioides* spp. infection. Significance was set at P < 0.05.

Twenty‐eight dogs with coccidioidomycosis (pulmonary, n = 16; disseminated, n = 12) and 10 healthy controls were included. Average serum CRP concentration was higher in dogs with coccidioidomycosis than controls (P < 0.001). Similarly, pulmonary coccidioidomycosis dogs had higher average serum CRP than those with disseminated disease (P = 0.001). Serum CRP at a cutoff of > 10399 ng/mL was a fair predictor of pulmonary coccidioidomycosis in our cohort with a sensitivity of 88% (95% CI: 64‐98%) and specificity of 75% (95% CI: 47‐91%). There were no significant differences in average leukocyte expression of TLR2 or TLR4 between either groups of dogs (all P‐values > 0.05). Average plasma KC‐like concentration was higher in dogs with coccidioidomycosis than controls (P = 0.02). No other differences in the remaining 11 cytokines were identified (P‐values > 0.05). Serum CRP might have utility as a biomarker to distinguish extent of *Coccidioides* spp. infection. Future studies with larger populations are needed to further investigate the immunopathogenesis of coccidioidomycosis in dogs.

**Disclosures**

No disclosures to report

## 82 ISCAID‐O‐10

### 82.1 ISCAID ‐ International Society for Companion Animal Infectious Diseases

#### 82.1.1 The impact of a short, animated film on owner attitudes towards antimicrobial use in companion animals – a randomised controlled trial

##### 82.1.1.1 E. Wright ^1^, S Pfleger ^2^, I Gajanayake ^1^, T Gemmill ^1^, L Jessen ^3^, T M Soerensen ^3^, I Battersby ^4^, E West ^4^, L Atkinson ^4^, M Mosher ^5^, C Rutland ^6^, D Singleton ^7^, A Tompson ^8^, F Allerton ^1^

###### 82.1.1.1.1 ^1^ Willows Veterinary Centre and Referrals Service Solihull United Kingdom; ^2^ NHS Highland, Public Health Directorate Inverness United Kingdom; ^3^ University of Copenhagen Copenhagen Denmark; ^4^ Davies Veterinary Specialists Hitchin United Kingdom; ^5^ Mars Veterinary Health New York United States; ^6^ Simplyhealth and Denplan Andover United Kingdom; ^7^ University of Liverpool Liverpool United Kingdom; ^8^ London School of Hygiene and Tropical Medicine London United Kingdom

Antimicrobial resistance (AMR) is a major cause of death globally; the veterinary profession needs to promote the prudent use of antimicrobials to help preserve their efficacy. Veterinarians cite pressure from clients as a contributor to the decision to prescribe antimicrobials. No studies have assessed the impact of engaging pet owners in antimicrobial stewardship.

The objective was to create an informative animated film containing a targeted message about AMR and to evaluate its effect on owner attitudes towards antimicrobial use.

Ethical approval was obtained. The animation was iteratively designed using commercially available software (Vyond™). It was centred on a clinical scenario of a dog with acute diarrhoea and highlighted the seriousness of antimicrobial resistance, and the owner's role in antimicrobial stewardship. Key opinion leaders were involved in development of this resource.

A survey was created based on the health belief model incorporating six constructs that provide a measure of health‐related behaviour. This included 20 statements (7 pre and 13 post‐animation) answered on a 5‐point Likert scale (strongly disagree to strongly agree). Owners attending six UK veterinary centres were invited to scan a QR code leading to a secure webpage. The website randomised participants to an ‘animation’ and a ‘no animation’ group (ratio 1:1). Responses were compared by the Mann‐Whitney U test and statistical significance was set at p<0.05.

Six hundred and forty‐seven (647) owners participated in the study. Responses were excluded if they did not proceed past consent (n=91), took longer than 1 hour to complete the survey (n=11), did not watch the entire video (n=6) or did not complete the survey (n=189). There were 350 complete responses.

Responses to 11 of the 13 questions asked after the animation were significantly different between the ‘animation’ and ‘no animation’ groups (all p<0.05). There was no significant difference between groups in response to the seven statements asked before the animation.

The animation group were more likely to agree that lower antimicrobial use will help maintain future efficacy (p<0.001) and that requesting antimicrobials from their vet could increase unnecessary use (p<0.001). The animation group were more likely to disagree that they would expect antimicrobials if their pet had diarrhoea (p<0.048).

Owners that watched a short AMR engagement animation had greater awareness of the impact of AMR and were more likely to support measures in line with antimicrobial stewardship. This behavioural‐nudge resource could support owners towards contributing to a multi‐faceted approach to AMR.

**Disclosures**

No disclosures to report

## 83 ESVONC‐O‐1

### 83.1 ESVONC ‐ European Society of Veterinary Oncology

#### 83.1.1 Subcutaneous mast cell tumors revisited: a prospective clinical study on 50 dogs

##### 83.1.1.1 L. Marconato, D. Stefanello, F. Solari Basano , E. Faroni, M. Dacasto, M. Giantin, G. Bettini, L. Aresu, U. Bonfanti, W. Bertazzolo, E. Vasconi, M. Annoni, C. Lecchi, S. Sabattini

###### 83.1.1.1.1 University of Bologna Bologna Italy

Canine subcutaneous mast cell tumors (ScMCTs) have been historically associated with a good prognosis due to a low metastatic potential and a favorable outcome if treated with surgery alone, regardless of histologic margins.

A multi‐center prospective study was conducted to provide more insight into their biologic behavior. Dogs with a firstly‐occurring, single ScMCT were enrolled upon primary tumor removal and regional lymphadenectomy. Post‐operatively, dogs were allocated into: 1) Monitoring Group, if the regional lymph node (LN) was HN0‐HN2 without distant metastasis; 2) Vinblastine Group, in the presence of HN3 LN and/or distant metastasis with wild‐type c‐kit; 3) Toceranib Group, in the presence of HN3 LN and/or distant metastasis with mutated c‐kit.

Data were collected through a cloud‐based electronic data‐capture platform.

Fifty dogs were enrolled. One (2%) had visceral metastasis. Thirty‐seven (74%) ScMCTs were completely removed and 13 (26%) had incomplete margins (12 underwent scar re‐excision, 1 radiation therapy). Seven (14%) dogs had a mitotic count (MC) >4, 5 (10%) had Ki67>23 and 18 (36%) had KIT‐pattern 2/3. ITD (n=6) and deleterious missense (n=1) c‐kit mutations were detected in 7 (14%) ScMCTs.

Eighteen (36%) dogs had at least one HN3 LN, 16 (32%) had at least one HN2 LN. Twenty‐six (52%) dogs were included in the Monitoring Group, 18 (36%) in the Vinblastine Group and 6 (12%) in the Toceranib Group. Median follow‐up time was 708 days (range, 119‐1860). Eleven (22%) dogs experienced tumor progression (n=9 local, n=6 nodal, n=3 distant) after a median of 193 days (range, 15‐857). Five (10%) dogs in the medical treatment groups died of MCT‐related causes. One‐ and two‐year survival rates were 97% and 79%, respectively.

Variables significantly associated with an increased risk of progression included tumor diameter >3 cm (HR, 3.8), ulceration (HR, 9.2), substage b (HR, 8.1), ≥1 HN3 LNs (HR, 9.2), high cyto‐grade (HR, 5.6) and MC>4 (HR, 10.0). Variables associated with tumor‐related death were substage b (HR, 17.0), high cyto‐grade (HR, 15.5), MC>4 (HR, 39.9) and c‐kit exon 11 ITD (HR, 15.0). MC and cyto‐grade remained significant on multivariable analysis.

Dogs with adequately locally controlled ScMCTs had good long‐term prognosis. Metastatic rate at admission was higher than previously reported, with 68% of dogs having HN2/HN3 nodal metastasis and 22% experiencing disease progression despite multimodal treatment. MC confirms as a reliable prognostic indicator, suggesting that histologic grading might be useful for ScMCT, and cyto‐grading may anticipate a more aggressive biologic behavior as well.

**Disclosures**

No disclosures to report

## 84 ESVONC‐O‐2

### 84.1 ESVONC ‐ European Society of Veterinary Oncology

#### 84.1.1 High sensitivity and negative predictive value of methylene blue as a single technique to rule out sentinel lymph nodes: a prospective study on 25 dogs with low‐grade cutaneous mast cell tumors

##### 84.1.1.1 D. Guerra, S. Zanardi, V. Cola, A. Foglia, S. Del Magno, L. Pisoni, L. Ciammaichella, E. Faroni, S. Sabattini, C. Agnoli, A. Renzi, L. Marconato

###### 84.1.1.1.1 University of Bologna Bologna Italy

In oncology, the identification of sentinel lymph nodes (SLNs) aims at mapping lymphatic drainage and helps in staging the disease. In canine cutaneous mast cell tumors (cMCTSs), lymph node involvement is an important prognostic factor, and lymphadenectomy is not influenced by the positive or negative status of SLNs, as it has been recommended regardless. The gold standard technique for SLNs identification includes the combined use of preoperative imaging techniques and intraoperative blue dye; nevertheless, this is not always feasible in daily practice due to costs or equipment unavailability. In this scenario, the regional lymph nodes (RLNs) are removed; however, RLNs and SLNs do not match in up to 25% of cases.

Aims of this prospective study were to evaluate the sensitivity and negative predictive value (NPV) of methylene blue (MB) staining alone in the assessment of RLNs in dogs with low‐grade cMCTs, and to determine whether non dye‐capturing lymph nodes in the same lymphocentrum were histologically metastases‐free.

Dogs with cytologically low‐grade cMCTs amenable to curative surgery and no evidence of distant metastasis based on a complete staging were eligible for recruitment. RLNs were identified based on anatomic location.

A total volume of 0.4 ml of 1% MB was injected peritumorally, 0.1 ml at each cardinal point, using a 26 Gauge needle syringe. To reduce the risk of degranulation, no tumor massage was performed after dye injection.

All dogs underwent wide surgical excision of cMCT and lymphadenectomy of RLNs in the same lymphocentrum, both dye‐capturing and non dye‐capturing yet suspicious (anatomical proximity to dye‐capturing RLNs or having a lymphatic blue channel).

Resected tumors underwent histopathologic evaluation and only histologically‐confirmed low‐grade cMCTs were ultimately included. All RLNs were microscopically examined after hematoxylin‐eosin and toluidine blue staining and classified according to Weishaar.

Twenty‐five dogs were enrolled and 60 RLNs were excised. Thirty‐one (51.7%) RLNs were not dye‐capturing and 29 (48.3%) were dye‐capturing. Histologic examination of not dye‐capturing RLNs revealed that 30 (97%) were HN0/HN1 and one (3%) was HN2. None was overtly metastatic (HN3). No complications related to MB injection were recorded.

NPV of intraoperative MB staining in identifying RLNs as non‐metastatic was 96%; sensitivity was 94%.

Intraoperative MB is reliable and feasible in identifying non‐metastatic RLNs in dogs with low‐grade cMCT, and can be done in clinical settings with limited access to the supporting combination technique. Excision of not dye‐capturing RLNs may provide no benefit to this population of dogs.

**Disclosures**

No disclosures to report

## 85 ESVONC‐O‐3

### 85.1 ESVONC ‐ European Society of Veterinary Oncology

#### 85.1.1 Patterns of nodal metastases, biological behaviour and prognosis of canine mast cell tumours of the pinnae: a multi‐institutional retrospective study

##### 85.1.1.1 C. Chalfon, R. Finotello , S. Sabattini, I. Gramer , J. Morris, M. Aralla, M.E. Morello , E.I. Ferraris , S. Ramos, G. Polton , L. Schiavo, J. Dobson , V. Cola, L. Marconato

###### 85.1.1.1.1 University of Bologna Bologna Italy

In dogs, anatomic location of mast cell tumours (MCTs) may have prognostic relevance. It has been suggested that head and neck MCTs, including pinna, behave more aggressively. According to anatomical studies, the regional lymph nodes (RLNs) draining the canine pinna include the prescapular and the parotid.

The first aim of this retrospective study was to describe the frequency, location and histologic stage of nodal metastases among dogs with MCTs of the pinnae that underwent sentinel lymph node (SLN) mapping. The second aim was to evaluate prognosis of dogs with MCTs of the pinnae.

Medical records of eight institutions were retrospectively reviewed to identify dogs with completely staged, histologically‐confirmed MCTs of the pinnae, that underwent tumour and SLN or non‐SLN (i.e., RLN and/or anatomically nearest lymph node [LN]) excision, with/without adjuvant medical treatment. SLNs were identified by means of lympho‐CT. LNs were classified according to Weishaar's criteria. The influence of potential prognostic variables (e.g., stage, substage, tumour diameter, histologic grade, margins, LN HN status) on time to progression (TTP) and tumour‐specific survival (TSS) was investigated.

Thirty‐nine dogs were included in the analysis; 19 (48.7%) had Kiupel high‐grade (K‐HG) and 20 (51.3%) had Kiupel low‐grade (K‐LG) MCTs. Eighteen (46.1%) dogs underwent SLN mapping: the prescapular LN was one of/the only SLN in 17 (94.4%) cases. Overall, 27 SLNs (17 prescapular, 6 mandibular, 3 parotid, 1 medial retropharyngeal) and 31 non‐SLNs (18 mandibular, 11 prescapular, 2 medial retropharyngeal) were removed. Twenty‐two (56.4%) dogs had nodal metastases; among them, the prescapular LN was always involved (n=10 HN2, n=12 HN3). None had distant metastasis. All dogs with K‐HG and 6 (30%) dogs with K‐LG received adjuvant medical treatment.

On multivariable analysis, K‐HG (P=0.038) and substage b (P=0.032) were associated with increased risk of tumour progression; K‐HG was the only variable associated with increased risk of tumour‐related death (P=0.021).

Median TTP was significantly shorter for dogs with K‐HG compared to dogs with K‐LG MCTs (270 days [35‐505] vs not reached, respectively; P<0.001); median TSS was significantly shorter for dogs with K‐HG compared to dogs with K‐LG MCTs (370 days [280‐460] vs not reached, respectively; P<0.001).

Our results suggest that the prescapular LN is most often the SLN in MCTs of the pinnae; furthermore, it often harbours early or overt metastasis. Approximately 50% of MCTs of the pinnae are histologically high‐grade and have nodal metastasis at presentation. A multimodal treatment leading to adequate tumour control improves outcome.

**Disclosures**

No disclosures to report

## 86 ESVONC‐O‐4

### 86.1 ESVONC ‐ European Society of Veterinary Oncology

#### 86.1.1 A retrospective study on bone metastasis in dogs with advanced‐stage solid cancers

##### 86.1.1.1 A. Ubiali ^1^, C. Agnoli ^1^, S. Sabattini ^1^, E. Battisti ^1^, F. Rossi ^2^, A. Diana ^1^, M.T. Camerino ^3^, S. Perfetti ^1^, L. Ciammaichella ^1^, D. Stefanello ^4^, M. Papa ^5^, R. Zaccone ^1^, L. Marconato ^1^

###### 86.1.1.1.1 ^1^ Alma Mater Studiorum University of Bologna Ozzano dell'Emilia BO Italy; ^2^ Clinica Veterinaria dell'Orologio Sasso Marconi (BO) Italy; ^3^ University of Torino Grugliasco TO Italy; ^4^ University of Milan Lodi (LO) Italy; ^5^ Clinica Veterinaria Gran Sasso Milano (MI) Italy

Bones are one of the most common metastatic sites for solid malignancies. As anticancer management has improved overall survival, bone metastases (BMs) have become an emerging problem, negatively impacting quality of life and survival. In dogs, research focusing on BMs is mainly limited to necropsy studies.

We completed a retrospective multicentric survey in dogs with solid cancer bearing BMs to investigate the potential impact of several clinical–pathological features and the therapeutic strategies on survival. BMs were diagnosed according to various imaging techniques and confirmed by cytology or histology.

Osteosarcoma cases were excluded.

In total, 44 bone‐metastatic dogs were identified. BMs were synchronous in 17 (38.6%) dogs and metachronous in 27 (61.4%). In the latter, the median latency time from primary cancer diagnosis to BMs was 160 days (range, 30‐1835).

The most common primary cancer locations included mammary gland (n=6), spleen (n=5), liver (n=4), prostate (n=4) and tonsil (n=4), whereas the most common histotypes were carcinoma (n=28) and hemangiosarcoma (n=9).

At the time of diagnosis, 28 (63.6%) dogs had solitary BM and 16 (36.4%) had multiple‐bone metastatic disease. By considering all affected bones, the most common site was the humerus (n=22), followed by spine (n=8), ribs (n=8), femur (n=8), pelvis (n=8), scapula (n=4), radius (n=4), ulna (n=4), tibia (n=3), sternum (n=1) and mandible (n=1).

After BMs diagnosis, 12 (27.3%) dogs received palliative treatment and 21 (47.7%) antitumoral therapy, including chemotherapy/toceranib (n=12), radiation therapy (n=5) and surgery (n=4). Eleven (25%) dogs received no treatment.

Overall median survival after BMs diagnosis was 30 days (range, 11‐49; 95% CI, 0‐44); 84% of dogs died because of skeletal‐related events, including pathologic fracture, nerve root/ spinal cord compression and intractable pain.

Based on univariable analysis, lack of antitumoral treatment (HR: 2.42 [95% CI, 1.27‐4.61]; P=0.007) was the only negative prognostic factor. A tendency to a worse outcome was observed for synchronous metastases (HR: 1.89 [95% CI, 0.99‐3.61]; P=0.054). BM location, primary cancer site, solitary/multiple BMs, pathologic fracture, concurrent extraskeletal metastases, ALP and LDH increase did not impact outcome. No bone‐specific metastatic pattern was identified; however, splenic and hepatic tumors tended to spread more frequently to the humerus.

BMs in dogs with solid cancers are associated with poor prognosis and high risk of skeletal‐related events. Latency time and treatment of BMs appear to have an impact on survival.

**Disclosures**

No disclosures to report

## 87 ESVONC‐O‐5

### 87.1 ESVONC ‐ European Society of Veterinary Oncology

#### 87.1.1 Timely adjuvant chemotherapy improves outcome in dogs with non‐metastatic splenic hemangiosarcoma undergoing splenectomy

##### 87.1.1.1 E. Faroni ^1^, S. Sabattini ^1^, D. Guerra ^1^, C. Iannuzzi ^1^, C. Chalfon ^1^, C. Agnoli ^1^, D. Stefanello ^2^, G. Polton ^3^, S. Ramos ^3^, M. Aralla ^4^, R. Ciaccini ^5^, A. Foglia ^1^, S. Okonji ^1^, L. Marconato ^1^

###### 87.1.1.1.1 ^1^ University of Bologna Ozzano dell'Emilia (BO) Italy; ^2^ University of Milan Lodi Italy; ^3^ North Downs Specialist Referrals Bletchingley United Kingdom; ^4^ Pronto Soccorso Veterinario Laudense Lodi Italy; ^5^ Clinica Veterinaria Ponte Felcino Perugia Italy

Recruitment of resting metastatic cells into the cell cycle after primary tumor removal is a well‐known phenomenon. Timely delivery of adjuvant chemotherapy has been shown to be advantageous in human cancers and canine osteosarcoma. Canine splenic hemangiosarcoma is an aggressive malignancy, for which surgery cannot be considered curative; thus, adjuvant chemotherapy is indicated.

Aim of this retrospective study was to investigate whether timely adjuvant chemotherapy administration resulted in longer time to progression (TTP) and tumor‐specific survival (TSS) in dogs with non‐metastatic splenic hemangiosarcoma undergoing splenectomy.

Medical records of seven oncology centers were searched for dogs with completely staged, non‐metastatic, splenic hemangiosarcoma, that received splenectomy and adjuvant doxorubicin‐based chemotherapy, and for which follow‐up was available. The number of days from surgery to the first chemotherapy dose (StoC) was retrieved and evaluated to identify the cut‐off value associated with the best survival advantage. Possible prognostic factors (e.g., stage, anemia, thrombocytopenia, coagulation abnormalities, blood products administration, chemotherapy protocol, rescue chemotherapy) and StoC were tested for influence on TTP and TSS.

Seventy dogs were included. Median StoC was 20 days (range, 4‐70). The time interval associated with the best survival benefit was 21 days. Dogs with StoC ≤21 days and those with StoC >21 days were well balanced in terms of the aforementioned prognostic factors. StoC >21 days was the only variable significantly associated with increased risk of tumor progression (HR 2.1, 95%CI 1.2‐3.6; P=0.010) and tumor‐related death (HR 2.3, 95%CI 1.3‐4.2; P=0.008) on multivariable analysis. Median TTP and TSS of dogs with StoC ≤21 days (195 days, 95%CI 118‐272, and 255 days, 95%CI 198‐312, respectively) were significantly longer than median TTP and TSS of those with StoC >21 days (144 days, 95%CI 122‐166, P=0.003, and 185 days, 95%CI 149‐221, P=0.001, respectively). Likewise, 1‐year survival rate was significantly higher in dogs with StoC ≤21 days compared to those with StoC >21 days (15% and 0%, respectively, P=0.03).

The importance of timely administration of adjuvant chemotherapy has been advocated before. According to our study, starting adjuvant chemotherapy within 21 days post‐surgery may be associated with a significant survival benefit in dogs with non‐metastatic splenic hemangiosarcoma, possibly due to the early targeting of newly‐recruited metastatic cells after surgery. In addition, 21 days would usually be enough for the dog to recover from surgery and for the clinician to receive a definitive histopathologic diagnosis. Prospective trials are needed to assess whether shorter StoC further improves prognosis.

**Disclosures**

No disclosures to report

## 88 ESVONC‐O‐6

### 88.1 ESVONC ‐ European Society of Veterinary Oncology

#### 88.1.1 benefit for capecitabine in dogs with biologically aggressive carcinoma of various origin: a single institution experience

##### 88.1.1.1 C. Agnoli, D. Guerra, S. Rimondi, G. Ghisoni, S. Perfetti, A. Tirolo, C. Selva Coddè, L. Marconato

###### 88.1.1.1.1 Unibo Ozzano dell'Emilia Bologna Italy

Capecitabine is an orally administered prodrug that is converted by three enzymatic reactions to 5‐fluorouracil. In people, capecitabine is indicated for the treatment of various malignant epithelial cancers, including metastatic breast and colorectal carcinoma. In dogs, capecitabine has not been extensively studied. The aim of this retrospective study was to investigate preliminary efficacy and toxicity of single‐agent capecitabine in dogs with biologically aggressive malignant epithelial cancers.

Dogs with unresectable, metastatic, or histologically aggressive carcinoma of any anatomic origin, for which standard treatment options were not available or declined by the owner, were included. Capecitabine was administered orally at 750 mg/m^2^ on days 1‐14, followed by 1‐week rest period, given as 3‐week cycles. Blood analysis was performed on day 1 of each cycle. Compliance for administration of capecitabine was monitored by questioning owners at each visit. In dogs with macroscopic disease, response was assessed every 2 cycles by follow‐up imaging studies and/or lesion cytology based on RECIST criteria and improvement of symptoms. Clinical benefit (CB) was defined as the percentage of dogs achieving complete response, partial response (PR) and stable disease. Progression‐free interval (PFI) was defined as the interval between the first day of capecitabine to the documentation of disease progression. Toxicity was graded according to VCOG‐CTCAE.

Fifteen dogs with hepatocellular (n=4), colic (n=3), anal sac (n=3), colorectal (n=1), exocrine pancreatic (n=1), mammary (n=1), ear canal (n=1) carcinoma and 1 carcinoma of unknown primary were included. Previous treatments consisted of surgery (n=11) and chemotherapy (n=8). At the start of capecitabine treatment, 13 dogs had macroscopic disease (3 unresectable, 4 unresectable and metastatic, 6 metastatic), and two had a microscopically reduced, histologically aggressive carcinoma. These last two were excluded from response analysis. All dogs were evaluable for toxicity. Median daily dose of capecitabine was 719 mg (range, 625‐817). Dogs received a median of 8 cycles (range, 3‐21). Three (23%) dogs achieved PR, 8 (62%) were stable and 2 (15%) progressed, for an overall CB of 85%. Overall PFI was 91 days (range, 42‐154). Toxicity occurred in 7 (46.6%) dogs and consisted of grade 1 (n=2) and grade 2 (n=3) gastrointestinal side effects, and grade 1 (n=2) neutropenia.

Single‐agent capecitabine is moderately active and well‐tolerated in dogs with advanced, metastatic or biologically aggressive carcinoma. Additionally, compared with intravenous chemotherapy, oral administration is convenient, requires fewer healthcare resources and is generally preferred by owners. Prospective studies are needed to confirm our findings.

**Disclosures**

No disclosures to report

## 89 ESVONC‐O‐7

### 89.1 ESVONC ‐ European Society of Veterinary Oncology

#### 89.1.1 Transcriptomic analysis of canine B and T cell lymphoma identifies differential gene expression

##### 89.1.1.1 M. Lopes, P. Capewell, E. Waugh, W. Weir, J. Morris

###### 89.1.1.1.1 Small Animal Hospital University of Glasgow Glasgow United Kingdom

Gene expression profiling in human lymphoma has identified genes and pathways with prognostic importance that could also be used as targets for therapy.

The aim of this study was to compare the gene expression of canine B‐cell lymphoma, T‐cell lymphoma and control lymph node samples using RNA‐Seq to identify transcriptional signatures which may have a role in pathogenesis.

RNA was extracted from lymph node tissue collected from six dogs with B‐cell lymphoma, four dogs with T‐cell lymphoma, and eight controls. The RNA was sequenced using an Illumina NextSeq500 platform, aligned to the current canine genome using STAR, and read counts analysed using DESeq2 to identify differentially expressed genes. These data were further analysed using the weighted gene set analysis tool of Reactome to highlight enriched pathways and a random forest machine learning algorithm was implemented using Caret to identify potentially diagnostic transcripts in the cohort.

Principal component analysis (PCA) revealed that the three groups clustered separately. Pathway analysis highlighted different pathways overrepresented in B‐cell versus T‐cell lymphoma: cell cycle control and mRNA synthesis, and innate immunity and cell proliferation, respectively. Additionally, the machine learning algorithm identified seven genes that could reliably differentiate between B‐cell and T‐cell lymphoma: RSPH1, CDK18, SPTB and XRRA1 were significantly overexpressed in T‐cell lymphoma and MTPN, CMPK1 and HTRA2 were significantly overexpressed in B‐cell lymphoma.

We have identified several genes as putative markers of B‐cell and T‐cell lymphoma that have not been reported before together with pathways which could be used as possible treatment targets.

**Disclosures**

No disclosures to report

## 90 ESVONC‐O‐8

### 90.1 ESVONC ‐ European Society of Veterinary Oncology

#### 90.1.1 Experiences from the first ten canine oral cancer patients treated with FLASH radiotherapy

##### 90.1.1.1 B. Børresen, M.L. Arendt, E. Konradsson, K. Bastholm Jensen, C. Ceberg, K. Petersson

###### 90.1.1.1.1 University of Copenhagen Frederiksberg Denmark

FLASH radiotherapy (RT) is a novel technique for delivering radiotherapy at an ultra‐high dose rate in one or a few fractions. Clinical data describing efficacy and toxicity is sparse, but it suggests that FLASH RT may have fewer adverse effects than conventional radiotherapy. Oral tumors in dogs are commonly treated with conventional radiotherapy, but toxicity to the oral mucosa may be severe and dose‐limiting. Whether the sparing effect of FLASH RT is also observed for oral tumors is currently unknown. Accordingly, the efficacy and toxicity of FLASH RT for treating canine oral tumors were investigated in the current study.

Canine cancer patients with spontaneous malignant oral tumors were enrolled if the responsible veterinary oncologist considered radiotherapy to be indicated or if owners decided against other treatment options. A single dose of FLASH RT was planned. Tumor size and adverse effects were documented on the treatment day, day 7 and 30 and 3, 6 and 12 months post therapy and graded based on RECIST (v1.0) and VRTOG criteria.

Ten dogs were included. These were diagnosed with malignant melanoma (n=4), squamous cell carcinoma (n=3), fibrosarcoma (n=2) and mast cell tumor (n=1). The maximum dose/fraction ranged from 30 to 42Gy. Two dogs were retreated in the same region.

The majority of adverse effects were grade 1 and mainly characterized by mild mucositis, however five dogs developed a grade 3 toxicity one to six months post treatment. These ranged from self‐limiting ulcerations to necrotic defects affecting soft tissue and underlying bone. Tumor responses ranged from complete response to short or long‐term stable disease. One dog died of unknown causes two weeks following treatment, precluding response assessment.

Dogs with oral tumors treated with FLASH RT in this study generally experienced some clinical benefit. Of the nine dogs with at least one month follow‐up post treatment, four experienced no or only minor toxicities, and five experienced high‐grade adverse effects. This differs from the low frequency of adverse effects previously observed for FLASH RT treatments of other body sites and suggests that the high end of the prescribed dose range is approaching the maximum tolerated dose. This is important to consider, when planning future FLASH RT treatments of the oral cavity.

**Disclosures**

No disclosures to report

## 91 ESVE‐O‐1

### 91.1 ESVE ‐ European Society of Veterinary Endocrinology

#### 91.1.1 Whole transcriptome analysis of canine pheochromocytoma and paraganglioma

##### 91.1.1.1 M. Berg, K. Sanders, H.S. Kooistra, G.C.M. Grinwis, M.M.J.M. Zandvliet, F. Steenbeek, S. Galac

###### 91.1.1.1.1 Faculty of Veterinary Medicine, Utrecht University Utrecht Netherlands

Pheochromocytomas and paragangliomas (PPGLs) are neuroendocrine tumors arising from the chromaffin cells in the adrenal medulla and extra‐adrenal paraganglia, respectively. Local invasion, concurrent disorders, and in rare occasions metastases, prevent surgical removal, which is the most effective treatment to date. Given the current lack of effective medical treatment, there is a need for novel therapeutic strategies. To identify druggable pathways driving PPGL development, we performed RNA sequencing on PPGLs and normal adrenal medullary tissue of dogs.

Tumor tissue was obtained from client‐owned dogs with PPGL (n=19; 18 pheochromocytomas, 1 paraganglioma) following surgical removal or immediately after euthanasia. Normal adrenal medullas (NAMs) (n=10) were obtained from healthy dogs euthanized for reasons unrelated to the present study. The diagnosis of PPGL was confirmed by histopathology and immunohistochemistry using the adrenomedullary markers synaptophysin and chromogranin A. Tissue was snap‐frozen in liquid nitrogen or fixed in RNA*later* stabilization solution and stored at ‐80°C until RNA extraction. Whole transcriptome sequencing was performed to determine the mRNA expression levels of all active genes in PPGL and NAM. The Mann–Whitney *U*‐test was used to calculate P‐values, which were corrected for multiple comparisons using the false discovery rate method. Genes were considered differentially expressed when the corrected P‐value was <0.01.

Principal component analysis revealed that PPGLs clearly clustered apart from NAMs. In total, 2,547 genes were differentially expressed between PPGLs and NAMs. Of these, 177 had a log_2_ fold change of >3 or <‐3, of which 94 were upregulated in PPGLs, and 83 were downregulated. Enrichment analysis revealed that differentially expressed genes were associated with biological processes involving pro‐ and anti‐tumorigenic factors (e.g., *KIF11, FAP, GJA1, ERBB4*), tumor progression and metastasis (e.g., *POSTN, FOXI3, IQGAP3, TIAM2, PRL‐3*), angiogenesis (e.g., *ANGPTL7, ESM1*), the cell cycle (e.g., *PCLAF, CCNB2, CDC6, BUB1, TOP2A*), the Wnt signaling pathway (e.g., *WNT3, NKD1*), the hypoxia pathway (e.g., *CDCP1*), and formation of human PPGL (e.g., *RET*).

This study has shed light on the transcriptomic profile of canine PPGL, which will be instrumental in understanding the pathogenesis of PPGL in dogs, and revealed novel targets for therapy.

**Disclosures**

No disclosures to report

## 92 ESVE‐O‐2

### 92.1 ESVE ‐ European Society of Veterinary Endocrinology

#### 92.1.1 Plasma and Urinary Metanephrine and Normetanephrine in Healthy Cats – a Pilot Study

##### 92.1.1.1 M.J. Dias ^1^, M.T. Prego ^2^, R.L. Ferreira ^2^, S. Goncalves ^3^, T.D. Domingues ^4^, G. Junius ^5^, E. Steen ^5^, S. Galac ^6^, R. Leal ^1^

###### 92.1.1.1.1 ^1^ Centre for Interdisciplinary Research in Animal Health, Fac.Vet.Med., ULisboa Lisbon Portugal; ^2^ Veterinary Teaching Hospital, Fac. Vet. Med., ULisboa Lisbon Portugal; ^3^ Laboratório de Análises Clínicas Prof. M.Braço Forte, Fac.Vet.Med, ULisboa Lisboa Portugal; ^4^ Department of Statistics and Operations Research ‐ Faculty of Sciences, U.Lisboa Lisbon Portugal; ^5^ AML – Algemeen Medisch Laboratorium Antwerp Belgium; ^6^ Department of Clinical Sciences – Utrecht University Utrecht Netherlands

Feline pheochromocytoma is considered to be rare and literature is limited to few case‐reports. In humans and dogs, biochemical diagnosis of a pheochromocytoma is based on measurements of plasma (PL) and/or urinary (U) metanephrine (MN) and normetanephrine (NMN) but little is known about these biomarkers in cats.

This pilot study aims to evaluate the feasibility of PL‐MN/NMN and U‐MN/NMN measurement in cats, using Liquid Chromatography with tandem mass spectrometry (LC‐MS‐MS). Furthermore, it intends to assess the U‐MN/NMN stability under refrigeration (+4°C) for 24h.

A cross‐sectional pilot study was conducted, using a group of healthy adult cats recruited among students and staff from a Veterinary Teaching Hospital. The study was approved by the local ethical committee. With owner's consent, all cats were submitted to a physical examination, complete bloodwork, urine analysis, abdominal ultrasound, and systolic blood pressure assessment. Cats were excluded in case of abnormal results were identified. After sampling, EDTA‐plasma and urine were stored at ‐80°C until measurement of PL‐MN/NMN and U‐MN/NMN. U‐MN/Creatinine and U‐NMN/Creatinine ratios were then calculated. For each cat, an additional urine sample was refrigerated (UR) for 24h before storage at ‐80°C. Leftover plasma and urine samples collected from a cat with a confirmed diagnosis of pheochromocytoma (PheoCat) were also submitted for analyses. Non‐parametric tests were used for data analysis.

A total of 10 healthy cats were recruited, with a median age of 6 years (IQR=4.5). The PL‐MN and PL‐NMN median values were 2.73nmol/L (IQR=2.37) and 7.02nmol/L (IQR=5.2), respectively. U‐MN/Creatinine and U‐NMN/Creatinine ratios had medians of 70μg/g (IQR=70) and 139μg/g (IQR=77), respectively. Results obtained from the PheoCat revealed a PL‐MN of 3.68nmol/L, PL‐NMN of 66.27nmol/L, U‐MN/Creatinine ratio of 179 μg/g and U‐NMN/Creatinine ratio of 1262 μg/g. None of these values overlapped with the medians obtained from healthy cats.

The U‐MN/NMN proved to be stable under refrigeration for 24h, as there was no statistical difference between U‐MN vs UR‐MN and U‐NMN vs UR‐NMN (p= 0.329 and p= 0.813, respectively).

This pilot study supports the feasibility of PL‐MN/NMN and U‐MN/NMN measurement in cats and urinary stability after 24h stored under refrigeration. The PheoCat had a substantial increase of all the measured parameters (particularly PL‐ and U‐NMN) when compared to the healthy cats, highlighting the clinical applicability of these findings. This is the first study reporting both PL‐MN/NMN and U‐MN/NMN measurements by LC‐MS‐MS in adult healthy cats and will contribute to the biochemical diagnosis of feline pheochromocytoma in the future.

**Disclosures**

Disclosures to report, please report below

No conflict of interest to declare. This work was supported by FCT – Fundação para a Ciência e Tecnologia IP, grant UIDB/00276/2020

## 93 ESVE‐O‐3

### 93.1 ESVE ‐ European Society of Veterinary Endocrinology

#### 93.1.1 Correlation between plasma renin activity determined by the traditional angiotensin I formation method and by a novel renin‐angiotensin‐system marker

##### 93.1.1.1 M Kurtz ^1^, A Chamagne ^2^, C Maurey ^2^, O Domenig ^3^, G Benchekroun ^2^

###### 93.1.1.1.1 ^1^ Ecole Nationale Vétérinaire d'Alfort Maisons Alfort France; ^2^ Ecole Nationale Vétérinaire d'Alfort MAISONS ALFORT France; ^3^ Attoquant Diagnostics Vienna Austria

Diagnosis of primary hyperaldosteronism (PHA) in cats is ideally based on the evidence of suppressed plasma renin activity (PRA) with increased aldosterone concentration in an evocative clinical context. Current laboratory methods for the determination of PRA rely on the evaluation of angiotensin I (Ang1) formation and are time‐consuming, not widely available and prone to analytical variability. Whole renin‐angiotensin‐aldosterone‐system (RAAS) evaluation was recently developed in human medicine. It is based on a mass‐spectrometry assay for simultaneous quantification of angiotensin I, angiotensin II and aldosterone in serum. Results are, in part, used to determine a marker of renin activity: PRA‐S (calculated as Ang1 + Ang2). Recently, the same panel has been described in cats.

The aims of our study were to evaluate the correlation between PRA determined by the traditional Ang1 formation method and PRA‐S. We hypothesized that the two methods would lead to well‐correlated results.

Leftover samples from another prospective study were used. Healthy cats and cats presenting diseases suspected to affect the RAAS (RAASd) were included. All blood specimen were collected by a single operator with a standardized sampling protocol. For the determination of PRA by the Ang1 formation method, EDTA plasma samples were mixed with an Ang1 stabilizing mix, aliquoted and incubated on ice and at 37°C respectively for one hour, before Ang1 concentration was measured by ultraperformance liquid chromatography and tandem mass spectrometry (UPLC‐MS/MS). PRA was then calculated as the rate of Ang1 formation as determined by the difference in Ang1 concentration between the two aliquots. For the measurement of PRA‐S, equilibrium levels of Ang1 and Ang2 concentrations were quantified in serum samples by LC‐MS/MS. Correlation between the results was evaluated through the Spearman correlation test. Variables are presented as medians.

Sixty‐one cats were included. There was a positive correlation between PRA and PRA‐S (r(59)=0.80, p<0.001). RAASd included CKD (hypertensive or non‐hypertensive), hyperthyroidism, idiopathic hypertension, and PHA. Median PRA (pmol/L/h) and PRA‐S (pmol/L) values were 300 [213‐832] and 167 [123‐282] in healthy cats, 585 [200‐2450] and 149 [88‐482] in cats with RAASd.

These results suggest that PRA and PRA‐S show substantial correlation in cats, and that PRA‐S could possibly be used as a surrogate marker for PRA in the diagnosis of RAAS disorders in cats. Reference intervals and decisions cut‐offs have yet to be determined for both parameters, as well as influence of pre‐analytical parameters.

**Disclosures**

Disclosures to report, please report below

Residency program of M. Kurtz is getting financial support by Royal Canin.

## 94 ESVE‐O‐4

### 94.1 ESVE ‐ European Society of Veterinary Endocrinology

#### 94.1.1 Retrospective multicentre study of the medical and surgical management of feline primary hyperaldosteronism

##### 94.1.1.1 I. Calvo ^1^, V. Fabrès ^2^, F. Allerton ^3^, M. Kurtz ^4^, L. Bree ^3^, E. Krafft ^5^, A. . Drut ^6^, R. Dumont ^7^, F. Jolivet ^8^, F.Y. Foo ^9^, A. Watson ^10^, H. Syme ^10^, F. Fracassi ^11^, E. Vangrinsven ^12^, J. Bazelle ^13^, M. Rodríguez Piñeiro ^14^, R. Leal ^15^, G. Ruiz ^16^, P. Silvestrini ^17^, J. Pena‐Ramos ^18^, A. Hrovat ^19^, F. Zeugswetter ^20^, V. Neale ^21^, F. Tee ^22^, P. Valiente ^22^, M. Nolff ^23^, N. Sieber‐Ruckstuhl ^23^, S. . Caulfield ^24^, S. . Keyte ^24^, J. Rieder ^25^, H. Volk ^25^, R. Heilmann ^26^, A. Schreiber ^26^, A. Champetier ^27^, O. Dossin ^27^, M. Cervone ^28^, G. Benchekroun ^4^, C.J. Scudder ^1^

###### 94.1.1.1.1 ^1^ Southfields Veterinary Specialists Laindon United Kingdom; ^2^ CHV Anicura‐Aquivet Eysines France; ^3^ Willows Veterinary Centre and Referral Service Shirley United Kingdom; ^4^ Ecole Nationale Vétérinaire d'Alfort Maisons Alfort France; ^5^ Université de Lyon Lyon France; ^6^ Nantes‐Atlantic College of Veterinary Medicine and Food Sciences Nantes France; ^7^ CHV Frégis Arcueil France; ^8^ Centre Hospitalier Vétérinaire Languedocia Montpellier France; ^9^ University of Cambridge Cambridge United Kingdom; ^10^ Royal Veterinary College London United Kingdom; ^11^ University of Bologna Ozzano dell'Emilia Italy; ^12^ University of Liège Liège Belgium; ^13^ Davies Veterinary Specialists Hitchin United Kingdom; ^14^ Hospital Veterinario Puchol Madrid Spain; ^15^ University of Lisbon Lisbon Portugal; ^16^ Highcroft Veterinary Referrals Bridgwater United Kingdom; ^17^ University of Pennsylvania Philadelphia United States; ^18^ Langford Vets Langford United Kingdom; ^19^ Pride Veterinary Centre Derby United Kingdom; ^20^ University of Veterinary Medicine Vienna Austria; ^21^ Anderson Moores veterinary Specialists Winchester United Kingdom; ^22^ North Downs Specialist Referrals Redhill United Kingdom; ^23^ University of Zürich Zürich Switzerland; ^24^ Lumbry Park Veterinary Specialists Hampshire United Kingdom; ^25^ University of Veterinary Medicine Hannover Germany; ^26^ University of Leipzig Leipzig Germany; ^27^ Advetia Centre Hospitalier Vétérinaire Vélizy‐Villacoublay France; ^28^ Clinique Veterinaire Evolia L'Isle‐Adam France

Feline primary hyperaldosteronism (PHA) is caused by excessive aldosterone secretion by adrenal hyperplasia or tumour. The study aimed to describe survival of cats managed medically or surgically.

Cases between 2010 and 2022 were retrospectively recruited and allocated into groups determined by absence (group A) or presence (group B) of factors, such as concurrent disease, which could affect endocrine results. Log‐rank tests and factors with p<0.2 on univariate Cox analysis were used in multivariate analysis to assess survival.

Twenty‐eight referral centres reported 149 cases and 127 met inclusion criteria. Treatment included medical management only in 77 (61%), adrenalectomy in 42 (33%) and none in 8 (6%) cases. Left adrenalectomy was performed in 21 (50%), right in 20 (48%) and not recorded in one case. Cavotomy was performed in 3/39 (8%), 8/38 (21%) received peri‐operative glucocorticoids and 1/38 (3%) cats received peri‐operative mineralocorticoids. Eleven of 42 (26%) cats died within 14 days of adrenalectomy.

Diabetes mellitus (HR 2.51, 95% CI 1.02–6.16, p=0.045), medical management only (HR 2.93, 95% CI 0.1.36‐6.31, p=0.006) and adrenal size (HR 1.04, 95% CI 1.01–1.06, p=0006) were associated with decreased survival in multivariate analysis. The median survival of surgical and medical treatment was 780 [range 2 to 1248] and 694 [range 0 ‐ 2113] days, respectively (p=0.031). There was difference of survival between groups A and B (median 737 and 675 days, p=0.837).

Cats with PHA undergoing adrenalectomy had longer median survival but high peri‐operative mortality. Medical management was associated with long‐term survival in many cats.

**Disclosures**

No disclosures to report

There are no disclosures to declare. However, we would like to clarify that five cases are going to be published as part of a detailed pathological review. This information has not been covered in this abstract

## 95 ESVE‐O‐5

### 95.1 ESVE ‐ European Society of Veterinary Endocrinology

#### 95.1.1 Prospective evaluation of the telmisartan suppression test as a diagnostic tool for primary hyperaldosteronism in cats

##### 95.1.1.1 M Kurtz ^1^, V Fabrès ^2^, R Dumont ^3^, A Chamagne ^4^, V Chetboul ^4^, V Saponaro ^4^, E Trehiou ^4^, C Poissonnier ^4^, P Passavin ^4^, S Chahory , C Jondeau ^4^, M Bott ^4^, T Buronfosse ^5^, O Domenig ^6^, G Benchekroun ^4^

###### 95.1.1.1.1 ^1^ Ecole Nationale Vétérinaire d'Alfort Maisons Alfort France; ^2^ CHV Aquivet Eysines France; ^3^ CHV Frégis Arcueil‐Cachan France; ^4^ Ecole Nationale Vétérinaire d'Alfort MAISONS ALFORT France; ^5^ VetAgroSup Campus Vétérinaire Marcy L'Etoile France; ^6^ Attoquant Diagnostics Vienna Austria

Diagnosis of primary hyperaldosteronism (PHA) in cats frequently requires measurement of plasma renin activity, which is not widely available. The aim of the present study was therefore to further investigate the use of a previously described telmisartan suppression test (TST) as an accessible diagnostic test for PHA.

Thirty‐eight cats were prospectively recruited as follows: 1/healthy middle‐aged cats (group 1; n=6); 2/acute or chronic kidney disease (group 2; n=16) with (n=9) or without (n=7) systemic hypertension; 3/hyperthyroidism (group 3; n=9); 4/PHA (group 4; n=5); 5/idiopathic systemic hypertension (group 5; n=2). The TST was performed as previously described (measurement of serum aldosterone concentration at T0, T1h and T1.5h after 2 mg/kg of oral telmisartan administration). Variables were compared using Kruskal‐Wallis and Mann‐Whitney tests, and are presented as medians [interquartile range].

The TST was well tolerated and did not induce hypotension or hyperkalemia in any cat. No significant difference in maximal percentage of aldosterone variation was observed between groups (29 [5‐78]; 5 [(‐27)‐75]; 10 [(‐6)‐95]; 25 [0‐30]; 53 [19‐86] for groups 1, 2, 3, 4, and 5, respectively; p=0.66). Basal serum aldosterone concentration (ng/mL) was significantly higher in group 4 (105 [101‐166] than in group 1 (4.9 [4.6‐8.2], p=0.048) and group 2 (9.1 [6.0‐23.5], p<0.01).

In conclusion, the TST did not discriminate cats with PHA from healthy middle‐aged cats or cats with secondary hyperaldosteronism. These results do not encourage using the TST as a diagnostic tool for detecting PHA, although the small study population may be responsible for a lack of statistical power.

**Disclosures**

Disclosures to report, please report below

Financial support of residency program of M. Kurtz by Royal Canin

## 96 ESVE‐O‐6

### 96.1 ESVE ‐ European Society of Veterinary Endocrinology

#### 96.1.1 Clinical features and long‐term management with DOCP and prednisolone of cats with primary hypoadrenocorticism

##### 96.1.1.1 N. Sieber‐Ruckstuhl, L. Harburger, N. Hofer, C. Kümmerle, C. Müller, M. Stirn, B. Riond, C. Reusch, F. Boretti

###### 96.1.1.1.1 Clinic for Small Animal Internal Medicine Zurich Switzerland

Primary hypoadrenocorticism is rare in cats and knowledge about optimal treatment is sparse. The aim of this study was to describe cats with hypoadrenocorticism with a focus on DOCP and glucocorticoid therapy.

Records between 2010 and 2021 were retrospectively reviewed and 11 cats included. Data are presented as range (median).

Common clinical signs were reduced appetite/anorexia (n=11), reduced general condition (11), lethargy (10), dehydration (9), obstipation (7), weakness (7), weight loss (6) and hypothermia (5). Two cats were euthanized after 0.5 and 2 months. One cat was lost to follow‐up.

Eight DOCP‐treated cats were followed for 13‐77 months (41). Three cats were started on a low DOCP dose of 1.5‐1.6 mg/kg (1.5) and five on a higher dose of 2‐2.5 mg/kg (2). In all low‐dose cats, DOCP had to be increased within the first months. The maximal DOCP doses used were 2.3‐2.8 mg/kg (2.4). In the higher‐dose cats, DOCP was increased (3) to 2.5‐3 mg/kg (2.5), left unchanged (1) or decreased (1). DOCP doses of all eight cats at the end of the follow‐up period ranged from 1.3‐3 mg/kg (2.25).

Prednisolone doses at discharge ranged from 0.4‐1 mg/kg/d (0.73) and could be decreased in all to doses of 0.08‐0.6 mg/kg/d (0.28) at the end of the follow‐up period.

Cats in the present study needed higher DOCP doses than what has been published in dogs with hypoadrenocorticism. A starting dose of 2.2 mg/kg in cats seems warranted. Also, prednisolone dose requirement seems to be higher in cats than in dogs.

**Disclosures**

Disclosures to report, please report below

Employee/salary:‐ Grants/research: Novartis Foundation for medical‐biological Research, Albert‐Heim Stiftung, Bern, Switzerland, Stiftung für wissenschaftliche Forschung der Universität Zürich, Gerber‐ten‐Bosch Stiftung (NSR and FB) Nestlé Purina, Hill's, Provet, Antlia SA, Glycemicon, Clinical Studies fund of the ECVIM‐CA and from the Society of Comparative Endocrinology (CR, now retired) Speaking & consultancies: Member of the Vetoryl novel monitoring meeting 2017 organized by Dechra Veterinary Products Ltd., invited speaker at the Zycortal Symposium hosted by Dechra near Manchester in 2017 (NSR) Investments/commercial interests: ‐ Gifts, hospitality, travel support: ‐

## 97 ESVE‐O‐7

### 97.1 ESVE ‐ European Society of Veterinary Endocrinology

#### 97.1.1 Canine pituitary organoids and tumoroids as 3D in vitro model for pituitary‐dependent hypercortisolism

##### 97.1.1.1 K. Sanders ^1^, F.C.A. Ringnalda ^2^, F.G. Steenbeek ^1^, L. Vree ^1^, H.S. Kooistra ^1^, B.P. Meij ^1^, M.L. Wetering ^2^, J.C. Clevers ^2^, S. Galac ^1^

###### 97.1.1.1.1 ^1^ Faculty of Veterinary Medicine, Utrecht University Utrecht Netherlands; ^2^ Princess Máxima Center Utrecht Netherlands

Pituitary‐dependent hypercortisolism (PDH), one of the most common endocrine disorders in dogs, is usually caused by an ACTH‐secreting pituitary adenoma. If left untreated, PDH causes significant morbidity and mortality. The pituitary adenoma can be surgically removed (hypophysectomy), but this is only performed in highly specialized clinics. Many dogs are therefore treated medically with the steroidogenesis inhibitor trilostane, which does not inhibit the growth of the pituitary adenoma. To find and test medical treatment options that do inhibit pituitary adenoma growth, we need a suitable *in vitro* model that mimics the adenoma's biological behavior. We therefore aimed to establish 3D canine pituitary organoid and tumoroid cultures.

We successfully cultured organoids from canine normal anterior pituitaries (n = 3) and tumoroids from surgically‐resected ACTH‐secreting pituitary adenomas (n = 12). Organoids/tumoroids efficiently grew out from all tissues. They expanded rapidly and could be passaged once every 7 days in a 1:4 ratio. At passage 5, we collected material for protein, RNA, and DNA analyses. We have performed whole genome and RNA sequencing on the tumoroids and corresponding primary adenomas and are currently analyzing these data to determine whether the tumoroids are derived from tumor cells and to assess the expression of drug targets. Additionally, we are characterizing all organoids/tumoroids with immunohistochemistry. Based on literature, we selected 10 drugs that could potentially inhibit pituitary adenoma growth and have tested these in a 10‐fold dilution series on the first tumoroid line. This first drug screen showed promising proliferation‐inhibiting results for several of the included drugs.

This study is the first to report culture of pituitary organoids from dogs, and of pituitary tumoroids from any species. This novel pituitary model will pave the way to identify new treatment options for PDH efficiently and reliably. Not only for dogs, but also for their best friends: humans.

**Disclosures**

No disclosures to report

## 98 ESVE‐O‐8

### 98.1 ESVE ‐ European Society of Veterinary Endocrinology

#### 98.1.1 Monitoring of diabetes mellitus with Flash glucose monitoring system: the owners’ point of view

##### 98.1.1.1 M. Re ^1^, F Del Baldo ^2^, A. Tardo ^2^, F Fracassi ^2^

###### 98.1.1.1.1 ^1^ University of Bologna Modena Italy; ^2^ University of Bologna Ozzano dell'Emilia (BO) Italy

Flash glucose monitoring system (FGMS) has recently become one of the most common monitoring methods in dogs and cats with diabetes mellitus; however, the owners' opinion on its use has never been investigated.

The aim of this study was to evaluate the impact of FGMS on diabetic pet owners (DPOs) quality of life and the satisfaction related to its usability.

DPOs who used at least one FGMS on their diabetic pet were eligible for inclusion in the study.

Owners were asked to answer 30 multiple‐choice and open‐ended questions survey.

Twenty‐nine diabetic dogs’ owners and twenty‐one diabetic cats’ owners were included.

The median (range) number of FGMSs used by DPOs was 4 (1‐10).

The FGMS was proposed by a referral center, by the primary care veterinarian or it was found out by owners’ self‐information in 62%, 20% and 12%, respectively. In 58% of cases, FGMS was placed by the veterinarian only, while 42% of DPOs (68% dogs’ owners and 32% cats’ owners) applied it on their own.Even if FGMS is already provided with an adhesive portion, 68% of DPOs used additional glue in order to extend the device wearing time; moreover, in 88% of cases, the sensor was protected with an additional bandage. Before using FGMS all diabetic pets were monitored with blood glucose curves (BGCs) performed at home or in the hospital. More than 80% of DPOs considered FGMS easier to use, less stressful and painful for the animal, and it allowed to obtain more glucose data with less effort, compared to BGCs. Overall, 92% of DPOs reported that their pet had better diabetes control since using FGMS. The most challenging aspects of using the FGMS were ensuring proper sensor fixation during the wearing period (46%), preventing premature detachment (38%) and purchasing the sensor (34%). Moreover, 36% of DPOs reported that the device cost was difficult to afford in the long term.Among DPOs using FGMS, the possibility to access the glucose concentrations generated either increased anxiety (12%) or else a sense of control (88%). Comparing dogs and cats, a significantly (p<0.05) higher number of dogs' owners retained FGMS well‐tolerated (79% vs 40%), less invasive than BGCs (79% vs 42%) and easier to maintain in situ (75% vs 42%).

In conclusion, FGMS is considered by DPOs easy to use, less stressful compared to BGCs while enabling better glycemic control. Nevertheless, costs related to its long‐term use might be difficult to sustain.

**Disclosures**

No disclosures to report

## 99 ESVE‐O‐9

### 99.1 ESVE ‐ European Society of Veterinary Endocrinology

#### 99.1.1 Accuracy of a flash glucose monitoring system for cats with diabetic ketosis or ketoacidosis

##### 99.1.1.1 Y Ciu^1^, J Eiermann ^2^, ^1^ Veltem‐Beisem Belgium; ^2^ Luzern Switzerland

###### 99.1.1.1.1 Cats with diabetic (keto)acidosis DK(A) require intensive glucose monitoring. The latest FreeStyle Libre 2.0 Abbott®, a factory‐calibrated flash glucose monitoring system (FGMS), has not been validated in such patients.

The aim of this prospective, observational study was to assess the accuracy of this new generation FGMS in cats with DK(A) and how this is impacted by inter‐individual variability and hydration status. An additional aim was to report owner satisfaction at home.

Within 24 hours of diagnosis, the FGMS sensor was placed in the cat's neck region. Blood glucose (BG) concentrations were measured with a portable blood glucose meter (PBGM) every 1‐3 hours before and at least every 4‐6 hours after resolution of DK(A) and compared to the FGMS interstitial glucose (IG) values. The accuracy of the FGMS was evaluated according to ISO 15197:2013 criteria. Calculation of differences and bland Altman‐plot were used for the analytical accuracy and Parkes Consensus Error Grid analysis for clinical accuracy. Kruskal‐Wallis ANOVA (KWA) was used to evaluate the differences in accuracy depending on the hydration status and inter‐individual variability was evaluated using box plots and KWA.

Nine client‐owned cats with naturally occurring DK(A) were included. Bland‐Altman plot showed only 51% (instead of 95%) of all results were within ± 0.83 mmol/L for BG <5.5 mmol/L and 19% (instead of 95%) of all results within 15% for BG ≥5.5 mmol/L. The mean absolute relative difference and median absolute relative difference were above 14%, independent of glycemic and hydration state, failing the requirements for analytical accuracy. The clinical accuracy was also not achieved. The total percentage of observations falling in zones A and B in the Parkes plots was 98.17%, which is below the desired limit of 99%. The median BG value (21.8 mmol/l dehydrated versus 17.1 mmol/l euhydrated) was significantly higher than the median IG value (15.9 mmol/l dehydrated versus 13.4 mmol/l euhydrated) in all hydration states (p<0.001). There was significant inter‐individual variation in the difference between BG and IG measurements (p <0.001). The effect of hydration status on the accuracy of the FGMS was minimal (p > 0.05). Overall owner satisfaction with the FGMS was good.

In conclusion, the new generation FGMS did not fulfill ISO requirements and is therefore not sufficiently accurate for glucose monitoring in cats with DK(A). Additionally, it showed significant inter‐individual variability. Comparing BG and IG concentrations to evaluate the FGMS accuracy in each individual cat is therefore strongly recommended.

**Disclosures**

Disclosures to report, please report below

An ECVIM‐CA CSF 2020 grant (1500€) was used to finance a part of this study.

## 100 ESVE‐O‐10

### 100.1 ESVE ‐ European Society of Veterinary Endocrinology

#### 100.1.1 A mutation in the KCNJ11 gene and diabetes mellitus in the Labrador retriever

##### 100.1.1.1 S Falcone ^1^, M Wallace ^2^, A Denyer ^2^, ME Herrtage ^3^, C Mellersh ^3^, S Ricketts ^3^, B McLaughlin ^3^, K Hughes ^3^, LJ Kennedy ^4^, A Psifidi ^2^, IK Ramsey ^5^, E Schofield ^3^, G Williams ^6^, PJ Watson ^3^, N Zimmerman ^7^, D Xia ^2^, CA O'Callaghan ^1^, B Catchpole ^2^, LJ Davison ^2^

###### 100.1.1.1.1 ^1^ Wellcome Centre for Human Genetics Oxford United Kingdom; ^2^ Royal Veterinary College London United Kingdom; ^3^ University of Cambridge Cambridge United Kingdom; ^4^ University of Manchester Manchester United Kingdom; ^5^ University of Glasgow Glasgow United Kingdom; ^6^ Dechra Veterinary Products Shrewsbury United Kingdom; ^7^ Dechra Veterinary Products Portland United States

The ATP‐sensitive potassium channel (K_ATP_ channel) has a pivotal role in pancreatic β‐cell physiology, regulating insulin secretion by closing in response to ATP produced by glucose metabolism. In humans, mutations in the genes encoding the two subunits of the K_ATP_ channel, *KCNJ11* (Kir6.2) and *ABCC8* (SUR1), impair insulin secretion and cause rare monogenic forms of diabetes mellitus (DM) in neonates and early adult life. Sulfonylurea drugs can be used to rectify the secretion impairment in many such human patients, closing the K_ATP_ channel directly by binding to the SUR1 subunit, and resolving hyperglycaemia without insulin therapy.

This study used DNA from client‐owned dogs, from mouth swabs or archived surplus clinical samples, to investigate the role of *KCNJ11* mutation in canine DM. Using whole exome sequencing, a homozygous missense mutation in *KCNJ11* (Kir6.2‐D274N) was identified in 2 diabetic Labrador retrievers (LRs), resulting in a change of a highly conserved aspartic acid (D) to asparagine (N) at position 274 of Kir6.2. A further 121 diabetic LRs (109 adult‐onset and 12 juvenile‐onset DM) and 200 non‐diabetic adult LRs were genotyped by Sanger sequencing, next‐generation sequencing or TaqMan genotyping. A total of 9 homozygotes for the mutation were identified, 8 of which were diabetic (age of onset 5 months to 9.5 years). The non‐diabetic D274N homozygote LR was reported to have suffered one episode of pancreatitis, but remained non‐diabetic when euthanised at 11 years of age. The Kir6.2‐D274N mutation was not identified in any breed outside the LR in publicly available data (Dog Biomedical Variant Database Consortium, Dog Genome Project, Give a Dog a Genome).

Kir6.2 three‐dimensional modelling was undertaken using The PyMOL Molecular Graphics System, using the reported human K_ATP_ bound to ATP and ADP in quatrefoil form (Protein Data Bank accession no. 6C3O). Substitution with the mutant N274 residue was predicted to disrupt one of the two polar contacts with Q279 and the polar contact with A271.

Functional work is currently being performed and a genetic test is in development. Human neonatal diabetes patients carrying damaging Kir6.2 mutations can be successfully treated with early intervention using oral sulfonylureas, implying that early oral sulfonylurea treatment may offer an effective therapy for canine DM associated with Kir6.2‐D274N. Genetic testing will not only have potential use in a precision medicine approach to diabetes mellitus in the LR, but may also help reduce the incidence of diabetes in the breed by avoiding inter‐breeding of Kir6.2‐D274N carriers.

**Disclosures**

Disclosures to report, please report below

Direct canine diabetes genetics funding – PetPlan Charitable Trust, ECVIM‐CSF, BBSRC LiDO Scheme, RVC Clinical Research Fellowship (MW), Dechra Veterinary Products. UK Canine Diabetes Archive and Database / sample and historic funding: MSD (Intervet), BSAVA PetSavers, Kennel Club Charitable Trust, Boehringer, Wellcome Trust, Samoyed Association Indirect funding supporting current research of authors (data not presented here): Medical Research Council (LJD Clinician Scientist Fellowship), American Kennel Club Canine Health Foundation, Morris Animal Foundation, UK International Coronavirus Network, Royal Canin, Novo Nordisk Foundation, Evetts‐Luff Foundation, BSAVA PetSavers, UK Research and Innovation, Hong Kong Jockey Club. Additional details: A patent application is in progress associated with this data, which may result in a genetic test in future, in association with the UK Kennel Club.

## 101 ESVE‐O‐11

### 101.1 ESVE ‐ European Society of Veterinary Endocrinology

#### 101.1.1 Once daily oral therapy for feline diabetes mellitus: SGLT‐2‐inhibitor velagliflozin as stand‐alone therapy compared to insulin injection therapy in diabetic cats

##### 101.1.1.1 S.J.M. Niessen ^1^, R. Voth ^2^, C. Kroh ^3^, L. Hennings ^3^

###### 101.1.1.1.1 ^1^ Veterinary Specialist Consultations Hilversum Netherlands; ^2^ Boehringer Ingelheim St Joseph United States; ^3^ Boehringer Ingelheim Vetmedica GmbH Germany

Currently, feline diabetes mellitus (DM) requires (often BID) insulin injections in most cases. Its management is relatively complex with a constant hypoglycaemia danger. Oral treatment methods, frequently employed in human type 2 DM, have previously been found ineffective in cats. The current study investigated the use of oral sodium‐glucose‐co‐transporter‐2‐inhibitor (SGLT‐2‐inhibitor) velagliflozin. In humans, SGLT‐2‐inhibitors can achieve euglycaemia through urinary glucose excretion, leading to reduced glucose toxicity and often improved beta‐cell function.

A prospective, randomised (1.0 mg/kg once daily oral velagliflozin or individually titrated BID lente insulin injections), open‐label, 60‐day field study was conducted. Diagnosis of DM was consistent with ALIVE‐criteria. Cats were screened for concurrent/ underlying disease (e.g., hypersomatotropism, hyperthyroidism) and excluded if present. Efficacy evaluation included clinical evaluation by clinician and owner and glycaemic control assessment (9‐hour blood glucose [BG]‐curve; fructosamine). Data were compared between days ‐1, 7, 14, 30 and 60. Urine was regularly checked for ketones.

Twenty‐six cats were included (13 per group; 4 [insulin] and 6 [velagliflozin] insulin pre‐treated). Fructosamine decreased significantly from baseline (least‐squares means) in the velagliflozin‐group from 604 to 489, 422, 401, and 398 μmol/L (days –1, 7, 14, 30, 60; p<0.0001); in the insulin‐group the decrease from day ‐1 was significant on day 60 (561 and 461 μmol/L; p<0.05). Mean BG from the curves decreased significantly at all visits for velagliflozin‐cats (388, 194, 212, 202, 185 mg/dL; p<0.0001). Insulin‐cats showed a significant decrease on days 30 and 60 (420, 405, 405, 351, 291 mg/dL; p<0.05). Mean BG was significantly lower in velagliflozin‐cats compared to insulin‐cats on days 7, 14 and 30. On days 30 and 60, the velagliflozin‐group achieved mean BG‐values below the renal threshold (<270 mg/dL; range: 166‐257 mg/dL) for the entire 9‐hour curve, including t=0 hours, implying >24 hours duration. Improvement in at least one DM sign had occurred in 11/13 (velagliflozin) and 10/13 (insulin) on day 60. Clinician‐ and owner‐assessed clinical control and quality of life improved in both groups. Non‐clinical hypoglycaemia was reported in 3/13 (velagliflozin) and 6/13 (insulin); clinical hypoglycaemia was not observed. Diabetic keto‐acidosis occurred in 1/13 (velagliflozin) and was treated successfully. Soft stools/ diarrhoea occurred in 8/13 (all velagliflozin; self‐limiting in 4/8).

The current study suggests use of stand‐alone once daily oral SGLT‐2‐inhibitor velagliflozin may result in clinical and biochemical control of DM in a majority of cats, resulting in improved clinical signs and quality of life, without the need for injection therapy.

**Disclosures**

Disclosures to report, please report below

S.J.M. Niessen ‐ performs paid consultancy work for Boehringer Ingelheim, the developer and future manufacturer of the drug discussed in the abstract ‐ also paid consultancy and research work for: Dechra Pharmaceuticals, Purina, Abbott/now Zoetis, Hill's, MSD Animal Health B. Voth: employee of Boerhinger Ingelheim, the developer and future manufacturer of the drug discussed in the abstract C. Kroh: employee of Boehringer Ingelheim, the developer and future manufacturer of the drug discussed in the abstract L. Hennings: employee of Boehringer Ingelheim, the developer and future manufacturer of the drug discussed

## 102 ESVE‐O‐12

### 102.1 ESVE ‐ European Society of Veterinary Endocrinology

#### 102.1.1 Histological investigation of the endocrine pancreas in non‐diabetic cats and its relationship with pancreatitis

##### 102.1.1.1 B. Lombardo ^2^, S. Ferro , P. Kook , D. Trez , L.M. Coppola , S. Ciccarelli, T.A. Lutz , E. Zini

###### 102.1.1.1.1 ^1^ Cinisello Balsamo Italy; ^2^ Cinisello balsamo Italy

Based on histology, pancreatitis is often encountered in cats that die due to a variety of causes, particularly diabetes mellitus. However, whether pancreatitis anticipates diabetes by decreasing the number of alpha and beta‐cells remains to be clarified. Hence, the aim of the study was to investigate the relationship between the endocrine pancreas and the inflammation of the exocrine pancreas based on histopathology and circulating markers of pancreatitis in non‐diabetic cats.

Cats without diabetes that died or were euthanized due to different diseases were included if the pancreas was removed within 3 hours after death, and the leftover of a blood sample collected 12 hours before death was available to measure 1.2‐o‐dilauryl‐rac‐glycero‐3‐glutaric acid‐6‐methylresorufin ester (DGGR)‐lipase and feline pancreas‐specific lipase (Spec‐fPL ) activity. Slides from the pancreatic body were obtained and immunohistochemistry was performed for insulin, glucagon, CD3 (T‐lymphocytes), CD20 (B‐lymphocytes), Iba‐1 (macrophages) and myeloperoxidase (neutrophils). Acute and chronic pancreatitis was diagnosed according to published criteria (De Cock et al., 2007). Furthermore, the histological activity index of pancreatitis (Oppliger et al., 2016) was calculated and the number of inflammatory cells counted. Insulin‐ and glucagon‐positive cross‐sectional areas were compared between cats with and without pancreatitis.

Sixty cats were included. The insulin‐ and glucagon‐positive areas did not differ between cats with and without acute and chronic pancreatitis. However, a negative correlation was observed between the glucagon‐positive area and the pancreatitis activity index (r=‐0.440, CI95%=‐0.643 to ‐0.179; P=0.001) as well as DGGR‐lipase (r=‐0.426, CI95%=‐0.633 to ‐0.163; P=0.002). Additionally, the glucagon‐positive area was lower in cats with Iba‐1‐positive cells in the exocrine pancreas than those without (median=0.87, range=0.26‐1.35, vs. median=1.18, range=0.26‐3.47; P=0.033). Differences were not observed for insulin‐positive area and any histological criteria, inflammatory cells and circulating markers of pancreatitis.

Collectively, the results suggest the presence of a negative relationship between pancreatitis and number of alpha‐cells but not beta‐cells in cats without diabetes. Because beta‐cell viability is supported by alpha‐cells in humans and rodents, it is tempting to speculate that pancreatitis in cats, by decreasing the number of alpha‐cells, has a negative effect on beta‐cells and promotes the development of diabetes in some.

**Disclosures**

No disclosures to report

## 103 ESVE‐O‐13

### 103.1 ESVE ‐ European Society of Veterinary Endocrinology

#### 103.1.1 Assessment of the effect of inflammation on the quality of glycaemic control, insulin sensitivity and β‐cell function in diabetic cats

##### 103.1.1.1 S. Thalmeier ^1^, T. Jaresova ^1^, R. Gostelow ^2^, N. Bauer ^1^, K. Hazuchova ^1^

###### 103.1.1.1.1 ^1^ Justus‐Liebig‐University Giessen Germany; ^2^ Royal Veterinary College London United Kingdom

It is suggested that inflammation might impact the quality of glycaemic control in diabetic cats. However, the crosstalk between inflammation and diabetes mellitus has never been evaluated in prospective clinical studies. Thus, this study aimed to assess the interaction between acute phase reaction (APR) (indicating inflammation) and markers reflecting diabetic control, ß‐cell function (BCF), and insulin resistance (IR) in diabetic cats.

Surplus serum samples from diabetic cats (n=69) who participated in 2 prospective clinical trials investigating different antihyperglycaemic therapies between 2013 and 2018 were used to measure acute phase proteins (APPs) serum amyloid A (SAA), a1‐acid glycoprotein (AGP) and haptoglobin (Hp). Cats diagnosed with DM <6 months ago were enrolled after passing screening laboratory tests and diagnostic imaging; cats with comorbidities were not excluded. Clinical and laboratory data including fructosamine concentration, BCF test results (insulin peak response [IPR] and total insulin secretion [TIS] from glucagon stimulation tests) and IR measures (Quantitative Insulin Sensitivity Check Index [QUICKI], Homeostasis Model Assessment of Insulin Resistance [HOMA‐IR]) from 4 trial visits (enrolment, month 1, 3 and 6) were included in the analysis. The effect of the quality of glycaemic control (fructosamine <450μmol/l indicating good control) on the concentration of the 3 APPs, as well as the effect of the presence of APR (indicated by increase in concentration of at least 2 APPs) on fructosamine concentration, BCF and IR measures was evaluated by mixed effects modelling. Significance was set at p<0.05, and average values expressed as median (range).

Glycaemic control and BCF improved over the 6‐month trial period. Fructosamine decreased between enrolment (489 [217‐717]) and month‐6 (326 [215‐581] μmol/l) (p<0.001). IPR and TIS increased between enrolment and month‐6 (IPR: enrolment 0 [0‐54.5], month‐6 19.6 [0‐423.8] pmol/l, p<0.001; TIS: enrolment 0 [0‐1185.1], month‐6 653 [0‐13107.8] pmol/l.min^‐1^, p<0.001). There was no effect of the quality of glycaemic control on the concentration of any of the 3 APPs (SAA p=0.35; AGP p=0.59; Hp p=0.1), nor an effect of the presence of APR on fructosamine concentration (p=0.35), BCF (IPR p=0.89; TIS p=0.99) or IR measures (QUICKI p=0.28; HOMA‐IR p=0.25). Individual cases with increased APPs concentrations had poor glycaemic control and were diagnosed with inflammatory or neoplastic diseases.

In conclusion, this study found no association between APR and glycaemic control, BCF or IR. However, in individual cats the presence of APR might indicate an inflammatory or neoplastic process.

**Disclosures**

Disclosures to report, please report below

The study is supported by the ECVIM‐CA and Purina Institute Resident Research Awards 2020

## 104 ESVCN‐O‐1

### 104.1 ESVCN ‐ European society of Veterinary & Comparative Nutrition

#### 104.1.1 Body composition and energy expenditure of obese dogs on weight loss programs fed diets with different amino acid profiles

##### 104.1.1.1 L. Luis ^1^, A. Carciofi ^1^, C. Goloni , M. Tozato , L. Pacheco , M. Monti ^2^

###### 104.1.1.1.1 ^1^ UNESP Jaboticabal Brazil; ^2^ Special Dog Company Santa Cruz do Rio Pardo Brazil

Body composition (BC) and energy expenditure (EE) of 20 non‐obese (NO; body condition score [BCS] 5/9) and 20 obese client owned dogs (OB; BCS 7‐9/9) were compared. The BC and EE were estimated using stable isotopes of deuterium and ^18^O. Two diets were used, a control (CO; 31.7% protein, 25.2% dietary fiber, and 3.0 kcal/g of dry matter), and the CO formulation supplemented with amino acids (AA; methionine, tryptophan, threonine and valine). On the sequence the OB dogs underwent a controlled weight loss (WL; 10 dogs per diet). Dogs were initially fed 60 kcal/kg^0.75^/day, adjusted to promote 1% WL/week. The EE and BC were measured at start, after 10% and 20% of WL. Phase 1 was evaluated in a 2x2 factorial arrangement and phase 2 by analysis of variance of repeated measures (P<0.05). In phase 1 OB dogs showed 43.1±1.3% of fatty mass (FM) and 56.9±1.3% of lean mass (LM), while NO 23.4±1.4%and 76.6±1.4% (P<0.01). The mean EE was similar among groups (98.6±6.7 kcal/kg^0.75^/day; P=0.67), but per kg/LM was higher for OB (P<0.01). This difference might be related to a greater muscular requirement for movement and cardiorespiratory function. In phase 2 the mean WL rate tended to be higher for AA (0.83±0.07%) than CO (0.63±0.08%; P=0.08). Although statistically similar (P=0.53), CO lost 0.9 kg of LM along the regimen, but AA maintained LM. The initial EE (97.0±4.6 kcal/kg^0.75^/day) increased 6% after 10% and 19% after 20% WL (P<0.01). This increase was higher for AA, that after 20% WL tented to present higher EE than CO (AA=128.4±6.7; CO=103.7±7.0 kcal/kg^0.75^/day; P=0.07). The EE represents the energy from food and body tissue oxidation. Energy supply from food did not change along the regimen (57.0±1.2 kcal/kg^0.75^/day; P=0.84). The difference between energy intake and the estimated EE represents heat production from body tissue oxidation, amounting respectively, 37±4, 43±4, and 54±5 kcal/kg^0.75^/day (P=0.03). So, despite the lower WL rate on the first (0‐10%) than the second half (10‐20% WL) of the regimen (0.91%±0.07 vs. 0.55%±0.08 of WL/week; P<0.01), the theoretical energy deficit and EE in fact increased. Possible explanations include lack of owners’ cooperation or changes in the oxidized energy substrate. Preservation of LM and greater oxidation of FM may have increased the energy production per g of body weight reduced. Amino acids correction may increase EE and maximize body fat oxidation, favoring a faster weight loss and helping to avoid LM loss.

**Disclosures**

No disclosures to report

## 105 ESVCN‐O‐2

### 105.1 ESVCN ‐ European society of Veterinary & Comparative Nutrition

#### 105.1.1 Development of a Diet‐Induced Dyslipidemia Model in Healthy Dogs Fed a Westernized Diet

##### 105.1.1.1 L. Van Vertloo ^1^, C Iennarella‐Servantez ^1^, N Tate ^2^, E Furrow ^2^, J Mochel ^1^, A Allenspach ^1^

###### 105.1.1.1.1 ^1^ Iowa State University Ames United States; ^2^ University of Minnesota St. Paul United States

Transition to a “Westernized” diet (WD) that is rich in fat and monosaccharides and low in fiber has corresponded with an increase in numerous chronic illnesses in humans. The development of dyslipidemia is one of the factors tied to these illnesses. Dogs are an ideal translational model for studying dietary effects applicable to humans due to similarities in gastrointestinal physiology and microbiota and the spontaneous development of similar chronic diseases. The objective of this study was to investigate the effect of isocaloric feeding of a WD on serum lipoproteins in a healthy dog model.

Ten healthy beagle dogs were fed either 1) a control diet (CD) formulated to meet National Academies recommendations or 2) a WD formulated to reproduce National Health and Nutrition Examination Survey dietary intake data. Diets were prepared cooked with the same ingredients and formulated to meet or exceed nutrient and energy requirements established for both humans and dogs. The study was divided into three consecutive feeding periods: (1) five weeks of CD, followed by (2) seven weeks of WD and (3) four weeks of recovery with CD. Serum was sampled at the end of each feeding period and four weeks into the WD period. Serum lipoprotein profiles were generated using a density gradient ultracentrifugation technique. Pairwise Wilcoxon rank sum tests were performed. Lipoprotein profiles were analyzed by principal component analysis (PCA) and clustered using unsupervised hierarchical cluster analysis based on Spearman correlation distances coupled with Ward's linkage method.

There were significant shifts in serum lipoproteins that occurred after four weeks of feeding a WD. Specifically, increases in triglyceride‐rich lipoproteins (TRL) (*P*<0.05) and multiple high‐density lipoprotein (HDL) subfractions (*P*<0.05) were observed as well as decreases in some low‐density lipoprotein (LDL) subfractions (*P*<0.05). Some of these changes did not normalize following feeding of CD for an additional four weeks. Hierarchical clustering and PCA grouped samples into three clusters, largely by feeding period. The changes observed, namely decreased LDL subfractions and increased TRL, are reminiscent of what is seen in Miniature Schnauzers with and without primary hypertriglyceridemia. This is in contrast with dyslipidemia associated with metabolic syndrome and various chronic illnesses in humans, which is characterized by an increase in LDL and a decrease in HDL subfractions.

Our preliminary findings suggest that lipoprotein profiles are altered after short‐term feeding with a WD in healthy dogs. Longer‐term studies are warranted to determine whether these changes are of clinical significance.

**Disclosures**

No disclosures to report

## 106 ESVCN‐O‐3

### 106.1 ESVCN ‐ European society of Veterinary & Comparative Nutrition

#### 106.1.1 The effect of starch to protein ratio in energy expenditure, body composition, and physical activity are different in neutered and intact, non‐obese male cats

##### 106.1.1.1 C. Goloni, L. Pacheco, L. Warde Luis, S. Souza Theodoro, L. Bassi Scarpim, C. Torres, A. Cavalieri Carciofi

###### 106.1.1.1.1 São Paulo State University ‐ UNESP Jaboticabal Brazil

Neutering is a risk factor for obesity, altering energy metabolism and body composition of male cats. The intake of diets with different starch to protein ratios was compared in non‐obese neutered (NM) and intact (IM) male cats living in homes and fed *ad libitum*. A kibble diet high in starch (HS: Starch 40%, crude protein [CP] 38%) or high in protein (HP: Starch 20%, 55% CP) was fed for 4 months in a *cross‐over* design. The physical activity was measured with 3‐axial accelerometer fixed to chest collar previously adapted to cats, and body composition, energy expenditure (EE) and water turnover (WT) were evaluated by the doubly labelled water method. Results were evaluated in a 2 (diets) x 2 (reproductive conditions) arrangement totaling 4 treatments, and compared by analysis of variance, Tukey test and Pearson correlation (P≤0.05). The cats which complete the study included: IM; n = 10; 1.6 ± 0.8 years, body condition score (BCS) 5.0 ± 0.0 (in a 9 point scale); NM, n = 9; 2.2 ± 1.2 years, BCS 5.1 ± 0.1. The age of the cats (P=0.54) and their BCS (P=0.98) were similar between groups. The IM cats presented higher EE (481.16 ± 29.12 kJ/kg^0.67^/d), lean mass (LM; 90 ± 0.8 %), physical activity (5900 ± 192 G, overall dynamic body acceleration [ODBA]) and water turnover rate (89.7 ± 6.4 mL/kg^0.67^/day) compared to NM (382.66 ± 28.11 kJ/kg^0.67^/d; 84 ± 1.1% of LM; 5424 ± 200 G, ODBA; 66 ± 6.4 mL/kg^0.67^/day; P<0.05). When NM were fed the HS diet, they did not change body weight (BW), but their fatty mass reduced 21% (P=0.04) and LM increased 4% (P<0.01). When fed the HP diet, the BW and LM increased 5% and 7% in NM (respectively, P<0.05). No diet effect was observed for BW or composition to IM cats (P>0.05). The intake of the HP diet, regardless of sexual condition, increased EE in 10% compared to HS diet (P<0.05) and tended to increase the physical activity of the cats in 12 % (P=0.09). Positive correlation was observed between EE and water turnover (R^2^ =0.62; P<0.01) and EE and physical activity (R^2^ =0.42; P<0.01). Intact male cats had lower fat mass and higher lean body mass in percentage, and higher EE, water turnover and physical activity than neutered non‐obese male cats. In NM the intake of the HS diet helped to maintain BW and improved body composition.

**Disclosures**

No disclosures to report

## 107 ESVCN‐O‐4

### 107.1 ESVCN ‐ European society of Veterinary & Comparative Nutrition

#### 107.1.1 Effects of physical exercise in dogs and dog owners – assessed quality of life, body condition score and body mass index

##### 107.1.1.1 E. Roman ^1^, E. Lundbeck ^1^, S. Spörndly‐Nees ^2^, L. Kallings ^3^, A. Bergh ^1^, J. Söder ^1^

###### 107.1.1.1.1 ^1^ Swedish University of Agricultural Sciences Uppsala Sweden; ^2^ Uppsala University Uppsala Sweden; ^3^ The Swedish School of Sport and Health Sciences Stockholm Sweden

Dog owners and dogs often share lifestyle, e.g. physical activity level, which could impact health parameters of both parties. Quality of life (QoL) is discussed to depend on psychological and physical factors. Overweigh is a common physical health problem in dogs, as in humans, associated with impaired QoL. The aim of the study was to evaluate the effect of physical exercise on owner‐assessed QoL in dogs and owners as well as on body condition score (BCS) in dogs and on body mass index (BMI) in dog owners, before and after a standardized exercise intervention.

The participants, 22 owners and their dogs, underwent a standardized exercise intervention for eight weeks where a target level of running 2, 5, 7.5 or 10 kilometers (km) were chosen by the owner. Before and after the intervention the dog owners completed two separate questionnaires evaluating QoL in dogs and themselves respectively, and underwent repeated clinical evaluations for BCS and BMI. The questionnaire for dogs was based on ten question‐blocks, evaluating different behaviors related to QoL. After the intervention, the dog owners were also asked to assess via direct estimation if they considered that the dog had changed its behavior regarding each question block. The questionnaire for dog owners consisted of selected questions from WHOQoL‐SRPB. The participants were divided into two groups; 2 km (n=8) and 5‐10 km (n=14) and data were evaluated by mixed model repeated measurement analyses.

Dog owners showed a significantly improved QoL (*P*=0.02) after the intervention (3.9±0.6 versus 4.1±0.7) and considered that their dogs had improved behaviors within the question blocks "vitality, activity and movement" and "direct assessment of QoL". However, there was no significant difference after the intervention compared to before in any of the ten question blocks estimating behaviors related to QoL in dogs. A significantly reduced mean BCS (*P*=0.008) was seen in dogs after the intervention (5.1±0.9 versus 4.7±0.6), but BMI of the owner was not affected.

Study results suggest that an active shared lifestyle in dog owners and dogs has a positive effect on BCS in dogs, as well as on self‐assessed QoL of the owner. These health benefits were obtained already at the lowest target level (2 km), which implies that even owners unable to perform physical exercise at a higher level should be encouraged to implement a shared active lifestyle together with their dog.

**Disclosures**

No disclosures to report

## 108 ESVCN‐O‐5

### 108.1 ESVCN ‐ European society of Veterinary & Comparative Nutrition

#### 108.1.1 Starch to protein ratio and moisture content of the food influence water balance and urine supersaturation for calcium oxalate and struvite in cats.

##### 108.1.1.1 M.E. Tozato, S. Theodoro, L.W. Luis, P. Costa, C. Goloni, A. Carciofi

###### 108.1.1.1.1 São Paulo State University (UNESP), Jaboticabal, Brazil Jaboticabal Brazil

The present study evaluated the water balance, urine characteristics, renal excretion of oxalate and calcium and the urine relative superssaturation (RSS) for calcium oxalate (CaOx) and struvite of cats fed dry or wet foods, with two starch to protein ratios. Two starch to protein ratios (high starch [HS]: 25% starch and 36% protein; high protein [HP]: 15% starch and 53% protein on dry matter), and two moisture contents (5%, dry kibbles; 80%, wet food) were compared in a 2 x 2 factorial arrangement totalling 4 diets. Each diet was evaluated in 9 cats, with 10 days of adaptation and 8 days of quantitative collection of urine and faeces. Urine volume, density, pH, calcium, phosphorus, uric acid, chloride, sodium, potassium, sulphur, citrate, oxalate, and ammonia were evaluated. Based on the results, urine RSS for CaOx was calculated using EQUIL‐93 software and SUPERSAT software was used to CaOx and struvite RSS calculations. The water balance was determined computing water intake at the drinker and via food, metabolic water, water excretion via faeces and urine and insensible losses . Results were submitted to analysis of variance considering the effects starch to protein ratio, moisture content and their interactions (P<0.05). The cats’ urine presented similar pH (P>0.05; 6.2±0.36 pH). Urine specific gravity was lower and their volume higher in cats fed wet foods (P<0.01), which also induced urine with lower RSS for CaOx and struvite than dry foods (P<0.01). High protein consumption increased urine volume (P<0.05), however, it did not reduce urine specific gravity (P<0.05). Ca intake was higher for dry and HS foods, but it urine concentration higher for dry and HP diets (P<0.05). Oxalate urine concentration was 60% higher for cats fed the HS than the HP formulations (P<0.05). It was observed that high protein intake resulted in higher calciuria in cats, however, the reduction in oxalate renal excretion due the limitation on starch intake, together with the induced increase in urine volume justified the lower RSS for CaOx observed for the HP (P<0.01), suggesting be an interesting approach in the control this urolith in cats. However, for dry foods, the HP increased the RSS for struvite in comparison with the HS diet (P<0.01).

**Disclosures**

No disclosures to report

## 109 Poster abstracts ECVIM‐CA Congress 2022

## 110 ESVC‐P‐1

### 110.1 ESVC ‐ European Society of Veterinary Cardiology

#### 110.1.1 Normalized left atrial diameter in healthy and cardiomyopathic cats

##### 110.1.1.1 V. Patata, V. Vezzosi , C. Fabris, F Marchesotti, M Bini, M Croce, O Domenech

###### 110.1.1.1.1 Anicura Istituto Veterinario Novara Novara Italy

Cardiomyopathies are frequently encountered in cats and the evaluation of left atrial size is important because offer diagnostic and prognostic information. Echocardiographic assessment of left atrial diameter (LAD) from right parasternal long axis view is frequently used to estimate left atrial size in dogs and cats. LAD is demonstrated to be correlated to body weight (BW) in dogs and cats. Reference ranges of normalized LAD to BW (LADn) and to aorta (LAD:Ao) exists for dogs but has not yet been described in a large feline population. The aim of this study was to investigate the correlation of LAD to BW in a significant sample of healthy cats providing normal reference range for LADn and LAD:Ao and investigating their cut‐offs for the prediction of left‐sided congestive heart failure (L‐CHF) in cats affected by cardiomyopathy. This was a retrospective, observational study. Medical records were reviewed for cats who had performed a complete echocardiographic examination. Cats were divided into three groups: healthy cats (H), asymptomatic cats with cardiomyopathy (CM) and cats with cardiomyopathy and L‐CHF (CM‐CHF). The following echocardiographic variables were gathered: LAD measured from the right parasternal four‐chamber long axis view and the aortic annulus measured from the right parasternal five‐chamber long axis view. The study enrolled 483 cats, 205 females and 278 males, with a median age of 8 years (range, 1‐21 years) and a median BW of 4.2 kg (range, 1.8‐12.5 kg), including: 303 H, 109 CM and 71 CM‐CHF. LAD (r^2^=0.32, p<0.001) and Ao (r^2^=0.32, p<0.001) presented a positive linear correlation with BW. According to allometric scales the formula to calculate LADn was: LAD/kg^0.19^. In the H group the median LADn was 10.7 (95% prediction intervals for LADn between 8.5 and 12.8) and LAD:Ao reference range was between 1.55 (90% CI 1.51‐1.59) and 2.47 (90% CI 2.4‐2.5). LAD >17.3 mm (AUC=0.94, sensitivity (Se) 88%, specificity (Sp) 94%), LADn >13 (AUC=0.97, Se 92%, Sp 91%) and LAD:Ao >2.5 (AUC=0.97, Se 89%, Sp 94%) predict L‐CHF in cats. LADn and LAD:Ao presented excellent diagnostic accuracy for the identification CM‐CHF cats. This study, for the first time provides normal values of LADn and LAD:Ao in a large sample of healthy cats and identifies their cut‐offs for predicting L‐CHF overcoming the limitation of the association between LAD and BW which may be useful especially in small and large cats.

**Disclosures**

No disclosures to report

## 111 ESVC‐P‐2

### 111.1 ESVC ‐ European Society of Veterinary Cardiology

#### 111.1.1 Evaluation of the "Eko DUO" device in dogs and cats: a new smartphone‐based digital stethoscope paired with phonocardiography and electrocardiography

##### 111.1.1.1 T. Vezzosi, L. Alibrandi, G. Grosso, R. Tognetti

###### 111.1.1.1.1 University of Pisa San Piero a Grado, Pisa Italy

Digital stethoscopes and smartphone‐based electrocardiographic (ECG) technologies have been developed in both human and veterinary medicine, especially because of their promising characteristics for telemedicine, teaching activity and cardiologic monitoring over time. The new Eko DUO device is a digital stethoscope paired with simultaneous phonocardiographic and 1‐lead electrocardiographic recording. It allows to store, review, and analyse the recordings on smartphone devices and to share them in telemedicine. The aim of this study was to compare audio recordings and ECG tracings obtained using the Eko DUO device with conventional auscultation and standard 6‐lead ECG in dogs and cats.

A total of 99 dogs and 9 cats were prospectively included in this observational study. All cases underwent conventional auscultation using an acoustic stethoscope, standard 6‐lead ECG, standard echocardiography and recording via the Eko DUO device. Thereafter, all the audio recordings, the phonocardiographic tracings and the ECG tracings were blinded reviewed by an expert operator. The agreement between the standard methodologies and the Eko DUO device was assessed using the Cohen's kappa and the Bland‐Altman test.

Audio recordings were deamed interpretable in 98% of cases. Substancial agreement was found in the diagnosis of heart murmur (κ=0.685) and gallop rhythm (k=0.741). In 9 cases with an echocardiographic diagnosis of heart disease (3 myxomatous mitral valve disease, 2 pulmonary hypertension, 1 aortic stenosis, 1 dilated cardiomyopathy and 2 hypertrophic cardiomiopathies) only digital stethoscope detected heart murmur or gallop rhythm. ECG tracings recorded with the Eko DUO device were deamed interpretable in 87% of cases. Good agreement in the assessment of heart rate was observed between the two methods (bias=8 bpm). Diagnosis of heart rhythm showed moderate agreement (k=0.554), with a sensitivity of 100% and a specificity of 90% in the identification of atrial fibrillation. The detection of ventricular premature complexes and bundle branch blocks revealed an almost perfect agreement (k=0.838 and k=1, respectively). Substantial agreement was found in the assessment of QRS polarity in comparison to lead II on standard ECG (k=0.614).

In the present study, the Eko DUO device showed a good diagnostic accuracy in the detection of heart murmurs, gallop rhythm and arrhythmias in dogs and cats, with a sensitivity possibly higher than acoustic stethoscope. The ability to archive and replay recordings can allow to review and monitor cardiac condition over time by an expert operator. Therefore, the Eko DUO device could also represent an interesting tool for the development of veterinary telemedicine.

**Disclosures**

No disclosures to report

## 112 ESVC‐P‐3

### 112.1 ESVC ‐ European Society of Veterinary Cardiology

#### 112.1.1 MiR‐146a may be a new biomarker of left ventricle remodeling in dogs with myxomatous mitral valve disease

##### 112.1.1.1 A Reis‐Ferreira ^1^, J Neto‐Mendes ^2^, C Brás‐Silva ^3^, L Lobo ^4^, A Fontes‐Sousa ^5^

###### 112.1.1.1.1 ^1^ Hospital Veterinário do Porto, Instituto de Ciências Biomédicas Abel Salazar UP Porto Portugal; ^2^ Instituto de Ciências Biomédicas Abel Salazar da Universidade do Porto, ICBAS‐UP Porto Portugal; ^3^ UnIC, Faculdade de Medicina, Universidade do Porto Porto Portugal; ^4^ Hospital Veterinário do Porto, FMV ‐ Universidade Lusófona, CECA ‐ UP Porto Portugal; ^5^ Instituto de Ciências Biomédicas Abel Salazar, MedInUP, UPVET Porto Portugal

Myxomatous mitral valve disease (MMVD) is the most common acquired cardiac disease in dogs. Circulating miRNAs are emerging as potential biomarkers for diagnosis, prognosis, monitorization and as therapeutical targets in cardiovascular diseases. It is thought that miR‐146a could play a role in preventing heart diseases associated with inflammation and cardiac remodeling. So, the aim of the present study was to evaluate the potential of circulating miR‐146a as a biomarker of cardiac remodeling in dogs with MMVD. Twenty‐four dogs of both genders were included in this study, with a median age and body weight of 12 years and 10.050 Kg, respectively. The animals were divided in groups according to the ACVIM MMVD staging [control, stage B1, stage B2 or stage C] and according to the presence or absence of left cardiac remodeling [normal left atrium (LA), LA enlargement (LAE), normal left ventricle (LV), LV enlargement (LVE), normal LA+LV or LAE+LVE]. Total RNA was extracted from plasma samples, and reverse transcription and qRT‐PCR were performed to estimate relative miR‐146a levels.

Circulating miR‐146a was significantly elevated (p < 0.001) in MMVD group with LVE when compared to those with normal sized cardiac chamber (LV and LA+LV). Additionally, LV enlargement correlated positively with miR‐146a plasma expression levels (p < 0.05). Regarding all studied groups, the highest relative miR‐146a expression was observed in the ACVIM stage C, LVE and LAE+LVE groups.

In conclusion, the current study suggests that miR‐146a might be a marker of LV remodeling in canine MMVD. Further large clinical trials will be needed to evaluate the real diagnostic relevance of this miR and its clinical relevance.

**Disclosures**

No disclosures to report

## 113 ESVC‐P‐4

### 113.1 ESVC ‐ European Society of Veterinary Cardiology

#### 113.1.1 Assessment of pulmonary hypertension in dogs with heartworm by using the ratio between the right and left ventricle

##### 113.1.1.1 J.I. Matos, Y Falcón, S García, N Costa, E Carretón, JA Montoya

###### 113.1.1.1.1 University of Las Palmas of Gran Canaria Las Palmas de Gran Canaria Spain

The infection caused by *Dirofilaria immitis* causes an increase in resistance to blood flow, which causes an increase in pressure in the pulmonary arteries and, consequently, the right ventricular cavity undergoes morphological changes that ultimately lead to the appearance of signs of congestive heart failure. Currently, thoracic computed tomography (CT) is an alternative modality to the use of transthoracic echocardiography (TTE) to evaluate lesions caused by *D. immitis*. The measurement of ventricular diameters expressed in the ratio between the right ventricle and left ventricle (RV:LV) has been used in human medicine and recently in veterinary medicine to estimate pulmonary hypertension (PH) of different origins. The purpose of this study was to quantify the RV:RL ratio in dogs suffering from *D. immtis*, in order to assess the potential use of this measure in the diagnosis of PH in dogs with heartworm.

Thoracic CT scans with contrast were performed in 31 owned‐dogs with heartworm, diagnosed by presence of circulating *D. immitis* antigens. There were 16 male and 15 female, aged mean of 8,77 years and body weight ranged from 4.5‐37.2 kg. A total of 15 different dog breeds were included. The presence or absence of PH was determined using TTE, based on the determination of the right pulmonary artery distensibility index (RPADi) as previously described and validated in heartworm disease. An RPADi <29.5% correlated with presence of PH (>50mmHg). The RV:LV ratio was determined following previously established protocols. The diameters of the right and left ventricles were obtained from acquired CT transverse images. The right ventricle and the left ventricle were measured at their largest diameter on the same axis. PH was echocardiographically present in 64.5% of the dogs, showing a mean RV:LV ratio of 0.94 ± 0.61 (99% CI: 0.83–1.22). Normotensive dogs (35.5%) had a mean RV:LV ratio of 0.62 ± 0.46 (99% CI: 0.33–0.81). Additionally, a high correlation was observed between RPADi and RV:LV in the dogs studied (R^2^>0.75) (P<0.05).

The results showed significant differences in the RV:LV ratio between the presence or absence of PH in dogs with *D. immitis*, and demonstrate that the measurement can be a useful tool to assess PH in patients with heartworm disease in images obtained by CT. New studies are indicated with a larger sample size, being able to standardize protocols and obtain reliable reference values ​​to establish the presence and severity of PH in these dogs.

**Disclosures**

No disclosures to report

## 114 ESVC‐P‐5

### 114.1 ESVC ‐ European Society of Veterinary Cardiology

#### 114.1.1 A modified subcostal echocardiographic view to assess patent ductus arteriosus size in dogs

##### 114.1.1.1 F. Marchesotti ^1^, T. Vezzosi ^2^, M. Croce ^1^, V. Patata ^1^, M. Bini ^1^, O. Domenech ^1^

###### 114.1.1.1.1 ^1^ Anicura Istituto Veterinario di Novara Granozzo con Monticello Italy; ^2^ University of Pisa Pisa Italy

Patent ductus arteriosus (PDA) is one of the most common congenital heart disease in dogs. Assessment of PDA minimal ductal diameter (MDD) and ampulla diameter (AD) is critical for appropriate device selection in case of minimally‐invasive PDA occlusion. Transthoracic echocardiography is commonly used for preliminary measurements of MDD and AD. We aimed to describe a modified subcostal echocardiographic view (SUBV) for the evaluation of PDA dimensions in dogs.

This was a retrospective observational study including dogs affected by PDA. The MDD and the AD were measured from the right parasternal short‐axis view (RPSAV) at the heart base, from the left cranial short‐axis view (LCSAV), and from the SUBV. The SUBV was obtained starting from the conventional subcostal five‐chamber view, tilting the probe and orienting the echo beam toward the left hemithorax until the appearance of the main pulmonary artery and the PDA. The measurement agreement between the SUBV and the conventional RPSAV and LCSAV was assessed using the Bland‐Altman analysis.

This study enrolled 45 dogs, 32 females and 13 males, with a median age of 5 months (range, 2 to 61 months) and a median body weight of 10.4 kg (range, 1.4 to 33 kg). The median MDD was 3.2 mm (range, 2.0 to 7.5 mm) from RPSAV, 3.1 mm (range, 1.5 to 7.4 mm) from LCSAV and 3.0 mm (range, 1.5 to 7.5 mm) from SUBV. Mean AD was 8.7 ± 3.4 mm from RPSAV, 8.5 ± 3.2 mm from LCSAV and 8.0 ± 3.0 from SUBV. Regarding MDD measurement, the SUBV showed a bias of 0.24 mm (95% limit of agreement, between ‐0.94 and 1.43 mm) in comparison to the RPSAV and a bias of 0.02 mm (95% limit of agreement, between ‐0.79 and 0.75) in comparison to LCSAV. Regarding AD measurement, the SUBV showed a bias of 0.35 mm (95% limit of agreement, between ‐2.35 and 3.06 mm) in comparison to the RPSAV and a bias of 0.42 mm (95% limit of agreement, between ‐2.26 and 3.10) in comparison to LCSAV. Intraobserver and interobserver measurement variabilities for the SUBV were 3.8% and 12.5% for the MDD, and 3.9% and 5.5% for the AD, respectively.

In conclusion, the SUBV yielded clinically similar values of MDD and AD in comparison to the standard RPSAV and LCSAV. Thus, it can be used as an alternative complementary view for the preliminary echocardiographic assessment of PDA size in dogs.

**Disclosures**

No disclosures to report

## 115 ESVC‐P‐6

### 115.1 ESVC ‐ European Society of Veterinary Cardiology

#### 115.1.1 Aldosterone‐progesterone relationship in sexually intact Chihuahua bitches

##### 115.1.1.1 A. Galizzi ^1^, G. Dossi ^2^, V. Borromeo ^2^, P. Pocar ^2^, G. Pizzi ^2^, M. Bagardi ^2^, S. Ghilardi ^2^, P. Brambilla ^2^, C. Locatelli ^2^

###### 115.1.1.1.1 ^1^ University of Milan Lodi Italy; ^2^ University of Milan Milano Italy

Chronic renin‐angiotensin‐aldosterone system (RAAS) activation promotes and perpetuates the heart failure syndrome. Aldosterone represents the last effector of the cascade and can be a good indicator of RAAS activity. However, aldosterone levels could be difficult to interpret because of the influence of individual factors (ie, breed and gender), as suggested by a recent study. In particular, Chihuahua and sexually intact bitches showed the highest values of urinary aldosterone‐to‐creatinine ratio (UAldo:C) among breeds and genders, respectively. Aldosterone has been proved to rise during the luteal phase of menstrual cycle in women. In humans and rats, progesterone has shown an antimineralocorticoid effect, binding to mineralocorticoid receptors. Even a direct stimulation of aldosterone secretion by progesterone has been suggested. In dogs, the aldosterone‐progesterone relationship has not yet been investigated.

The aim of this study was to investigate the relationship among serum progesterone, serum aldosterone, urinary aldosterone, UAldo:C and plasma NT‐proBNP in sexually intact Chihuahua bitches, measuring them in anoestrus and mid‐dioestrus. Dogs enrolled in this prospective study were recruited among breeding dogs referred to the Veterinary Teaching Hospital for cardiological and reproductive screening/monitoring. All dogs underwent physical examination, systemic blood pressure measurement, vaginal cytology, CBC, biochemistry profile, urinalysis, echocardiography and hormones measurement during anoestrus. In mid‐dioestrus, urinalysis, vaginal cytology and hormones assessment were repeated. Aldosterone and NT‐proBNP (IDEXX Cardiopet® proBNP) were measured by ELISA kits; progesterone was measured by a MINI‐VIDAS® analyzer (bioMérieux). Hormones were assessed on left‐over samples.

Fourteen sexually intact Chihuahua females were included (11 healthy and 3 affected by mitral valve disease ACVIM stage B1). Mean (±SD) age was 3.71±1.55 years and mean body weight was 2.82±0.41 kg. Median (IQR) serum progesterone was significantly higher in mid‐dioestrus compared to anoestrus (34.06 [21.17‐44.90] vs 0.19 [0.13‐0.38] ng/ml; p=0.001), as well as median serum aldosterone (124.88 [108.57‐187.75] vs 77.23 [55.87‐102.56] pg/ml; p=0.001) and mean urinary aldosterone (9887±5735 vs 5006±2128 pg/ml; p=0.01). Median UAldo:C was higher in mid‐dioestrus compared to anoestrus (4.16 [3.17‐6.80] vs 3.08 [2.33‐4.22] μg/g; p=0.056). In the overall population (anoestrus+mid‐dioestrus) serum progesterone showed a positive moderate correlation with serum aldosterone (ρ=0.532, p=0.004), urinary aldosterone (ρ=0.638, p<0.0001) and UAldo:C (ρ=0.516, p=0.005).

These results strongly suggest the existence of a progesterone‐aldosterone relationship in canine species. In particular, an increase in serum progesterone seems to induce an increase in aldosterone concentrations in both serum and urine. Thus, reproductive phase should be taken into account when evaluating aldosterone levels in female dogs.

**Disclosures**

Disclosures to report, please report below

Drs. Locatelli have received funding from CEVA within the last 1 year for all of the following activities: research, speaking fees, consultancy fees, and preparation of educational materials. Drs. Bagardi have received funding from Esaote and Purina within the last 1 year for the following activities: speaking fees.

## 116 ESVC‐P‐7

### 116.1 ESVC ‐ European Society of Veterinary Cardiology

#### 116.1.1 Cardiac troponin I as a prognostic marker for dogs admitted to the emergency service.

##### 116.1.1.1 P. Peters ^1^, S. Cortellini ^2^, D. .J. Connolly ^2^

###### 116.1.1.1.1 ^1^ Potters Bar United Kingdom; ^2^ Royal Veterinary College Hatfield United Kingdom

Cardiac troponin I (cTnI) is known to provide additional prognostic information to acute patient physiologic and laboratory evaluation (APPLE) scores in dogs with systemic inflammation. In a heterogenous population of dogs presentating to the emergency service (ES), cTnI alone has also been shown to have prognostic use. Further studies evaluating cTnI and APPLE scores in a similar population are lacking. The study aim was to identify myocardial injury in a heterogenous population of dogs admitted to the ES and determine whether cTnI could predict outcome in these dogs alone and when used in conjunction with APPLE scores in both primary cardiac and non‐cardiac cases. Client owned dogs admitted to the ES of a referral hospital between 01.01.2017‐31.12.2019 were included in this single center retrospective study. Patients were classified into cardiac or non‐cardiac cohorts depending on the underlying disease. Serum cTnI and APPLE_FULL_ and APPLE_FAST_ scores were analysed if data was collected and cTnI measured within 24 hours of admission. cTnI fold change was calculated and used for all further statistical analysis. Prognostic capabilities of cTnI and APPLE scores for survival to discharge were evaluated using simple and multivariate logistical regression and receiver operating characteristic (ROC) curve analysis. Survival to discharge was 72% (85/118 dogs). Survivors (median) had a significantly lower cTnI fold change (3.88) than non‐survivors (80.5). Survivors (mean) also had significantly lower APPLE full (28.77) and fast scores (22.58) than non‐survivors (35.52 and 26.62) respectively. No significant difference in medians of cTnI fold change was found between the cardiac and non‐cardiac group. cTnI and APPLE scores were all significant predictors of survival, as measured by simple logistical regression. No significant association was found between cTnI and survival in the non‐cardiac group, preventing further analysis by multivariate models. cTnI contributed to the APPLE_FULL_ and APPLE_FAST_ scores by increasing the prediction power of the overall models as measured by R^2^, which increased by 17.7% and 16.7% respectively. The ROC‐AUC (95% CI) for APPLE_FULL_ and APPLE_FAST_ increased from 0.7146 (0.5961‐0.8332) and 0.7032 (0.5731‐0.8333) to 0.7233 (0.6067‐0.8400) and 0.7199 (0.5916‐0.8481), respectively. A significant association was also found between cTnI and the presence of arrhythmia; simple linear regression (R^2^ = 0.043, standardized coefficient Beta = 0.207, P = 0.025). This study demonstrates that cTnI aids prediction of short‐term outcome in a heterogenous population of dogs presented to the ES. When added to the APPLE scores, cTnI increases the overall predictive utility for outcome.

**Disclosures**

Disclosures to report, please report below

David J. Connolly's research is supported by the PetPlan Charitable Trust, The Cat Welfare Trust, The EveryCat Health Foundation and BBSRC.

## 117 ESVC‐P‐8

### 117.1 ESVC ‐ European Society of Veterinary Cardiology

#### 117.1.1 Echocardiographic variables predicting patent ductus arteriosus minimal ductal diameter and ampulla dimension in dogs

##### 117.1.1.1 M. Bini

###### 117.1.1.1.1 Anicura Istituto Veterinario Novara Granozzo con Monticello (NO) Italy

Diagnostic assessment of patent ductus arteriosus (PDA) is mainly based on transthoracic echocardiography (TTE), which allows the evaluation of cardiac size and PDA dimension. PDA minimal ductal (MDD) and ampulla diameters (AD) are particularly important for device selection for PDA interventional closure. The aim of this study was to investigate the correlation between MDD and AD with echocardiographic measurements in order to determine if some TTE variables might be useful in the prediction of PDA size and subsequently Amplatz Canine Duct Occluder (ACDO) dimension.

This was a retrospective observational study. Medical records were reviewed for dogs with PDA that had undergone complete occlusion with ACDO. MDD and AD obtained by TTE using left parasternal short‐axis view were gathered. The following TTE measurements were included: left atrium to aorta ratio (LA/Ao), left atrial diameter normalized to body weight (BW) (LADn), Simpson's method of discs derived left ventricle end‐diastolic volume indexed to body surface area (LVEDV‐I) (ml/m^2^) and left ventricle end‐systolic volume indexed to body surface area (LVESV‐I ml/m^2^), normalized left ventricular internal diameter in diastole (LVIDDn) and systole (LVIDSn), transmitral E and A waves velocities, aortic (AVmax) and pulmonary (PVmax) peak velocities.

Twenty‐eight dogs were included, 22 females and 6 males, with a median age of 0.8 years (0.2–5 years) and a median BW of 13 kg (4.1–33.7 kg). A strong linear correlation was found between MDD and LVESV‐I ml/m^2^ (r=0.80; p<0.001). MDD was moderately correlated with LVEDV‐I ml/m^2^ (r=0.68; p<0.001), LA/Ao (r=0.57; p=0.001), LVIDSn (r=0.56; P=0.002), LADn (r=0.55; P=0.002), Emax (r=0.51; P=0.005) and LVIDDn (r=0.50; p=0.006). AD was moderately correlated with LVESV‐I ml/m^2^ (r=0.64; p<0.001), Emax (r=0.54; p=0.003), LVEDV‐I ml/m^2^ (r=0.53; p=0.004) and LADn (r=0.52; p=0.005). According to multiple linear regression analysis the formulas to estimate the MDD and AD were the following: MDD=2.53+(0.04*LVESV‐I ml/m^2^); AD=5.8+(0.06*LVESV‐I ml/m^2^).

The ACDO diameter to MDD ratio (oversize factor) was then calculated dividing the ACDO dimension used for the complete occlusion of the PDA to the TTE‐measured MDD and to the formula‐derived MDD.

The oversize factors calculated with the TTE‐measured MDD (1.61 ±0.24) were not significantly different from ones obtained with the formula‐derived MDD (1.56 ±0.33) (p=0.147).

In conclusion, PDA size is correlated with standard echocardiographic measurements in dogs. Moreover, the equations provided in this study might be used to predict MDD and AD which may contribute to select ACDO device dimension needed to fully occlude the PDA.

**Disclosures**

No disclosures to report

## 118 ESVC‐P‐9

### 118.1 ESVC ‐ European Society of Veterinary Cardiology

#### 118.1.1 hypertrophic cardiomyopathy is not the sole echocardiographic phenotype associated with feline hyperthyroidism: a retrospective study on 107 cats (2005‐2020).

##### 118.1.1.1 P. Foulex, B Reslinger, L Desquilbet, M Kurtz, E Trehiou, C Poissonnier, P Passavin, G Benchekroun, V Chetboul

###### 118.1.1.1.1 École Nationale Vétérinaire d'Alfort Maisons‐Alfort France

Hyperthyroidism, the most commonly diagnosed endocrinopathy in geriatric cats, is frequently associated with cardiac abnormalities, potentially leading to congestive heart failure. One study, including 91 pretreated hyperthyroid cats, observed an increase in end‐diastolic interventricular septal (IVSd), and left ventricular (LV) free wall (LVFWd) thicknesses (also defined as hypertrophic cardiomyopathy phenotype, HCMP), with reversion toward normal values following radioiodine administration. Apart from the hyperthyroid‐induced HCMP, restrictive cardiomyopathy phenotype (RCMP) may be associated with feline hyperthyroidism. However, to the best of the authors’ knowledge, no study has confirmed the latter hypothesis.

The aims of this retrospective study were: 1) to describe the echocardiographic abnormalities and cardiomyopathy phenotypes associated with feline hyperthyroidism, 2) to compare echocardiographic features of hyperthyroid cats with HCMP with those from a contemporary age‐ and body‐weight matched control group of cats with primary HCM (n=140). Among the 195 cats diagnosed with hyperthyroidism during the study period, 107 were retrospectively included: the diagnostic of hyperthyroidism was established less than 3 months before or after echocardiographic examination (median age at presentation=14.2 years; interquartile range (IQR)=[12.6‐15.9]; body weight=3.9 kg [2.9‐4.8]; total T4=98.5 nmol/L [74.7;138.0]). An abnormal LV myocardial phenotype was diagnosed in most hyperthyroid cats (88/107, 82%), including HCMP in 74/88 (84%) cats, RCMP in 11/88 (13%) cats, and nonspecific phenotypes in 3/88 (3%) cats. Among the 74 HCMP cats, 50 (68%) showed symmetrical LV hypertrophy (0.7<IVSd‐to‐LVFWd ratio<1.3), 13 (18%) IVS hypertrophy only, 3 (4%) LVFW hypertrophy only, and 8 (11%) focal sub‐aortic IVS (SaIVS) hypertrophy only.

End‐diastolic LV diameter was significantly higher (p<0.01) in cats with hyperthyroidism‐associated HCMP (14.7 mm [13.3‐16.3], n=74) than in cats with primary HCM (13.5 mm [11.9‐15.3], n=103). Additionally, focal SaIVS hypertrophy was significantly (p<0.01) more common in HCM cats (102/117, 87%) than in hyperthyroidism‐associated HCMP cats (33/74, 46%), the SaIVS being significantly thicker (p<0.01) in cats with primary HCM (7.0 mm [6.3;7.8]) than in cats with hyperthyroidism‐associated HCMP (5.7 mm [5.2;6.9]).

Lastly, end diastolic left atrium‐to‐aorta ratio and early‐to‐late diastolic mitral flow velocities of the 11 RCMP cats were 1.67 [1.49‐2.03] and 2.29 [2.21‐2.73], respectively.

In conclusion, these results confirm that HCMP is the predominant hyperthyroidism‐associated phenotype, with more LV enlargement and fewer sub‐aortic IVS hypertrophy than in primary feline HCM, other phenotypes including RCMP and nonspecific phenotypes representing 16% of all abnormal LV geometric patterns.

**Disclosures**

No disclosures to report

## 119 ESVC‐P‐10

### 119.1 ESVC ‐ European Society of Veterinary Cardiology

#### 119.1.1 Ventricular pre‐excitation in cats: 17 cases

##### 119.1.1.1 N. Schreiber ^1^, M. Sidler ^1^, G. Santarelli ^2^, A. Kovacevic ^3^, J. Novo Matos ^4^, M. Baron Toaldo ^1^

###### 119.1.1.1.1 ^1^ Vetsuisse Faculty, University of Zurich Zurich Switzerland; ^2^ University of Utrecht Utrecht Netherlands; ^3^ Vetsuisse Faculty, University of Bern Bern Switzerland; ^4^ University of Cambridge Cambridge United Kingdom

Atrioventricular accessory pathways are abnormal electrical connections between atria and ventricles that predispose to ventricular pre‐excitation (VPE) and reentrant tachycardias. While extensively described in humans and dogs, clinical features related to atrioventricular accessory pathways are poorly documented in cats. In this multicenter study, we aimed to retrospectively analyze the clinical and imaging findings of cats with an ECG diagnosis of VPE. Data obtained from the electronic database of 4 institutions included: signalment, history, clinical presentation, ECG and Holter findings, echocardiographic and radiographic data, treatment, and outcome. A control group of clinically healthy cats was selected to compare the echocardiographic measurements. Seventeen cats with VPE were identified. There were 16 males and one female, breeds included mainly European shorthair cats (11/17), mean age and body weight were 7.1 years and 4.7 kg in the study cats, and these did not differ from the control cats (P=0.832, P=0.688, respectively). Clinical signs included mainly apathy, syncope, tachypnea, while in two cases presented with trauma, VPE was an incidental finding. Two cats had left sided congestive heart failure at presentation. On ECG and Holter all cats had at least one episode of VPE, 9/17 cats showed tachyarrhythmias, 7/9 cats had narrow‐QRS‐complex tachycardia, and 2/9 had wide‐QRS‐complex tachycardia. Three cats had variables degrees of ventricular arrhythmias (other than tachycardia). Drugs mainly used, alone or in combination, included sotalol (5/17 cats), diltiazem (5/17 cats), atenolol (4/17 cats), antithrombotics/anticoagulants (4/17 cats) and furosemide (4/17 cats). Cardiac troponin I was measured in 6/17 cats, and it was elevated in 4/6. Among 8/17 cats receiving an antiarrhythmic treatment, a clinical improvement was observed in 5/8. Cats with VPE had larger maximal left (P<0.001) and right (P<0.001) atrial diameters, and thicker end‐diastolic interventricular septum (P=0.019) and left ventricular free wall (P=0.028) compared to controls. Three cats had a maximal end‐diastolic left ventricular wall thickness >6 mm. Two cats didn't have an echocardiography performed. Five cats died during the follow‐up time, all for cardiac reasons (median survival time 1,682 days [2‐1,882]). The remaining 12 cats were still alive with a median follow‐up time of 567 days (20‐2,390).

VPE is an uncommon condition in cats. Cats with VPE tend to have larger atria and thicker left ventricular walls compared to healthy subjects. Due to the variety of presentations, a specific treatment must be chosen for each case. Survival time is relatively long, and death occurs for cardiac reasons.

**Disclosures**

No disclosures to report

## 120 ESVC‐P‐11

### 120.1 ESVC ‐ European Society of Veterinary Cardiology

#### 120.1.1 Echocardiographic changes in puppies of dogs under 10 kg

##### 120.1.1.1 M. Huk ^1^, N. Re ^2^, P. Batista ^3^, D.O. Arias ^2^, C. Gobello ^3^, P.G. Blanco ^3^

###### 120.1.1.1.1 ^1^ National University of La Plata & CONICET La Plata Argentina; ^2^ Faculty of Veterinary Sciences La Plata Argentina; ^3^ Faculty of Veterinary Sciences & CONICET La Plata Argentina

Echocardiographic changes in growing puppies have only been reported in a few large breeds. The aim of this study was to describe the morphological and functional echocardiographic changes in puppies of dogs under 10 kg (adult body weight).

Twelve healthy, 2‐month‐old, pure and mixed‐bred, weighing 1.9±0.5 (1.3‐2.5) kg, client‐owned puppies were evaluated every 2 months up to one year of age. At each time points, body weight and echocardiographic evaluations ‐two‐dimensional, M‐mode, and Doppler‐ were performed using a 10 MHz transducer (Toshiba Nemio XG, Japan), without sedation. To minimize variability, three consecutive measurements of each variable were recorded by a single trained operator. Repeated measures ANOVA followed by Tukey test was carried out to evaluate all the variables (SPSS 22.0; SPSS, Chicago, IL, USA).

In these puppies, body weight increased up to 10 months of age (P<0.01). At the end of the study, the dogs weighed 6.5±2 (4‐10.4) kg. In these animals, interventricular septal thickness at end‐diastole, left ventricular free wall thickness at end‐diastole and interventricular septal thickness at end‐ systole increased from 2 months to 4 months of age (P<0.01). Left ventricular internal dimension at end‐systole (P<0.01), ejection time (P<0.01) and left ventricular free wall thickness at end‐systole (P<0.01) augmented up to 6, 8 and 10 months of age, respectively. Left ventricular internal dimension at end‐diastole (P<0.01), left atrium (LA) diameter (P<0.01), LA area (P<0.01), diameter of the aortic root (P<0.01), stroke volume (P<0.01), cardiac output (P<0.01) and mitral annulus (P<0.01) increased from 2 months to 12 months of age (P<0.01). Conversely, heart rate decreased from 2 months to 8 months of age (P<0.01). Fractional shortening, LA‐to‐aortic root ratio, aortic peak systolic velocity, peak velocities of early (E) and late (A) diastolic transmitral flow, E/A ratio and E wave deceleration time did not change throughout the study period (P>0.05).

In these puppies of small‐sized dogs, morphological and functional echocardiographic parameters changed during the first year of life. These results will not only improve the cardiac evaluation of growing canine patients but also the early diagnosis of congenital heart diseases in this species.

**Disclosures**

No disclosures to report

## 121 ESVC‐P‐12

### 121.1 ESVC ‐ European Society of Veterinary Cardiology

#### 121.1.1 Effects of coenzyme Q10 supplementation on oxidative stress markers, inflammatory markers, lymphocyte subpopulations, and clinical status in dogs with myxomatous mitral valve disease

##### 121.1.1.1 N. Druzhaeva ^1^, A. Nemec Svete ^2^, G. Tavčar‐Kalcher ^2^, J. Babič ^2^, A. Ihan ^3^, K. Pohar , U. Krapež ^2^, A. Domanjko Petrič ^2^

###### 121.1.1.1.1 ^1^ Small Animal Clinic , University of Ljubljana Ljubljana Slovenia; ^2^ Veterinary faculty Ljubljana Slovenia; ^3^ Faculty of Medicine Ljubljana Slovenia

There are limited data on the effects of coenzyme Q_10_ (CoQ_10_) supplementation in dogs with myxomatous mitral valve disease (MMVD). The aim of this study was to evaluate the effect of 3‐month supplementation of water‐soluble CoQ_10_ on oxidative stress markers (glutathione peroxidase, F2‐isoprostanes), inflammatory markers (TNF soluble receptor II, leukocytes, and their subtypes), lymphocyte subpopulations (T helper and cytotoxic T lymphocytes, including activated T and B lymphocytes), echocardiographic and clinical parameters, and NT ‐proBNP and cardiac troponin I in dogs with MMVD. In this randomized, placebo‐controlled, double‐blind, longitudinal study, 43 MMVD dogs in stages ACVIM B2 (20 dogs) and ACVIM C and D (CHF; 23 dogs) received water‐soluble CoQ_10_ (200 mg daily, divided into 2 doses) or placebo for 3 months; 12 non‐supplemented healthy dogs served as controls. All parameters were measured at baseline and after 3 months of supplementation in the MMVD dogs and at baseline in the group of healthy dogs. The Mann‐Whitney test was used to detect significant differences in the change (final value minus baseline value) of the measured parameters between the placebo and CoQ_10_ groups in both patient groups. The P‐value < 0.05 was considered significant.

In a group of CHF patients, a significantly higher change in CoQ_10_ concentration (P < 0.001) and lymphocytes (% (P=0.044), absolute count (P=0.041)) and a significantly lower change in relative neutrophil count (P=0.041) were found in the CoQ_10_‐supplemented group than in the placebo group. In the ACVIM B2 group, we found only a significantly higher change in CoQ_10_ concentration (P < 0.001) in the CoQ_10_ group than in the placebo group. All other parameters were not affected by CoQ_10_ supplementation in either group.

We conclude that 3 months of CoQ_10_ supplementation had no effect on the measured parameters in ACVIM B2 and CHF patients, except for CoQ_10_ concentration in both groups and neutrophils and lymphocytes in the latter group.

**Disclosures**

No disclosures to report

## 122 ESVC‐P‐13

### 122.1 ESVC ‐ European Society of Veterinary Cardiology

#### 122.1.1 Elevations in serum cortisol in dogs with heartworm disease as a biomarker of stress

##### 122.1.1.1 N Costa‐Rodríguez L Hernández‐Jiménez, Y Falcón‐Cordón, SN García‐Rodríguez, JI Matos, JA Montoya‐Alonso, E Carretón

###### 122.1.1.1.1 Institute for Biomedical and Healthcare Research Gran Canaria (Canary Island) Spain

Cortisol is a glucocorticoid which production and secretion is regulated by the hypothalamus‐pituitary‐adrenal axis (HPA), and is released in response to stress. It has been described that chronic elevations of glucocorticoids can directly influence the impairment of the immune response, being detrimental to the organism. Moreover, high cortisol levels have been associated with infectious diseases, and it has been demonstrated its utility as a biomarker of chronic stress in cardiovascular disease in humans. Furthermore, previous studies relate the presence of parasites and cortisol levels in several species. Therefore, the aim of this study was to evaluate the cortisol levels in dogs with heartworm disease (*Dirofilaria immitis*) in serum from 92 dogs with heartworm. The parasite load was echocardiographically assessed and the presence/absence of microfilariae was determined by using the modified Knott test. Serum cortisol was measured by using VCHECK V200 Veterinary Immunoassay Analyzer (Bionote, Minnesota, USA). Reference ranges for healthy dogs were established as 2.3±1.1 ng/ml. Of the studied dogs, 39.1% were females and 60.9% were males; 54.3% were microfilaremic and 30.4% showed high parasite burden. Mean cortisol levels were 20.2±10.1 ng/ml, being significantly higher than in healthy dogs (p<0.05). No statistically significant differences were found by sex or microfilaremic status, although dogs with high parasite burden showed higher levels of cortisol (p<0.05). These results suggest that *D. immitis* increases the systemic levels of cortisol. Infectious diseases, including parasitic infections, produce a joint action of neuroendocrine and immune networks to facilitate the host response. Immunological changes have been studied in depth; however, the impact on immunoendocrine circuits has been less studied. In canine heartworm, it has been published that the presence of the parasite and its endosymbiotic bacteria *Wolbachia pipientis* stimulate the production of interleukins and other inflammatory mediators. Since other studies have shown that interleukins stimulates the production of C‐reactive protein (CRP) and cortisol in humans, and CRP elevations have been previously described in canine heartworm, the presence of cortisol elevations were to be expected. The results of this study show that cortisol levels are higher in dogs with high parasite load. Similar results have been described for other parasites, being considered as an indicator of severity by some authors. The results encourage continuing studying the presence of disorders in the hypothalamic‐pituitary‐adrenal (HPA) axis with the presence of canine heartworm, in order to determine the usefulness of this glucocorticoid in the management of the disease.

**Disclosures**

No disclosures to report

## 123 ESVC‐P‐14

### 123.1 ESVC ‐ European Society of Veterinary Cardiology

#### 123.1.1 Effects of levomepromazine on electrocardiographic features in propofol and isoflurane‐anesthetized dogs

##### 123.1.1.1 O. Robledo ^1^, M. Tórtora ^1^, M. Marcos ^1^, S. Arioni ^1^, A. Blasco ^1^, P. Batista ^2^

###### 123.1.1.1.1 ^1^ Faculty of Veterinary Sciences La Plata Argentina; ^2^ Faculty of Veterinary Sciences & CONICET La Plata Argentina

Phenothiazines are commonly used in anesthetic premedication. However, the effects of these drugs on electocardiographic parameters have not been reported so far. The objective of this study was to evaluate and compare the effects of two different doses of levomepromazine on electrocardiographic features in propofol and isoflurane‐anesthetized dogs. Eighteen 1‐5 years old healthy bitches from 10‐30 kg derived for ovariohysterectomy were included in this study. In all the animals, primary cardiac diseases were ruled out. The bitches were randomly assigned to one of three groups according to the premedication used: levomepromazine at 0.25 mg/kg IV (L25, n=6), levomepromazine at 0.5 mg/kg IV (L50, n=6) and acepromazine at 0.05 mg/kg IV as the control group (ACE, n=6). All the bitches received tramadol at 3 mg/kg IV. Anesthesia was induced by administering propofol (6 mg/kg IV) followed by a continuous infusion at 25 mg/kg/h, and then they were maintained with isoflurane (2,5%) in oxygen (2 L/min) administered through a circle rebreathing system. Bitches were electrocardiographically evaluated before premedication administration (T0) and 10 minutes (T1) and 45 minutes (T2) following premedication administration; 3 minutes after propofol administration (T3) and 10 minutes after isoflurane administration (T4). Finally, bitches were evaluated 15 minutes after discontinuation of isoflurane (T5). A standardized sedation score based on vocalization, posture, appearance, interactive behaviors, restrainability, noise response and analgesia was assessed at T0, T1 and T2. All variables were analyzed through ANOVA for repeated‐measure followed by Tukey test, while sedation score was analyzed through a Kruskall‐Wallis test. No differences were found in sedation scores at each evaluation point (P>0.1). Interactions between time and group were found for P wave amplitude (Pa; P<0.05) and PR interval (PR; P<0.01). Pa decreased at T1 and then increased at T5 in L50 and ACE, while it remained unchanged in L25. PR showed an increase at T1 in ACE, while it was observed at T3 in L25 and L50. Heart rate (HR) progressively decreased from T0 to T4, followed by an increase at T5 (P<0.01), while QT interval (QT) showed a progressive increase from T0 to T4, followed by a decrease at T5 (P<0.01). No differences were found between groups for these variables (P>0.1). It is concluded that premedication with levomepromazine exerts a decrease of HR and Pa and an increase of PR and QT. However, for the same level of sedation, it has showed a lower impact on Pa and PR than acepromazine.

**Disclosures**

No disclosures to report

## 124 ESVC‐P‐15

### 124.1 ESVC ‐ European Society of Veterinary Cardiology

#### 124.1.1 Prognostic role of clinical and echocardiographic factors in dogs with atrial fibrillation secondary to myxomatous mitral valve disease and dilated cardiomyopathy

##### 124.1.1.1 G. Romito ^1^, D. Darida ^1^, V. Valente ^2^, H. Poser ^2^, B. Contiero ^2^, M. Cipone ^1^, Cc Guglielmini ^2^

###### 124.1.1.1.1 ^1^ University of Bologna Ozzano dell'Emilia Italy; ^2^ University of Padua Padua Italy

Atrial fibrillation (AF) is the most common supraventricular tachyarrhythmia in dogs. In this species, AF is usually secondary to structural heart diseases, namely myxomatous mitral valve disease (MMVD) and dilated cardiomyopathy (DCM). Although the development of secondary AF seems associated with a worse prognosis in the dog, few studies have evaluated the outcome in dogs with AF secondary to heart disease. Therefore, the aim of this study was to investigate the prognostic role of some clinical and echocardiographic parameters in dogs with AF secondary to MMVD or DCM.

For the purpose of this multicenter, retrospective cohort study, medical databases of two Veterinary Teaching Hospitals were reviewed searching for dogs with MMVD and DCM that developed AF. Electrocardiographic and echocardiographic exams were reviewed by two expert cardiologists and various demographical, clinical, and echocardiographic data as well as survival data were retrieved. Independent predictors of death were identified using univariate and multivariable Cox proportional hazards regression models for the endpoint cardiac‐related death and all‐cause mortality.

Fifty‐five dogs with AF were included, 40 (73%) with MMVD and 15 (27%) with DCM. Forty‐seven dogs (85%) died after a median follow‐up time of 219 days (range 1‐994 days), including 38 dogs (81%) and 9 dogs (19%) with cardiac‐related death and non‐cardiac related death, respectively. On multivariable analysis, left ventricular systolic diameter normalized to body weight (LVSDn) (hazard ratio [HR] = 4.41, 95% confidence interval [CI] = 1.18‐16.54; P = 0.028) and heart rate recorded at the time of Doppler mitral inflow analysis (HR‐DMI) (HR = 1.01, 95% CI = 1.00‐1.013; P = 0.041) were predictors of cardiac‐related death. Furthermore, LVSDn (HR = 9.39, 95% CI = 2.49‐35.32; P < 0.001) and HR‐DMI (HR = 1.01, 95% CI = 1.00‐1.013; P = 0.036), as well as age at AF diagnosis (HR = 1.17, 95% CI = 1.04‐1.32; P = 0.008), were also significant predictors of all‐cause mortality.

Increased LVSDn and HR‐DMI represent significant predictors for both cardiac‐related death and all‐cause mortality in dogs with AF secondary to MMVD or DCM.

**Disclosures**

No disclosures to report

## 125 ESVC‐P‐16

### 125.1 ESVC ‐ European Society of Veterinary Cardiology

#### 125.1.1 Usefulness of tissue doppler imaging in the evaluation of pulmonary hypertension in dogs with heartworm

##### 125.1.1.1 J.I. Matos, D Perez, S García, N Costa, JA Montoya, E Carretón

###### 125.1.1.1.1 University of Las Palmas of Gran Canaria Las Palmas de Gran Canaria Spain

Canine heartworm infection can cause a serious and highly fatal disease developed by the nematode *Dirofilaria immitis*. Severe cardiorespiratory clinical symptoms are related to the appearance and persistence of pulmonary hypertension (PH) of precapillary origin. The evaluation of the measurements obtained by transthoracic echocardiographic (TTE) exam offers the most comfortable and useful analysis indexes in canine heartworm disease. However, the hemodynamic functionality through the Tissue Doppler imaging (TDI) study in this disease is unknown. Therefore, the aim was to evaluate the potential use of TDI measurements in the diagnosis of animals suffering from HP caused by *D. immitis*.

To this aim, 75 owned‐dogs infected by *D. immitis* were evaluated. Of them 37 were male and 38 female, aged between 2‐14 years and with a body weight range from 4 to 32 kg. A total of 16 different dog breeds were included. Parasite burden was echocardiographically estimated, and the presence and severity of PH was based on the determination of the right pulmonary artery distensibility index (RPADi) as previously described and validated for canine heartworm. An RPADi <29,5% correlated with presence of PH (>50mmHg). The global TDI index of myocardial function (G‐TDI) was obtained by the determination of the peak myocardial velocities in systole (Stdi), early diastole (Etdi), and late diastole (Atdi), following previously established protocols. The following standard TDI indicators were also determined: isovolumic relaxation time (IVRT), isovolumic contraction time (IVCT), the TDI ratio of right E/A picks, and the right‐ventricle TEI index.

Based on the results obtained in the RPADi, 48% of the dogs were normotensive and 52% presented PH. High parasite burden was present in 62,8 % of the dogs. No statistically significant differences were observed in the TDI measurements regarding sex, age, breed and parasite burden. G‐TDI was 5,1 ± 3,8 cm/s in dogs with PH and 13,3 ± 5,1 cm/s in normotensive dogs and significant differences were reported (P< 0.05). Similarly, significant differences were shown between presence or absence of PH in the study of IVRT (0,09 vs 0,04), IVCT (0,06 vs 0,02), TDI ratio of right E/A picks (0,55 vs 1,06), and the right‐ventricle TEI index (0,53 vs 0,19) (P< 0.05).

The study carried out shows that the measured TDI parameters can be a useful tool to assess PH in canine heartworm. Further studies are indicated with a larger sample size, aimed to determine standardize protocols and obtain reliable reference values for a correct diagnosis.

**Disclosures**

No disclosures to report

## 126 SCH‐P‐1

### 126.1 SCH ‐ Society of Comparative Hepatology

#### 126.1.1 Investigation of the role of bacteria in canine neutrophilic cholangitis using fluorescence in situ hybridisation

##### 126.1.1.1 C. Martinez ^1^, H Jahns ^2^, K Simpson ^3^, EJ O'Neill ^2^

###### 126.1.1.1.1 ^1^ AÚNA Especialidades Veterinarias ‐ IVC Evidensia Valencia Spain; ^2^ University College Dublin Veterinary Hospital Dublin Ireland; ^3^ College of Veterinary Medicine, Cornell University Ithaca United States

Neutrophilic cholangitis (NC) is increasingly identified in dogs. Bacterial infection is often suspected but frequently remains unconfirmed in these cases. The association of NC with gallbladder (GB) disease suggests a common aetiopathogenesis. The aims of the study were to; (1) determine the presence and spatial distribution of bacteria within the hepatic parenchyma of dogs with and without NC using culture‐independent fluorescence *in situ* hybridisation (FISH), (2) to evaluate the presence of bacteria within the GB of dogs with NC, (3) to compare the ability of FISH to identify bacteria to that of cytology and culture.

Archived tissue samples were retrospectively identified (January 2000–May 2019): 32 NC (culture negative n=23, culture positive n=9), 38 other liver diseases (non‐NC: portosystemic shunt n=8, degenerative hepatopathy n=10, hepatic neoplasia n=8, chronic hepatitis n=12), and six normal liver. Twelve dogs with NC (six culture/cytology positive / six culture/cytology negative) had concurrent cholecystitis (n=8) or cystic mucinous hyperplasia (n=4).

FISH positive bacteria were detected in hepatic samples from 8/32 NC, 1/8 portosystemic shunt, 4/10 degenerative hepatopathy, 0/8 hepatic neoplasia, 0/12 chronic hepatitis and 0/5 controls. In 7/8 NC and 5/5 non‐NC these bacteria were restricted to the edge and may represent contamination during sampling. Intrahepatic bacteria were visualised in 1/32 NC as rod‐shaped organisms within dilated luminae; a dog with cholecystitis, visible bactibilia and *Escherichia coli* cultured from GB tissue. In dogs with NC and GB disease, FISH positive bacteria were identified in GB samples from 5/8 dogs with cholecystitis (all culture/cytology positive) and 0/4 with CMH (all culture/cytology negative). Overall, FISH (liver and GB samples) was concordant with culture/cytology in 29/34 samples where comparison was possible. In discordant cases, FISH identified bacteria at the edge of liver tissue in 4 culture/cytology negative samples, while the fifth sample was positive on GB cytology but FISH‐negative. In summary, all samples that were FISH positive within liver parenchyma (n=1: NC) and GB (n=5: cholecystitis) were culture positive or cytology identified bacteria.

In conclusion, intrahepatic bacteria were uncommon in dogs with NC. Bacteria were more frequently identified associated with GB tissue or in bile aspirates than in hepatic biopsies, particularly in dogs with concurrent NC and cholecystitis. Our findings support cytology, culture and antimicrobial sensitivity testing of GB / bile samples to guide antimicrobial therapy in dogs with NC.

**Disclosures**

Disclosures to report, please report below

We would like to acknowledge that this work has been supported by an ECVIM Clinical Studies Fund grant.

## 127 SCH‐P‐2

### 127.1 SCH ‐ Society of Comparative Hepatology

#### 127.1.1 Serum amino acids in canine chronic hepatitis: results in 16 dogs

##### 127.1.1.1 V. Habermaass, E. Gori, F. Bartoli, A. Pierini, C. Mariti, I. Lippi, V. Marchetti

###### 127.1.1.1.1 University of Pisa Pisa Italy

In humans, chronic liver disease may cause alterations in amino acids (AAs) metabolism with serum branched‐chain AAs (BCAAs) decreasing, and aromatic AAs (AAAs) increasing. It results in decreased Fischer ratio (BCAAs/AAAs) and BCAAs/Tyrosine ratio, which are reported to be useful to assess human cirrhosis prognosis. In veterinary medicine, few studies have been performed to evaluate whether serum AAs may vary in chronic hepatopathic patients, and it seems that BCAAs/AAAs tends to reduce only in case of congenital portosystemic shunts.

The aim of this study was to evaluate serum AAs pattern in dogs with chronic hepatitis (CH) compared to a healthy control group.

Leftover serum samples of 16 client‐owned dogs with histological diagnosis of CH (group A) and 25 healthy dogs (group B) were included in this case‐control study. Dogs with AAs supplementation in recent clinical history were excluded. Glycine(GLY), alanine(ALA), valine(VAL), leucine(LEU), isoleucine(ILE), proline(PRO), serine(SER), threonine(THR), cysteine(CYS), methionine(MET), phenylalanine(PHE), tyrosine(TYR), tryptophan(TRP), aspartate(ASP), glutamate(GLU), histidine(HIS), lysine(LYS) and arginine(ARG) were measured with High Performance Liquid Chromatoghaphy (HPLC). Total Serum protein were recorded. Normally distributed data are expressed as mean±SD, whereas non‐normally distributed data as median and range. Unpaired t‐test or Mann‐Whitney U‐test were used to investigate differences between groups based on normality distribution.

Some serum AAs resulted significantly higher in CH dogs than healthy dogs as follows: SER 58.5nmol/ml±23.6 vs 41.3±14.7; GLU median 19.2nmol/ml(4.9‐102.6) vs 8.8(4.4‐20.6); GLY 70.6nmol/ml±21.8 vs 48±16.47; HIS 246.8nmol/ml±82.7 vs 198±58.83.8; PRO median 55.9nmol/ml(22.96‐76.46) vs 6.3(3.9‐67.46); TYR median 20.5nmol/ml(11.42‐67.72) vs 11.3(6.3‐54); VAL median 34.9nmol/ml(17.8‐63.3) vs 4.8(3‐14.2); MET median 8.6nmol/ml(3.4‐36.6) vs 4.8(0.3‐58.5); LYS median 55.9nmol/ml(29.1‐96.4) vs 7.3(3.8‐64.1); LEU median 26.7nmol/ml(8.6‐51.7) vs 10.7(7.8‐34.4); PHE median 25.3nmol/ml(10.4‐63.1) vs 10.9(4.5‐24.6). Contrarily, CYS, ILE (5.8nmol/ml±1.3 vs 38.3±15.3; 9.3nmol/ml±3.6 vs 26.1±8.3, respectively) and TRP (median 19.8nmol/ml(9.9‐33.8) vs 48.2(19.7‐108.3), P value< 0.0001), were lower in group A. BCAAs/AAAs did not significantly differ between the two groups. No difference in serum protein between CH and healthy dogs was found(6.2±1.1 vs 6.4±0.8, respectively).

Several serum AAs concentrations are significantly different between dogs presenting CH and healthy dogs. Many metabolic mechanisms could be involved, as already known for human liver disease. According to human medicine, AAAs seems to increase during CH disease, instead of decreased isoleucine. BCAAs/AAAs ratio not differ significantly from healthy controls. Even if proteinemia did not significantly differ, we observed changes in the proportions of serum AAs between healthy and CH dogs, reflecting qualitative AA imbalances.

**Disclosures**

No disclosures to report

## 128 ESVNU‐P‐1

### 128.1 ESVNU ‐ European Society of Veterinary Nephrology and Urology

#### 128.1.1 Renal hyperparathyroidism and survival in dogs affected by chronic kidney disease

##### 128.1.1.1 J. Zambarbieri ^1^, F. Tagliasacchi ^2^, L. Melatti ^2^, P. Moretti ^2^, A. Giordano ^2^, P. Scarpa ^2^

###### 128.1.1.1.1 ^1^ Veterinary Teaching Hospital ‐ University of Milan Lodi Italy; ^2^ Department of Veterinary Medicine and Animal Sciences ‐ University of Milan Lodi Italy

Renal secondary hyperparathyroidism (RHPT) is a known consequence of chronic kidney disease (CKD) in dogs. However, its role as a prognostic factor has not been clarified yet, both in human and veterinary medicine.

The aim of this study was to evaluate the impact of parathyroid hormone (PTH) serum concentration on survival times in dogs with CKD.

Eighty‐nine privately owned dogs affected by CKD undergoing physical examination, blood works, and urinalysis for diagnostic purposes from January 2019 to December 2021 were included in the study. All the dogs were staged according to International Renal Interest Society (IRIS) guidelines. PTH was measured on left‐over samples by an immunoenzymatic method validated for dogs (ST AIA‐PACK® Intact PTH, Tosoh Bioscience, Tessenderlo, Belgium). Data obtained during their first examination were analyzed and a telephonic follow‐up was obtained by the owner. JMP14 (SAS Inc., Cary, USA) and MedCalc (MedCalc Software Ltd, Ostend, Belgium) were used for statistical analysis.

According to the IRIS CKD staging system, dogs were distributed as follows: 24 (27%) in stage 1, 36 (40.4%) in stage 2, 12 (13.5%) in stage 3, and 17 (19.1%) in stage 4. RHPT was identified in 36% of dogs in stage 1, 44.1% in stage 2, 77.8% in stage 3, and 91.7% in stage 4. RHPT was found in 78.9% of the dead and 29.3% of the alive dogs, respectively. Kruskal‐Wallis test showed a significant difference (p<0.01) between PTH assessment in the different IRIS stages, except between stage 1 and stage 2. At the end of the investigation, 41 (46.1%) dogs were alive, 38 (42.7%) were dead due to CKD and 10 (11.2%) were lost at the follow‐up. Wilcoxon test showed that PTH values were significantly different between dead and alive dogs (p<0.05).

Survival curves were calculated with Kaplan‐Meier analysis. Log‐rank test was used to compare curves. PTH values were previously categorized into 3 groups (<53.6 pg/mL; 53.6‐107.9 pg/mL; >107.9 pg/mL), according to the upper values measured in stages 2 and 3 of CKD. Survival times were significantly different between the groups (p<0.05) and the median survival time was 681, 99, and 6 days, respectively. In the third group, the risk of death was 21.2 and 6.5 times higher than in the first and second group, respectively.

Although different survival times could be related to the disease severity associated with RHPT, serum concentration of PTH has to be considered a factor affecting survival in dogs with CKD.

**Disclosures**

No disclosures to report

## 129 ESVNU‐P‐2

### 129.1 ESVNU ‐ European Society of Veterinary Nephrology and Urology

#### 129.1.1 Prospective testing of a model to predict development of chronic kidney disease in aged cats attending routine veterinarian consultations.

##### 129.1.1.1 I. Gourdon ^1^, S Delmotte ^2^, J Elliott ^3^, M Guignard ^1^, A Feugier ^1^, V Biourge ^1^

###### 129.1.1.1.1 ^1^ Royal Canin SAS Aimargues France; ^2^ Mad‐environnement Nailloux France; ^3^ The Royal Veterinary College London United Kingdom

Chronic kidney disease (CKD) is a common cause of death in senior cats, but early diagnosis is challenging. Previously, models that use three clinical variables (plasma creatinine, BUN and urine specific gravity [USG]) at a single timepoint to predict the development of CKD in aged cats within different time periods were developed and retrospectively validated. This study aimed to test one of these models prospectively – the 12‐month model – in a blinded, multi‐national trial conducted in primary clinical practices. Client‐owned healthy cats aged ≥7 years with no prior diagnosis of CKD, kidney injury or significant chronic conditions were recruited. Model variables at baseline were used to predict CKD status at Month 12. Serum symmetric dimethylarginine (SDMA) was also measured at baseline and Month 18. Practitioners, unaware of the model predictions or SDMA concentrations, assessed clinical and laboratory variables at baseline and Month 12, and determined CKD status, making their clinical diagnoses at Month 12. The performance of the model was measured by sensitivity, specificity, positive predictive value (PPV), negative predictive value (NPV) and area under the receiver operator curve (AUC ROC). As PPV and NPV depend upon prevalence, they were also evaluated *post hoc* for a fixed, hypothetical CKD prevalence of 15%.

The study enrolled 182 cats: median [range] age was 10.1 [7.0–18.5] years; 103 were female (102 neutered) and 79 were male (78 neutered); median body weight was 4.8 [2.5–11.3] kg. Over 12 months, five cats (2.7%) were diagnosed with CKD. The model's sensitivity, specificity, PPV and NPV were 60% (95% confidence interval 20–100%), 97% (94–99%), 43% (10%–77%) and 99% (97–100%), respectively. The AUC ROC was 0.79. When prevalence of CKD was normalized to 15% (more typical of an aged cat population), PPV increased to 80% and NPV decreased to 93%. In a secondary analysis, baseline SDMA concentrations of ≥15 μg/dL correctly predicted only one CKD‐positive cat over 18 months.

We have prospectively tested an existing model for predicting the development of CKD within 12 months using serum creatinine, BUN and USG values at a single visit. The unexpectedly low number of cats developing CKD, probably driven by the relatively low median age of 10 years, reduced the study's power to test the model's PPV. The results indicate that the model retains good accuracy and high NPV under field conditions, making this an effective renal health screening test.

**Disclosures**

Disclosures to report, please report below

Isabelle Gourdon, Maeva Guignard, Alexandre Feugier and Vincent Biourge are employees of Royal Canin SAS, which funded the study. Jonathan Elliott is in receipt of grant funding from Royal Canin SAS. Sebastien Delmotte is a consultant for Royal Canin SAS .

## 130 ESVNU‐P‐3

### 130.1 ESVNU ‐ European Society of Veterinary Nephrology and Urology

#### 130.1.1 Evaluation of preoperative ultrasonographic parameters associated with long‐term renal recovery after treatment of feline unilateral ureteral obstruction

##### 130.1.1.1 D. Pulido Vega ^1^, J. Ficheroulle ^2^, M. Manassero ^1^, J. Mortier ^1^, C. Maurey ^1^

###### 130.1.1.1.1 ^1^ EnvA Maisons Alfort France; ^2^ Université de Liège Liège Belgium

Ureteral obstruction (UO) has been increasingly diagnosed in cats over the past two decades and is most frequently secondary to ureterolithiasis, ureteral strictures and dried solidified blood calculi. The use of a subcutaneous ureteral bypass (SUB) device has been described as an effective way of surgically managing feline UO. Yet this technique comes with risks, expenses and demanding follow‐up and does not always allow a return to a satisfying renal function. Preoperative predictive factors of long‐term renal recovery have not yet been identified. The aim of this study is to identify structural preoperative ultrasonographic (US) parameters associated with a better long‐term recovery of renal function after successful renal decompression with SUB device.

Sixty cats with unilateral benign UO treated with SUB device were included in this retrospective study and medical data was available for each cat. Preoperative US parameters were measured (including renal length, parenchymal thickness, renal pelvic dilation, ureteral dilation) or calculated (including renal parenchymal area, parenchyma to hydronephrosis area ratio and chronic kidney disease (CKD) score). The latter was calculated from the semi‐quantitative assessment of five structural US indicators associated with CKD. Cats with bilateral UO, ureteral rupture or treated as part of a second surgery on UO were excluded. A good renal recovery was defined as serum creatinine inferior or equal to 28 mg/dL at least 3 months postoperatively. Cats with luminal obstruction of SUB device or with contralateral UO during follow‐up were also excluded. Associations between US variables and long‐term renal recovery were examined with univariable analysis.

Forty‐three cats presented a good renal recovery at a median time of 4 months following surgery. A greater parenchymal thickness and a lower CKD score of the kidney contralateral to the UO were associated with a lower long‐term serum creatinine. No preoperative US parameter regarding the obstructed kidney was significantly associated with a better long term renal recovery.

US parameters usually used in humans to predict functional recovery during hydronephrosis (e.g. ratio of the area of renal parenchyma to the area of hydronephrosis) did not allow to predict renal recovery after renal decompression in our study population. Finally, the CKD score of the contralateral kidney is the best correlated with the evolution of renal function postoperatively. A larger population and a multivariable analysis are required in order to draw causal inference.

**Disclosures**

No disclosures to report

## 131 ESVNU‐P‐4

### 131.1 ESVNU ‐ European Society of Veterinary Nephrology and Urology

#### 131.1.1 Comparison of liquid chromatography‐tandem mass spectrometry method and enzyme linked immunosorbent assay for serum symmetric dimethylarginine in elderly dogs

##### 131.1.1.1 S. Marynissen ^1^, G. Junius ^2^, E. Van Den Steen ^2^, L. Patteet ^2^, S. Daminet ^1^, D. Paepe ^1^

###### 131.1.1.1.1 ^1^ Ghent University, Faculty of Veterinary Medicine Merelbeke Belgium; ^2^ MEDVET‐AML, Algemeen Medisch Laboratorium Antwerpen Belgium

Serum symmetric dimethylarginine (SDMA) appears to be of added value combined with serum creatinine to screen for early renal dysfunction in dogs. Liquid chromatography‐tandem mass spectrometry (LC‐MS/MS) is considered the gold standard technique when measuring SDMA. The LC‐MS/MS is however less widely available compared to a frequently used enzyme linked immunosorbent sandwich assay (ELISA).

This prospective study aimed to compare a LC‐MS/MS performed in a commercial laboratory with a routinely used ELISA in elderly dogs.

Serum of elderly dogs undergoing a health check‐up was separated within 2 hours of collection and transported cooled overnight for ELISA SDMA measurement. For the LC‐MS/MS serum was aliquoted and frozen at ‐80°C until batch analysis. Results of the LC‐MS/MS and ELISA were statistically compared using a Wilcoxon signed rank test.

In total, serum of 121 dogs was analyzed. Median storage time until LC‐MS/MS analysis was 3 (1‐19) months. In 3 samples SDMA could not be assessed through ELISA, due to interfering lipemia/hemolysis, so 118 duplicate measurements were available for statistical analysis. Median SDMA was 9.4 (5‐21.2) and 12 (5‐22) μg/dL for LC‐MS/MS and ELISA respectively and a significant difference (p < 0.05) was found between the two methods. In 9 cases (8%) SDMA was above the upper reference limit (>14 μg/dL) for ELISA, while still within the reference interval for LC‐MS/MS.

Despite a similar upper reference limit, the ELISA method provided significantly higher SDMA values compared to the LC‐MS/MS method. In a subgroup of cases this led to different interpretation of renal function.

**Disclosures**

Disclosures to report, please report below

Greet Junius, Evi Van den Steen en Lisbeth Patteet are professionaly affiliated with the commercial laboratory performing the LC‐MS/MS

## 132 ESVNU‐P‐6

### 132.1 ESVNU ‐ European Society of Veterinary Nephrology and Urology

#### 132.1.1 Presentation, diagnosis, mineral findings and management of ureteral calculi in dogs: 14 cases (2010 – 2021)

##### 132.1.1.1 H Kaufmann, G Benchekroun, M Manassero, C Maurey

###### 132.1.1.1.1 Ecole Nationale Vétérinaire d'Alfort Maisons‐Alfort France

Ureterolithiasis is a rare condition in dogs and management relies on few case series. The aim of this study is to review clinical presentation, imaging findings, therapeutic management and mineral composition of ureteroliths in a canine retrospective case series.

In this retrospective study, 14 dogs presenting ureteral calculus identified on imaging examination between 2010 and 2021 were included. Signalment, clinical history, clinical examination, blood findings, urinalysis, urolith composition, imaging findings, treatment and outcome were recorded.

Results revealed that only mature small breeds were affected, with a high prevalence in Yorkshire Terrier (n=5) and Shih Tzu (n=4). Five dogs had a history of urolithiasis prior to presentation. Major chief complaints and clinical findings included lethargy (n=10), dysorexia (n=9), vomiting (n=8), abdominal pain (n=6), pyrexia (n=4) and dehydration (n=3). Azotemia (n=4), anemia (n=4), neutrophilic leukocytosis (n=3) and hypercalcemia (n=1) were identified. Urinalysis revealed that 5 dogs had positive bladder urine culture at time of diagnosis and 1 other dog had positive renal pelvic urine culture. Calcium oxalate was the most common mineral findings (suspected in 5 dogs and confirmed in 5 dogs) while other compositions remained rare (struvite n=2, urate n=1 and xanthine n=1). Risk factors identified included primary hyperparathyroidism (calcium oxalate, n=1), urinary tract infection (struvite, n=1), portosystemic shunt (urate, n=1) and allopurinol medication (xanthine, n=1).

Surgical management was performed on 8 dogs, including 5 ureterotomies (followed by stent placement in 4 dogs), 1 subcutaneous ureteral bypass placement and 2 nephrectomies. All 4 dogs that were azotemic underwent surgical procedure and improved renal function post‐operatively. Stents were successfully removed endoscopically in all cases after 51 days on average. Short term post‐operative follow‐up revealed that all dogs were recovering 1 month later. Medical management was performed on 6 dogs and was based on symptomatic medication and antibiotic therapy. Two dogs treated medically had a naturally removal of ureterolith 1 month to 1 year following diagnosis. Independently of treatment, long‐term outcome seemed to be excellent: all 7 dogs followed were alive 1 year later without azotemia.

These revised data on canine ureterolithiasis revealed predispositions in mature small breeds. Unlike cats, azotemia was rarely related to ureteroliths in dogs, but abdominal pain is a relevant clinical finding. Various mineral compositions are reported but calcium oxalate stones seem to be predominant as noticed in cats.

**Disclosures**

No disclosures to report

## 133 ESVNU‐P‐7

### 133.1 ESVNU ‐ European Society of Veterinary Nephrology and Urology

#### 133.1.1 A prospective study of kidney injury following Vipera berus envenomation in dogs sampled over a 5‐month period

##### 133.1.1.1 T. Nicolaysen ^1^, B. Sævik ^2^, R. Rørtveit ^1^, H. Sjetne Lund ^1^, K. Zimmer ^1^

###### 133.1.1.1.1 ^1^ Norwegian University of Life Sciences Ås Norway; ^2^ AniCura Jeløy Dyresykehus Moss Norway

Dogs bitten by *Vipera berus (V. berus)*, the only venomous snake in Scandinavia, may present with a range of clinical signs including painful swelling and cardiac arrythmias. In recent years the sparce evidence base for this condition has expanded, but we know little about the potential long‐term effects. In this study, our aim was to investigate whether readily available biomarkers can be used to evaluate systemic effects, with emphasis on kidney damage, following canine *V. berus* envenomation, both in the acute setting and over time.

From two animal hospitals we recruited 29 otherwise healthy dogs with a history of, and clinical findings consistent with, recent viper envenomation. Cases were sampled at up to five time points over a five‐month period. The control group consisted of 65 healthy dogs with no history of viper envenomation and were sampled once. Serum and free‐catch urine were collected at each time point. A serum biochemical profile and urine, including urine‐ protein to creatinine ratio (UPC) and albumin to creatinine ratio (uACR), were analyzed.

The relevant differences in cases compared to controls were increased creatin kinase (CK) (p = <.001), aspartate aminotransferase (AST) (p = <.001), C‐reactive protein (CRP) (p = <.001), decreased albumin and creatinine (sCr) (p = <.001, p = <.001) in serum, and increased UPC and uACR in urine (p = 0.025, p = 0.002) 24 hours after envenomation. Analytes were mostly within reference intervals two weeks later. None of the cases had changes in sCr that exceeded the reference change value during the sampling period.

Increased UPC and uACR in the acute phase suggests that *V. berus* envenomation may cause transient acute kidney injury (AKI). Although, systemic inflammation, as indicated in our case group by high CRP and low serum albumin, has also been shown to increase UPC and uACR. Increased CK and AST most likely reflect muscle injury at the envenomation site. Low sCr in cases at 24 hours was likely due to fluid therapy.

Our study strengthens evidence of a transient AKI following canine *V. berus* envenomation and indicates that UPC and uACR may be useful biomarkers for this effect. We observed that cases showed marked improvement in serum biochemical and urinary analytes at the two‐week follow‐up. No evidence was found to support development of chronic kidney disease in envenomated dogs treated according to current best practice.

**Disclosures**

Disclosures to report, please report below

The study was partly funded by a grant from "Astri og Birger Torsteds legat til fordel for dyrene"/"Astri and Birger Torsteds fund for the benefit of animals"

## 134 ESVNU‐P‐8

### 134.1 ESVNU ‐ European Society of Veterinary Nephrology and Urology

#### 134.1.1 Canine pyometra and urinary tract infection

##### 134.1.1.1 C M Öman ^1^, A Schlag ^2^, O Höglund ^1^

###### 134.1.1.1.1 ^1^ Swedish University of Agricultural Sciences Uppsala Sweden; ^2^ Western College of Veterinary Medicine, University of Saskatchewan Saskatoon Canada

ABSTRACT

Pyometra is common in intact female dogs. There are reasons to assume pyometra patients may also have urinary tract infections (UTIs) due to bacterial ascension from the genital tract to the bladder. The aim was to investigate the prevalence of UTIs amongst pyometra patients. The hypothesis was that bitches with pyometra occasionally exhibited signs of UTI.

Patient records of a university animal hospital were searched for bitches diagnosed with pyometra. This was further refined by searching for a combination of two disease processes, pyometra and urinary tract infection, diagnosed at the same consultation. This retrospective study included 8 years (2013‐01‐01 – 2020‐12‐31). Descriptive data were reported.

There were 1,095 dogs diagnosed with pyometra. In 11 cases, pyometra and UTI were diagnosed during the same consultation.

Clinicians did not commonly recognize the diagnosis UTI in canine pyometra patients. Approximately 1% of pyometra patients were given the additional diagnosis UTI. A limitation was the study's retrospective nature. Clinicians may have, for various reasons, chosen not to insert an additional diagnosis into the patient records, thereby omitting double diagnoses. Patients that were diagnosed with one diagnosis may later have been diagnosed with the other, without use of both diagnoses at the same consultation. The study was limited to investigation of prevalence of double diagnoses in the patient records, without further investigation of performed urine analysis. In pyometra patients, UTI is potentially an overlooked diagnosis.

**Disclosures**

No disclosures to report

## 135 ESCG‐P‐1

### 135.1 ESCG ‐ European Society of Comparative Gastroenterology

#### 135.1.1 Altered Tryptophan Metabolism in Cats with Chronic Inflammatory Enteropathy or Alimentary Small Cell Lymphoma

##### 135.1.1.1 P Barko ^1^, YA Wu ^2^, J Steiner ^2^, G Norsworthy ^3^, A Gal ^1^, D Williams ^1^, S Estep ^4^

###### 135.1.1.1.1 ^1^ University of Illinois Urbana United States; ^2^ Texas A&M University GI Laboratory College Station United States; ^3^ Alamo Feline Health Center San Antonio United States; ^4^ Texas Veterinary Pathology Spring Branch United States

Chronic inflammatory enteropathy (CIE) and alimentary small cell lymphoma (SCL) are common chronic enteropathies (CE) in domestic cats. Studies in humans and rodent models have implicated tryptophan (Trp) and microbial indole catabolites of tryptophan (MICT) in regulating gastrointestinal health and decreased serum and fecal concentrations of MICTs are associated with inflammation and impaired mucosal barrier function. Previous studies have identified decreased serum concentrations of Trp and decreased fecal concentrations of MICTs in cats with chronic enteropathies. However, the role Trp catabolites play in the maintenance of intestinal mucosal health in cats is unknown. The objective of this pilot study was to measure serum concentrations of Trp and MICTs in cats with clinical signs of CE and histopathologic diagnoses of either CIE or SCL and compare them to healthy cats. We hypothesized that cats with CIE and SCL would have decreased serum concentrations of Trp and MICTs compared with healthy controls.

Using surplus serum samples, Trp and three different MICTs (indole‐3‐propionic acid, indole‐3‐lactic acid, indole‐3‐acetic acid) were quantified in healthy cats (n=10) and cats with CIE (n=13) or SCL (n=7) using liquid chromatography mass‐spectroscopy against analytic standards. Serum concentrations of Trp and MICTs were log‐transformed and one‐way ANOVA was used for the overall test. Significant variables were compared between groups using pairwise t‐tests with correction for multiple testing (*P*_*adj*_).

Concentrations (mean ± SD) of Trp were significantly lower in the sera of cats with CIE (6.1 ± 1.5 μg/mL; *P*_*adj*_<0.001) and SCL (7.1 ± 2.5 μg/mL; *P*_*adj*_=0.001) compared with healthy controls (15.1 ± 2.2 μg/mL). There were no significant differences in serum concentrations of indole‐3‐propionic acid (ANOVA *P*=0.83) or indole‐3‐acetic acid (ANOVA *P*=0.19) among groups, but indole‐3‐lactic acid was significantly lower in cats with CIE (194.2 ± 189.2 ng/mL; *P*_*adj*_ =0.015) and SCL (129.8 ± 56.9 μg/mL; *P*_*adj*_=0.0019) compared with healthy cats (302.6 ± 114.6 μg/mL). There were no significant differences between the CIE and SCL groups for either Trp or indole‐3‐lactic acid.

Trp is an essential amino acid, and our findings suggest that CEI and SCL are associated with altered Trp metabolism in cats. Indole‐3‐lactic acid is known to have an anti‐inflammatory effect in the intestinal epithelium and our results establish a plausible association between altered Trp metabolism and the pathobiology of chronic enteropathies in cats. Follow‐up studies in a larger population of cats with CIE and SCL are underway to confirm our findings and elucidate their pathophysiologic significance.

**Disclosures**

Disclosures to report, please report below

This investigation was not supported by any specific grants. Three of the authors are affiliated with the Gastrointestinal Laboratory at Texas A&M University (Williams, Steiner, Wu), which provides commercial diagnostic testing and consultation services. Two of the authors receive royalties from, and are consultants with, IDEXX Laboratories (Williams, Steiner). One of the authors serves on the Nestle‐Purina Pet Care Advisory Board (Williams). Several of the authors are working on unrelated research projects funded by veterinary charitable foundations including the Morris Animal Foundation, EveryCat Health Foundation, and the American Kennel Club Canine Health Foundation (Barko, Williams, Steiner). One of the authors receives industry funding (Nestle‐Purina Pet Care) for unrelated research (Williams).

## 136 ESCG‐P‐2

### 136.1 ESCG ‐ European Society of Comparative Gastroenterology

#### 136.1.1 Gastric mucosal and plasma matrix metalloproteinase‐9 activities in dogs with gastric mucous dysplasia and carcinoma

##### 136.1.1.1 M. Hanifeh ^1^, M. Vinicius Candido ^2^, N. Koho ^2^, P. Syrjä ^2^, T. Spillmann ^2^

###### 136.1.1.1.1 ^1^ University of Helsinki, Helsinki, Finland Helsinki Finland; ^2^ University of Helsinki Helsinki Finland

Gastric carcinoma (GC) is the most common neoplasm in the canine stomach. Between 70‐90% of canine GC have metastasized by the time of diagnosis or euthanasia. Presumably, early diagnosis of GC by objective biomarkers provides improved clinical outcomes. Matrix metalloproteinase (MMP) ‐9 plays an important role in tumor cell proliferation and invasion, by degrading and remodeling extracellular matrix surrounding the tumor. MMP‐9 is upregulated in mucosal and plasma samples of human patients with GC, proposing its utilization for tumor screening, and as a prognostic biomarker. Comparable information is missing in dogs with GC. Therefore, we investigated the activity of mucosal and plasma MMP‐9 in Belgian Shepherd dogs, a breed predisposed to GC. We hypothesized MMP‐9 would behigher in dogs with GC than with suspected preneoplastic changes (mucous metaplasia and glandular dysplasia), or dogs with no gastric neoplastic/preneoplastic changes.

In this study, 19 Belgian Shepherd dogs were enrolled, comprising Tervueren (n=11), Groenendael (n=7), and Malinois (n=1). Based on histologic classification of endoscopically taken gastric mucosa biopsies, six dogs had no gastric neoplastic changes (control group), seven dogs had preneoplastic changes such as mucous metaplasia (n=3) and/or glandular dysplasia (n=4), and six dogs had gastric carcinoma. Pro‐ and active‐MMP‐9 activity was determined by zymography in gastric mucosal biopsies of 16/19 dogs and in EDTA plasma of all 19 dogs. Data are presented as medians (ranges).

The results showed that mucosal pro‐MMP‐9 activities were significantly higher in dogs with gastric carcinoma (n=5) than in the control group (n=5) (0.982 [0.509–1.247] arbitrary units (AU) vs. 0.050 [0–0.581] AU, p=0.016) and in dogs with glandular dysplasia (n=4) (0.982 [0.509–1.247] AU vs. 0.153 [0.125–0.178] AU, p=0.016). In addition, mucosal active‐MMP‐9 had a moderate to strong positive correlation with lamina propria eosinophils (r=0.737, p=0.001) and total gastric mucosal histopathologic scores (r=0.54, p=0.031). Plasma active‐MMP‐9 activities were significantly higher in dogs with neoplastic/preneoplastic changes (n=13) than in the control group (n=6) (0.0679 [0–0.215] AU vs. 0.0179 [0–0.089] AU, p=0.047). Plasma active‐MMP‐9 had a moderate positive correlation with lamina propria neutrophils (r=0.532, p=0.019).

This study showed that mucosal pro‐MMP‐9 and plasma active‐MMP‐9 activities are increased in dogs with GC compared to dogs with no gastric neoplastic/preneoplastic changes. The results provide supporting evidence for the potential diagnostic value of plasma MMP‐9 activity as a biomarker in dogs with GC and support the need for further studies in a larger patient population.

**Disclosures**

No disclosures to report

## 137 ESCG‐P‐3

### 137.1 ESCG ‐ European Society of Comparative Gastroenterology

#### 137.1.1 Description and treatment of non‐neoplastic duodenal bulb ulcer in twelve dogs: an observational study

##### 137.1.1.1 M. Sabetti ^1^, L. Pisoni ^2^, A. Foglia ^2^, V. Cola ^2^, D. Stanzani ^2^, G. Galiazzo ^3^, C. Tagliavia ^2^, M. Pietra ^2^

###### 137.1.1.1.1 ^1^ University of Parma Parma Italy; ^2^ University of Bologna Bologna Italy; ^3^ Veterinary Practitioner Rovigo Italy

The duodenal bulb ulcer is an injury developing when deleterious effects of gastric acid overwhelm the defensive properties of the duodenal mucosa; it is often characterized by continuous bleeding and treatment is challenging whether medical, endoscopic, or surgical. The purposes of this observational study are to: 1) describe the clinical and endoscopic presentation; 2) describe the treatment of duodenal bulb ulcer in dogs; 3) investigate the role of vascularization in the persistence of ulcer.

The study included all dogs, presented at our institution between 2016 and 2022, with signs referable to persisting gastrointestinal bleeding, and an endoscopic diagnosis of duodenal bulb ulcer.

Polyurethane foam casts of gastro‐duodenal vessels, used to investigate the role of vascularization in the persistence of ulcer, were obtained, after owner authorization, from 5 dogs died for reasons unrelated to the alimentary or cardiovascular system.

This study involved 12 dogs of various breeds, 5 males (3 neutered) and 7 females (5 spayed), aged 6.5 ± 4.3 years, with duodenal non‐neoplastic ulcer. The most recurring clinical signs were dysorexia (10/12), vomiting (8/12), and melaena (8/12). Common clinicopathological findings were severe normochromic normocytic regenerative anemia (8/12), hypoproteinemia, (8/12) and hypoalbuminemia (9/12). Predisposing factors or comorbidities detected here were: exocrine pancreatic insufficiency, cardiomyopathy, chronic enteritis, chronic kidney disease, immunomediated thrombocytopenia, pulmonary carcinoma. Due to the severe bleeding, a blood transfusion was needed in 7/12 dogs before the endoscopy. On endoscopic examination, the ulcer was flat in 4/12, slightly excavated in 6/12, and deep in 2/12 dogs; in all cases, the ulcer affected the mesenterial side of the duodenal bulb. Active bleeding was observed in 10/12 patients, and in 9 of them it was associated with wall thickening. Medical treatment, including proton‐pump inhibitors, was started after endoscopy. Dogs not responding to one week of medical therapy were subsequently treated with endoscopic electrocauterization (4/6), or surgical resection Billroth type 1 (2/6). Surgical and endoscopic treatments successfully resolved the ulcer bleeding, without any recurrences, regardless of the predisposing factors.

All the polyurethane foam casts of gastro‐duodenal vessels revealed a venous plexus at the mesenteric side of the duodenal bulb.

In all examined cases, the ulcer was located at the mesenteric part of the duodenum, on a side less exposed to the trauma caused by the passage of ingesta.

We hypothesized that the presence of venous plexus of the duodenal bulb may inhibit ulcer healing, resulting in continuous bleeding.

**Disclosures**

No disclosures to report

## 138 ESCG‐P‐4

### 138.1 ESCG ‐ European Society of Comparative Gastroenterology

#### 138.1.1 Pre‐illness dietary risk factors in dogs with chronic enteropathy

##### 138.1.1.1 I. Trewin, A. Kathrani

###### 138.1.1.1.1 The Royal Veterinary College Hatfield United Kingdom

Inflammatory bowel disease (IBD) in people has a complex pathophysiology, involving the interplay of environmental factors, gastrointestinal microbiome, genetics, and the immune response. Diet has been extensively studied in people as a potential trigger of IBD. However, scant literature exists regarding the role of diet as a pre‐illness risk factor in canine chronic enteropathy (CE). Therefore, the aim of our study was to evaluate possible pre‐illness dietary risk factors in dogs with CE.

This was a prospective case‐control, questionnaire‐based study performed at a referral teaching hospital in the U.K. between August 2020 and January 2022 and included 100 dogs; 50 in the CE group and 50 in the control group. The CE group comprised of 25 dogs with a definitive diagnosis based on histopathology of gastrointestinal biopsy specimens, and 25 dogs with suspected CE based on thorough diagnostic investigations, ruling out all known causes, but without gastrointestinal histopathology. The control group included those dogs that presented to our Internal Medicine Service with no current or historical gastrointestinal signs during the same period. A diet history was obtained relating to the point of onset of presenting clinical signs for both groups. The main diet provided underwent ingredient analysis and caloric distribution calculation using a guaranteed analysis convertor software.

Dogs in the CE group were younger with a median age of 42 months (range 3‐121) compared to a median age of 92 months (range 7‐187) in the control group (P<0.001). The main diets were fed for a shorter duration of time prior to the onset of clinical signs in the CE group (median 12 months; range 0.5‐84 months) compared to the control group (median 36 months; range 1‐96 months) (P<0.001). The frequency of the main diet containing no carbohydrate was greater in the control group compared to the CE group (7/47 dogs in control group, 0/48 dogs in CE group; P=0.008). The presence of a red meat source in the main diet was lower in the CE group (16.7%, 8/48 dogs) compared to the control group (34.0%, 16/47 dogs), however this did not meet statistical significance (P=0.051).

A shorter duration of the diet fed at the onset of signs may play a role in the pathogenesis of canine CE, however studies using age‐matched control dogs would be needed to further investigate this. The presence and source of carbohydrate and protein, respectively as potential triggers for canine CE also requires further investigation.

**Disclosures**

No disclosures to report

## 139 ESCG‐P‐5

### 139.1 ESCG ‐ European Society of Comparative Gastroenterology

#### 139.1.1 Fecal microbiota analysis in cats with inflammatory bowel disease or small cell lymphoma before and after therapy: a prospective comparison study

##### 139.1.1.1 E. Benvenuti ^1^, R. Ferriani ^2^, P. Gianella ^3^, P. Ruggiero ^1^, F. Cagnasso ^3^, A. Borrelli ^3^, E. Sattin ^4^, E. Bottero ^1^

###### 139.1.1.1.1 ^1^ EndoVet group Rome Italy; ^2^ Ospedale Veterinario San Francesco Milan Italy; ^3^ University of Turin Grugliasco (To) Italy; ^4^ BMR Genomics Padua Italy

There are only limited data on the microbiota of cats with inflammatory bowel disease (IBD) and small cell lymphoma (SCL) before and after therapy. The aim of this multicentric prospective comparison study was to characterize the faecal bacterial microbiota before (T0) and after 1 month (T1) of therapy in cats with histologically confirmed IBD or SCL. Based on a three‐month follow‐up, cats were assigned to group A (improvement) or group B (no improvement/worsening). Faecal samples were collected at T0 and T1. Microbiota DNA was extracted using Cador Pathogen 96 QIAcube HT Kit protocol and sequenced using the V3 kit‐300PE strategy. Alpha‐ and beta‐diversity were calculated and heatmaps were created. Twenty cats with IBD (13) or SCL (7) and 5 healthy cats were enrolled. Fifteen cats were assigned to group A (11 IBD, 4 SCL) and 5 to group B (2 IBD, 3SCL). All diseased cats received glucocorticoids (G). In addition, 3 cats (1 IBD, 2 SCL) received antibiotics (GA). No differences in alpha‐ and beta‐diversity were found neither at T0 nor at T1 between healthy and diseased cats, IBD and SCL cats, G and GA therapy. Heatmap analysis showed an increase in the family *Fusobacteriacee* in cats with SCL at T0. The small number of cases and the mere execution of metagenomics do not allow to draw meaningful conclusions. Despite this, on the basis of the data collected, the metagenomic evaluation does not seem to be able to discriminate IBD from SCL, nor to help in the prognostic prediction.

**Disclosures**

No disclosures to report

## 140 ESCG‐P‐6

### 140.1 ESCG ‐ European Society of Comparative Gastroenterology

#### 140.1.1 The role of hydrolysed diet in the management of chronic diarrhoea in dogs – is it always the first therapeutic step?‐ A retrospective multicentric study

##### 140.1.1.1 S. Duvergé ^1^, M.J. Dias ^2^, N. Santos ^3^, M. Hebert ^4^, E. Bettin ^5^, F. Signorelli ^5^, J. Hernandez ^4^, F. Procoli ^5^, R. Leal ^2^

###### 140.1.1.1.1 ^1^ Veterinary Teaching Hospital ‐ Faculty of Veterinary Medicine ; ULisboa Lisbon Portugal; ^2^ Centre for Interdisciplinary Research in Animal Health, Fac.Vet.Med., ULisboa Lisbon Portugal; ^3^ Veterinary Teaching Hospital, Fac. Vet. Med., ULisboa Lisbon Portugal; ^4^ Oniris, Ecole Nationale Vétérinaire Agroalimentaire et de l'Alimentation Nantes France; ^5^ Anicura Ospedale Veterinario Portoni Rossi, Bologna Italy

Diet has a major role in the therapeutic approach to canine chronic diarrhoea. In these cases, hydrolysed diets are generally used as a first treatment step, with a good response and a better long‐term outcome.

This study aims to evaluate the percentage of canine chronic diarrhoea cases presented for referral or second opinion consultation, to which a hydrolysed dietary trial was not previously performed, assessing the relevance of dietary change in those cases.

A retrospective multicentric study involving three referral centres was conducted. Medical records of dogs referred or consulted for second opinion presenting chronic diarrhoea (>3weeks duration) between April 2017 and December 2021 were reviewed. Cases were included if there was a complete medical history and follow‐up for at least one month. Data concerning supportive therapy (probiotics, antiemetics, gastric protectants or cobalamin supplementation), antibiotics and immunosuppressants prescribed prior to the consultation was assessed. Cases were excluded if an extra‐digestive cause of chronic diarrhoea was suspected or confirmed. Dogs that had no previous history of a hydrolysed diet trial were further characterized. For data presentation, descriptive statistic was used.

From 142 dogs that met the inclusion criteria, 81 (57%) had never received hydrolyzed diet prior to these consultations. A total of 38/81(47%) dogs were not under medication when consulted; 24/81(30%) were on supportive therapy; 10/81(12%) were receiving antibiotics and 9/81(11%) were under immunosuppressants.

At first consultation, the transition onto a hydrolysed diet was the only treatment decision in 16/81 (20%) dogs. In the remaining 65/81(80%), a dietary trial was prescribed in association with either supportive therapy (40/65; 62%), antibiotics (12/65; 18%) and/or immunosuppressants (13/65; 20%).

Overall, a clinical improvement was documented in 57/81(70%) cases: 8 over 16 dogs exclusively treated with dietary trial and 49 over 65 with diet and ancillary treatment. In 24/81(30%) no response was observed: 8 cases over 16 to which diet trial was the only treatment and 16 over 65 from those treated with diet and ancillary treatments.

More than reinforcing the role of hydrolysed diet on the therapeutic approach of canine chronic diarrhoea, this study highlights that over half of dogs that consult at a referral center never experienced a hydrolysed dietary trial. A minor percentage had received immunosuppressants and antibiotics prior to a hydrolyzed diet, stressing the need of a better standardized therapeutic approach of a case of canine chronic diarrhoea among first‐opinion practitioners.

**Disclosures**

Disclosures to report, please report below

No conflict of interest to declare. This work was supported by FCT – Fundação para a Ciência e Tecnologia IP, grant UIDB/00276/2020

## 141 ESCG‐P‐7

### 141.1 ESCG ‐ European Society of Comparative Gastroenterology

#### 141.1.1 Study of oxidative stress biomarkers in dogs with chronic inflammatory enteropathy

##### 141.1.1.1 J. Cristóbal Verdejo, S Martínez Morcillo, F. J. Duque Carrasco, M. P Míguez Santiyan, J.M Usón Casaús, E.M Pérez Merino

###### 141.1.1.1.1 Veterinary Clínical Hospial, University of Extremadura Cáceres Spain

Oxidative stress plays an important role in the pathogenesis of canine chronic inflammatory enteropathy (CIE). Malondialdehyde (MDA), as a byproduct of lipid peroxidation and reduced glutathione (GSH) are widely used biomarkers as important oxidative markers, but they never have been described in CIE dogs. Moreover, albumin represents a very abundant and important circulating antioxidant. Therefore, the aim is to evaluate the oxidative stress in CIE dogs through those parameters.

For this purpose, 30 dogs divided into a control group with 10 healthy dogs and a CIE‐group consisting of 20 diagnosed with CIE. MDA, GSH, and albumin values from surplus plasma samples left over from CIE and healthy animals were obtained. Dogs in the control group attended a routine veterinary check‐up. A Mann‐Whitney U test were used to compare values of healthy and CIE dogs. Results were expressed as median (minimum ‐ maximum). Statistical significance was set at P < 0.05.

MDA concentrations were increased in CIE dogs compared to the control group (7.98 [3.90 ‐ 13.16]) *vs* 6.46 [2.04 ‐ 9.17] and GSH concentrations were lower in CIE dogs compared to healthy animals (18.00 [5.34 ‐ 38.14] *vs* 26.39 [8.06 ‐ 48.22]). Despite the trend to increase in dogs with CIE for MDA and the trend to decrease for GSH, no significant differences were observed. Albumin concentration shows a significant decrease in diseased animals compared to healthy animals (2.60 [1.50 ‐ 4.64]) *vs*. 3.00 [2.50 ‐ 5]; *P* = 0.037).

As differences in albumin in CIE dogs with respect to healthy dogs, can be considered as marker of CIE and of the oxidative status of that disease. Despite the changes observed in MDA and GSH, they cannot be considered biomarkers of oxidative stress. Future studies expanding the number of patients would be necessary.

**Disclosures**

No disclosures to report

## 142 ESCG‐P‐8

### 142.1 ESCG ‐ European Society of Comparative Gastroenterology

#### 142.1.1 Feline chronic inflammatory enteropathy: evaluation of treatment outcome and survival in 80 cases

##### 142.1.1.1 Y. Bandara, S Priestnall, Y.M. Chang, A Kathrani

###### 142.1.1.1.1 Royal Veterinary College Hatfield United Kingdom

Feline chronic inflammatory enteropathy (FCIE) is a debilitating idiopathic disease that may result in euthanasia due to treatment failure. Currently, there is limited data on factors that might affect treatment outcome and survival in affected cats. Therefore, the aim of this study was to determine if clinical and laboratory variables at the time of histological diagnosis are associated with treatment outcome and survival in cats with FCIE.

The medical records at a single referral teaching hospital in the UK between September 2011 and August 2021 were retrospectively searched for cases of FCIE on the basis of histopathology of intestinal biopsy specimens. A board‐certified veterinary pathologist then re‐reviewed all intestinal biopsy specimens for inclusion. For each eligible case, medical records were thoroughly reviewed and various variables at the time of diagnostic investigation were recorded. Follow‐up information was obtained either by telephone or written contact with the referring veterinary surgeon. Clinical remission was defined as the absence of previous presenting clinical signs for gastrointestinal disease, consisting of vomiting, diarrhoea, hyporexia and weight loss.

At discharge from the referral teaching hospital, 47 (59%) cats were prescribed dietary therapy alone; 8 (10%) received an antibiotic with or without dietary therapy and 25 (31%) received an immunosuppressant with or without dietary therapy. Treatment and survival outcome was obtained in 67 (84%) cats. Of these 67 cats, 24 (36%) were euthanised due to their gastrointestinal disease within a median of 104 days following histological diagnosis (range 2‐2970). Thirty‐one (46%) cats were in clinical remission (median follow‐up of 916 days). Of these cats in clinical remission, 23 (74%) were receiving the same treatment prescribed at discharge, whereas 4 (13%) received escalated therapy and 4 (13%) received reduction of therapy at follow‐up. Attaining remission decreased the likelihood of death due to gastrointestinal disease (OR=0.23, 95% confidence interval: 0.003‐0.190, *P*=0.001). Escalation of therapy following discharge did not impact death due to gastrointestinal disease (*P*=0.552). There were no significant effects of body condition score, body weight, serum albumin concentration, serum alanine transaminase activity and serum cobalamin and folate concentrations on survival (*P*>0.05).

Measured physical and laboratory parameters at the time of histological diagnosis of FCIE were not predictors of survival. However, our study suggests that attaining clinical remission can be used as a predictor of survival in cats with FCIE. Alternative diagnostic measures may be required to definitively investigate treatment outcome and survival in cats with FCIE.

**Disclosures**

No disclosures to report

## 143 ESVIM‐P‐1

### 143.1 ESVIM ‐ European Society of Veterinary Internal Medicine

#### 143.1.1 Utility of historical findings, cytologic assessment, and microbial culture in determining the etiology of pyothorax in 29 cats and 60 dogs (2010‐2020)

##### 143.1.1.1 L. Johnson, S. Epstein, K. Reagan

###### 143.1.1.1.1 University of California‐Davis Davis United States

Pyothorax in dogs is often ascribed to foreign body inhalation, particularly in some geographic regions, while the etiology in cats can be more difficult to discern.

The aim of this study was to examine clinical, microbiologic findings, and etiology in cats and dogs with pyothorax.

Medical records of cats and dogs diagnosed with pyothorax from 2010‐2020 were reviewed. Clinical history, physical examination findings, fluid analysis, and microbiologic results were retrieved.

Results were available for 29 cats and 60 dogs. Antimicrobials had been administered to equal proportions of cats and dogs prior to admission (45 and 47%). Median age and total protein and percent neutrophils in effusion did not differ between species, although median effusion cell count was significantly higher in cats (195,720/μl) than in dogs (115,160/μl), P = .005. Neutrophils containing intracellular bacteria were identified in more cats (27/29;93%) than dogs (44/60;73%), P=.046. Penetrating injuries (bite wounds or foreign body inhalation) were implicated as the cause of pyothorax in equal percentages of cats (79%) and dogs (73%). Pneumonic or hematogenous spread also affected similar proportions of cats (4/29;14%) and dogs (9/60;15%). Cats had greater numbers of bacteria isolated (median 3) than dogs (median 1), P = .006, and anaerobes were isolated more often in cats (23/29;73%) than in dogs (27/60;45%, P = 0.003).

In this study, pyothorax was caused by similar etiologies in cats and dogs. Cats had higher fluid cell counts, greater numbers of bacteria isolated, and intracellular bacteria detected more commonly than dogs.

**Disclosures**

No disclosures to report

## 144 ESVIM‐P‐2

### 144.1 ESVIM ‐ European Society of Veterinary Internal Medicine

#### 144.1.1 Investigation of serum circulating cell free nucleosomes as biomarker of canine idiopathic pulmonary fibrosis in West Highland white terriers

##### 144.1.1.1 F. Fastrès ^1^, H. Herzog ^2^, P. Pamart ^2^, R. Roels ^3^, C. Clercx ^3^

###### 144.1.1.1.1 ^1^ University of Liège Ougrée Belgium; ^2^ Belgian Volition SRL Namur Belgium; ^3^ University of Liège Liège Belgium

Canine idiopathic pulmonary fibrosis (CIPF) occurs in aged West Highland white terriers (WHWTs). The disease is characterized by profound fibrotic lung distortion leading to progressive respiratory impairment. Cell‐free nucleosomes (cf‐nucleosomes), which contain DNA and histones, contribute to gene expression regulation and are released into the circulation from any damaged cells. Accordingly, cf‐nucleosomes have been shown increased in dogs with sepsis, cancer, immune‐mediated haemolytic anaemia and trauma with both diagnostic and prognostic values. In human idiopathic pulmonary fibrosis (IPF), a disease similar to CIPF, a combination of circulating cf‐nucleosomes with specific epigenetic features has been identified with a good diagnostic accuracy to discriminate IPF patients from healthy subjects.

In this study, we aimed to determine whether the amount of cf‐nucleosomes in blood can be used as a non‐invasive diagnostic biomarker for CIPF in WHWTs. Serum cf‐nucleosomes were measured in 12 WHWTs affected with CIPF and compared with 10 healthy age‐matched WHWTs, 9 healthy age‐matched dogs from other breeds and 9 dogs affected with community acquired bacterial pneumonia (CAP). ELISA kits (Nu.Q®) validated for dogs were used to measure cf‐nucleosomes concentrations. Kruskal Wallis test with pairwise post‐hoc Conover‐Iman tests and Bonferroni correction for multiple comparisons were used to compare cf‐nucleosomes concentrations between groups.

Cf‐nucleosomes concentration was significantly increased in dogs affected with CAP (22.06ng/mL (12.79‐30.56)) in comparison with WHWTs affected with CIPF (1.53ng/mL (0.94‐3.33); P<0.0001) and healthy WHWTs (3.12ng/mL (2.42‐6.16); P=0.002). Cf‐nucleosomes concentration was also higher in dogs affected with CAP in comparison with healthy dogs from other breeds although it was not significant because of 2 outliers (4.88ng/mL (2.98‐10.41); P=0.019). There was no difference between WHWTs affected with CIPF and healthy dogs of the same breed (P=0.087) or different breeds (P=0.012).

Results of this study did not support the use of circulating cf‐nucleosomes for the non‐invasive diagnosis of CIPF in WHWTs. However, increased circulating cf‐nucleosomes concentration was found in dogs affected with CAP, probably because of a higher rate of cell injury induced by the acute inflammation that occurs in such disease. Whether measurement of circulating cf‐nucleosome can help discriminate CAP from other acute respiratory diseases and serve as monitoring tool for medical therapy requires further studies.

**Disclosures**

Disclosures to report, please report below

M. Herzog and D. Pamart are working for the firm that has produced the ELISA kit that we used.

## 145 ESVIM‐P‐3

### 145.1 ESVIM ‐ European Society of Veterinary Internal Medicine

#### 145.1.1 A retrospective evaluation of efficacy and tolerability of buprenorphine as an antitussive agent

##### 145.1.1.1 W.‐.T. Chang ^1^, P.‐.Y. Lo ^2^, C.‐.H. Lin ^1^

###### 145.1.1.1.1 ^1^ 1. National Taiwan University, 2. TACS‐Alliance Research Center Taipei Taiwan; ^2^ TACS‐Alliance Research Center Taipei Taiwan

Buprenorphine is an opioid approved for analgesia or sedation in dogs. It has high affinity to mu‐opioid receptor, which affects the modulation of narcotic antitussive effect in the cough center. This study aimed to retrospectively evaluate the antitussive effect of buprenorphine in dogs.

Fifty‐one dogs were prescribed buprenorphine (Temgesic Injection, Reckitt Benckiser) for the exacerbation of cough via the oral transmucosal route (OTM) due to the unavailability of butorphanol or the clinician's concern. Medical records were reviewed for cough characteristics, diagnosis, and therapeutic response. The overall severity of cough before and after the administration of buprenorphine was scored from 0 (no cough) to 5 (most severe) by a single clinician. Cases were excluded as insufficient information was available for assessing the efficacy.

Thirty‐one of the 51 dogs were enrolled in the final analysis, and the median dose administered was 14.1 μg/kg (range 6.3–20). Cough reduction was observed in 90.3% of the dogs, and the median score of cough severity decreased from 4 (2–5) to 2 (1–4) (p<0.001). The duration of the antitussive effect was recorded in 10 dogs, ranging from 2.5 to 24 hours. The antitussive efficacy of the drug was not associated with the chronicity or diseases (tracheobronchomalacia or left atrium enlargement). Adverse effects were minor and disappeared following dose reduction, including sedation (9.7%), gastrointestinal disturbances (9.7%), hypersalivation (6.5%), and anxiety (6.5%).

In conclusion, these data suggest that buprenorphine OTM can effectively suppress cough and appears to be an option for antitussive use in dogs.

**Disclosures**

Disclosures to report, please report below

A part of this study was supported by National Taiwan University and the grant from Ministry of Science and Technology, Taiwan.

## 146 ESVIM‐P‐4

### 146.1 ESVIM ‐ European Society of Veterinary Internal Medicine

#### 146.1.1 Calculation of a reference interval for normal rectal temperature in dogs using an algorithm for mixed data.

##### 146.1.1.1 E. Dorn ^1^, K. Bogedale ^2^, A. Pankraz ^3^, R. Neiger ^4^

###### 146.1.1.1.1 ^1^ Ghent University Merelbeke Belgium; ^2^ Clinic of Small Animal Medicine, LMU University of Munich Munich Germany; ^3^ Bioscientia Healthcare GmbH Ingelheim Germany; ^4^ Evidensia DACH Giessen Germany

Veterinarians rely on the measurement of body temperature in dogs every day. Aberrance from the normal temperature may indicate the presence of disease. The physiological range of body temperature in dogs has been the objective of few studies based only on a small number of dogs. There are no studies defining the reference range of the rectal body temperature from a mixed data set based on a large group of dogs so far.

The aim of this retrospective study was to define the rectal body temperature of adult dogs based on a large data set of diseased and non‐diseased animals and to evaluate the capability of the employed algorithm to calculate reference intervals of numerical clinical data. The reference interval was calculated using an algorithm developed by the Deutsche Gesellschaft für Klinische Chemie und Laboratoriumsmedizin e.V. in its Reference Limit Estimator software.

Out of the initial 25218 measurements, data of 9782 dogs > 1‐year of age that underwent clinical examination at a University Clinic for Small Animals, between January 2008 and June 2017 underwent further statistical analysis. Data exclusion was due to age (2262 dogs), duplication of measurement (11962 dogs) or being outside expected temperature values of <30°C or > 43°C (seven dogs).

Overall, 664 measured rectal temperatures were identified as outliers, as identified by the 1.5 IQR rule, and 9117 were used for further statistical analysis. The median rectal temperature was 38.6°C. The lower and upper reference ranges were 37.7°C (95% CI: 37.7‐37.7°C) and 39.5°C (95% CI: 39.5‐39.5°C).

The determination of a reference interval for rectal temperatures in dogs using an algorithm for mixed data set yielded results comparable to the existing reference intervals. This demonstrates that the calculation of reference intervals from mixed data sets is feasible for clinical numerical data and can be used to confirm existing reference intervals or establish such de novo.

**Disclosures**

No disclosures to report

## 147 ESVIM‐P‐5

### 147.1 ESVIM ‐ European Society of Veterinary Internal Medicine

#### 147.1.1 Comparison of mycophenolate mofetil and cyclosporine A in dogs with primary immune‐mediated hemolytic anemia in terms of therapeutic response, side effects and outcome

##### 147.1.1.1 A.Z. Rosu ^1^, V. Steiner ^2^, I.A. Burgener ^2^, I. Schwendenwein ^2^, N. Luckschander‐Zeller ^2^

###### 147.1.1.1.1 ^1^ University of Veterinary Medicine Vienna Wien Austria; ^2^ University of Veterinary Medicine Vienna Vienna Austria

Immunosuppression is the primary treatment principle for immune‐mediated hemolytic anemia (IMHA) in dogs. To keep prednisolone‐associated side effects low, it is combined with second‐line immunosuppressants such as cyclosporine (CsA) or mycophenolate mofetil (MFT). The aims of the study were to compare MFT vs. CsA with respect to treatment response, side effects and outcome.

In a retrospective study medical case records of dogs with primary IMHA treated between 2018 and 2022 were evaluated. Diagnoses were based on the ACVIM consensus statement. Twenty dogs met inclusion criteria and were divided according to the add‐on immunosuppressive therapy (N=10/group). Treatment response was measured by increase of hematocrit (hct) and duration of prednisolone treatment. Side effects were assessed according to the following clinical criteria: diarrhea, vomiting, nausea, hypertension, increased body temperature, coagulation‐status and ALT‐increase. Outcome was evaluated by clinical improvement and survival. For statistical analysis the graph pad prism 7.0 program was used, p< 0.05 was considered significant.

No significant differences were found in regard to initial hct, hct increase and number of blood transfusions. The timepoint of prednisolone discontinuation differed significantly between the two groups (p=0.01) with a shorter administration of prednisolone in the CsA group. No statistically significant differences could be detected regarding side effects, clinical improvement and outcome.

In summary, the two groups were equal considering side effects and treatment outcome. However, for dogs suffering severely from the side effects of prednisolone, add‐on with CsA seems to be beneficial by shortening the requirement for prednisolone administration.

**Disclosures**

No disclosures to report

## 148 ESVIM‐P‐6

### 148.1 ESVIM ‐ European Society of Veterinary Internal Medicine

#### 148.1.1 Pharmacokinetics of a single orally administered immunosuppressive dosage of cyclosporine A in healthy cats

##### 148.1.1.1 R. Mischke ^1^ L. Schuh ^1^, D. Grote‐Koskas ^2^, M. Kietzmann ^1^

###### 148.1.1.1.1 ^1^ University of Veterinary Medicine Hannover Hannover Germany; ^2^ Hannover Medical School Hannover Germany

The aim of this study was to determine a pharmacokinetic profile for a single oral dosage of cyclosporine A (CsA) clinically used for immunosuppression in cats. Blood‐CsA‐concentrations were measured before and 1, 2, 4, 6, 8, 12 and 24 hours after oral administration of 7 mg/kg body weight (BW) CsA (Atopica® oral solution) to 8 healthy adult cats using high‐performance liquid chromatography. Pharmacokinetic parameters were calculated using WinNonLin software mainly based on a 1‐compartment‐model and additional parameters using a non‐compartment model. The median maximum plasma‐concentration of 1466 ng/ml (530–2235 ng/ml; minimum–maximum) was reached after 2.0 h (1.0–4.7 h). The area under the curve was 12568 h x ng/ml (5732–20820 h x ng/ml) and the apparent total clearance of the drug from plasma was 557 ml/h/kg (336–1221 ml/h/kg). Half‐life of absorption into the central compartment was 0.6 h (0.4–2.6 h), half‐life of elimination from the central compartment was 4.6 h (1.4–7.5 h). Additional calculations using a non‐compartment‐model included apparent volume of distribution during the terminal phase (4986 ml/kg, 3362–10542 ml/kg) and the predicted apparent total clearance of the drug from plasma (458 ml/h/kg, 305–1107 ml/h/kg). Mean residence time was 8 h (5.8–9.3 h) and apparent terminal elimination half‐life time was 7.5 h (5.6–10.3 h). Concentrations after 12 h (C_12_) were 445 ng/ml (205–671 ng/ml). In conclusion, C_12_ results comply with recommended blood trough levels, indicating that 7 mg/kg BW CsA twice daily are adequate for initiation of immunosuppressive therapy in cats.

**Disclosures**

No disclosures to report

## 149 ESVIM‐P‐7

### 149.1 ESVIM ‐ European Society of Veterinary Internal Medicine

#### 149.1.1 Laryngeal silicone stent as a treatment option for laryngeal paralysis in dogs: A case series of 8 dogs (2020‐2022)

##### 149.1.1.1 M.‐.L. THERON, T. LAHUERTA‐SMITH

###### 149.1.1.1.1 VETIVIA Biarritz France

Laryngeal paralysis (LP) results in upper airway obstruction and can be life‐threatening. Currently, surgical correction is the treatment of choice and unilateral arytenoid cartilage lateralization is the preferred technique. Nevertheless, surgery cannot always be performed in the emergency setting as it needs of an experienced surgeon. Furthermore, it is an invasive procedure that is sometimes refused by owners due to the old age of the pet or due to significant comorbidities. In human medicine, the use of silicone laryngeal stents has been described for treatment of laryngeal disease, especially for laryngo‐tracheal stenosis.

The aim of this study was to describe the use of laryngeal silicone stents as a treatment for LP in dogs. Dogs diagnosed with LP via endoscopy where surgery was refused by its owners were included in the study. Signalment, endoscopic and radiographic findings, as well as immediate clinical response, at 1 week and at 1 month after stent placement were recorded. Another follow‐up phone call was performed at the time of writing.

Eight dogs with bilateral LP were included in the study (6 acquired idiopathic LP, 1 LP du to cervical injury and 1 congenital LP). The median age was eleven years, ranging from 8 months to 12 years. All dogs experienced an improvement of their clinical signs immediately after the procedure. One week after the procedure, 6 dogs had any signs of recurrence of clinical signs, aspiration pneumonia or discomfort. Two dogs aspirated water while drinking, which disappeared after repositioning the stent (which was thought to interfere with correct functioning of the epiglottis). Three weeks later, one of these 2 dogs developed megaoesophagus. Quality of life steadily worsened and euthanasia was performed. Finally, another dog had a relapse of stridor caused by caudal migration of the stent into the cranial trachea due to an inappropriate stent diameter. This dog underwent arytenoid lateralization surgery due to an unavailability of larger stents. At the follow‐up phone call one‐month post‐stenting, none of the 6 remaining dogs had any signs of recurrence of clinical signs, aspiration pneumonia or discomfort. At the time of writing, a few months after stent placement, no significant incidents had occurred.

Laryngeal silicone stenting appears to be an interesting alternative for treating dogs with LP when classic arytenoid lateralisation surgery is refused by owners. Furthermore, stent placement can be a temporary solution to stabilize these dogs until a permanent surgical treatment can be performed.

**Disclosures**

No disclosures to report

## 150 ESVIM‐P‐8

### 150.1 ESVIM ‐ European Society of Veterinary Internal Medicine

#### 150.1.1 Cyclosporin A blood levels and dose adjustment in dogs with primary immune‐mediated haemolytic anaemia

##### 150.1.1.1 R. Mischke, L. Schuh

###### 150.1.1.1.1 University of Veterinary Medicine Hannover Hannover Germany

Detailed studies on measurements of blood levels of cyclosporin A (CsA) and of the efficacy of CsA dose adjustments are not available for dogs suffering from primary immune‐mediated haemolytic anemia (pIMHA). This retrospective study aimed to assess, whether a target range of 200–600 ng/ml for blood trough levels (C_0_) was achieved using a protocol with twice daily oral CsA‐administrations. In addition, the efficacy of CsA dose adjustments and the theurapeutic effect (in combination with oral prednisolon) were examined, as well as possible adverse reactions.

31 Patients were included. Blood samples for measurements of C_0_‐values by high performance liquid chromatography (HPLC) (coupled with tandem mass spectrometry) were optained after a treatment period of at least 8 days. Blood collection was performed 12 hours after the previous and directly before the next CsA administration. Of 24 dogs that received twice daily CsA dosages of 7 mg/kg of body weight (standard protocol at the Small Animal Clinic), 7 dogs had C_0_‐values within the target range, 7 dogs had values above and 10 dogs below the target range. Dose adjustments only partly led to values within the target range. CsA blood levels showed marked inter‐ and intraindividual variability and no correlation with CsA dosages (r_s_ = ‐0.008; *P* = 0.947). Dogs which did not receive blood transfusions during the CsA therapy achieved an increase of the haematocrit of 5 or 10 % after on average 4.5 or 8 days, respectively. Side effects were mostly mild and transient and mainly included gastrointestinal, but also dermatological symptoms.

Results of this study illustrate that in principle twice daily dosages of 7 mg CsA/kg of body weight are suitable to achieve defined target blood levels. Monitoring of blood levels seems to be necessary due to high interindividual variability, even though dose adjustments had not always the desired effect possibly reflecting also high intraindividual variability. In addition, clinical efficacy should be considered.

**Disclosures**

No disclosures to report

## 151 ESVIM‐P‐9

### 151.1 ESVIM ‐ European Society of Veterinary Internal Medicine

#### 151.1.1 Obtaining reference values of rectal temperature in adult cats using an algorithm for mixed data

##### 151.1.1.1 K. Bogedale ^1^, E Dorn ^2^, A Pankraz ^3^, R Neiger ^4^

###### 151.1.1.1.1 ^1^ Clinic of Small Animal Medicine, Centre for Clinical Veterinary Medicine Munich Germany; ^2^ Ghent University Merelbeke Belgium; ^3^ Bioscientia Healthcare GmbH Ingelheim Germany; ^4^ Evidensia DACH Giessen Germany

The rectal temperature is a crucial parameter during general examination in sick cats. Increased or decreased temperature is an important finding that, combined with the further clinical, laboratory and imaging results, helps to obtain a diagnosis. In cats, only few studies report on the reference range of rectal temperature, whereas non were based on a large group.

The aim of this retrospective study was to define a reference interval of rectal temperature in adult cats based on a mixed data set of diseased and non‐diseased animals.

Rectal temperature of adult (>1 year old) cats presented at a referring‐hospital was measured using a digital predictive rectal thermometer. The reference interval was calculated using an algorithm developed by the Deutsche Gesellschaft für Klinische Chemie und Laboratoriumsmedizin e.V. (DGKL) in its Reference Limit Estimator software (https://www.dgkl.de). Normal rectal temperature was defined as being within a lower limit of 1.5*Inter‐Quartile Range (IQR) ‐lower quartile and an upper limit of 1.5*IQR +upper quartile. Normal distribution was tested with the Anderson‐Darling Test. Data was evaluated non‐ parametrically using the Kruskal‐Wallis‐Test.

Originally, temperature was measured in 5101 cats between 2007 and 2017. Filtering for >1 year‐old individuals left data of 4818 cats for analysis. Results of 30 cats were also excluded as they were outside of expected temperature values (<33°C or > 43°C). Overall, 524 cats were identified as outliers based on the 1,5 x Interquartile Range Rule, leaving 4263 cats for statistical analysis. The lower reference limit was calculated as 37.1°C (95% CI 37.1 – 73.1°C) and the upper reference limit was calculated as 39.6°C (95% CI 39.6 – 39.6°C).

The results of this study showed that calculating reference intervals on clinical date is feasible using a mixed data set and results are comparable to existing values. Based on this study, the reference interval for rectal temperature in adult cats is mildly wider compared to commonly used values.

**Disclosures**

No disclosures to report

## 152 ESVIM‐P‐10

### 152.1 ESVIM ‐ European Society of Veterinary Internal Medicine

#### 152.1.1 Degenerative condition in lower airway cartilage as predisposing factor for recurrent bacterial bronchopneumonia in Irish Wolfhounds

##### 152.1.1.1 V. Hukkinen, K. Kegler, S. Viitanen

###### 152.1.1.1.1 Helsinki Finland

High incidence of recurrent bacterial bronchopneumonia has been reported in Irish Wolfhounds (IWHs) without evident immune deficit or primary ciliary disease. To date, breed‐specific predisposing factors contributing to this condition are largely unknown, but predisposition to bronchiectasis development has been reported. Congenital and acquired defects in hyaline cartilage may lead to airway remodeling and bronchiectasis development, and as such could play a role in recurrent bronchopneumonia in IWHs.

The aim of this study was to evaluate the structural integrity of airway cartilages in IWHs with previous pneumonia (n=7, median age 6.0 years, interquartile range (IQR) 4.8–8.6 years; median number of previous pneumonias 4, IQR 2‐5), in IWHs without pneumonia (n=4, median age 6.1 years, IQR 5.2–8.0 years) and in control animals (n=5, median age 9.9 years, IQR 8.4–13.7 years) comprising large‐breed dogs with no history of respiratory disease. Histological sections of the tracheal, main and intrapulmonary bronchial cartilages from all dogs were stained with hematoxylin and eosin (H&E) to assess the overall structure and chondrocyte integrity. Safranin O and Periodic Acid‐Schiff (PAS) stains were used to detect proteoglycans, and toluidine blue stain for glycosaminoglycan and degree of metachromasia. Degenerative cartilage changes were identified commonly in IWHs (both with and without pneumonia) but in none of the control dogs.

Herein we hypothesize that breed‐specific degenerative changes in the hyaline cartilage of lower airways contribute to the pathogenesis of bronchiectasis development and recurrent bronchopneumonia in IWHs.

**Disclosures**

Disclosures to report, please report below

V.M. Hukkinen has received research grants from the Finnish Foundation of Veterinary Research (Suomen Eläinlääketieteen säätiö, SELS), the Finnish Veterinary Research Foundation (Eläinlääketieteen tutkimuksen tukisäätiö, ETTS), and the Finnish Kennel Club.

## 153 ESVIM‐P‐11

### 153.1 ESVIM ‐ European Society of Veterinary Internal Medicine

#### 153.1.1 Are clinical signs predictive of the aetiology of chronic nasal diseases in dogs?

##### 153.1.1.1 M Garcia Liotard ^1^, V Mariaud ^2^, N Noél ^2^, B Houdellier ^2^, M Harel ^3^, J Dahan ^2^, M Cervone ^1^

###### 153.1.1.1.1 ^1^ Clinique vétérinaire Evolia L'Isle Adam France; ^2^ VetOption Aix‐en‐Provence France; ^3^ Sonhar Ecully France

The clinical determination of the etiology of chronic nasal diseases (CND) in dogs is challenging. The aim of this study was to compare the clinical presentation among the most frequent CND and to assess the utility of a new clinical score (the “Canine Nasal Disease Activity Index”; CNDAI) to predict the etiology of CND in dogs.

Dogs diagnosed with CND between 2020 and 2022 among two referral centers were retrospectively included. Signalment, clinical presentation, imaging and rhinoscopy, and clinicopathological findings were recorded. Dogs were divided into 5 groups, based on the final diagnosis: inflammatory (IR), neoplastic (NR) and mycotic (MR) rhinitis, nasal foreign body (NFB) and oro‐nasal fistula (ONF). The CNDAI was calculated by adding one point for each recorded clinical sign, including lethargy, inspiratory dyspnea, open mouth breathing, respiratory noises, nasal discharge, epistaxis, sneezing, reverse sneezing, diminished nasal permeability, facial deformity, loss of *planum nasale* pigmentation, exophthalmos, dysphagia, and seizures. The frequency of each clinical sign was compared between groups using the Fisher exact test or the Chi‐square test. The mean age, the mean body weight (BW), and the mean CNDAI were compared between groups with one‐way ANOVA and Tukey post‐hoc analysis. Significance level was set at p=0.05.

Overall, 69 dogs were included. Twenty‐one dogs had IR, 21 had NR, 9 had MR, 11 had NFB and 7 had ONF. The mean age was significantly different between groups (F=4.59;p=0.002), and specifically between MR *versus* NR (p=0.04), MR *versus* ONF (p=0.006), and ONF *versus* NFB (p=0.02). The mean BW was not significantly different between groups. Respiratory noises were less frequent in dogs with IR compared to dogs with NR (p=0.05), and dogs with NFB (p=0.005); nasal discharge was more frequent in dogs with MR *versus* dogs with NR (p=0.03) and dogs with IR (p=0.03); reverse sneezing was more frequent in dogs with IR *versus* MR (p=0.03); epistaxis was more frequent in dogs with NR *versus* IR (p=0.01), and in dogs with NFB *versus* IR (p=0.03); diminished nasal permeability was more frequent in dogs with NR *versus* IR (p=0.0002). Although the mean CNDAI was significantly different between groups (F=2.69; p=0.04), the post‐hoc analysis failed to confirm such significance.

This study further suggests a diagnostic challenge in determining a specific diagnosis for CND based on the clinical presentation only. Although some differences in clinical presentation were found between groups, the proposed CNDAI failed to differentiate different nasal diseases from each other.

**Disclosures**

No disclosures to report

## 154 ISCAID‐P‐2

### 154.1 ISCAID ‐ International Society for Companion Animal Infectious Diseases

#### 154.1.1 Immune responses of 4321 European dogs to Borrelia antigens (2019‐2021)

##### 154.1.1.1 D. Breu ^1^, C. Wenk ^2^, E. Müller ^1^

###### 154.1.1.1.1 ^1^ Laboklin GmbH & Co KG Bad Kissingen Germany; ^2^ Laboklin GmbH & Co KG Basel Switzerland

The study aimed to evaluate the spectrum of immune responses from serum samples of 4321 European dogs clinically suspected of *Borrelia burgdorferi* infection to a *Borrelia* Immunoblot. The *Borrelia* Immunoblot was performed employing 9 recombinant antigens.

Among them, C_6_ peptide (C_6_) represents the invariable sequence (IR_6_) of the VlsE lipoprotein (VlsE/variable major protein‐like sequence, expressed). Both C_6_ and VlsE antigens were described to be specific for active infection whereas they're absent in vaccinated dogs. OspC (outer‐surface protein C) has been acknowledged as early marker of exposure/re‐exposure in non‐vaccinated dogs. OspC is detectable by 2 weeks after infection and wanes after 3‐5 months without natural booster. Antibodies against OspA (outer‐surface protein A) are usually seen after vaccination.

Applying the evaluation criteria of the Immunoblot manufacturer, 64.5% of dogs were negative (≤ 1 band positive, except OspA, C_6_/VlsE) and 22.6% positive (C_6_^+^ and/or VlsE^+^ or > 4 other bands, OspA excluded). 9.3% showed band patterns of vaccination (OspA^+^ and/or together with ≤ 3 other bands, except VlsE^+^/C_6_^+^). A small fraction (1.2%) showed a pattern for vaccination plus infection (OspA^+^, OspC^+^ and/or VlsE^+^ or OspA^+^ plus ≥ 4 other bands). In 2.4% of dogs, band patterns were borderline presenting 2‐3 positive bands (excluding OspA^+^, C_6_^+^/VlsE^+^).

Antibodies against single proteins were found at different frequencies as follows: p18 (DbpA/ Decorin‐binding protein A, 13.5%), p23 (OspC‐mix, 8.6%), p31 (OspA‐mix, 10.6%), p39 (BmpA/Borrelia membrane protein A, 12.8%), p41 (Flagellin, 17.5%), p58 (14.3%), C_6_ (20%), VlsE (19%) and p83/100 (31.9%).

Antibodies against C_6_^+^ and VlsE^+^ were found in a total of 865 (20%) and 822 (19%) dogs, respectively. The dogs showing consistent positive results to both antigens (C_6_^+^/ VlsE^+^) were 65.1% (665/1022), while VlsE^+^/C_6_^‐^ was 15.4% (157/1022) and C_6_^+^/VlsE^‐^ was 19.6% (200/1022). Antibodies unique to OspC ‐ an early marker of exposure ‐ were found in 30 (0.7%) dogs. Antibodies to OspC combined with either VlsE^+^ or C_6_^+^ were detected in 229 (5.3%) dogs, indicating infection and/or re‐exposure to the pathogen.

With regard to breeds (>100 individuals), Bernese mountain dogs were most frequently positive to VlsE^+^/C_6_^+^ (~65%/65%), followed by Golden Retrievers (26%/19%) and Labradors (16%/14%).

Our data showed that immune responses to the infection‐specific antigens (VlsE/C_6_) varied widely with inconsistent results in 35%. Furthermore, substantial, breed‐specific differences were observed in anti‐VlsE/C_6_ responses. Antibody responses to OspC are likely to provide complementary information in assessing and staging early/reinfection in non‐vaccinated dogs.

**Disclosures**

Disclosures to report, please report below

Disclosures The authors are employed at the Laboklin GmbH & Co KG, Bad Kissingen, Germany (D. Breu) and Laboklin GmbH & Co KG Basel, Switzerland (C. Wenk). E. Müller is owner/manager of the Laboklin GmbH & Co KG, Germany.

## 155 ISCAID‐P‐3

### 155.1 ISCAID ‐ International Society for Companion Animal Infectious Diseases

#### 155.1.1 Evaluation of serum 25‐hydroxyvitamin D, c‐reactive protein, and haptoglobin as biomarkers in dogs with histoplasmosis

##### 155.1.1.1 J. Jaffey ^1^, B. Hernandez ^2^, L. Cohn ^3^, R. Backus ^3^, K. KuKanich ^4^, A. Hanzlicek ^5^, V. Parker ^2^, M. White ^1^, R. Ringold ^6^, E. Westerback ^1^, L. Freilich ^4^

###### 155.1.1.1.1 ^1^ Midwestern University College of Veterinary Medicine Glendale United States; ^2^ Ohio State University College of Veterinary Medicine Columbus United States; ^3^ University of Missouri College of Veterinary Medicine Columbia United States; ^4^ Kansas State University College of Veterinary Medicine Manhattan United States; ^5^ Oklahoma State University College of Veterinary Medicine Stillwater United States; ^6^ VDI Laboratory Simi Valley United States

Histoplasmosis, which can cause substantial morbidity and mortality, is one of the most common invasive systemic mycoses in dogs worldwide. Clinical disease can be limited to the lungs, or disseminate to extra‐pulmonary sites. Serum 25‐hydroxyvitamin (OH)D, c‐reactive protein (CRP), and haptoglobin are useful biomarkers for several infectious diseases and inflammatory disorders in dogs, but their utility in histoplasmosis is unknown. Therefore, we aimed to determine whether serum 25(OH)D, CRP, and haptoglobin concentrations were different in dogs with histoplasmosis compared to healthy controls and if any variables were associated with serum 25(OH)D concentration. Dogs were included in this prospective cohort study if they were diagnosed with histoplasmosis via identification of organisms on cytological or histopathological examination. Dogs were categorized based on organ involvement. Healthy control dogs were also included. Dogs were included after obtaining informed owner consent with IACUC approval or if leftover serum from other diagnostic purposes was available. Serum 25(OH)D concentrations were measured with modified‐HPLC. Serum CRP and haptoglobin were measured at a reference laboratory using commercial ELISA assays. Normally distributed continuous data were compared using Student's *t*‐tests and non‐normally distributed continuous data with Mann‐Whitney rank sum tests. Association analyses were performed using linear regression tests. P < 0.05 was significant. Twelve dogs with histoplasmosis were enrolled (disseminated, n = 8; GI tract, n = 4), and 10 healthy controls. Dogs with histoplasmosis had lower serum 25(OH)D concentrations than healthy controls (P < 0.001). Serum 25(OH)D concentrations did not differ in dogs that had histoplasmosis with and without GI tract involvement. Serum CRP (P < 0.001) and haptoglobin (P = 0.003) concentrations were higher in dogs with histoplasmosis than healthy controls. Serum 25(OH)D concentration was positively associated with fold change in serum albumin (r^2^ = 0.54; P < 0.001), and negatively associated with fold change in serum globulin (r^2^ = 0.43; P < 0.001) and serum CRP concentration (r^2^ = 0.35; P = 0.007). These results indicate that serum 25(OH)D, CRP, and haptoglobin could be valuable biomarkers in dogs with histoplasmosis. Future studies investigating their role in prognosis, assessing treatment response, and detecting relapse are warranted.

**Disclosures**

Disclosures to report, please report below

Randy Ringold is employed by VDI laboratory and this commercial laboratory offers c‐reactive protein and haptoglobin measurements in companion animals.

## 156 ISCAID‐P‐4

### 156.1 ISCAID ‐ International Society for Companion Animal Infectious Diseases

#### 156.1.1 Evolution over a 20‐year period of the predisposing factors and clinical outcomes of dogs with leishmaniosis: Are hosts getting weaker or parasites stronger?

##### 156.1.1.1 C. Rodriguez‐Sanz, J. Sarquis, G. Miró

###### 156.1.1.1.1 Complutense University of Madrid Madrid Spain

The treatment of canine leishmaniosis (CanL) is more and more challenging mainly due to the emerging anti‐leishmanial drug resistances. However, a wide range of factors can affect host‐parasite interactions and result in high selective pressure, often associated with reciprocally induced changes in both host and parasite populations.

Here, we carried out a retrospective study of the clinical data of 28,818 dogs attending a Veterinary Teaching Hospital, between 2000‐2020. Our main goals were to identify predisposing factors to acquire the infection and/or develop severe forms of disease (stages III‐IV, based on LeishVet guidelines); and to evaluate the evolution of these predisposing factors and outcomes of dogs with leishmaniosis throughout the last two decades. Binary (sex, prophylaxis measures, habitat) and multivariable (age, breed, clinical stage, outcome) logistic regression models were built using R (cutoff p‐value < 0.05). The diagnosis of CanL was established based on compatible clinical signs, quantitative serology, cytology and/or PCR results. Favorable outcome was considered in those dogs that improved their clinical stage after treatment and never develop clinical signs again; while unfavorable outcome was defined as progression of clinical signs and clinicopathological disorders despite of treatment/s, or natural death/euthanasia due to CanL. The outcome of dogs whose clinical stage did not vary after treatment/s was defined as stable.

A total of 1,194 dogs were diagnosed with CanL at the Consultant of Infectious Diseases. The statistical analysis resulted in the identification of breed and habitat as predisposing factors to acquire the infection and to develop clinical stages III‐IV. Surprisingly, prophylaxis measures were not associated with a reduction in the acquisition of the infection (p‐value = 0.37) or the severity of the disease (p‐value = 0.50), although we detected a significant increase in their use. We also observed a significant increase in the frequency of dogs living indoors, which was accompanied by a significant decrease in the frequency of dogs with advanced clinical stages. Nevertheless, the frequency of dogs with unfavorable outcomes significantly increased in the last years. This could be partially explained by the fact that certain breeds here identified as predisposed to develop clinical leishmaniosis were significantly overrepresented during the last years. Variations in dog breed populations, which may display slightly different immune responses, could also favor the co‐adaptation process of parasites, forcing them to develop new strategies of evasion and, thus, increasing their fitness. However, genomic studies on *L. infantum* isolates are necessary to confirm this hypothesis.

**Disclosures**

No disclosures to report

## 157 ISCAID‐P‐5

### 157.1 ISCAID ‐ International Society for Companion Animal Infectious Diseases

#### 157.1.1 Molecular and serological detection of Anaplasma phagocytophilum in dogs from Germany (2008‐2020)

##### 157.1.1.1 I. Schäfer, B. Kohn, C. Silaghi, S. Fischer, C. Marsboom, G. Hendrickx, E. Müller

###### 157.1.1.1.1 Laboklin GmbH & Co. KG. Bad Kissingen Germany

*Anaplasma* (*A*.) *phagocytophilum* is an obligate intracellular bacterium causing granulocytic anaplasmosis. Ticks of the *Ixodes persulcatus* complex are the primary vectors, in Germany mainly *Ixodes ricinus*. The aim of the study was to assess the percentage of positive test results for *A. phagocytophilum* in dogs living in Germany, to identify possible risk factors for infection and to analyze potential abnormalities in the complete blood count (CBC).

Results of direct (polymerase chain reaction [PCR]) and indirect (immunofluorescence antibody test [IFAT], antibody enzyme‐linked immunosorbent assay [ELISA]) detection methods requested by German veterinarians between 2008 and 2020 from a commercial laboratory were included in the retrospective study. Dogs were included in the hematological analysis, if a CBC was available combined with positive PCR results.

In total, 25,724/110,240 dogs (23.3%) tested positive by PCR (1,332/27,368 dogs; 4.9%) and/or IFAT/ELISA (24,720/90,376 dogs; 27.4%). The months with highest PCR incidences overlapped with the ones with highest vector activity. Four weeks delayed to PCR‐testing, peaks in serology were observed in positive tested dogs. Male and elder dogs had a higher likelihood of being tested serologically positive. A CBC was available in 706 dogs (thrombocytopenia 66%, anemia 58%, leukopenia 19%, unremarkable 13%). Pancytopenia was present in 66/706 dogs (9%).

Dynamic of infections with *A. phagocytophilum* in dogs in Germany is consistent with peaks in vector activity. Hematological abnormalities are common in dogs infected with *A. phagocytophilum*. The rising amounts of dogs tested positive from 2008 to 2020 indicate an increasing importance of canine granulocytic anaplasmosis in Germany.

**Disclosures**

Disclosures to report, please report below

Ingo Schäfer and Elisabeth Müller are members of the LABOKLIN laboratory, offering the diagnostic tests on a fee‐for‐service‐basis.

## 158 ISCAID‐P‐6

### 158.1 ISCAID ‐ International Society for Companion Animal Infectious Diseases

#### 158.1.1 Aelurostrongylus abstrusus prevalence in cats from Spain

##### 158.1.1.1 A. Rodriguez‐Cobos ^1^, R. Rodon ^2^, S. Atencia ^1^

###### 158.1.1.1.1 ^1^ VETSIA Veterinary Hospital Leganés Spain; ^2^ IDEXX Laboratories Barcelona Spain

The metastrongiloid *Aelurostrongylus abstrusus* is a nematode that infects the terminal bronchioles, alveoli and alveolar ducts of cats. Commonly known as feline lungworm, several studies have reported its prevalence around the world, which can vary from 1% to 50%. In Spain, only one study documented the prevalence of *A. abstrusus* in a mixed population of cats using the Telemann method, estimating it as 1%.

The aim of this study was to retrospectively describe the prevalence of *A. abstrusus* in cats in Spain, and to determine the distribution of positive cases based on Baermann technique testing.

The results of 1041 Baermann tests performed at IDEXX Reference Laboratories in Spain over a 6‐year period, from 2017 to 2022, were obtained. Samples were grouped by season of diagnosis (spring – March 21^st^, summer – June 21^st^, autumn – September 21^st^, and winter – December 21^st^), by postcode of origin, and by geographic area.

Eighty‐three out of 1124 samples (7.8%; 95% CI 6.3‐9.6) were positive for *A. abstrusus*. The season with the highest rate of positive samples was winter (29/203 samples, 9.6%), followed by summer (19/214, 8.2%), spring (16/192, 7.7%) and autumn (19/278, 6.4%). No significant variation on seasonality was found (χ2 <0.553). The region with the highest positive rate (PR) was the Northwest, with a PR of 15.38% (95% CI 4.06‐26.47), and the region with the lowest PR was central Spain, with 6.11% (95% CI 3.45‐8.77). No significant differences were found between regions (χ2 <0.94).

In conclusion, the overall prevalence of *A. abstrusus* in Spain determined in this study was 7.8%. This is substantially higher than previously reported. This could be explained by two factors: Firstly, the latest study used a more accurate technique for detecting *A. abstrusus* larvae. Secondly, the population of cats included had presented for investigation of respiratory signs, whereas the previous prevalence study's population of cats was randomly selected.

Neither the season of infection, nor the geographic location within Spain, were found to have a significant impact on the positive rates.

**Disclosures**

Disclosures to report, please report below

‐ Alfredo Rodríguez‐Cobos's ECVIM‐CA Residency programme is partially funded by Idexx Laboratories ‐Jaume Rodon is employee at Idexx Laboratories as Medical Director

## 159 ISCAID‐P‐7

### 159.1 ISCAID ‐ International Society for Companion Animal Infectious Diseases

#### 159.1.1 Prognostic factors for in‐hospital mortality in dogs with clinical leishmaniosis ‐ a retrospective study

##### 159.1.1.1 C. Molina ^1^, M.J. Dias ^2^, T.D. Domingues ^3^, R. Leal ^2^

###### 159.1.1.1.1 ^1^ Veterinary Teaching Hospital ‐ Faculty of Veterinary Medicine ; ULisboa Lisboa Portugal; ^2^ Centre for Interdisciplinary Research in Animal Health, Fac.Vet.Med., ULisboa Lisbon Portugal; ^3^ Department of Statistics and Operations Research ‐ Faculty of Sciences, U.Lisboa Lisbon Portugal

Medical management of Canine Leishmaniosis (CL) can be challenging, often requiring dog's hospitalization. It is known that azotemia, anemia and Leishvet clinical staging are negative prognostic factors for the standard management of the disease. However, literature concerning in‐hospital outcome of CL is scarce.

This study aims to assess prognostic factors for in‐hospital mortality in CL.

A retrospective case‐control study was conducted. Medical records of dogs hospitalized due to clinical Leishmaniosis in a Portuguese Veterinary Teaching Hospital, between August 2018 and January 2022 were reviewed. Data concerning medical history, clinical signs, laboratory findings, Leishvet clinical staging, and outcome (discharge versus death) was assessed. Dogs were excluded if data was unavailable or incomplete. Descriptive and non‐parametric tests were used. Categorical variables were compared using a Fisher's Exact Test (p<0.05).

A total of 31 dogs were included: 18 females (12/18; 67% neutered) and 13 males (10/13; 77% intact) with a median age of 7 years old (IQR=7). The mean weight was 22.3Kg (±10); 27/31 (87%) were purebred. Labrador Retriever (n=4), Boxer (n=3) and Border Collie (n=3) were the most represented breeds. Eleven dogs (11/31; 36%) were classified as Leishvet stage II/IV, while ten (10/31; 32%) were at stage III/IV and ten (10/31; 32%) at stage IV/IV. Lethargy was observed in 26/31 (84%) of the dogs, followed by pale mucous membranes (20/31; 65%) and weight loss (15/31; 48%). Hypoalbuminemia was the most prevalent laboratory finding (26/31; 84%) followed by thrombocytopenia (22/31; 71%) and anemia in (21/31; 68%). Regarding outcome, 17/31 (55%) of the cases were discharged while 14/31 (45%) of dogs died during hospitalization. Nine dogs (9/14; 64.3%) were submitted to euthanasia whilst the remaining (5/14; 35.7%) died from cardiorespiratory arrest. Leucocytosis (p<0.01), azotemia (p=0.012), hypoalbuminemia (p=0.048), and Leishvet stage IV classification (p=0.027) were significantly associated with a higher in‐hospital mortality.

This study highlights that mortality is elevated in dogs with Leishmaniosis requiring in‐hospital management, as approximately half of them die or are submitted to euthanasia. Purebred adult dogs were overrepresented among those requiring hospitalization. Leucocytosis, hypoalbuminemia and azotemia can be considered negative prognostic factors for in‐hospital mortality in dogs with Leishmaniosis. Leishvet staging is a well‐known negative prognostic factor for the long‐term management of CL and it also seems to affect the in‐hospital outcome, stressing the relevance of an appropriate staging of these dogs.

**Disclosures**

Disclosures to report, please report below

No conflict of interest to declare. This work was supported by FCT – Fundação para a Ciência e Tecnologia IP, grant UIDB/00276/2020

## 160 ISCAID‐P‐8

### 160.1 ISCAID ‐ International Society for Companion Animal Infectious Diseases

#### 160.1.1 Biomolecular investigation for Capillaria spp. infections on bronchoalveolar lavage fluid of owned domestic dogs presented for chronic cough in Belgium

##### 160.1.1.1 E Roels ^1^, M Schnyder Gasparoli ^2^, S Vroomen ^3^, M Garigliany ^3^, C Clercx ^3^

###### 160.1.1.1.1 ^1^ University of Liège Liège Belgium; ^2^ Vetsuisse Faculty, University of Zurich Zurich Switzerland; ^3^ Faculty of Veterinary Medicine, University of Liège Liège Belgium

The trichuroid parasitic nematode *Capillaria aerophila* (syn. *Eucoleus aerophilus*) is responsible for lower respiratory infections and *Capillaria boehmi* (syn. *Eucoleus boehmi*) for sino‐nasal infections in wild and domestic carnivores. Animals become infected by eating environmental embryonated eggs or earthworms. The adult worms live embedded in the epithelia of the bronchioles, bronchi, and trachea or in the nasal sinuses, respectively. Infections with *C. aerophila* can be sub‐clinical or lead to chronic bronchial inflammation, rarely bronchopneumonia. *C. boehmi* may cause nasal discharge, sneezing or olfactory impairment. Knowledge about prevalence and distribution of both parasites beyond Eastern Europe and Mediterranean countries is lacking. The aim of this study was to assess the prevalence of *C. aerophila* infection in coughing, client‐owned, domestic dogs in Belgium. Stored bronchoalveolar lavage fluid (BALF) from 125 dogs (median age 7.3 years, range: 0.3‐17.2 years) from March 2018‐2022 was retrieved. All dogs had history of chronic cough (> 2 weeks duration) and underwent BALF collection for microbiologic testing of common respiratory pathogens. DNA extracted from BALF samples was stored at ‐80°C until further analysis. A conventional polymerase chain reaction targeting a region internal to the *cox1* gene of *C. aerophila*, a Capillarinae consensus sequence, was performed on BALF samples in duplicate and in batch analysis using previously published primers sequences. DNA of adult *C. boehmi* specimens was included as a positive control. Molecular grade water was used as a negative control. Neither DNA of *C. aerophila* nor *C. boehmi* were detected in the BALF samples. Sixty‐seven dogs (54%) had a recent history of deworming against lungworms with either moxidectin or fenbendazole (deworming protocol not standardized), 9 dogs (7%) were not up to date with deworming therapy, and the remaining 49 dogs (39%) had unknown deworming status. Result of this study suggest that *C. aerophila* infection is not prevalent in Belgium in dogs with chronic cough. This might be explained by recent deworming therapy in half of the included dogs. Epidemiosurveillance of capillarid infection may be considered in wild canids, such as foxes, to determine whether these parasites are a potential risk for domestic animals.

**Disclosures**

No disclosures to report

## 161 ISCAID‐P‐9

### 161.1 ISCAID ‐ International Society for Companion Animal Infectious Diseases

#### 161.1.1 Primary and secondary aspergillosis in dogs

##### 161.1.1.1 S Rösch ^1^, G. Oechtering ^2^

###### 161.1.1.1.1 ^1^ Stiftung Tierärztliche Hochschule Hannover Hannover Germany; ^2^ Kleintierklinik Leipzig Germany

In dogs with sinonasal aspergillosis (SNA) it seems to be crucial for prognosis and therapy whether SNA occurs alone or together with another nasal cavity pathology (NCP) and whether the frontal sinus is affected.

Thirty dogs with SNA included in this retrospective study were divided into group 1: without any other NCP and group 2: with another NCP (e.g., dental root disease, nasal foreign body). Patient reports were reviewed. Data from medical history, computed tomography, endoscopy videos, laboratory, interventional endoscopic therapy, and follow‐up examinations were analyzed.

19/30 patients (63 %) had aspergillosis without any additional NCP (group 1). 15/19 dogs (79 %) showed sinusitis and the number of follow‐up examinations was twice as high in these dogs as in patients without sinusitis. Dogs without sinusitis did not require repeated interventional endoscopic therapy.

11/30 dogs (37 %) had additional NCP (group 2). While in 9/11 dogs (82 %) aspergillosis was limited to the nasal cavity, only 2/11 dogs (18 %) also had sinusitis. Treatment of the underlying disease, debridement and antifungal therapy in 9/11 (82 %) dogs resulted in complete cure in 10/11 dogs (91 %). Only one patient with additional sinusitis required more than one treatment.

In group 2, mycosis was confined to the nasal cavity in the absolute majority of cases. Localization and the better course of therapy support the hypothesis of a secondary aspergillosis. We refer to SNA without other findings as primary SNA. It is predominantly associated with sinusitis and has a much worse prognosis.

**Disclosures**

No disclosures to report

## 162 ESVONC‐P‐1

### 162.1 ESVONC ‐ European Society of Veterinary Oncology

#### 162.1.1 Metronomic chlorambucil as treatment for canine solid tumours

##### 162.1.1.1 F. Lyseight, C. Pittaway

###### 162.1.1.1.1 Dick White Referrals Cambridgeshire United Kingdom

Metronomic chlorambucil has been reported to have antitumor activity, particularly in urothelial cell carcinomas. There is only a single study addressing the clinical benefit in other subtypes of natural occurring cancer in 36 dogs. The aim of this study was to determine the response and adverse effects in dogs with solid tumours treated with metronomic chlorambucil.

Dogs were included that had received chlorambucil at a dose of 4 mg/m^2^ orally once daily for at least 4 weeks at a single referral hospital between 2018 and 2021. Dogs could be concurrently treated with other medications normally found in metronomic protocols, eg. thalidomide or NSAIDs. Dogs with urothelial cell carcinomas and mast cell tumours were excluded. Response was evaluated according to RECIST criteria v 1.0 and adverse effects according to VCOG‐CAE v 1.1.

Thirty‐seven dogs met the inclusion criteria. There were 13 types of neoplasia; apocrine gland anal sac adenocarcinoma (n=8); melanoma (n=5); and hemangiosarcoma (n=4) were the most common. Twenty‐two dogs also received an NSAID (n= 22) and/or thalidomide (n=11). Chlorambucil was prescribed as first line treatment in 19% (n= 7), as primary adjuvant treatment in 24% (n=9), and as a rescue in 57% (n=21) of cases. Six dogs received chlorambucil after developing sterile haemorrhagic cystitis whilst receiving cyclophosphamide. Median treatment duration was 100 days (range 29 – 289 days) with a median follow‐up time of 190 days (range 29 – 841 days) from start of treatment.

No dogs had a partial or complete response to treatment. Thirty‐four dogs had stable disease with a median time to progression of 80 days (range 38 – 512 days). Twenty‐one (61%) of these dogs developed progressive disease. Dogs receiving chlorambucil as first line or as first adjuvant treatment had similar median time to progression as rescue cases (76 days vs 72 days, respectively). Interestingly, of the cases started on chlorambucil due to side‐effects to cyclophosphamide only one in six developed progressive disease (at day 108).

Six dogs had adverse effects, including three which required treatment discontinuation: one developed grade 2 polyuria/polydipsia at 101 days and two developed grade 2 thrombocytopenia at day 160 and day 272 respectively.

No dogs achieved had a complete or partial response. Time to progression in the present study was similar to that previously reported. Chlorambucil is a well‐tolerated treatment that may be appropriate in cases that require metronomic chemotherapy but where there are concerns about the use of cyclophosphamide.

**Disclosures**

No disclosures to report

## 163 ESVONC‐P‐2

### 163.1 ESVONC ‐ European Society of Veterinary Oncology

#### 163.1.1 Electrochemotherapy with carboplatin for the treatment of feline squamous cell carcinoma: a pilot study regarding safety and effectiveness.

##### 163.1.1.1 M. Lajoinie, T Chavalle, G Bernardo Marques, G Chamel, F Ponce

###### 163.1.1.1.1 VetAgro Sup Marcy‐l'étoile France

Electrochemotherapy (ECT) is a procedure aiming to increase cytotoxic agent concentration in a selected area through electroporation. It is an efficient conservative treatment for feline squamous cell carcinoma and is commonly performed with bleomycin. However, this molecule is not available in all countries. Other molecules that can be used for ECT include platinum‐based agents. The use of cisplatin is reported; though, the use of carboplatin has never been evaluated to date. The aim of our pilot study was to assess the safety and preliminary efficacy of electrochemotherapy with carboplatin for the treatment of feline squamous cell carcinomas. Five cats with histologically diagnosed cutaneous squamous cell carcinomas and no regional or distant metastasis were included. Median tumoral surface was 78.5 mm^2^. Tumors were classified as stage T1N0M0 (4/5) or T2N0M0 (1/5). All cats received carboplatin intravenously (145‐200 mg/m^2^, median: 170 mg/m^2^) and intratumorally (1.17‐5.94 mg/cm^2^, median: 3.37 mg/cm^2^) at a total dosage of 190‐240 mg/m^2^, followed by electroporation with a dedicated device (Electrovet EZ v3.0, LeroyBiotech®; 8 orthogonal pulses at 1300 V/cm). Only one cat presented general toxicity with VCOG‐CTCAE grade 3 anorexia. All cats presented erythema and crusting; no cat presented signs of local pain. Two cats presented sneezing during the first 10 to 15 days. Response was assessed 6 weeks after ECT performance, and all cats presented CR: after one session in 4/5 cats and after two sessions in the remainder. Regarding follow‐up: four cats were still in remission at data closure (2, 9 and 15 months after ECT session) or at time of death from another cause (7 months after ECT session) and, one cat presented a recurrence 11 months after the first ECT session. Finally, electrochemotherapy with carboplatin appears to be well tolerated when used for the treatment of cats with cutaneous squamous cell carcinoma. Preliminary response rates are promising; however, further studies are warranted to assess the efficacy, to standardize and refine the procedure.

**Disclosures**

No disclosures to report

## 164 ESVE‐P‐1

### 164.1 ESVE ‐ European Society of Veterinary Endocrinology

#### 164.1.1 Evaluation of the accuracy of an alternative cortisol immunoassay as a screening test for canine hypoadrenocorticism.

##### 164.1.1.1 J. Bacon ^1^, P. Graham ^2^, A. Hrovat ^3^, S.K. Kilpatrick ^4^, N. Mann ^5^, H. Swales ^6^

###### 164.1.1.1.1 ^1^ Stockton‐on‐Tees United Kingdom; ^2^ University of Nottingham Nottingham United Kingdom; ^3^ Pride Veterinary Centre Derby United Kingdom; ^4^ Veterinary Thought Exchange Glasgow United Kingdom; ^5^ Wear Referrals Stockton‐on‐tees United Kingdom; ^6^ Moorview Referrals Cramlington United Kingdom

The TOSOH AIA360 is an in‐practice, bench‐top, veterinary endocrinology analyser which can measure canine serum or plasma cortisol using an immunoassay (IA). Although previous studies have demonstrated that basal cortisol concentrations can have high sensitivity when used as a screening test for canine hypoadrenocorticism (HA), these studies were conducted using the Siemens Coat‐a‐count® radioimmunoassay or the Immulite® chemiluminescent assays. Consequently, further research is warranted to assess whether the cut‐offs derived from these studies are applicable to other cortisol immunoassays such as that used by the TOSOH AIA360.

The objective of the study was to assess the diagnostic performance of the TOSOH AIA360 cortisol IA as a screening test for canine HA. Medical records from two referral hospitals were searched retrospectively. Dogs were included if they underwent an ACTH stimulation test owing to a clinical suspicion of HA between June 2015 and October 2019. Dogs were excluded if there was a clinical suspicion of hypercortisolism or had received medications known to affect the pituitary‐adrenal axis in the preceding four weeks. The cut‐point corresponding to maximum diagnostic accuracy was calculated alongside the accuracy of previously reported cut‐offs.

173 dogs were included in the study. 20 dogs met the criteria for HA and 153 for non‐adrenal illness (NAI). Median cortisol in the HA group was 10nmol/L (IQR: 0‐16) and 55nmol/L (IQR: 35‐98) in the NAI group. Using previously reported cut‐offs, a basal cortisol concentration less than 55nmol/L had a sensitivity and specificity of 100% and 50% respectively for the diagnosis of hypoadrenocorticism. A basal cortisol concentration less than 22nmol/L had a sensitivity and specificity of 100% and 94% respectively for the diagnosis of hypoadrenocorticism. An optimised threshold for basal cortisol in dogs with hypoadrenocorticism using the TOSOH AIA360 of 17nmol/L corresponded to a sensitivity and specificity 97% and 98% respectively.

In conclusion, the results of this study suggest that an ACTH stimulation test need only be performed if the basal cortisol concentration is less than 17nmol/L when using the TOSOH AIA360 cortisol immunoassay to screen for canine hypoadrenocorticism.

**Disclosures**

Disclosures to report, please report below

Peter Graham is an employee of the University of Nottingham and is a consultant for Nationwide Laboratories. Harry Swales is an employee of IVC Evidensia. He has received speaker fees/remuneration unrelated to the subject matter from University of Liverpool, Veterinary Thought Exchange, Improve International, North East Veterinary Association, Royal Army Veterinary Corps, BSAVA, and VetsNow. Jessica Bacon is an employee of Linnaeus Veterinary Limited. She works at a centre where the alternative immunoassay is used. Nicholas Mann is an employee of Linnaeus Veterinary Limited. He works at a centre where the alternative immunoassay is used.

## 165 ESVE‐P‐2

### 165.1 ESVE ‐ European Society of Veterinary Endocrinology

#### 165.1.1 Serum Amyloid A concentration at diagnosis as a predictor of clinical remission in cats with diabetes mellitus: a retrospective study of 53 cases

##### 165.1.1.1 L. Lecot, M. Hugonnard, T. Buronfosse, T. Bouzouraa

###### 165.1.1.1.1 Péronnas France

Diabetes mellitus (DM) is common in cats. Remission can occur, especially if insulin resistance factors are detected and managed promptly. DM is also recognized as an inflammatory condition. Serum Amyloid A (SAA), which is a major acute phase protein in cats, is a helpful prognostic indicator in many inflammatory disease processes.

In the lack of knowledge regarding the usefulness of SAA as a prognostic marker in cats with DM, we aimed at describing SAA values at diagnosis in diabetic cats with or without comorbidities and at comparing these values between cats with favorable (remission) or unfavorable (no remission) outcomes.

SAA level was retrospectively measured on left‐over samples from diabetic cats submitted between January 2017 and 2020. Only cats treated with insulin therapy and with appropriate follow‐up over at least one‐year were enrolled. The cases were classified as having gone in remission (R) or not (NR). Remission was defined as euglycemia without insulin for > 4 weeks.

Dichotomous and continuous variables at diagnosis were compared between subgroups (R versus NR) using Fisher and Mann‐Whitney Wilcoxon tests, respectively. SAA was used as a dichotomous variable (increased (> 12mg/L) versus not increased). Significance was considered for p<0.05.

Fifty‐three cats were enrolled, including 26/53 (49%) treated with porcine lente insulin, 17/53 (32%) with insulin glargine, 9/53 (17%) with protamine zinc insulin and 1/53 (2%) with insulin detemir. Forty cats (75%) had at least one comorbidity: obesity (36/40), FIV (3/40), UTI (3/40), acromegaly (2/40), hyperthyroidism (2/40), pancreatitis (2/40), CKD (2/40), miscellaneous (6/40).

After one year of follow‐up, 11/53 (21%) cats had gone in remission and 42/53 (79%) were still under treatment.

Ten of 53 cats (19%) had an increased SAA at diagnosis, including 6/10 with comorbidities. There was no significant difference between SAA of cats with and without comorbidity at diagnosis (p=0,15). Remission was more likely in cats with increased SAA (p=0,0002).

This study suggests that increased SAA at diagnosis of DM was associated with remission within the first year after diagnosis. An increased SAA might be either due to pancreatic islet beta cells inflammation (induced by glucotoxicity and chronic hyperglycemia) or to concurrent inflammatory processes (infection, neoplasia, metabolic diseases). In cats with DM, the management of concurrent inflammatory conditions might help improving glycemic control and achieving remission.

An increased SAA at diagnosis of feline DM could be a putative positive prognostic factor of remission.

**Disclosures**

No disclosures to report

## 166 ESVE‐P‐3

### 166.1 ESVE ‐ European Society of Veterinary Endocrinology

#### 166.1.1 Tailored reference intervals for urinary corticoid:creatinine ratio in dogs based to the outcome of the low‐dose dexamethasone suppression test

##### 166.1.1.1 I. Schäfer, S. Rehbein, A. Holtdirk, T. Kottmann, R. Klein, E. Müller, K. Thoren‐Tolling

###### 166.1.1.1.1 Laboklin GmbH & Co. KG. Bad Kissingen Germany

Hyperadrenocorticism (HAC) is one of the most common canine endocrinopathies. Currently, the low‐dose dexamethasone suppression test (LDDST) is the screening test of choice for diagnosing spontaneous HAC. The diagnostic value of an increased urinary corticoid:creatinine ratio (UCCR) is discussed controversially.

The aim of this study was to determine cut‐off values for the UCCR‐test as the index test in comparison with the LDDST as clinical reference standard and to calculate sensitivity and specificity.

Data was obtained retrospectively from 2018‐2020 from a commercial laboratory. The LDDST and UCCR‐test were performed by automated chemiluminescent immunoassay (CLIA) in each dog considering timeframes in‐between both tests up to 14 days. The optimal cut‐off for UCCR‐testing was calculated by Youden‐Index. Sensitivity and specificity of the calculated reference intervals for the UCCR‐test and LDDST were assessed by Bayesian latent class models (BLCM) to compensate the LDDST as the clinical reference standard classified as an imperfect test for diagnosing HAC.

Three‐hundred‐and‐twenty‐four dogs were included in the study. An UCCR of 47.44 x 10^‐6^ was calculated as the optimal cut‐off. An UCCR < 40 x 10^‐6^ was classified as negative, 40‐60 x 10^‐6^ as questionable and > 60 x 10^‐6^ as positive. Using the 60 x 10^‐6^‐cutt‐off, BLCM revealed a sensitivity/specificity of 91%/54% (LDDST) and 86%/63% (UCCR‐test).

For diagnosing HAC, the UCCR‐test can be considered equal to LDDST looking at sensitivity with the advantage of being a non‐invasive test. However, LDDST should be considered as follow up in questionable outcomes of the UCCR‐test.

**Disclosures**

Disclosures to report, please report below

Ingo Schäfer, Ruth Klein and Elisabeth Müller are members of the LABOKLIN laboratory in Bad Kissingen (Germany) offering the diagnostic tests on a fee‐for‐service‐basis.

## 167 ESVE‐P‐4

### 167.1 ESVE ‐ European Society of Veterinary Endocrinology

#### 167.1.1 Development and validation of a questionnaire to assess health‐related quality‐of‐life in cats with hyperthyroidism

##### 167.1.1.1 F. Blunschi ^1^, I. Schofield ^2^, N. Bauer ^1^, K. Hazuchova ^1^

###### 167.1.1.1.1 ^1^ Small Animal Clinic Giessen Germany; ^2^ CVS (UK) Ltd Norfolk United Kingdom

Feline hyperthyroidism causes a wide range of clinical signs and thus the disease as well as its treatment is likely to affect the quality of life (QoL) of both cats and their owners. However, this impact has not yet been evaluated by objective methods.

A preliminary list of 28 questions considered to relate to either the health‐related quality‐of‐life (HRQoL) of hyperthyroid cats or to the impact their cat's disease might have on the owners’ daily lives was created. Each HRQoL question consisted of two sub‐questions: 1. to describe the frequency of that item (how often does the item apply); 2. to measure the impact of each item on the QoL (how strongly does the item affect QoL). A questionnaire was made available online in English and German to be completed by owners of cats with and without hyperthyroidism between November 2021 and February 2022. To develop the finalised HRQoL tool, the questions were refined based on statistical analysis of the questionnaire responses. Mann‐Whitney‐U tests were performed on each item, comparing the results from cats with and without hyperthyroidism. Items with at least weak evidence of a difference between the two groups (p<0.20) were retained because these were deemed to specifically relate to the impact of hyperthyroidism on a cat's HRQoL. Internal consistency and reliability of the questionnaire was measured by Cronbach's alpha. To assess the validity of the tool, Mann‐Whitney‐U test compared median HRQoL scores for cats with and without hyperthyroidism. P<0.05 was considered significant.

There were 551 owner‐completed questionnaires available for statistical analysis; 229 (41.6%) were by owners of cats with and 322 (58.4%) by owners of cats without hyperthyroidism. Twenty‐five out of 28 questions were retained within the final HRQoL tool which had an excellent internal consistency (α=0.92). The final HRQoL tool produced a score that ranges between 0 – 382 (lower scores meaning better QoL). The median HRQoL score was 87.5 (IQR 58 – 130.5, range 2 – 348) for cats with hyperthyroidism, and 27 (IQR 10 – 51, range 0 – 249) for cats without the disease (p<0.001), suggesting the QoL was poorer in hyperthyroid cats.

In conclusion, this validated HRQoL tool is useful to reliably quantify the influence of hyperthyroidism on the QoL of affected cats and their owners. In the future, this tool could be considered as a new approach in monitoring the success of the therapy of feline hyperthyroidism.

**Disclosures**

No disclosures to report

## 168 ESVE‐P‐5

### 168.1 ESVE ‐ European Society of Veterinary Endocrinology

#### 168.1.1 mRNA expression of canine glucocorticoid receptor α and P splice variants in peripheral blood samples of dogs with Cushing's syndrome

##### 168.1.1.1 J. Csöndes ^1^, B.M. Kállai ^2^, G. Kiss ^3^, Á. Máthé ^3^, N. Balogh ^1^, Z.S. Rónai ^2^, T. Mészáros ^2^

###### 168.1.1.1.1 ^1^ Praxislab Ltd. Budapest Hungary; ^2^ Department of Molecular Biology, Faculty of Medicine, Semmelweis University Budapest Hungary; ^3^ University of Veterinary Medicine Budapest Budapest Hungary

A new canine glucocorticoid receptor splice variant (cGR‐P) was recently detected at mRNA level in peripheral blood samples of healthy dogs and dogs suffering from Systemic Inflammatory Response Syndrome. The GR‐P is a truncated protein with a shortened ligand‐binding domain. Although the exact biological function of GR‐P is still not clarified even in humans, the hGR‐P is the second most abundant GR splice variant after hGRα in healthy subjects and in patients with Cushing's syndrome as well.

The objective of this study was to assess whether canine GR‐P is expressed at mRNA level in peripheral blood samples of dogs with Cushing's syndrome.

Blood samples obtained from 4 dogs diagnosed with Cushing's syndrome and 7 healthy control subjects were collected in Qiagen RNAprotect Animal Blood Tubes. Total RNA was extracted using RNeasy® Protect Animal Blood Kit and reverse transcribed to cDNA with RevertAid H Minus First Strand cDNA Synthesis Kit. The measurement of canine GRα and GR‐P mRNA expressions was performed with TaqMan‐based real‐time PCR assay in singleplex reactions. Ribosomal protein S5 was used as the reference gene and its expression was measured in separate parallel assays by SYBR Green chemistry. To calculate the relative change in cGRα and cGR‐P mRNA expression the comparative threshold cycle method was used. Two‐group comparisons were performed using Mann‐Whitney *U* test and correlation analysis with Spearman's rank correlation test. Blood samples were originally obtained for a research project on canine glucocorticoid receptor splice variants.

The median mRNA expression of cGRα was increased 5.9‐fold in dogs with Cushing's syndrome compared to healthy controls (*p* < 0.01). The median mRNA expression of the cGR‐P splice variant was increased 5.2‐fold in dogs with Cushing's syndrome compared to controls (*p* < 0.01). A strong correlation between cGRα and cGR‐P mRNA expression (*r*_*s*_ = 0.9749, *p* < 0.0001) was found. The ratio of cGRα/cGR‐P mRNA expression was not significantly different when the Cushing's disease cohort was compared to the control group (*p* = 0.5273).

In conclusion, cGRα and cGR‐P mRNA expression was elevated in peripheral blood samples of dogs with hypercortisolism. Interdependent expression of the investigated cGR splice variants presumes coregulation of these isoforms. The ligand binding capability and transactivation activity of the cGR‐P isoform is not yet known.

**Disclosures**

No disclosures to report

## 169 ESVE‐P‐6

### 169.1 ESVE ‐ European Society of Veterinary Endocrinology

#### 169.1.1 Evaluating feline plasma steroid profiles using liquid chromatography tandem mass spectrometry

##### 169.1.1.1 A. Watson ^1^, L. Gilligan ^2^, A. Taylor ^2^, W. Arlt ^2^, H. Syme ^3^

###### 169.1.1.1.1 ^1^ London United Kingdom; ^2^ University of Birmingham Birmingham United Kingdom; ^3^ Royal Veterinary College London United Kingdom

Mass spectrometry (MS) is used to evaluate multiple steroid profiles and in the diagnosis of human endocrinopathies. This study aims to evaluate feline plasma steroid profiles using liquid chromatography tandem MS (LC‐MS/MS) and compare to a validated aldosterone radioimmune assay (RIA).

Surplus feline serum and plasma samples were obtained from nine neutered geriatric cats (16.4 years [13.0‐20.5]). The LC‐MS/MS assay measures 25 steroid hormones. Aldosterone concentrations were measured commercially by RIA (n=8). Results are reported as median [range]. Spearman rank‐order correlation and Wilcoxon signed‐rank sum tests were applied.

Most androgens were undetectable, except 11ß‐hydroxyandrostenedione (11OHA4; 3.1nM [0.7‐5.3]). 11OHA4 is reported to be the most abundant androgen in most non‐primate species. Plasma glucocorticoid measurements included deoxycorticosterone (1.6nM [0.6‐4.4]), corticosterone (28.2nM [6.8‐58.6]), cortisol (220nM [88‐272]) and cortisone (23nM [16‐36]). Detectable steroid precursors include progesterone (1.53nM [0.4‐2.7]), 17‐hydroxyprogesterone (0.38nM [0.07‐0.80]) and 11‐deoxycortisol (1.52nM [0.15‐3.62]).

Aldosterone measured by LC‐MS/MS (382pM [11‐1033]) and RIA (635pM [131‐1140]) were not statistically different (p=0.28). However, correlation between the methods was weak (p=0.17; rho=0.55).

This study demonstrates measurement of multiple steroids within 200μl of feline plasma using LC‐MS/MS. As all cats were neutered the steroid profile reflects adrenal activity. Aldosterone measured by RIA and LC‐MS/MS were similar, although the lower limit of quantification for LC‐MS/MS (420 pM) is higher than existing reference ranges for RIA. LC‐MS/MS steroid profiling may be beneficial for endocrine conditions such as primary hyperaldosteronism where other steroid hormones may be concurrently elevated.

**Disclosures**

Disclosures to report, please report below

Alice Watson's PhD is funded by Biotechnology and Biological Sciences Research Council and Boehringer Ingelheim. Lorna Gilligham, Angela Taylor and Weibke Arlt have not conflicts of interest to declare. No funding has been obtained that is directly related to the material presented in this abstract. However, general support for the feline research group that Harriet Syme is part of, and consultancies and speakers honoraria have been received from Royal Canin, Mars Petcare, Hill's, Idexx, Boehringer Ingelheim, Vetoquinol, MSD, Pfizer, Elanco and PetPlan Charitable Trust.

## 170 ESVE‐P‐7

### 170.1 ESVE ‐ European Society of Veterinary Endocrinology

#### 170.1.1 Clinical features of primary hyperaldosteronism in 127 cats: a multicentre retrospective review

##### 170.1.1.1 V. Fabrès ^1^, I. Calvo ^2^, F. Allerton ^3^, M. Kurtz ^4^, L. Bree ^3^, E. Krafft ^5^, A. Drut ^6^, R. Dumont ^7^, F. Jolivet ^8^, F.Y. Foo ^9^, A.H. Watson ^10^, H. Syme ^10^, F. Fracassi ^11^, E. Vangrinsven ^12^, J. Bazelle ^13^, M.I. Rodríguez Piñeiro ^14^, R. Leal ^15^, G.C. Ruiz ^16^, P. Silvestrini ^17^, J. Pena‐Ramos ^18^, A. Hrovat ^19^, F. Zeugswetter ^20^, V. Neale ^21^, F. Tee ^22^, P. Valiente ^22^, M. Nolff ^23^, S. Sieber‐Ruckstuhl ^23^, S. Caulfield ^24^, S. Keyte ^24^, J. Rieder ^25^, H. Volk ^25^, R. Heilmann ^26^, A. Schreiber ^26^, A. Champetier ^27^, O. Dossin ^27^, M. Cervone ^28^, C.J. Scudder ^2^, G. Benchekroun ^4^

###### 170.1.1.1.1 ^1^ CHV Anicura‐Aquivet Eysines France; ^2^ Southfields Veterinary Specialists Laindon Essex United Kingdom; ^3^ Willows Veterinary Centre and Referral Service Shirley United Kingdom; ^4^ Ecole Nationale Vétérinaire d'Alfort Maisons Alfort France; ^5^ Université de Lyon, VetAgro Sup, campus vétérinaire Marcy l'Etoile France; ^6^ Nantes‐Atlantic College of Veterinary Medicine and Food Sciences (Oniris) Nantes France; ^7^ CHV Frégis Arcueil France; ^8^ Centre Hospitalier Vétérinaire Languedocia Montpellier France; ^9^ University of Cambridge Cambridge United Kingdom; ^10^ RVC London United Kingdom; ^11^ Department of Veterinary Medical Sciences, University of Bologna Ozzano dell'Emilia Italy; ^12^ University of Liège Liège Belgium; ^13^ Davies Veterinary Specialists Hitchin United Kingdom; ^14^ Hospital Veterinario Puchol Madrid Spain; ^15^ CIISA – Centre for Interdisciplinary Research in Animal Health – Faculty of Vete Lisbon Portugal; ^16^ Highcroft Veterinary Referrals Bristol United Kingdom; ^17^ PennVet – University of Pennsylvania Philadelphia United States; ^18^ Langford Vets Langford United Kingdom; ^19^ Pride Veterinary Centre Derby United Kingdom; ^20^ University of Veterinary Medicine Vienna Vienna Austria; ^21^ Anderson Moores veterinary Specialists Winchester United Kingdom; ^22^ North Downs Specialist Referrals Redhill United Kingdom; ^23^ Clinic for Small Animal Surgery, Vetsuisse Faculty, University of Zürich Zürich Switzerland; ^24^ Lumbry Park Veterinary Specialists, CVS Group Plc Hampshire United Kingdom; ^25^ University of Veterinary Medicine Hannover Hannover Germany; ^26^ University of Leipzig College of Veterinary Medicine Leipzig Germany; ^27^ ADVETIA Vélizy Villacoublay France; ^28^ Clinique Veterinaire Evolia L'Isle‐Adam France

Feline primary hyperaldosteronism (PHA) is an uncommon but emerging endocrinopathy with approximately 100 published cases. The aim of the study was to describe history, clinical, clinicopathological and imaging findings in a large population of cats with PHA.

Case records from 28 European referral centres between 2010 and 2022 were retrospectively reviewed and entered into an online data‐capture platform (CastorEDC). Continuous and categorical data are presented as median (range) and percentages respectively.

There were 149 entries and 127 cats were included (60 females and 67 males). The age was 12 years (3‐20). Most cats were domestic shorthair (82.6%).

Most common clinical signs were lethargy (62.2%), weakness (41.7%), decreased appetite (41.7%) and PUPD (40.3%). Neurological examination was abnormal in 45/127 cats (35.4%) with cervical ventroflexion in 19/127 (15.0%). Fifty seven of 85 cats (67.0%) had systemic hypertension, with severe hypertension (>179 mmHg) in 41/57 (71.9%).

Hypokalaemia was noted in 115/127 (90.6%) and hypernatraemia in 19/115 (16.5%) cats. Increased creatinine concentration was present in 32/121 (26.4%) cats. Aldosterone concentration was 1897 pmol/L (128‐17257). Concurrent diabetes mellitus was present in 15/127 (11.8%) cats. Increased corticosteroid secretion was diagnosed in 8/25 (32.0%) cases. Adrenomegaly was present in 118/127 (92.9%) and was unilateral in 96/118 (81.4%) cases. Histological examination was consistent with cortical carcinoma in 19/37 (51.4%) and cortical adenoma in 16/37 (48.6%) cats.

The clinical presentation of PHA in cats is non‐specific and should be investigated in middle‐aged to older cats with hypokalaemia or systemic hypertension especially when adrenal imaging shows adrenomegaly.

**Disclosures**

No disclosures to report

## 171 ESVE‐P‐8

### 171.1 ESVE ‐ European Society of Veterinary Endocrinology

#### 171.1.1 Validation and establishment of reference intervals of a chemiluminescent assay for the measurement of insulin‐like growth factor‐1 in cats

##### 171.1.1.1 A. Güssow ^1^, S. Thalmeier ^1^, R. Gostelow ^2^, J. Langenstein ^3^, G. Foerster ^3^, N. Bauer ^1^, K. Hazuchova ^1^

###### 171.1.1.1.1 ^1^ Justus‐Liebig‐University Giessen Germany; ^2^ The Royal Veterinary College London United Kingdom; ^3^ SYNLAB.vet Augsburg Germany

Hypersomatotropism (HS) is a common cause of feline diabetes. For many years, radioimmunoassay (RIA) has been the only diagnostic test to measure feline insulin‐like growth factor‐1 (IGF‐1). However, due to radiation concerns IGF‐1 RIA has limited availability and is costly. Thus, validation of assays which can be run on automated analysers such as a chemiluminescent assay (CLIA) is needed.

The aim of this study was to validate a CLIA for measurement of feline IGF‐1 on an automated analyser (Immulite 2000®, Samsung), to establish reference range, and to determine a cut‐off value for diagnosis of HS in diabetic cats.

Validation of assay performance included precision and linearity studies, performed using pooled surplus serum samples of low (≈250ng/ml), medium (≈740ng/ml) and high (≈880ng/ml) IGF‐1 concentration. Surplus serum samples of 68 diabetic cats with known IGF‐1 concentration with (n=32/68) and without HS (n=36/68) were used for method comparison with RIA serving as reference method (Spearman's rank correlation coefficient (rs), Bland‐Altman analysis). Right‐sided reference interval was determined using surplus serum of 50 healthy adult cats (≥1 and ≤10 years old; median age 4 years) presented for blood donation or health check between 2020 and 2022 using a robust method after Box‐Cox transformation. Cut‐off for diagnosis of HS was established in the group of 68 diabetic cats using receiver operating characteristic (ROC) analysis.

The intra‐assay coefficient of variation (CV) was 2.5%, 4.7% and 1.6%, and the inter‐assay CV was 5.1%, 5.6% and 4.1% for a sample with low, medium and high IGF‐1 concentration, respectively. Linearity was excellent (R^2^>0.99) for IGF‐1 concentrations ranging between 218 – 888 ng/ml. There was a good correlation between CLIA and RIA (rs=0.97), with a mean negative bias for CLIA of 24.5%. The upper limit of reference interval was 670ng/ml (90% confidence interval 575 to 761). The ROC analysis showed an area under the curve of 0.94, with the best cut‐off for diagnosis of HS at 746ng/ml (sensitivity, 84.4%; specificity, 97.2%).

The current study showed good performance of a CLIA for measurement of feline IGF‐1 concentration, established a reference interval in healthy cats, and a cut‐off for diagnosis of HS in diabetic cats.

**Disclosures**

Disclosures to report, please report below

The study was financially supported by and the measurement were performed at Synlab GmbH

## 172 ESVE‐P‐9

### 172.1 ESVE ‐ European Society of Veterinary Endocrinology

#### 172.1.1 Dopamine and somatostatin receptors and filamin A expression in normal pituitaries and corticotroph adenomas in dogs

##### 172.1.1.1 S. Golinelli ^1^, S Galac ^2^, B Meij ^2^, H Kooistra ^2^, F Fracassi ^3^, F Steenbeek ^2^, K Sanders ^2^

###### 172.1.1.1.1 ^1^ University of Bologna Bologna Italy; ^2^ University of Utrecht Utrecht Netherlands; ^3^ University of Bologna Bologna Italy

Dopamine agonists and somatostatin analogs have been investigated as potential pituitary‐targeting drugs in both human and canine pituitary‐dependent hypercortisolism (PDH). Information about the expression of dopamine and somatostatin receptors subtypes in canine corticotroph adenomas (CAs) is sparse. In humans, the actin‐binding protein filamin A (FLNA) is required for somatostatin receptor 2 (SSTR2) and 5 (SSTR5) and dopamine receptor 2 (DRD2) expression and signaling in pituitary tumors, thereby playing a role in tumor responsiveness to somatostatin receptors ligands and dopaminergic drugs. The present study aimed to evaluate the mRNA expression of all the different dopamine and somatostatin receptor subtypes and FLNA in canine normal adenohypophysis (NAs) and CAs.

Tissues from nine NAs and 32 CAs were included in the study. The CAs were collected from dogs that were diagnosed with PDH and underwent transsphenoidal hypophysectomy between 2015 and 2021. The NAs were collected from healthy dogs that were euthanized for reasons unrelated to this study. The gene expression levels of dopamine and somatostatin receptor subtypes and FLNA were evaluated with RNA sequencing.

mRNA of the DRD2 and the SSTR2 was detected in all NAs and in 31 out of 32 CAs (97%). None of the other annotated dopamine and somatostatin receptor subtypes were found to be expressed in either NAs or CAs. The NAs showed significantly higher expression of DRD2 (P=8.3x10^‐5^) and SSTR2 (P=1.5x10^‐3^) in comparison with the CAs. FLNA was expressed in all samples and showed no significant difference in expression levels between NAs and CAs (P=0.64). The DRD2 mRNA expression was significantly positively correlated with SSTR2 mRNA expression (r=0.76, P=9.4x10^‐9^).

This study shows that DRD2 and SSTR2 were expressed in the majority of CAs, albeit at lower levels than in NAs. FLNA, an acting‐binding protein that is required for SSTR2 and DRD2 expression and signaling, was expressed in all CAs, indicating that these signaling pathways are potentially activated. Interestingly, DRD2 and SSTR2 expression levels were strongly correlated, highlighting the possibility of combined targeting of these receptor subtypes.

**Disclosures**

Disclosures to report, please report below

Federico Fracassi: Financial support: Dechra, MSD Speaking & consultancies: Boehringer Ingelheim, Dechra, MSD, Royal Canin, Hill's, Nestlé Purina, La Vallonea. Stefania Golinelli: Financial support: PhD scholarship funded by Dechra Speaking & consultancies: Dechra

## 173 ESVE‐P‐10

### 173.1 ESVE ‐ European Society of Veterinary Endocrinology

#### 173.1.1 Laparoscopic versus open adrenalectomy in 70 dogs with functional or hormonally silent adrenal tumors

##### 173.1.1.1 K.L. Bokhorst, S. Galac, H.S. Kooistra, B. Nimwegen

###### 173.1.1.1.1 Utrecht University Utrecht Netherlands

Adrenalectomy is the treatment of choice in hormone‐secreting adrenal tumors to reverse clinical signs associated with hormone excess and to avoid complications of uncontrolled tumor growth. Surgery is also indicated in hormonally silent adrenal tumors when malignancy is suspected. In recent years, laparoscopic adrenalectomy gained popularity, however, clinical studies on larger groups of dogs including their endocrine diagnoses and outcome are still scarce. In this retrospective study the hormone analyses, perioperative‐ and recurrence data of 70 dogs that underwent adrenalectomy by laparoscopy or laparotomy between 2008 and 2022 are described.

Diagnosis was based on history, clinical signs, endocrine testing including measurement of plasma endogenous ACTH, (nor)metanephrines and aldosterone concentrations, and advanced diagnostic imaging. Endocrine diagnoses were naturally occurring hypercortisolism (n=52), pheochromocytoma (n=8) and hormonally silent ‘incidentalomas’ (n=10). Median age at adrenalectomy was 10 years (range 5.8‐13 years). Most dogs (n=55) were neutered (25 male, 30 female), 15 were intact (12 male, 3 female).

Laparoscopic adrenalectomy was performed in 45 dogs (median weight 13.9 kg, range 5.1‐50.3) of which 3 cases were converted to laparotomy. In the remaining 25 dogs (median weight 18.0 kg, range 6.0‐43.6) a laparotomy approach was used. Bilateral adrenalectomy was performed in 8/70 dogs (6 laparoscopic, 2 laparotomy). Based on preoperative imaging, median maximal adrenal tumor dimension was 2.4 (range 1.0‐5.2) and 3.2 cm (range 1.6‐9.9) in the laparoscopy and laparotomy group, respectively (p=0.029). Surgical time of laparoscopic and open adrenalectomy did not differ significantly (p=0.147 and p=0.102 for unilateral and bilateral cases, respectively). Histopathology of hormonally silent adrenal tumors revealed a cortical adenoma in 7/10, carcinoma in 2/10 and unspecified adrenocortical tumor in 1/10 dogs. Forty‐three out of 45 dogs in the laparoscopy group and 24/25 dogs in the laparotomy group survived to discharge. Median duration of hospitalization was shorter after laparoscopic (1 day, range 1‐11) versus laparotomic unilateral adrenalectomy (2 days, range 1‐4; p=0.012). Relapse, defined by recurrence of clinical signs of hypercortisolism or catecholamine‐excess and/or regrowth of adrenal tumor or metastasis, was not different in the laparoscopy (7/43) versus laparotomy group (4/24; p=1.00).

This study shows a favorable outcome of laparoscopic adrenalectomy in dogs with various endocrinopathies due to adrenal tumors. Based on shorter hospitalization time and comparable long‐term outcome in dogs treated with laparoscopic adrenalectomy, this is considered the preferred technique whenever clinically possible.

**Disclosures**

No disclosures to report

## 174 ESVE‐P‐11

### 174.1 ESVE ‐ European Society of Veterinary Endocrinology

#### 174.1.1 Trilostane treatment for feline hypercortisolism: Latin America multicenter study, 43 cases (2012‐2022)

##### 174.1.1.1 D. Miceli, F Tavares, M Zapata Montoya, J García, M Peña, K Weege, A Muschner, C Vecino, A Mierowski, A Schnabel, A Pöppl

###### 174.1.1.1.1 Caba Argentina

Spontaneous hypercortisolism (HC), or Cushing's syndrome, is a rare condition in cats (*Felis catus*). Feline HC is characterized by presence of diabetes mellitus (DM), with variable degrees of insulin resistance. Trilostane is a competitive 3‐β hydroxysteroid dehydrogenase inhibitor with high safety and efficacy in canine HC. Large case series of feline HC and studies assessing the use of trilostane in cats are lacking. The aim of this study was to evaluate clinical features, clinicopathologic findings, diagnostic imaging, safety and efficacy of trilostane treatment in cats with HC. In this multicenter descriptive retrospective study, fourty‐three (n=43) client‐owned cats were diagnosed with spontaneous HC. All cats received an initial lower dose of trilostane (0.3‐2.9 mg/kg q12h, PO) than previously reported. Values were expressed as mean±SD or, median and range (p<0.05). Twenty‐six cats were male (60%) and 17 were female (40%); mean age was 10.4±2.8 years; mean body weight was 5.3±1.5 kg. Thirty‐six cats were domestic shorthairs, four Persian, two domestic longhairs and one Russian blue. Thirty‐five of 43 (81%) cats had pituitary‐dependent HC, whereas eight of 43 (19%) cats had adrenal‐dependent HC (1/8 bilateral adrenal tumor). The mean initial and final dose of trilostane were 1.3±0.6 and 1.9±1.4 mg/kg q12h (p<0.01). Nineteen of 43 cases (44%) required an increase in the trilostane dose, 20 of 43 (47%) maintained the dose and four of 43 (9%) reduced the dose. Most cats (53%) had an improvement in the clinical signs after trilostane treatment. Thirty‐four of 43 (79%) cats had concurrent DM. The mean initial and final dose of insulin were 0.5±0.4 and 0.6±0.6 IU/kg q12h (p<0.4). Sixteen of 34 cases (47%) improved the diabetic control, nine cases (26%) required a reduction in insulin dose and five cases (15%) achieved diabetic remission after trilostane treatment. Adverse effects were observed in 19% of cases, and included: reduced appetite (3/43), diarrhea (2/43), vomiting (2/43), neurological signs (1/43), and increased libido (1/43). Six of 34 cats (18%) had plantigrade stance. The most common complications were: chronic kidney disease (13/43) and pancreatitis (5/43). The median survival time was 657 days (60‐2555). This is the largest study to date to describe cases of feline HC. This study shows that low‐doses of trilostane could improve clinical signs of HC and DM control in 53% and 47% cases, respectively. In addition, trilostane treatment allows remission of DM in 15% of diabetic cats with HC.

**Disclosures**

No disclosures to report

## 175 ESVCN‐P‐1

### 175.1 ESVCN ‐ European society of Veterinary & Comparative Nutrition

#### 175.1.1 The perception of Portuguese pet owners about veterinary approach to nutrition and weight management in companion animals

##### 175.1.1.1 M. Pires Gonçalves ^1^, R. Leal ^1^, M. Hervera ^2^

###### 175.1.1.1.1 ^1^ CIISA ‐ Centre for Interdisciplinary Research in Animal Health LISBOA Portugal; ^2^ Expert Pet Nutrition Zurich Switzerland

Obesity numbers in humans and pets keep rising. While it is easy to consider the owner as the main influencer for obesity in dogs and cats, it is also important to evaluate the performance of veterinary teams and the owners' perception of this. This study aims to assess the impression of Portuguese pet owners about how the veterinary community approaches nutrition and weight management in pets.

An observational cross sectional survey developed for pet owners was implemented on waiting rooms of Portuguese veterinary practices. The study comprised 21 questions covering good practices in nutrition by veterinarians, advice from clinicians on diet and exercise, and whether clients were aware of the existence of weight management programmes and if they would consider this service if their pet was overweight.

A total of 432 answers were obtained from 292 (68%) veterinary hospitals and 140 (32%) clinics. Based on the recommendations of WSAVA and AAHA Nutritional and Weight Management Guidelines, it was observed that 93% of the animals were always or frequently weighed in consultation. However, the body condition score was only 49% routinely evaluated and a nutritional assessment was regularly performed in just 27% of the cases. Nevertheless, 97% of the pet owners surveyed, wanted their veterinarian to make a food recommendation and 92% a physical exercise plan. The majority (93%) of pet owners agreed that veterinarians have warned them about the risk of obesity in pets, mainly in paediatric visits or at the time of spay/neuter surgery. However, 65% of the respondents were unaware of the existence of weight management programmes for pets, and only 9% of the remaining mentioned that this service existed in the practice they visit. Lastly, a total of 82% of pet owners admit that if their animal was overweight they would resort to the help of this health service.

This study supports that pet owners are being informed by their veterinarians about obesity in pets. However, procedures like body condition score and nutritional assessment, which are needed to manage overweight animals, are not being performed often enough. Most owners reinforce the need of food and physical exercise recommendations by clinicians. Although veterinarians acknowledge that about half of the animals they observe are overweight, the majority of the respondents were not aware of the existence of weight management programmes, which raises the question if obesity is being properly managed.

**Disclosures**

Disclosures to report, please report below

This work was supported by FCT ‐ Fundação para a Ciência e Tecnologia IP, grant UIDB/00276/2020.

## 176 ESVCN‐P‐2

### 176.1 ESVCN ‐ European society of Veterinary & Comparative Nutrition

#### 176.1.1 Different starch to protein ratios in kibble diets fed ad libitum on body weight control, body composition, water turnover and physical activity in neutered cats living in homes

##### 176.1.1.1 C. Goloni, L. Pacheco, L. Warde Luis, S. Souza Theodoro, L. Bassi Scarpim, C. Torres, A. Cavalieri Carciofi

###### 176.1.1.1.1 São Paulo State University ‐ UNESP Jaboticabal Brazil

The intake of diets with different starch to protein ratios was compared in neutered cats housed in homes. Male (M) and female (F), obese (OB) and non‐obese (NO) client owned cats were fed *ad libitum* for four months kibble diets high in starch (HS: Starch 40%, protein 38%; on dry matter basis) or high in protein (HP: Starch 20%, protein 52%), in a *cross‐over* design. Physical activity was evaluated with an accelerometer fixed to chest collar previously adapted to cats, and body composition (BC), energy expenditure (EE) and water turnover (WT) were evaluated by the doubly labeled water method. Results were compared in a 2 diets x 2 sex x 2 body conditions factorial arrangement, totally 8 treatments, and submitted to variance analysis, Tukey test and Pearson correlation (P≤0.05 as significant; P≤0.10 as trend). A total of 30 cats, belonged to 23 owners finished the project: F‐NO with 2.9±2.1 years (n=9); F‐OB with 4.2±1.6 years (n=7); M‐NO with 2.0±1.1 years (n=9); M‐OB with 4.5±2.6 years (n=5). The mean age of the groups differed (P<0.05), and then age was used in the statistical analysis as a co‐variate. Cats fed HS diet maintained a constant body weight (BW) along the 4 months of diet intake (P>0.05). However, the lean mass (LM) tended to reduce in F‐OB (P=0.07), which presented a numerically lower EE (324±7 kJ/kg^0.67^/day) than the other groups. This may limited food and protein intake (6.84±0.38 g of CP/kg^0.67^/day), explaining the tendency of LM reduction. The intake of HP diet induced an increase on cats BW and LM (P<0.05), but fat mass also increased 17% on F‐NO (P=0.04) and tended to increase 7% in F‐OB (P=0.06). The EE tended to be higher in M (351±8 kJ/kg^0.67^/day) than F (330±8; P=0.06), was not affected by diet or BC, and reduced as the age of the cat's increase (R^2^=0.44; P<0.01). The EE increased as the physical activity increase (R^2^=0.58; P<0.01). Physical activity was negatively correlated with age (R^2^=0.51; P<0.01). The WT was higher for HP diet (P<0.01) and increased as the EE increases (R^2^ 0.65; P<0.01; 0.151±0.004 mL/kJ of EE). The HS diet favored BW control in *ad libitum* feeding. Caution is necessary to balance protein content on diets of obese females with more than 4 years old, as this cat group may have low energy expenditure, limiting food and amino acid intake resulting in lean mass loss.

**Disclosures**

No disclosures to report

177

### 177.1 Research Reports ECVIM‐CA Congress 2022

#### 177.1.1 Vaccination in dogs and cats – owner compliance, antibody prevalence, and necessity of revaccination

##### 177.1.1.1 MB Bergmann, KH Hartmann

###### 177.1.1.1.1 Centre for Clinical Veterinary Medicine, LMU Munich Munich Germany

This research investigated (1) the vaccination compliance of dog and cat owners, (2) the immune response against infections with viral vaccine core‐components, and the benefit of regular revaccinations in both, healthy animals and those with immunocompromising diseases, (3) and the quality of four canine pre‐vaccination antibody point‐of care tests (POCTs).

Data on owners' vaccination compliance were obtained by online questionnaires; a linear logistic regression model was used to identify factors that could improve compliance. Antibodies against canine distemper, adeno‐, and parvovirus (CDV, CAV, CPV), feline panleukopenia‐, herpes‐, and calicivirus (FPV, FHV, FCV) were measured in sera of adult animals (not vaccinated within one year before enrollment) before (day 0) and after regular revaccinations with modified live viruses (except FCV being an inactivated component) by virus neutralization (VN) or haemagglutination inhibition. A ≥ four‐fold titer increase by day 28 was defined as vaccination response. Quality of POCTs detecting anti‐CPV, ‐CDV‐ and ‐CAV antibodies was assessed in comparison to VN; specificity was considered most important.

According to owners, vaccination status of cats and dogs was considered “up‐to‐date” in 78% and 47% of animals, respectively. Among dog owners, 83% had a positive attitude towards vaccination in general, 14% were ambivalent, and 3% refused vaccinations. Veterinary advice on vaccination was significantly associated with vaccination status in both species. Response after revaccination against CDV, CAV‐1, FPV, FCV, FHV was seen in only 0%, 6%, 48%, 14%, and 8% of healthy animals. Lack of response was common in animals that already had preexisting antibodies on day 0 (which was the case in 95%, 93%, 64%,62%, and 41%, respectively) and significantly associated with preexisting antibodies in FPV (p<0.001); mild transient vaccine‐associated adverse events (VAAEs) were common in dogs (36%) and cats (10%) and associated with FPV antibody response. Antibodies against CPV in dogs treated for hyperadrenocortizism or hypothyreosis, and against FPV in cats infected with retroviruses were comparable to those of healthy animals before and after parvovirus revaccination; risk of VAAEs was not significantly higher compared to that of healthy animals, although 55% of dogs with hyperadrenocortizism showed VAAEs. For detection of anti‐CPV, anti‐CDV, and anti‐CAV antibodies, specificity of POCTs ranged between 73‐98%, 8‐59%, and 88‐90%, respectively.

Veterinary advice can help to improve vaccination compliance. Necessity of revaccination against CDV, CAV, and FPV should be determined by antibody detection. Available POCT should be modified to increase specificity to safely detect dogs that might be unprotected.

**Disclosures**

No disclosures to report

178

178.1

#### 178.1.1 A multimodal investigation into the suitability of plant‐based diets for companion dogs and cats

##### 178.1.1.1 SD Dodd, AV Verbrugghe

###### 178.1.1.1.1 Ontario Veterinary College, University of Guelph Guelph Canada

Dogs and cats were domesticated from wild carnivores, though a small proportion of pet owners feed exclusively plant‐based diets to the dogs and cats in their care. The impact to the health of dogs and cats from exclusion of animal‐derived ingredients from their diet is not currently well understood. The research presented here included three studies investigating the sufficiency of plant‐based diets for dogs and cats: a survey of owner perception of health and wellbeing in dogs and cats fed plant‐based or animal‐containing diets, analysis of nutrients in commercial plant‐based diets, and a diet trial comparing an experimental plant‐based diet to a conventional commercial animal‐containing diet in client‐owned dogs. Survey responses from 1,413 dog owners and 1,325 cat owners across Canada and the USA revealed similar results for both dogs and cats. Owners reported fewer perceived health disorders, specifically with respect to gastrointestinal and hepatic conditions. More owners of cats fed plant‐based diets believed their cats to be in ideal body condition and very good health. Lifespan of dogs fed plant‐based diets appeared to be longer, no difference in lifespan was reported for cats. Nutritional analyses of 26 commercial plant‐based diets available in Ontario, Canada, revealed sulfur amino acids, taurine, arachidonic acid, EPA + DHA, calcium, phosphorus and vitamin D consistently lower than American and European industry recommendations. Four products labelled for adult dogs met minimum recommendations for canine maintenance, no diet met minimum recommendations for feline maintenance or growth of puppies or kittens. In the diet trial, all 61 dogs completing the diet trial maintained body weight and composition, as well as measured indices of health and wellness. Reductions in platelet count, branched‐chain amino acids, creatinine, blood urea nitrogen, cholesterol and ratio of omega‐6 to omega‐3 fatty acids were detected in dogs fed the plant‐based diet, though values were maintained within the normal reference ranges. A shift from vitamin D3 to D2 metabolites occurred, though total vitamin D analogues, ionized calcium and bone mineralization were unaffected. The findings of this research provide veterinarians with insight into the perspectives of pet owners to improve communications regarding pet health and nutrition, and demonstrate to nutritionists areas requiring improvement in this industry niche. Finally, further investigations of impacts of plant‐based diets on canine health are proposed.

**Disclosures**

Disclosures to report, please report below

Dr. Sarah Dodd is the owner/operator of Dodd Veterinary Services and provides nutritional consultation to private and industry clients. Dr. Dodd also serves on the Advisory Board for Dog Child (previously The Animals Kitchen) and consults part‐time for Vivus Pets Inc and Veterinary Nutritional Consultation (petdiets.com). Dr Verbrugghe is the Royal Canine Veterinary Diets Endowed Chair in Canine and Feline Clinical Nutrition at the Ontario Veterinary College, University of Guelph, serves on the Health and Nutrition Advisory Board for Vetdiet and has received, honoraria and research funding from various pet food manufacturers and ingredient suppliers.

